# Zirconium-catalyzed asymmetric Kabachnik–Fields reactions of aromatic and aliphatic aldehydes†

**DOI:** 10.1039/d1sc03222d

**Published:** 2021-08-03

**Authors:** Yijing Dai, Li Zheng, Debarshi Chakraborty, Babak Borhan, William D. Wulff

**Affiliations:** Department of Chemistry, Michigan State University East Lansing MI 48824 USA wulff@chemistry.msu.edu

## Abstract

An effective catalyst has been developed for the three-component reaction of aldehydes, anilines and phosphites in an asymmetric catalytic Kabachnik–Fields reaction to give α-aminophosphonates. A catalyst was sought that would give high asymmetric inductions for aromatic and, and more particularly, for aliphatic aldehydes since there has not previously been an effective catalyst developed for this class of aldehydes. The optimal catalyst is prepared from three equivalents of the 7,7′-di-*t*-butylVANOL ligand, one equivalent of *N*-methylimidazole and one equivalent of zirconium tetraisopropoxide. This catalyst was most efficient in the presence of 10 mol% benzoic acid. Optimal conditions for aryl aldehydes required the use of 3,5-diisopropyl-2-hydroxyaniline and gave the aryl α-aminophosphonates in up to 96% yield and 98% ee over 11 different aryl aldehydes. The best aniline for aliphatic aldehydes was found to be 3-*t*-butyl-2-hydroxyaniline and gave the corresponding phosphonates in up to 83% yield and 97% ee over 18 examples. The asymmetric inductions for aliphatic aldehydes were comparable with those for aromatic aldehydes with a mean induction of 90% ee for the former and 91% ee for the latter. The best method for the liberation of the free amine from the aniline substituted α-aminophosphonates involved oxidation with *N*-iodosuccinimide.

## Abstract 2

An effective catalyst has been developed for the three-component reaction of aldehydes, anilines and phosphites in an asymmetric catalytic Kabachnik–Fields reaction to give α-aminophosphonates.

## Graphical abstract

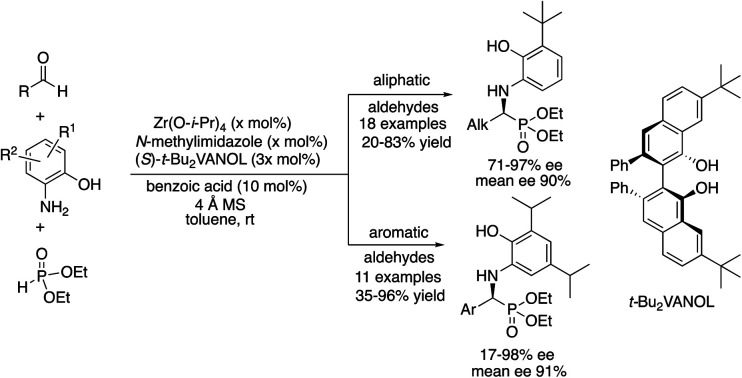

## 1. Introduction

**Scheme 1:**
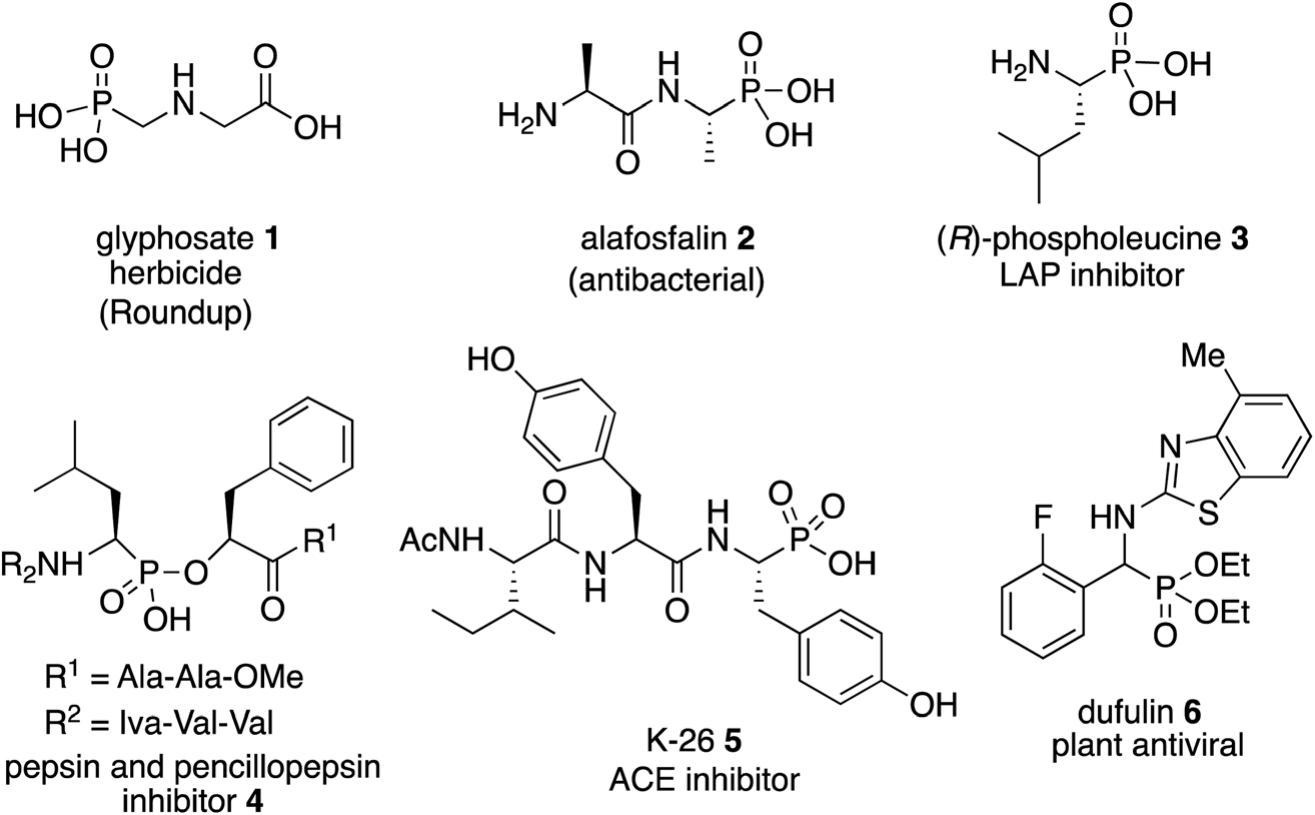
Examples of biologically active α-aminophosphonic acids.

The most important analogs of α-amino acids are α-aminophosphonic acids and examples include both natural and synthetic derivatives.^1^ Among their biological activities many are related to their ability to inhibit enzymes that are involved in cleavage of peptide bonds since they can serve as transition-state analogs of a tetrahedral intermediate formed from an amide carbonyl during hydrolysis.^1,2^ Examples of biologically active α-aminophosphonic acids and esters are shown in Scheme 1 and include glyphosate **1** (Roundup),^3^ the antibacterial alafosfalin **2** (the other three isomers are less active)^4^ and phospholeucine **3** which is a leucine aminopeptidase inhibitor (the (*S*)-enantiomer is 10^3^ times less active than the (*R*)-enantiomer).^5^ The phospholeucine **3** is a key component of, and plays an important role in, the activity of the pepsin and penicillopepsin inhibitor **4**.^6^ The naturally occurring phosphotyrosine tripeptide K-26 **5** is an ACE inhibitor with comparable activity to captopril,^1*b*,7*a*^ although analogs of **5** were found to be more active.^7*b*,*c*^ Dufulin **6** has been widely used to treat viral diseases in agricultural crops in China.^8^

**Fig. 1:**
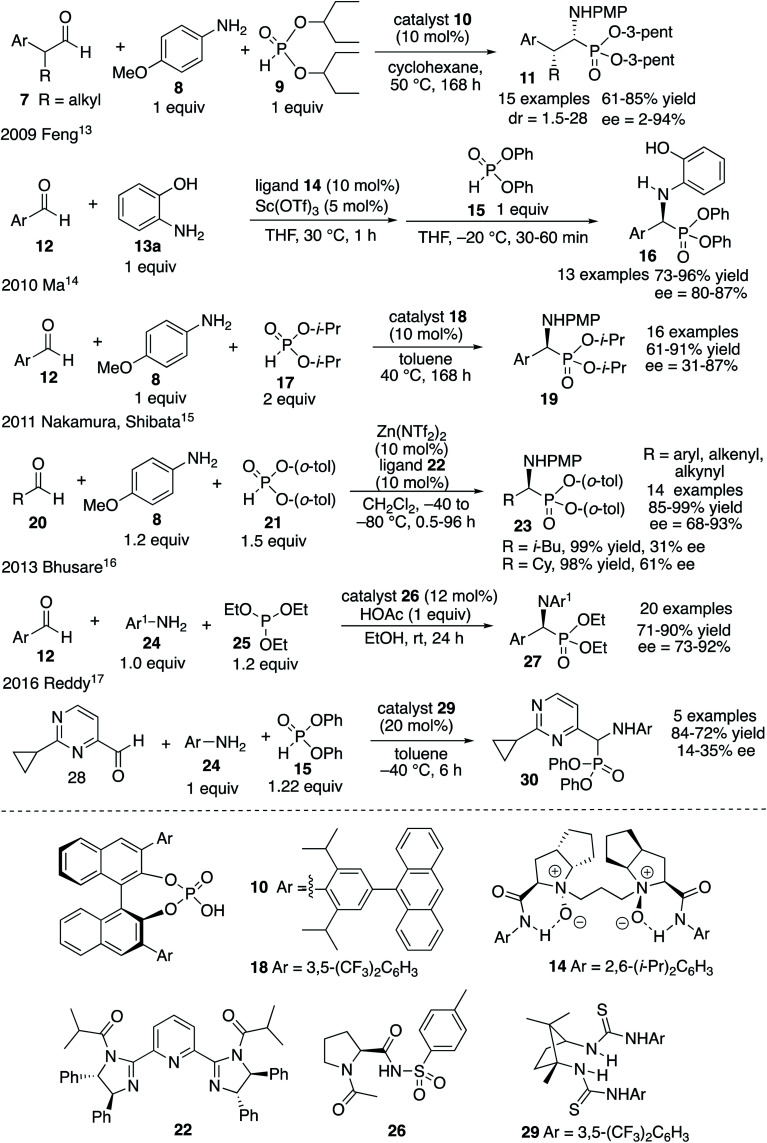
Reported examples of the asymmetric catalytic Kabachnik–Fields reaction.

The most common methods for the synthesis of α-aminophosphonic acids involve the two component reaction of an imine and a phosphite (Pudovik reaction^9^) and the three component reaction of an aldehyde, an amine and a phosphite (Kabachnik–Fields reaction^10,11^). Given the level of difficulty it is not surprising that the three component Kabachnik–Fields reaction has been the more difficult of the two to develop asymmetric catalytic versions.^11^ The first catalytic asymmetric Kabachnik–Fields reaction was reported by List and coworkers in 2008 (Fig. 1).^12^ They developed the BINOL hydrogen phosphate catalyst **10** for the three component reaction to give α-aminophosphonic esters **11** with high enantioselectivity. Interestingly, this reaction also involved a dynamic kinetic resolution of the aldehyde, yielding product **11** with high diastereoselectivity. The transformation was limited to aryl acetaldehydes and the reaction times were quite long. Feng and coworkers reported that a scandium catalyst with the bis-amine oxide ligand **14** gives good asymmetric inductions with aryl aldehydes and aniline **13a** with the diphenyl phosphite **15**.^13^ However, this was not actually a Kabachnik–Fields reaction since the imine was generated first at 30 °C and then the phosphite was added at −20 °C. A second example involving a BINOL hydrogen phosphate catalyst was published by Ma and coworkers in 2010 and gave the α-aminophosphonate **19** in low to high asymmetric induction with 16 different aromatic aldehydes.^14^ In 2011 Nakamura and Shibata and coworkers reported the success of a zinc-bis-imidazolidine catalyst in the reaction of 16 different aldehydes with the aniline **8** and the bis-*o*-tolylphosphite **21**.^15^ The α-aminophosphonate diester **23** was obtained in 68–93% ee with aryl aldehydes but the two alkyl aldehydes that were examined gave low to moderate induction (31–61% ee). The organocatalyst **26** has been reported by Bhusare and coworkers to give good to excellent asymmetric inductions in the Kabachnik–Fields reaction for a variety of aryl aldehydes.^16^ This reaction is unusual in that, instead of a phosphite diester, a phosphite triester was employed in this Kabachnik–Fields reaction where one of the ethyl groups was cleaved during the reaction to give the phophonate diester **27**. Finally, Reddy and coworkers reported the use of the bis-thiourea catalyst **29** to give the α-aminophosphonate **30** from the aldehyde **28** in low enantioselectivity.^17^

To summarize, the existing methods for the asymmetric catalytic Kabachnik–Fields reaction work well only for aromatic aldehydes. The only two examples with aliphatic aldehydes come from the work of Nakamura and Shibata who were able to prepare the α-aminophosphonate **23** from cyclohexanecarboxaldehyde and isovaleraldehyde in 61% and 31% ee, respectively.^15^ Given that α-aminophosphonates are largely of interest as analogs of α-amino acids, and that most α-amino acids do not contain aromatic groups, it is clear that improved methods for the catalytic asymmetric Kabachnik–Fields reaction are needed.

## 2. Results and discussion

**Scheme 2:**
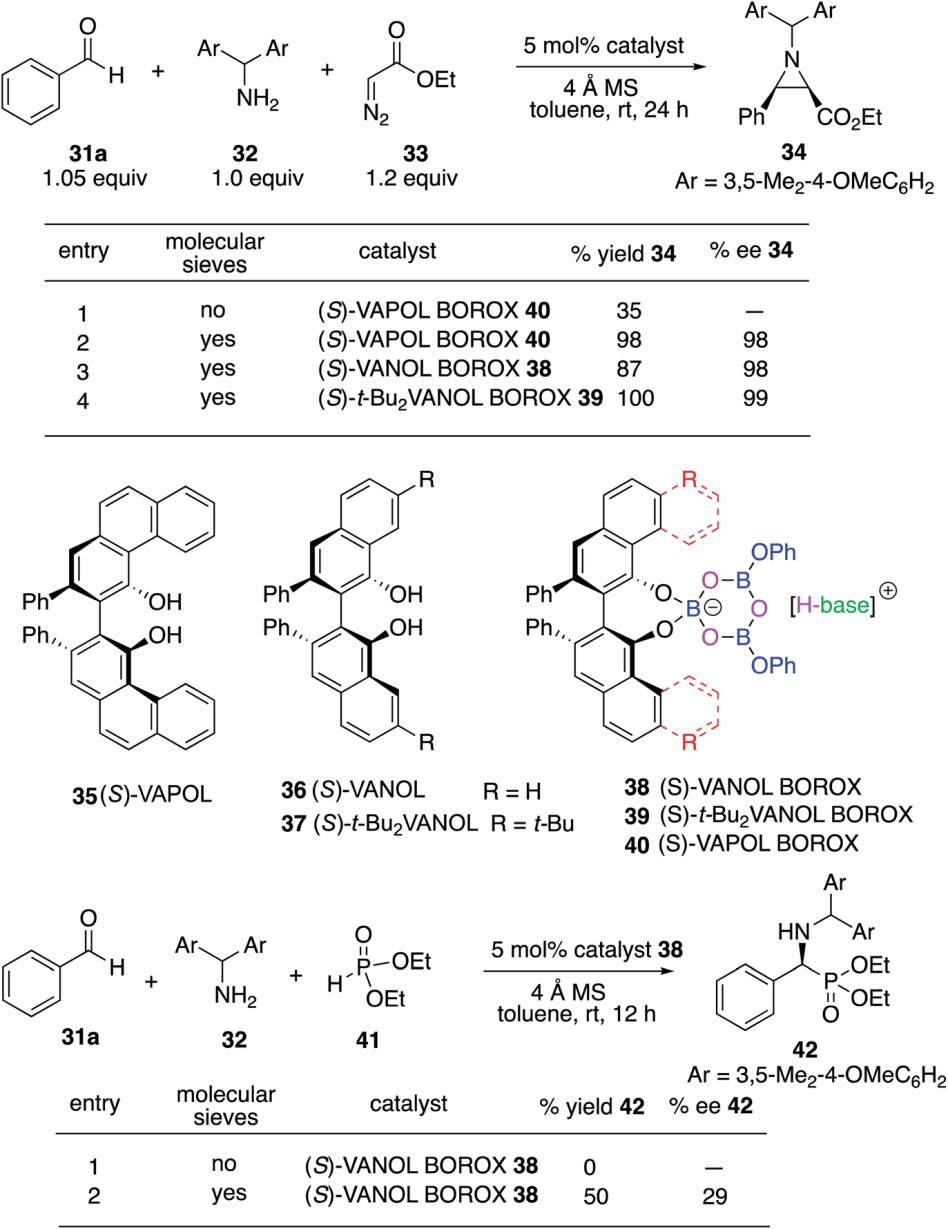
Previous three-component aziridination with BOROX catalysts.

We decided to begin the search for new catalysts for the Kabachnik–Field reaction by examining aromatic aldehydes since it was the expectation that reaction optimization would not be as challenging. We had previously reported a three-component reaction for the catalytic asymmetric synthesis of aziridines from an aldehyde, amine and an α-diazoacetate (Scheme 2).^18^ The catalyst was a boroxinate (BOROX) derived from either the VAPOL or VANOL ligands and both gave the aziridines **34** in 98% ee. The reaction gives low conversion in the absence of molecular sieves. This was not surprising given that an imine is generated *in situ*. Later it was found that the BOROX catalyst **39** generated from the *t*-Bu_2_VANOL ligand **37** gives the highest yields and asymmetric inductions.^19^ Unfortunately, the success with the BOROX catalysts did not transfer from the three component aziridination reaction to the three component Kabachnik–Fields reaction since the VANOL-BOROX catalyst **38** only gave the α-aminophosphonate **42** in 29% ee.

**Scheme 3:**
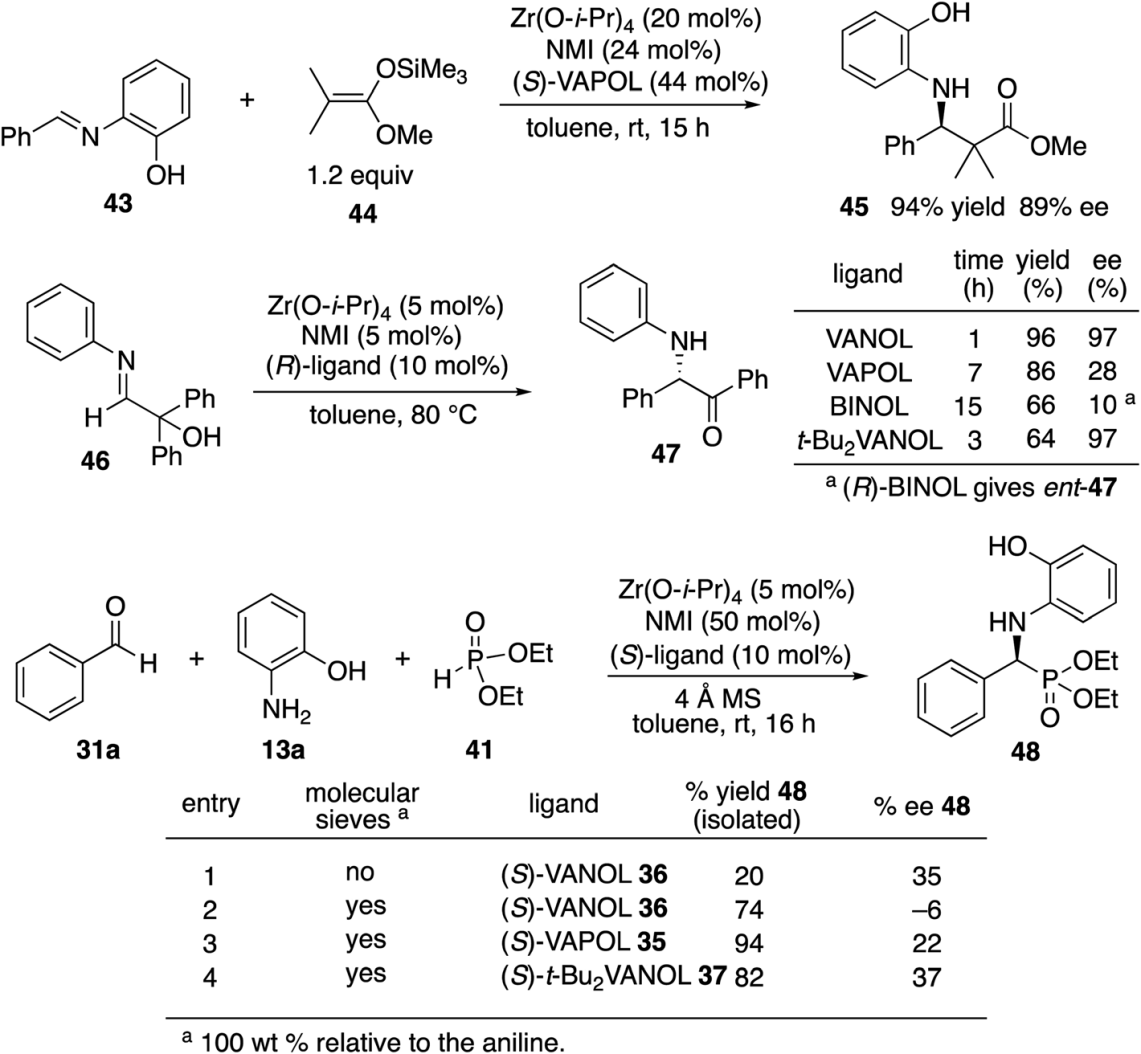
Initial success with zirconium catalysts.

Although not three-component reactions, we have had success with zirconium catalysts in the asymmetric catalytic transformation of imines in Mannich reactions^20^ and α-imino rearrangements^21^ (Scheme 3). In each case the catalyst is generated by combining zirconium tetraisopropoxide, a vaulted biaryl ligand and *N*-methylimidazole. This catalyst type was initially screened in the reaction of benzaldehyde, 2-hydroxyaniline **13a** and diethyl phosphite **41** with three different vaulted biaryl ligands (Scheme 3). In the absence of molecular sieves, only a low 20% yield of the α-aminophosphonate **48** was obtained with the VANOL ligand **36** in 35% ee (entry 1). The yield was greatly increased in the presence of molecular sieves, but the product was nearly racemic (entry 2). The yield was excellent with a catalyst generated from the VAPOL ligand **35** but the asymmetric induction was low (22% ee) (entry 3). The best result was achieved with the 7,7′-di-*t*-butylVANOL ligand **37** giving **48** in 82% yield and 37% ee (entry 4).

In the Mannich reaction with the zirconium catalyst indicated in Scheme 3, we had observed that higher asymmetric inductions and yields were obtained with the 3,5-dimethyl-2-hydroxyaniline **13b**.^20^ This was also the case in the present study where the asymmetric induction of the α-aminophosphonate increased from 37 to 67% ee (Table 1, entries 1 and 2). Further studies revealed that if the amount of molecular sieves was increased from 100 to 200 wt% relative to the aniline, the asymmetric induction with aniline **13b** could be increased from 67 to 82% ee (entries 2 *vs.* 3). Additional amounts of molecular sieves were not beneficial (see the ESI†). Further optimization was possible by screening other 3,5-disubstituted-2-hydroxyanilines in this reaction. The α-aminophosphonate **51** could be obtained in 90% ee with the 3,5-diisopropyl-2-hydroxyaniline **13c** (entry 5). We had also observed that if benzaldehyde **31a** was not freshly distilled, the yield improved. Assuming that the difference here was due to benzoic acid, this prompted a study into the effect of added benzoic acid. Here the yield increased by 10% upon the addition of 5 mol% benzoic acid (entries 5 *vs.* 6), and the induction was slightly higher with 10 mol% benzoic acid but dropped with 100 mol% benzoic acid (entries 5 to 8). This effect of benzoic acid was not noted for the 3,5-dimethyl-2-hydroxyaniline **13b** (entries 3 and 4). The di-*n*-butylaniline **13d** did not give as high an asymmetric induction as the di-i-propylaniline **13c** (82% *vs.* 90%, entries 5 *vs.* 9) and the highest induction was observed with the di-*t*-butylaniline **13e** (95%, entry 10). Unfortunately in the latter case, the reaction was very slow and in the same time period, only a 12% yield of **53** was isolated. The yield could only be slightly recovered upon the addition of benzoic acid (entries 10 *vs.* 13). The yield could be increased to 52% with a reaction temperature of 60 °C, but the induction fell to 89%. The absolute configuration of the phosphonate **51** from the (*S*)-catalyst was determined to be (*S*)-**51** after deprotection to the free amine as indicated in Scheme 6.

**Table 1:** The effect of substituents on aniline **13** on the Kabachnik–Fields reaction^a^

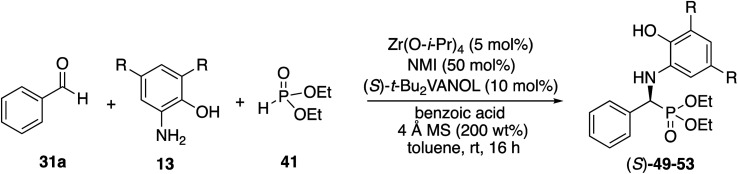							
Entry	R	Aniline	Temp (°C)	Benzoic acid (mol%)	Product	% Yield^b^	% ee
1	H^c^	**13a**	rt	0	**49**	82	37
2	Me^c^	**13b**	rt	0	**50**	55	67
3	Me	**13b**	rt	0	**50**	87	82
4	Me	**13b**	rt	10	**50**	87	80
5	i-Pr	**13c**	rt	0	**51**	76	90
6	i-Pr	**13c**	rt	5	**51**	86	92
7	i-Pr	**13c**	rt	10	**51**	80	94
8	i-Pr	**13c**	rt	100	**51**	59	77
9	*n*-Bu	**13d**	rt	0	**52**	73	−82^d^
10	*t*-Bu	**13e**	rt	0	**53**	12	95
11	*t*-Bu	**13e**	40	0	**53**	41	92
12	*t*-Bu	**13e**	60	0	**53**	52	−89^d^
13	*t*-Bu	**13e**	rt	10	**53**	17	95

a Unless otherwise specified, the reactions were carried out on 0.1 mmol of aldehyde with 1.0 equiv. of aniline and 1.0 equiv. of phosphite and with 200 wt% of 4 Å MS relative to the aniline.

b Isolated yield.

c 100 wt% MS relative to the aniline.

d (*R*)-*t*-Bu_2_ VANOL ligand was used and *ent*-**52**/**53** was obtained.

**Scheme 4:**
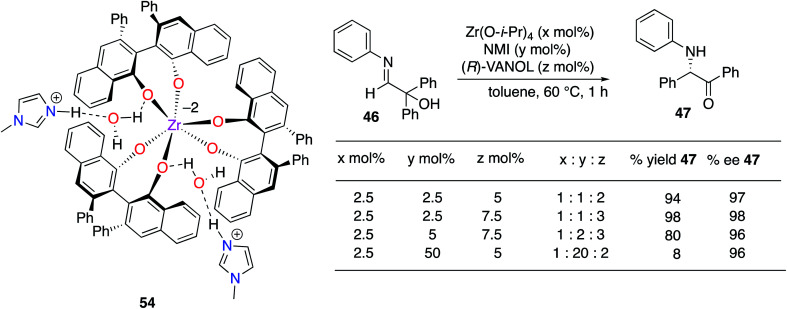
Stucture of the homoleptic VANOL complex of zirconium.

The structure(s) of the zirconium catalysts shown in Scheme 3 are not known with great certainty. We had assumed that the structure of the catalyst for the Mannich reaction in Scheme 3 (ref. 20) had two molecules of ligand per zirconium as this had been reported for the BINOL analog.^22^ However, in studies on the α-imino rearrangements^21^ we were able to grow crystals of the zirconium complex **54** (Scheme 4) which revealed that the zirconium has three VANOL ligands around the zirconium with two protonated *N*-methylimidazoles to balance the charge. A homoleptic zirconium complex with three bis-phenol ligands has not been reported before, but Shibasaki has reported that rare earth catalysts with three BINOL ligands are effective for a number of reactions.^23^ However, later, Schelter and Walsh have shown that Shibasaki's catalysts are in equilibrium with species that only have two BINOL ligands and may be the actual active catalyst species.^24^ Although we have examined the structure of the zirconium catalyst in the present work by NMR the results were not conclusive. Thus, presently it is not known whether the complex **54** is the actual catalyst or if it loses a VANOL ligand in solution to give the active catalyst with only two molecules of VANOL per zirconium. It was found that if the catalyst was prepared from a 1 : 1 : 3 mixture of Zr(O-i-Pr)_4_, *N*-methylimidazole and VANOL the rearrangement of **46** to **47** occurred with essentially the same result as from a 1 : 1 : 2 mixture (Scheme 4). If the catalyst was prepared from a 1 : 2 : 3 mixture the reaction was a little slower and with a 1 : 20 : 2 ratio the yield drops to 8% in the same time (Scheme 4). This may suggest that one of the imidazoles needs to dissociate to initiate the reaction. Additionally, crystals were grown from both the 1 : 1 : 2 and the 1 : 1 : 3 mixtures, their structures were solved, and both were found to be zirconium complex **54**.

Based on the above discussion, the final optimization of the Kabachnik–Field reaction of aryl aldehydes involved examining the effect of the ratio of the components in catalyst formation (Table 2). Unlike the case with α-imino rearrangements (Scheme 4) the use of a large amount of *N*-methylimidazole (10 equiv.) relative to zirconium was not noticeably detrimental to the reaction (entry 1). The yield was slightly higher with three equivalents of VANOL per zirconium than with two but the asymmetric induction was the same (entries 3 *vs.* 5). The yield dropped a bit with a 1 : 2 : 3 ratio of zirconium to NMI to ligand (entry 7 *vs.* entries 3 and 5). The outcomes of the reactions with all variations of the ratio of catalyst components were greatly dependent on the presence of benzoic acid with a decrease in yields of 30 to 69% in the absence of benzoic acid although the asymmetric inductions only decreased by 5 to 10% (entries 1 *vs.* 2, 3 *vs.* 4 and 5 *vs.* 6). The decrease in yields was smaller for a larger ratio of NMI to zirconium (entries 1 *vs.* 2).

**Table 2:** The effect of the catalyst composition and benzoic acid on the Kabachnik–Fields reaction^a^

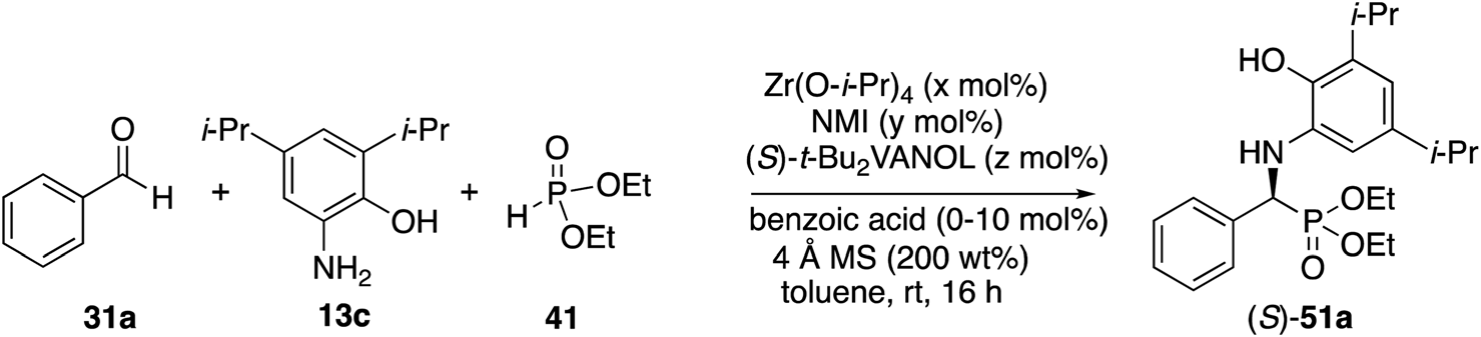							
Entry	*x* mol%	*y* mol%	*z* mol%	*x* : *y* : *z*	Benzoic acid (mol%)	% Yield **51a**	% ee **51a**
1	5	50	10	1 : 10 : 2	10	80	94
2	5	50	10	1 : 10 : 2	0	50	89
3	5	5	10	1 : 1 : 2	10	83	93
4	5	5	10	1 : 1 : 2	0	30	85
5	5	5	15	1 : 1 : 3	10	90	93
6	5	5	15	1 : 1 : 3	0	21	83
7	5	10	15	1 : 2 : 3	10	70	94

a Unless otherwise specified, the reactions were carried out on 0.1 mmol of aldehyde with 1.0 equiv. of aniline and 1.0 equiv. of phosphite and with 200 wt% of 4 Å MS relative to the aniline.

The scope of the Kabachnik–Fields reaction with aromatic aldehydes is summarized in Table 3 and the optimal conditions identified in entry 5 of Table 2 are employed. Both electron rich and electron poor substituted benzaldehydes are tolerated by this catalyst and all but one give α-amino phosphonates **51** with 90 to 98% ee and in 70 to 96% yield. The exception is 4-methylbenzaldehyde **31e**. This aldehyde gives the α-aminophosphonate **51e** in 70% yield and 75% ee under the standard conditions indicated in Table 3. However, if the amount of benzoic acid is lowered from 10 mol% to 5 mol% the % ee increases to 81% with 96% yield. This stands out from most of the other benzaldehydes that were investigated with both 5 and 10 mol% benzoic acid that are indicated in Table 3 where the higher asymmetric induction is observed with 10 mol% benzoic acid. The reaction with pyrrole-2-carboxaldehyde immediately turns very dark and led to the consumption of the starting material with no detectable product formation. However, the reaction with Boc protected pyrrole-2-carboxaldehyde proceeds to give a 92% yield of the α-aminophosphonate **51j** in 77% ee. Pyridine-4-carboxaldehyde **31k** was not a suitable substrate giving the α-aminophosphonate **51k** in 35% yield with 17% ee. The absolute configuration of the α-aminophosphonate **51a** from the (*S*)-catalyst was determined to be (*S*)-**51a** after deprotection to the free amine as shown in Scheme 6. The products from the other aromatic aldehydes were assumed to be homo-chiral.

**Table 3:** Substrate scope for aromatic aldehydes in the Kabachnik–Fields reaction^a^

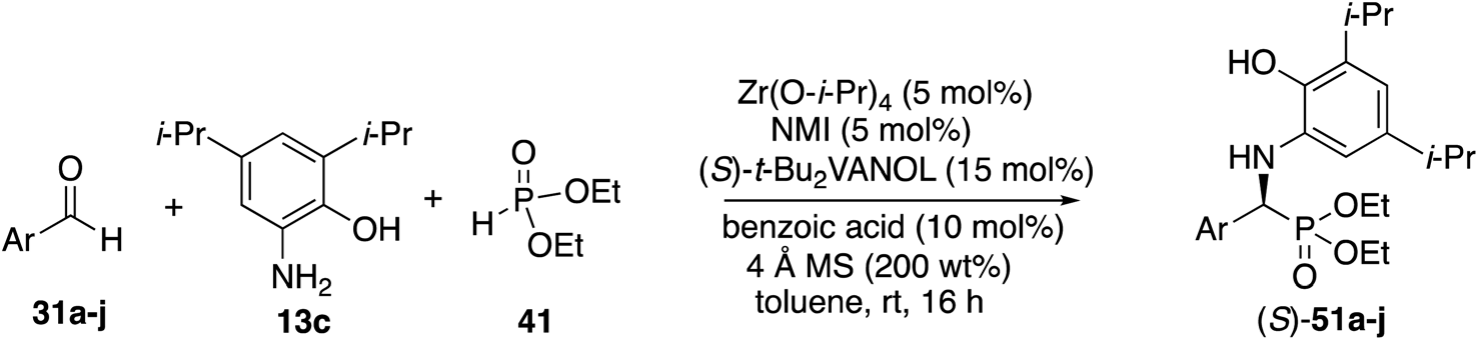
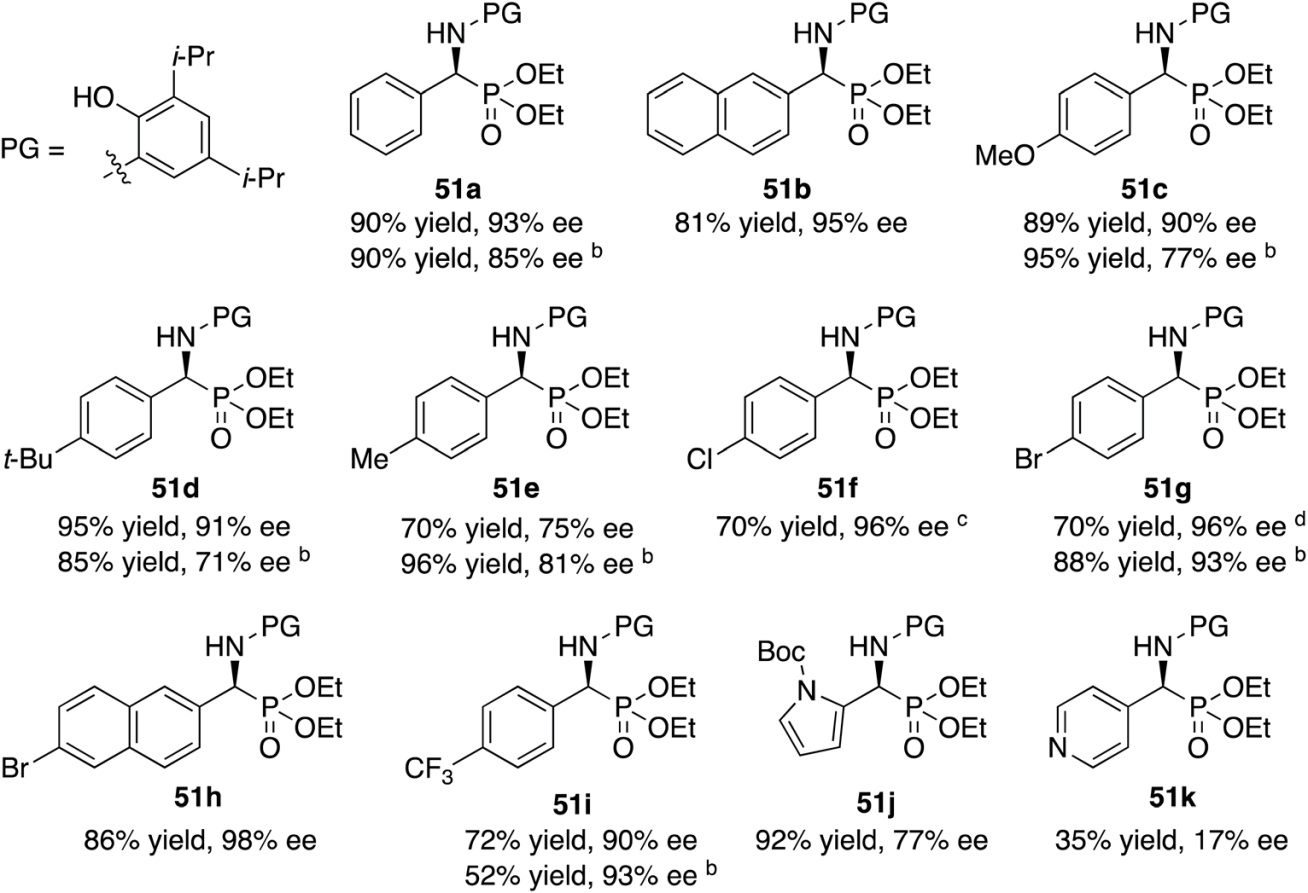

a Zirconium catalysts were prepared by stirring a mixture of 5 mol% Zr(O-i-Pr)_4_(HO-i-Pr), 5 mol% *N*-methylimidazole (NMI) and 15 mol% (*S*)-*t*-Bu_2_VANOL ligand in dry toluene for 30 min at rt under air. Unless otherwise specified, all reactions were carrired out under nitrogen on 0.1 mmol of aldehyde **31** with 1 equiv. of aniline **13c** and 1 equiv. of diethylphosphite **41** in toluene at rt for 16 h in the presence of 4 Å molecular sieves (200 wt% relative to **13c**) and 10 mol% of benzoic acid. All yields are isolated yields. The % ee was determined by HPLC.

b Reaction with 5 mol% benzoic acid.

c With a reaction time of 40 h the yield was 80% with 96% ee.

d With a reaction time of 40 h the yield was 92% with 96% ee.

Next, attention was turned to aliphatic aldehydes. The cyclohexanecarboxaldehyde **55o** served as a prototypical aliphatic aldehyde and the initial results from its reaction with aniline **13c** and phosphite **41** are presented in Table 4. It was quite interesting to observe that there is a vast difference between the reaction carried out with the catalyst prepared from a 1 : 1 : 2 mixture of zirconium/NMI/ligand and that with a 1 : 1 : 3 mixture (Table 4, entry 1 *vs.* 3). The α-aminophosphonate **56** was isolated in 45% yield with 11% ee with the former and 80% yield and 82% ee with the latter. This is in sharp contrast to the observation with benzaldehyde, where no real significant difference was observed between a 1 : 1 : 2 and 1 : 1 : 3 catalyst ratio (Table 2, entries 3 *vs.* entry 5). However, as with benzaldehyde (Table 2), there is a significant difference between the effect of benzoic acid on the reaction of cyclohexanecarboxaldehyde **55o**. With the 1 : 1 : 2 catalyst, the yield dropped from 45% to 9% without benzoic acid although the % ee was slightly enhanced (Table 4, entries 1 and 2). This strong dependence was also observed with the 1 : 1 : 3 catalyst where the yield dropped from 80% to 14% without benzoic acid but in this case the % ee also dropped significantly (entries 3 *vs.* 4). Such a strong dependence on benzoic acid was not seen for a 1 : 10 : 3 catalyst preparation (entries 5 *vs.* 6). The optimal conditions for the reaction of cyclohexanecarboxaldehyde gave phosphonate **56** in 80% yield with 82% ee (Table 4, entry 3). This is to be compared to the same reaction of benzaldehyde under the same conditions which gave phosphonate **51a** in 90% yield and 93% ee (Table 2, entry 5). This is a less than desirable outcome for the reaction of this aliphatic aldehyde, and thus further optimization was needed.

**Table 4:** Optimization of the reaction of cyclohexanecarboxaldehyde **55o** with aniline **13c**^a^

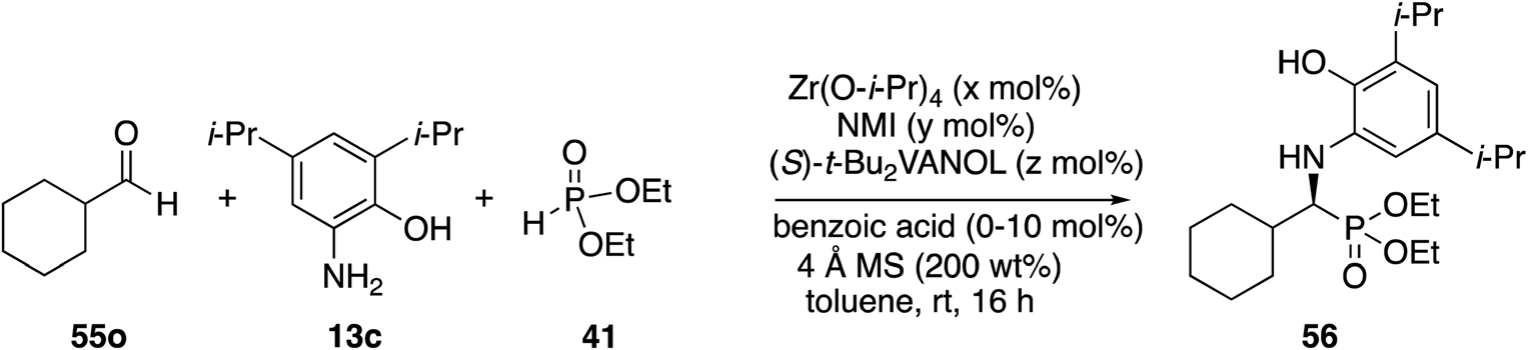							
Entry	*x* mol%	*y* mol%	*z* mol%	*x* : *y* : *z*	mol% benzoic acid	% Yield **56**	% ee **56**
1	5	5	10	1 : 1 : 2	10	45	11
2	5	5	10	1 : 1 : 2	0	9	39
3	5	5	15	1 : 1 : 3	10	80	82
4	5	5	15	1 : 1 : 3	0	14	31
5	5	50	15	1 : 10 : 3	10	78	80
6	5	50	15	1 : 10 : 3	0	82	68
7	5	100	15	1 : 20 : 3	10	60	82

a Unless otherwise specified, the reactions were carried out on 0.1 mmol of aldehyde with 1.0 equiv. of aniline and 1.0 equiv. of phosphite and with 200 wt% of 4 Å MS relative to the aniline.

**Scheme 5:**
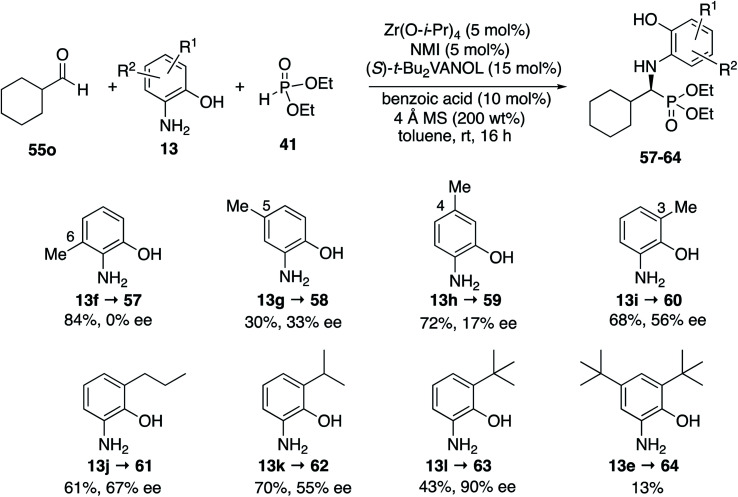
Optimization of aniline 13 for cyclohexanecarboxaldehyde.

It was decided to probe the effect of substituents on all four of the aryl positions of the aniline **13** and determine the consequence of their resulting interactions with the catalyst in a systematic way. All four methyl derivatives **13f** to **13i** were prepared and their reactions with cyclohexanecarboxaldehyde **55o** were examined under the optimal conditions given in Table 4 (entry 3), with the results outlined in Scheme 5. The greatest asymmetric induction (56%) was observed with a methyl group in the 3-position. A further increase to 67% ee was observed when the larger *n*-propyl group was introduced into the 3-position (**13j**) but this was found to decrease to 55% ee when replaced with an iso-propyl (**13k**). Surprisingly, the induction was found to increase again to 90% when the even larger *t*-butyl group was introduced into the 3-position (**13l**), leading to the isolation of the phosphonate **63** in 43% yield. The introduction of *t*-butyl groups into both the 3- and 5-positions leads to a slow reaction and the isolation of phosphonate **64** in only 13% yield. As a result of the screening of the various anilines shown in Scheme 5, the highest asymmetric induction (90% ee) was realized with the 3-*t*-butyl-2-hydroxyaniline **13l** and this was identified as the aniline of choice for screening additional aliphatic aldehydes. The set of anilines shown in Scheme 5 was also employed in the screening of the reaction of benzaldehyde but none gave higher asymmetric inductions than 3,5-diisopropyl-2-hydroxyaniline **13c** used in Table 3 for aromatic aldehydes, although the 3-*t*-butyl-2-hydroxyaniline **13l** gave an identical induction of 93% ee (see the ESI†).

Before committing to a broad investigation of the scope of the reactions of aliphatic aldehydes, we probed the use of 3-*t*-butyl substituted aniline **13l** instead of 3,5-diisopropyl substituted aniline **13c**. The data for the reactions of butanal **55a** are presented in Table 5. The *t*-Bu_2_VANOL ligand **37** was superior to the VANOL ligand **36**, giving the α-aminophosphonate **65** in 81% ee *vs.* 39% ee with aniline **13c** (Table 5, entries 1 *vs.* 2). A slight increase in induction to 85% was observed with the catalyst prepared from a 1 : 1 : 3 mixture of zirconium/NMI/ligand and the yield was increased slightly when the catalyst loading was increased to 10 mol% (entries 3 and 4). Finally, the reaction of butanal was compared under the same conditions with both anilines **13c** and **13l** with the result that the *t*-butyl substituted aniline **13l** gave both higher yield and higher asymmetric induction than aniline **13c** (entries 4 *vs.* 5).

**Table 5:** Evaluation of the reaction of *n*-butanal with anilines **13c** and **13l**^a^

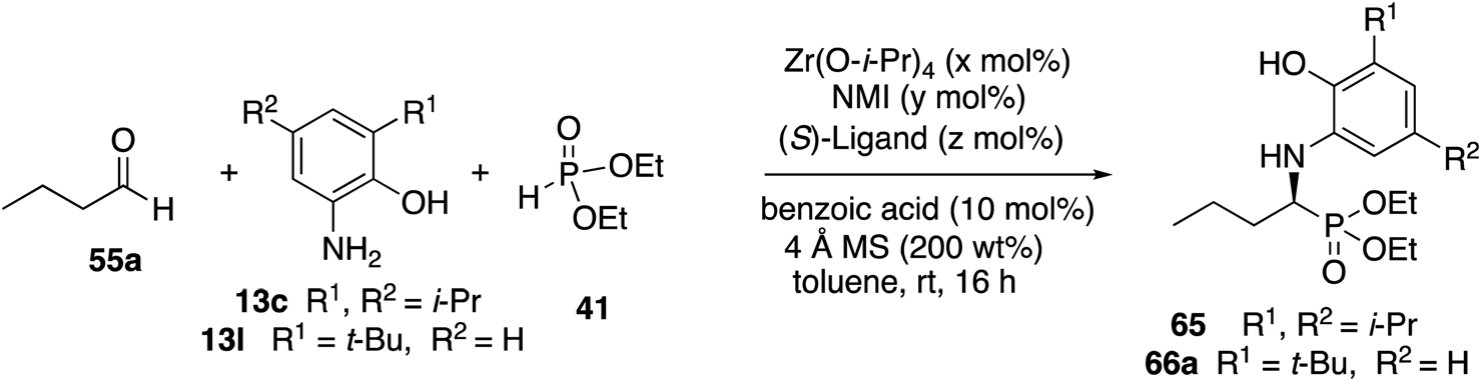								
Entry	Ligand	*x* mol%	*y* mol%	*z* mol%	*x* : *y* : *z*	Aniline	% Yield **65/66a**	% ee **65/66a**
1	VANOL **36**	5	10	15	1 : 2 : 3	**13c**	52	39
2	*t*-Bu_2_VANOL **37**	5	10	15	1 : 2 : 3	**13c**	51	81
3	*t*-Bu_2_VANOL **37**	5	5	15	1 : 1 : 3	**13c**	48	85
4	*t*-Bu_2_VANOL **37**	10	10	30	1 : 1 : 3	**13c**	56	85
5	*t*-Bu_2_VANOL **37**	10	10	30	1 : 1 : 3	**13l**	80	89

a Unless otherwise specified, the reactions were carried out on 0.1 mmol of aldehyde with 1.0 equiv. of aniline and 1.0 equiv. of phosphite and with 200 wt% of 4 Å MS relative to the aniline and 10 mol% benzoic acid.

**Scheme 6:**
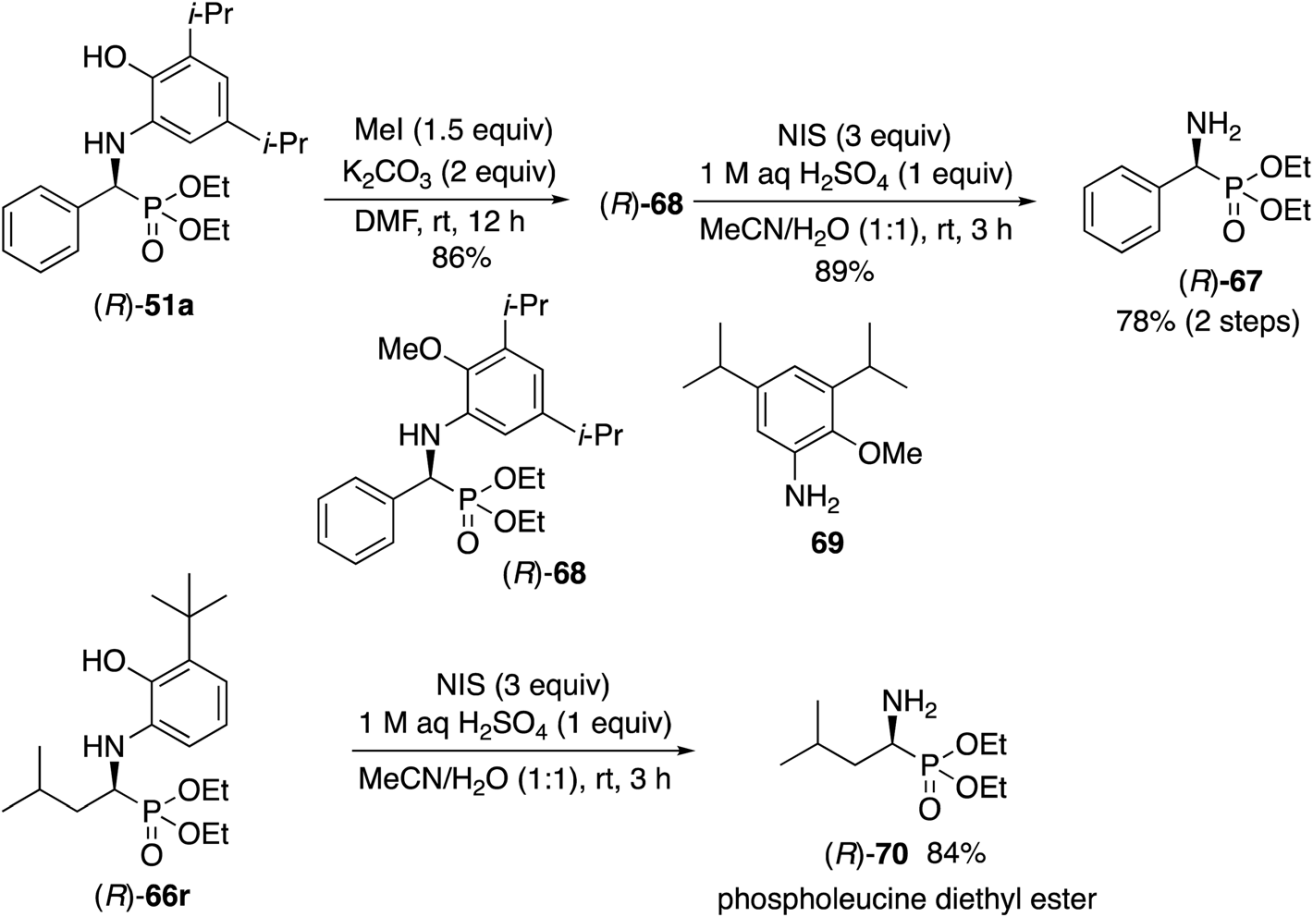
Liberation of α-aminophosphonates.

With the identification of the aniline **13l** as the optimal third component for the Kabachnik–Fields reaction of aliphatic aldehydes, a general study of the scope was undertaken with a set of 18 aliphatic aldehydes (Table 6). The yields for these reactions varied from 20 to 83% and the asymmetric inductions ranged from 71–97%. The asymmetric inductions for the aliphatic aldehydes compared favorably with those for the aromatic aldehydes with the mean asymmetric induction of 90% ee for the aliphatic aldehydes and 91% ee for the aromatic aldehydes shown in Table 3. The best yields were observed in general for α-unbranched aldehydes while α,α-dibranched aldehydes generally gave less product. 2,2-Dimethylpropanal **55q** gave the highest asymmetric induction of 97% but the yield of α-aminophosphonate **66q** was only 28%. Extension of the reaction time from 16 to 48 h did not improve the yield. The reaction is tolerant of a number of functional groups including alkyne (**66e**), a primary bromide (**66f**), silyl ethers (**66g** and **66h**), protected amines (**66i** and **66j**), azides (**66k** and **66l**) and an ester (**66m**). Interestingly, for reasons that we do not understand at this point, the terminal olefin in 5-hexenal **55d** only reacted to give the α-aminophosphonate **66d** in 20% yield. Increasing the reaction time with aldehyde **55d** from 16 to 48 h did not improve the yield of **66d**. This was true of many of the aldehydes in Table 6, however, an increase in the reaction time with the phthalimide protected γ-amino butanal **55j** from 16 to 72 h increased the yield of **66j** from 24% to 53%. The α-branched aldehydes **55n**, **55o** and **55p** gave the corresponding α-aminophosphonates in moderate yields and good to excellent asymmetric inductions. The β-branched aldehyde isovaleraldehyde **55r** gave the phosphonoleucine diethyl ester **66r** in 83% yield and 91% ee. The absolute configuration of the α-aminophosphonate **66r** from the (*S*)-catalyst was determined to be (*S*)-**66r** after deprotection to the free amine as shown in Scheme 6. The products from the other aliphatic aldehydes in Table 6 were assumed to be homo-chiral.

**Table 6:** Substrate scope for aliphatic aldehydes in the Kabachnik–Fields reaction^a^

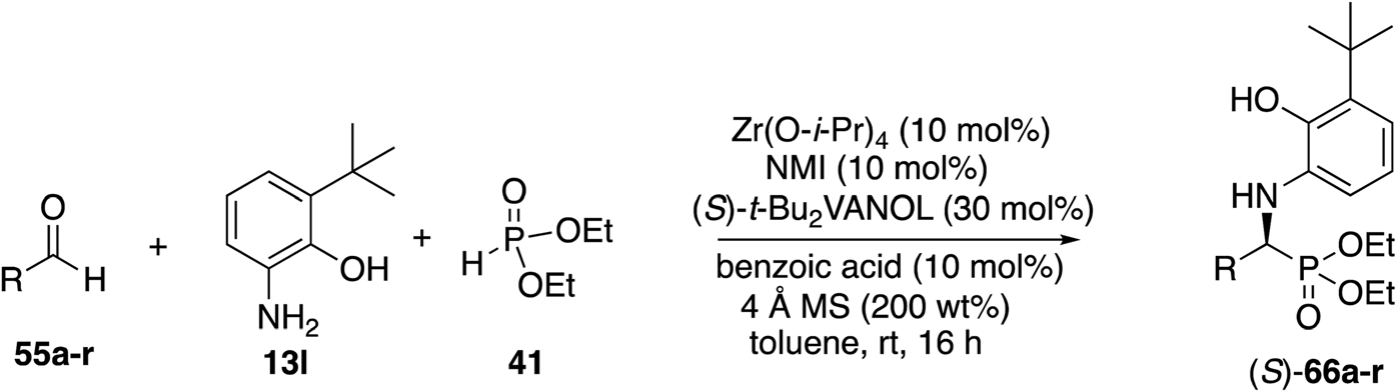
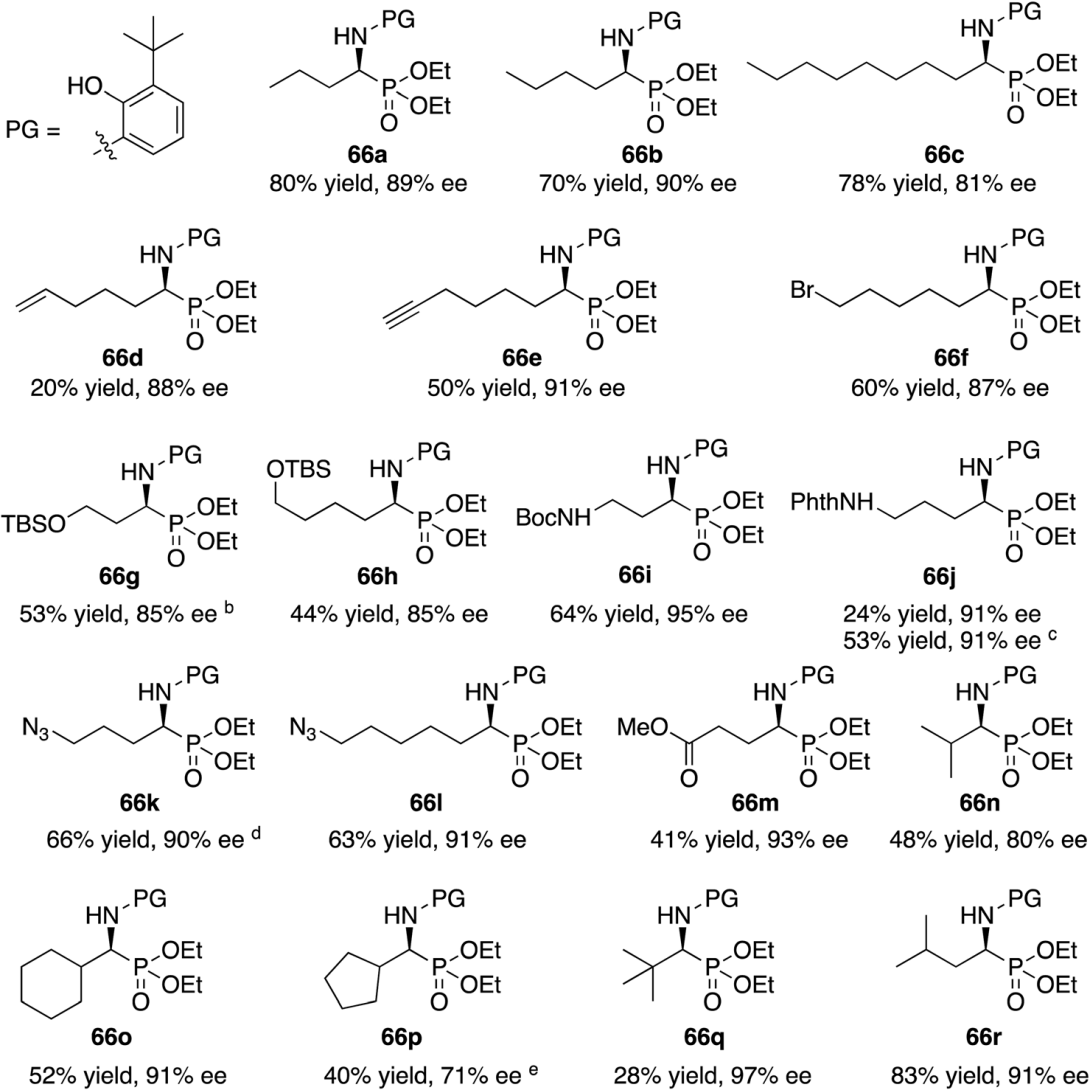

a Zirconium catalysts were prepared by stirring a mixture of 10 mol% Zr(O-i-Pr)_4_(HO-i-Pr), 10 mol% *N*-methylimidazole (NMI) and 30 mol% (*S*)-*t*-Bu_2_VANOL ligand in dry toluene for 30 min at rt under air. Unless otherwise specified, all reactions were carried out under nitrogen on 0.1 mmol of aldehyde **55** with 1 equiv. of aniline **13l** and 1 equiv. of diethylphosphite **41** in toluene at rt for 16 h in the presence of 4 Å molecular sieves (200 wt% relative to **13l**) and 10 mol% of benzoic acid. All yields are isolated yields. The % ee was determined by HPLC.

b Reaction time was 24 h.

c Reaction time was 72 h.

d Reaction time was 48 h.

e This reaction was repeated and both times 71% ee was obtained.

The liberation of the α-aminophosphonates requires a slightly different protocol for the aryl and aliphatic aldehydes. For the phosphonate (*R*)-**51a** from benzaldehyde it is first necessary to methylate the phenol function to give the methyl ether (*R*)-**68**. The free amine can then be obtained by oxidative deprotection of (*R*)-**68** with *N*-iodosuccinimide to give the α-aminophosphonate (*R*)-**67** in 78% yield in two steps. The absolute configuration of **67** was determined by comparison of its optical rotation with that previously reported for this compound^25^ and by the ECCD method^26^ (see the ESI†). Direct oxidation of the unprotected phenol unit in (*R*)-**51a** with *N*-iodosuccinimide gave the desired deprotected product (*R*)-**67** but in only 10% yield along with several other products. Direct oxidation of (*R*)-**51a** with ceric ammonium nitrate gave a benzoxazole (see the ESI†). As a consequence of this failure, the Kabachnick–Fields reaction of benzaldehyde **31a** was performed with the *O*-methylated aniline derivative **69** according to the optimized procedure in Table 3 but only gave a 9% yield of **68** as a racemic compound. Thus, the phenol function in the aniline **13c** must play an important role in the reaction by either H-bonding to the catalyst center or by forming a covalent bond with the zirconium. The deprotection of the α-amino phosphonates derived from 3-*t*-butyl-2-hydroxyaniline **13l** and aliphatic aldehydes is much more straightforward. Treatment of the α-amino phosphonate (*R*)-**66r** directly with three equivalents of *N*-iodosuccinimide and 1 equivalent of sulfuric acid in a mixture of water and acetonitrile gave phospholeucine diethyl ester (*R*)-**70** in 84% yield. The absolute configuration of **70** was determined by comparison of its optical rotation with that previously reported for this compound^27^ and by the ECCD method developed previously^26^ (see the ESI†).

## 3. Conclusion

An effective asymmetric catalyst has been developed for the Kabachnik–Fields three component reaction of aldehydes, amines and phosphites to give α-aminophosphonates. This catalyst was first optimized for aromatic aldehydes and later was extensively re-optimized to find a suitable catalyst for aliphatic aldehydes. The catalyst is generated *in situ* from zirconium tetraisopropoxide, *N*-methylimidazole (NMI) and a vaulted biaryl ligand with the optimal ratio of 1 : 1 : 3. Several different vaulted biaryl ligands were examined including VAPOL, VANOL and several 7,7′-disubstituted VANOL ligands with 7,7′-di-*t*-butylVANOL found to be the most effective. It was found that the yields and to some extent the asymmetric inductions could be increased in the presence of 10 mol% benzoic acid. For aromatic aldehydes the optimal amine was found to be a substituted 2-hydroxyaniline and of the several anilines screened 3,5-diisopropyl-2-hydroxyaniline was identified as superior and the optimal phosphite was diethyl phosphite. To achieve the desired level of asymmetric induction with aliphatic aldehydes an additional set of eight substituted 2-hydroxyanilines was prepared and screened and the most effective was found to be 3-*t*-butyl-2-hydroxyaniline. The asymmetric inductions for aliphatic aldehydes were comparable with those for aromatic aldehydes with a mean induction of 90% ee for the former and 91% ee for the latter. The best method for the liberation of the free amine from the aniline substituted α-aminophosphonates involved oxidation with *N*-iodosuccinimide and in the case of aromatic substrates, this first required the *O*-methylated phosphonate.

## Data availability

The data is in the ESI.†

## Author contributions

YD conceived the project and carried out the large majority of the reactions. WW wrote the manuscript and the other authors were minor contributors in the laboratory. All authors contributed to discussions.

## Conflicts of interest

There are no conflicts to declare.

## Supplementary Material

SC-012-D1SC03222D-s001

SC-012-D1SC03222D-s002
