# Supplementary material for: Zirconium-catalyzed asymmetric Kabachnik–Fields reactions of aromatic and aliphatic aldehydes
Source: Chem Sci. 2021 Aug 3;12(37):12333–45. doi: 10.1039/d1sc03222d (PMC8480333; doi:10.1039/d1sc03222d)
Supplement: SC-012-D1SC03222D-s001 [file SC-012-D1SC03222D-s001.pdf]

Electronic Supplementary Material (ESI) for Chemical Science.
This journal is © The Royal Society of Chemistry 2021

Zirconium-Catalyzed Asymmetric Kabachnik-Fields Reactions of Aromatic and Aliphatic Aldehydes

Yijing Dai, Li Zheng, Debarshi Chakraborty, Babak Borhan and William D. Wulff,*

Department of Chemistry, Michigan State University, East Lansing, MI 48824

wulff@chemistry.msu.edu

Supporting Information

Table of contents

1. General Information S-1
2. Procedures for the preparation of substituted hydroxyanline **13c**, **13d**, **13e**, **13i**, **13j**, **13k**, **13l** S-2
   General procedure A for the nitration of phenols S-3
   General procedure B for hydrazine reduction of nitroarenes S-4
3. Procedure for the preparation of the aromatic aldehyde **51j** S-14
4. Procedures for the preparation of the aliphatic aldehydes **55d-m** S-15
   General procedure C for the Swern oxidation of alcohols S-15
5. The asymmetric Kabachnik-Fields reaction with aromatic aldehydes **51a-51j** S-23
   The effect of molecular sieves on the reaction of benzaldehyde S-23
   The effect of substituted hydroxyl-anilines on the reaction of benzaldehyde S-24
   The effect of phosphite esters on the reaction of benzaldehyde S-25
   General procedure D for assembling the t-Bu_2VANOL-zirconium catalyst S-26
   General procedure E Kabachnik-Fields reaction with aromatic aldehydes S-27

6. The asymmetric Kabachnik-Fields reaction with aliphatic aldehydes **66a-r** S-39

General procedure F for reaction with aliphatic aldehydes S-39

7. Procedure for the deprotection of product **51a** from benzaldehyde S-57

Deprotection of **51a** by ceric ammonium nitrate S-59

8. Procedure for the deprotection of product **66r** from 3-methylbutanal S-60

9. Determination of the absolute configuration of **67** and **70** by ECCD S-61

10. References S-XX

1. General information

Tetrahydrofuran (THF), diethyl ether and toluene were distilled from sodium metal under nitrogen with benzophenone as indicator. Dichloromethane was distilled from calcium hydride under nitrogen. DMF and acetonitrile were distilled from 4Å molecular sieves under nitrogen. Hexane, ethyl acetate, methanol and ethanol were used as purchased in ACS grade. Commercially available aldehydes and benzoic acids were purified by sublimation or distillation before use. Other reagents were used as purchased from Aldrich or Alfa Aesar. All ligands, including VANOL, VAPOL and their derivatives were prepared according to the literature procedures and were determined to be 99% optical purity.[2-7]

Melting points were determined on a Thomas Hoover capillary melting point apparatus and were uncorrected. IR spectra were taken on Thermo Fisher scientific Nicolet iS5 and Jasco FT/IR-6600. ^{1}H NMR and ^{13}C NMR were recorded on a Varian UnityPlus-500 MHz or Varian Inova-600 MHz instrument in $CDCl_3$ unless otherwise noted. $CHCl_3$ was used as the internal standard for both ^{1}H NMR (δ = 7.26) and ^{13}C NMR (δ = 77.0). HRMS was performed in the Department of Biochemistry at Michigan State University. ECCD was performed by professor Babak Borhan's lab. Analytical thin-layer chromatography (TLC) was performed on silica gel plates with F-254 indicator. Visualization was by short wave (254 nm) and long wave (365 nm) ultraviolet light, by staining with phosphomolybdic acid in ethanol or with the aid of Iodine vapor in silica gel. Column chromatography was performed with silica gel 60 (230 - 450 mesh). HPLC analyses were carried out using a Varian Prostar 210 Solvent Delivery Module with a Prostar 330 PDA Detector and a Prostar Workstation. Optical rotations were obtained

on a Perkin-Elmer 341 polarimeter at a wavelength of 589 nm (sodium D line) using a 1.0-decimeter cell with a total volume of 1.00 mL. Specific rotations are reported in degrees per decimeter at 20 °C.

2. Procedures for the preparation of substituted hydroxyanlines 13c, 13d, 13e, 13i, 13j, 13k, 13l.

*Preparation of aniline **13c***

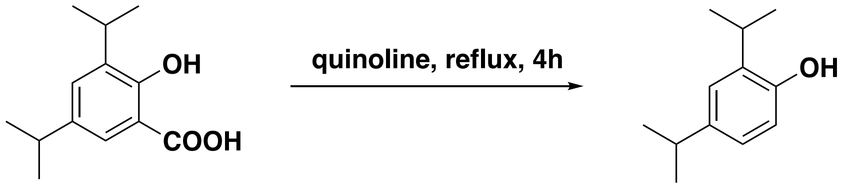

79

2,4-Diisopropylphenol ***80***: To a flame-dried round bottomed flask were added 2-hydroxy-3,5-diisopropylbenzoic acid **79** (5.00 g, 21.9 mmol, 1.00 equiv.) and quinoline (10.0 mL). The reaction was refluxing for 4 hours under nitrogen. After cooling down to the room temperature, the mixture was diluted with ethyl acetate (40.0 mL) and washed by 1 M HCl (2 x 40.0 mL) until pH 1 is reached. The resulting organic layer was separated and then washed with brine (40 mL) and dried over $NaSO_4$. Filtration and removal of the volatiles to gave the crude product as a brown oil. Flash chromatography on silica gel (40 mm x 150 mm, hexane/EtOAc 9:1) provided the purified product **80** as a yellow oil (3.80 g, 21.6 mmol, 98.4% yield). R_f= 0.8 (7:3 hexane/EtOAc). Spectral data for **80**: ^{1}H NMR (500 MHz, Chloroform-*d*) δ 7.30 (d, *J* = 2.3 Hz, 1H), 7.13 (dd, *J* = 8.1, 2.3 Hz, 1H), 6.89 (d, *J* = 8.2 Hz, 1H), 5.96 (s, 1H), 3.55 - 3.43 (hept, *J* = 6.9 Hz, 1H), 3.13 - 3.01 (hept, *J* = 6.9 Hz, 1H), 1.47 (dd, *J* = 14.0, 7.0 Hz, 12H), ^{13}C NMR (126 MHz, Chloroform-*d*) δ 150.75, 141.29, 134.10, 124.55, 124.15, 115.11, 33.59, 27.20, 24.34

(2C), 22.67 (2C). These spectral data match the previously reported data for this compound.[8]

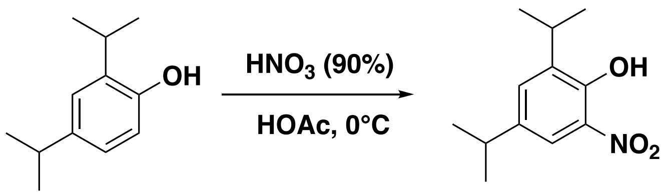

80 **81 (51%)**

*General procedure A for the nitration of phenols: illustrated for the synthesis of 2,4-diisopropyl-6-nitrophenol **81**:*

To a 250 mL Erlenmeyer flask was added a magnetic stir bar, 2,4-di*iso*propylphenol **80** (3.85 g, 24.1 mmol, 1.00 equiv.) and acetic acid (27 mL). The flask was cooled in ice bath for 5 min. To another 50 mL Erlenmeyer flask was added acetic acid (9.00 mL) and HNO_3 (1.70 mL, c = 90%) and then the solution was cooled in ice bath for 5 min. The HNO_3 solution was transferred to the 2,4-di*iso*propylphenol **80** solution dropwise with a pipette. After stirring in an ice bath for 2 min, H_2O (200 mL) was added in one portion to quench the reaction. The aqueous solution was divided into two portions, and both portions were extracted with DCM (20.0 mL x 4). The combined organic layer was successively washed with H_2O (20.0 mL) and brine (20.0 mL) and dried over $NaSO_4$. The solution was filtered and concentrated under rotary evaporation. The crude product was purified by silica gel column chromatography (40 mm x 100 mm, hexane/EtOAc 95:5). The reaction afforded the product **81** (2.47 g, 11.1 mmol, 52% yield) as a yellow oil. R_f= 0.9 (7:3 hexane/EtOAc). Spectral data for **81**: ^{1}H NMR (500 MHz, Chloroform-*d*) δ 10.96 (s, 1H), 7.80 (d, *J* = 2.3 Hz, 1H), 7.38 (d, *J* = 2.3 Hz, 1H), 3.42 (m, *J* = 7.0 Hz, 1H), 2.90 (hept, *J* = 6.9 Hz, 1H), 1.26 (dd, *J* = 9.1, 6.9 Hz, 12H). ^{13}C

NMR (126 MHz, Chloroform-*d*) δ 151.38, 140.24, 139.21, 133.25, 133.19, 119.10, 33.38, 27.09, 23.79 (2C), 22.31 (2C). These spectral data match the previously reported data for this compound.[9]

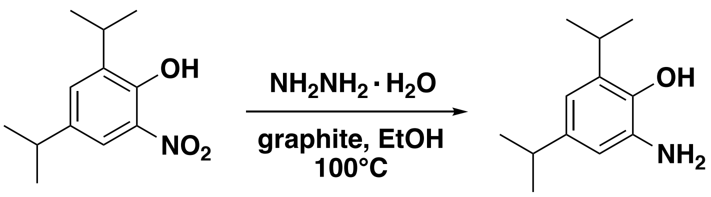

81

13c (60%)

*General procedure B for hydrazine reduction of nitroarenes: Illustrated for the synthesis of 2-hydroxy-3.5-diisopropylaniline **13c**.*

To a 100 mL round bottomed flask was added 2,4-diisopropyl-6-nitrophenol **81** (2.47 g, 11.1 mmol, 1.00 equiv.), graphite (1.58 g), hydrazine monohydrate (1.35 mL, 27.7 mmol, 2.5 equiv.) and ethanol (31.7 mL). The flask was equipped with a condenser and the top was sealed by a septum with a needle attached to it. The flask was heated in a 100 °C for 3.5 h (another 5.00 equiv. of hydrazine monohydrate was added in two equal portions every other hour). The solution was allowed to cool to room temperature, and filtered through a Celite pad, the filter cake was washed with dichloromethane (50.0 mL). The filtrate was concentrated with a rotary evaporation and flash chromatography (40 mm x 160 mm, Hexane/EtOAc 8:2) afford the crude product as a brown solid. Crystallization with Hexane/EtOAc (9:1) gave the pure product **13c** as a white solid (1.30 g, 6.64 mmol, 60% yield, mp: 106.8-108.2 °C). R_f= 0.4 (7:3 hexane/EtOAc). Spectral data for **13c**: ^{1}H NMR (500 MHz, Chloroform-*d*) δ 6.50 (s, 1H), 6.46 (s, 1H), 3.67-3.64 (br, NH_2), 3.21 (hept, *J* = 6.9 Hz, 1H), 2.73 (hept, *J* = 6.9 Hz, 1H), 1.18 (dd, *J* = 10.5, 6.8 Hz, 12H). ^{13}C NMR (126 MHz, Chloroform-*d*) δ 141.68, 139.62, 136.58,

135.83, 114.53, 112.10, 33.71, 27.20, 24.26 (2C), 22.94 (2C). IR (thin film) 3390s, 3317s, 2956vs, 1740vs, 1592vs, 1486s,1436s, 1215vs, 859vs cm^{-1}. HRMS (ESI-TOF) *m/z* found 194.1554 ($[M+H]^+$); calcd. 194.1545 for $C_{12}H_{20}NO$.

Preparation of aniline ***13d***

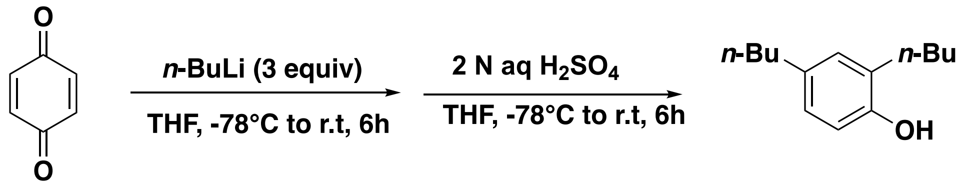

82 **83 (35%)**

2,4-Di-*n*-*b*utylphenol ***83***: To a solution of *p*-benzoquinone **82** (1.00 g, 9.25 mmol, 1.00 equiv.) in THF (25.0 ml) was added *n*-butyllithium (11.1 mL, 2.5 M in hexane, 3.00 equiv) at -78°C. The resulting solution was stirred for 6 hours while allowing the temperature to rise to 20°C. The resulting mixture was then hydrolyzed with water (20 mL), acidified with 2 N sulfuric acid, and extracted with ether (3 x 20.0 mL). The organic layer was dried over $NaSO_4$ and concentrated to give the crude product **83** (0.67 g, 3.23 mmol, 35% yield) as a yellow oil, which was purified by flash chromatography (silica gel, 40 mm x 160 mm, hexane/EtOAc 9:1). R_f= 0.8 (7:3 hexane/EtOAc). Spectral data for **83**: ^{1}H NMR (500 MHz, Chloroform-*d*) δ 7.07 (d, *J* = 2.3 Hz, 1H), 6.99 (dd, *J* = 8.1, 2.3 Hz, 1H), 6.79 (d, *J* = 8.0 Hz, 1H), 5.55 (s, 1H, OH), 2.76 - 2.69 (m, 2H), 2.69 - 2.62 (m, 2H), 1.78 - 1.65 (m, 4H), 1.56 - 1.35 (m, 5H), 1.07 (t, *J* = 7.3 Hz, 6H). ^{13}C NMR (126 MHz, Chloroform-d) δ 151.42, 135.12, 130.25, 128.68, 126.71, 115.24, 35.00, 34.15, 32.25, 22.80, 22.50, 14.10 (2C). These spectral data match those previously reported for this compound.[10]

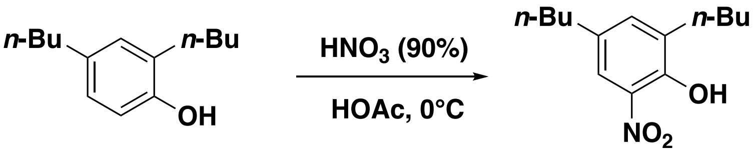

83 **84 (100%)**

2,4-Di-*n*-Butyl-6-nitrophenol ***84***: According to the *general procedure A*, 2,4-di*n*Butylphenol **83** (0.67 g, 3.26 mmol), nitric acid (0.25 mL) and acetic acid (4.05 mL + 1.35 mL) were reacted. After purification by flash chromatography (silica gel, 20 mm x 160 mm, hexane/EtOAc 95:5), 3,5-di*n*butyl-6-nitrophenol **84** (0.93 g, 3.26 mmol, 100% yield) was obtained as an orange oil. R_f= 0.9 (7:3 hexane/EtOAc). Spectral data for **84**: ^{1}H NMR (500 MHz, Chloroform-*d*) δ 10.80 (d, 1H, OH), 7.72 (d, *J* = 2.3 Hz, 1H), 7.26 (d, *J* = 2.2 Hz, 1H), 2.72 - 2.65 (m, 2H), 2.58 - 2.51 (m, 2H), 1.63 - 1.52 (m, 4H), 1.43 - 1.29 (m, 4H), 0.93 (t, *J* = 7.4 Hz, 6H). ^{13}C NMR (126 MHz, Chloroform-*d*) δ 151.69, 138.10, 134.04, 133.54, 133.13, 121.22, 34.45, 33.30, 31.52, 29.52, 22.52, 22.18, 13.86, 13.81.

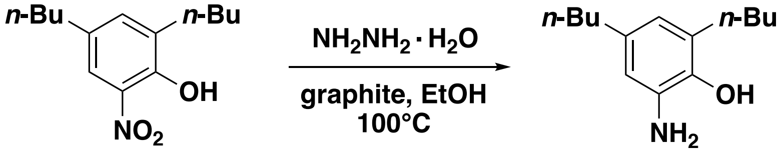

84 **13d (49%)**

2-Hydroxy-3,5-di-*n*-butylaniline ***13d***: According to *general procedure B,* 2,4-di*n*butyl-6-nitrophenol **84** (0.82 g, 3.26 mmol, 1.00 equiv.), hydrazine (0.40 mL x 3, 2.5 equiv.) and graphite (0.47 g) were reacted in ethanol (9.32 mL). After purification by flash chromatography (silica gel, 20 mm x 160 mm, hexane/EtOAc 8:2) and crystallization with hexane/EtOAc (9:1), the reaction afforded the product **13d** (0.35 g, 1.59 mmol, 49% yield) as a yellow oil. R_f= 0.4 (7:3 hexane/EtOAc). Spectral data for **13d**: ^{1}H NMR (500 MHz, Chloroform-*d*) δ 6.50 (d, *J* = 2.1 Hz, 1H), 6.44 (d, *J* = 2.1 Hz, 1H),

3.52 (s, 2H, NH_2), 2.58 - 2.51 (m, 2H), 2.49 - 2.42 (m, 2H), 1.64 - 1.49 (m, 4H), 1.46 - 1.32 (m, 4H), 0.94 (t, *J* = 7.3 Hz, 6H). ^{13}C NMR (126 MHz, Chloroform-*d*) δ 140.64, 135.40, 128.14, 120.82, 115.59, 35.08, 33.94, 32.08, 29.84, 22.69, 22.41, 14.00 (2C) (one sp^2 carbon is not located). IR (thin film) 3395s, 3313s, 2925vs, 1602vs, 1486vs, 1203vs, 848, 724s cm^{-1}. HRMS (ESI-TOF) *m/z* found 222.1866 ($[M+H]^+$); calcd. 222.1858 for $C_{14}H_{24}NO$.

*Preparation of aniline **13e***

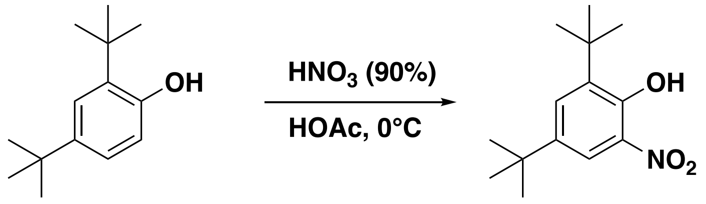

85 **86 (56%)**

2,4-di*tert*-Butyl-6-nitrophenol ***86***: According to *general procedure A*, 2,4-di-*t*-butylphenol **85** (4.97 g, 24.1 mmol, 1.00 equiv.), nitric acid (1.90 mL) and acetic acid (30.0 mL + 10.0 mL) were reacted. After purification by flash chromatography (silica gel, 40 mm x 160 mm, hexane/EtOAc 95:5), 2,4-di-*t*-butyl-6-nitrophenol **86** (3.38 g, 13.5 mmol, 56% yield) was isolated as a yellow oil. R_f= 0.9 (7:3 hexane/EtOAc). Spectral data for **86**: ^1H NMR (500 MHz, Chloroform-*d*) δ 11.47 (d, *J* = 0.6 Hz, 1H, OH), 7.97 (d, *J* = 2.5 Hz, 1H), 7.66 (d, *J* = 2.6 Hz, 1H), 1.46 (s, 9H), 1.33 (s, 9H). ^{13}C NMR (126 MHz, Chloroform-*d*) δ 153.01, 141.93, 139.82, 133.62, 132.59, 118.84, 35.72, 34.51, 31.10 (3C), 29.36 (3C).

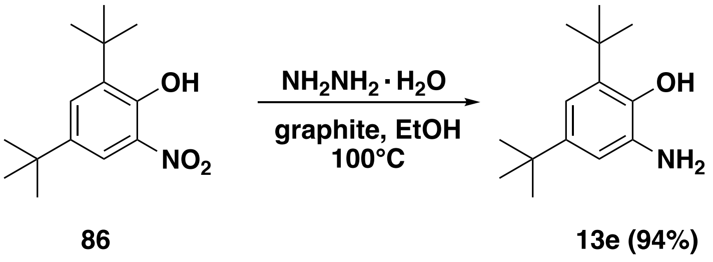

2-Hydroxy-3,5-di*t*butylaniline ***13e***: According to *general procedure B*, 2,4-di-*t*-butyl-6-nitrophenol **86** (3.38 g, 13.5 mmol, 1.00 equiv.), hydrazine (1.64 mL x 3, 2.5 equiv.) and graphite (1.92 g) were added to ethanol (38.5 mL). After purification by flash chromatography (silica gel, 40 mm x 160 mm, hexane/EtOAc 8:2) and crystallization with hexane/EtOAc (9:1), the reaction afforded the product **13e** as a light purple solid (1.80 g, 8.14 mmol, 61% yield, mp: 163-168 °C). R_f= 0.4 (7:3 hexane/EtOAc). Spectral data for **13e**: ^{1}H NMR (500 MHz, Chloroform-*d*) δ 6.91 (d, *J* = 2.3 Hz, 1H), 6.81 (d, *J* = 2.3 Hz, 1H), 3.22 (br, 2H, NH2), 1.42 (s, 9H), 1.28 (s, 9H). ^{13}C NMR (126 MHz, Chloroform-*d*) δ 143.91, 142.48, 135.80, 132.62, 116.88, 115.59, 34.69, 34.26, 31.61(6C), 29.84 (6C). IR (thin film) 3365s, 3289s, 2943vs, 2365s, 1592s, 1486vs, 1418vs, 1331vs, 1237s, 946vs, 741vs cm^{-1}. HRMS (ESI-TOF) *m/z* found 222.1860 ([M+H]$^+$); calcd. 222.1858 for $C_{14}H_{24}NO$. The spectral data match those previously reported for this compound.[13]

0

*Preparation of aniline **13i***

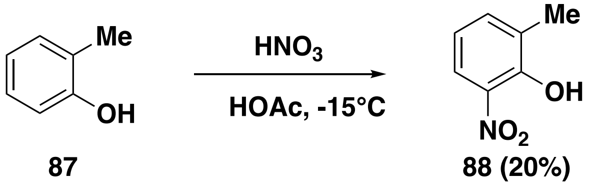

2-Methyl-6-nitrophenol **88**: According to *general procedure A*, 2-methylphenol **87** (1.00 g, 9.25 mmol), nitric acid (1.37 mL) and acetic acid (2.75 mL + 1.53 mL) were reacted together and stirred at -15°C. After purification by flash chromatography (silica gel, 20 mm x 160 mm, hexane/EtOAc 95:5), the reaction afforded 2-methyl-6-nitrophenol **88** (0.28 g, 1.85 mmol, 20% yield) as a yellow oil. R_f= 0.9 (7:3 hexane/EtOAc). Spectral data for **88**: ^{1}H NMR (500 MHz, Chloroform-*d*) δ 10.92 (d, *J* = 0.6 Hz, 1H, OH), 7.96 (ddd, *J* = 8.6, 1.7, 0.7 Hz, 1H), 7.45 (ddq, *J* = 7.4, 1.7, 0.8 Hz, 1H), 6.88 (dd, *J* = 8.6, 7.3 Hz, 1H), 2.34 (d, *J* = 0.7 Hz, 3H).

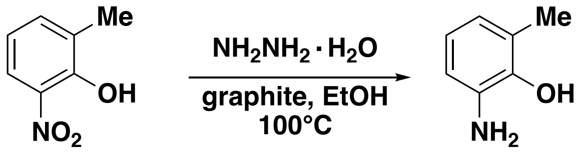

88

13i (70%)

2-Hydroxy-3-methylaniline **13i**: According to *general procedure B*, 2-methyl-6-nitrophenol **88** (0.22 g, 1.43 mmol, 1.00 equiv.), hydrazine (0.17 mL x 3, 2.5 equiv.) and graphite (0.21 g) were added to ethanol (4.10 mL). After purification by flash chromatography (silica gel, 20 mm x 160 mm, hexane/EtOAc 8:2) and crystallization with hexane/EtOAc (9:1), the reaction afforded the product **13i** as a yellow solid (0.12 g, 1.0 mmol, 70% yield, mp: 82.8-85.6 °C). R_f= 0.4 (7:3 hexane/EtOAc). Spectral data for **13i**: ^{1}H NMR (500 MHz, Chloroform-*d*) δ 6.89 - 6.42 (m, 3H), 3.56 (br, 2H, NH_2), 2.24 (s, 3H). ^{13}C NMR (126 MHz, Chloroform-*d*) δ 143.71, 132.64, 122.84, 118.72, 112.84, 17.28 (one sp^2 carbon is not located). IR (thin film) 3396s, 3314s, 2924s, 1582vs, 1480vs, 1330s, 1205vs, 804vs, 724vs cm^{-1}. HRMS (ESI-TOF) *m/z* found 124.0773 ([M+H]$^+$); calcd. 124.0762 for $C_7H_{10}NO$.

*Preparation of aniline **13j***

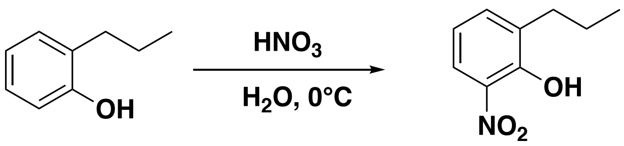

89 90 (17%)

2-*n*-Propyl-6-nitrophenol **90**: To an oven-dried 25 mL round bottomed flask were added 2-*n*-propyl-phenol **89** (1.00 g, 7.30 mmol), nitric acid (1.10 mL) and water (5.00 mL) at 0°C under nitrogen. Then the mixture was allowed to stir at room temperature for 2 hours. Diethyl ether (5.00 mL x 3) was added to extract the aqueous layers. The combined organic layers were dried over $NaSO_4$ and concentrated under rotary evaporation. After purification of the crude product by flash chromatography (silica gel, 20 mm x 160 mm, hexane/EtOAc 95:5), the reaction afforded 2-*n*-propyl-6-nitrophenol **90** (0.22 g, 1.24 mmol, 17% yield) as a yellow oil. R_f= 0.9 (7:3 hexane/EtOAc). Spectral data for **90**:^{1}H NMR (500 MHz, Chloroform-*d*) δ 10.92 (s, 1H, OH), 7.93 (d, *J* = 8.6 Hz, 1H), 7.42 (d, *J* = 7.3 Hz, 1H), 6.88 (t, *J* = 7.9 Hz, 1H), 2.68 (t, *J* = 7.7 Hz, 2H), 1.76 - 1.57 (m, 3H), 0.97 (t, *J* = 7.4 Hz, 3H). The spectral data match those previously reported for this compound.[12]

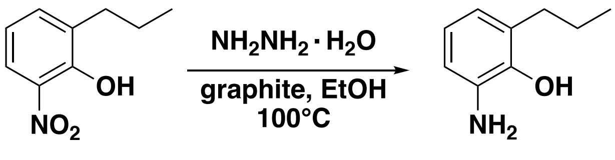

90 13j (70%)

2-Hydroxy-3-*n*propylaniline **13j**: According to *general procedure B*, 2-*n*-propyl-6-nitrophenol **90** (0.23 g, 1.24 mmol, 1.00 equiv.), hydrazine (0.15 mL x 3, 2.5 equiv.) and

graphite (0.18 g) were added to ethanol (3.60 mL). After purification by flash chromatography (silica gel, 20 mm x 160 mm, hexane/EtOAc 8:2) and crystallization with pure hexane, the reaction afforded the product **13j** as a light yellow solid (0.13 g, 0.87 mmol, 70% yield, mp: 55.2-54.3 °C). R_f= 0.4 (7:3 hexane/EtOAc). Spectral data for **13j**: ^{1}H NMR (500 MHz, Chloroform-*d*) δ 6.76 - 6.59 (m, 3H), 2.55 (t, *J* = 7.7 Hz, 2H), 1.64 (h, *J* = 7.5 Hz, 2H), 0.98 (t, *J* = 7.3 Hz, 3H). ^{13}C NMR (126 MHz, Chloroform-*d*) δ 143.04, 133.88, 128.08, 121.16, 120.68, 115.83, 32.01, 22.93, 14.06. IR (thin film) 3392s, 3299s, 2928vs, 1477vs, 1328s, 1193vs, 806vs, 726vs cm^1. HRMS (ESI-TOF) *m/z* found 152.1077 ($[M+H]^+$); calcd. 152.1075 for $C_9H_{14}NO$.

Preparation of aniline 13j

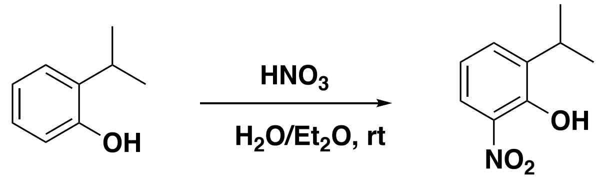

91 92 (25%)

2-*i*-Propyl-6-nitrophenol **92**: To an oven-dried 25 mL round bottomed flask were added 2-*i*-propyl-phenol **91** (2.00 g, 14.6 mmol), nitric acid (2.20 mL), water (20.0 mL) and diethyl ether (20 mL) at room temperature under nitrogen. Then the mixture was stirring for 2 days and the aqueous layer was extracted with diethyl ether (20.0 mL x 3). The combined organic layer was dried over $NaSO_4$ and concentrated under rotary evaporation. After purification of the crude product by flash chromatography (silica gel, 20 mm x 160 mm, hexane/EtOAc 95:5), the reaction afforded 2-*i*-propyl-6-nitrophenol **92** (0.66 g, 3.65 mmol, 25% yield) as a yellow oil. R_f= 0.9 (7:3 hexane/EtOAc. Spectral data for **92**: ^{1}H NMR (500 MHz, Chloroform-*d*) δ 11.07 (d, *J* = 0.6 Hz, 1H, OH), 7.96 (dd,

J = 8.5, 1.6 Hz, 1H), 7.52 (dd, J = 7.5, 1.7 Hz, 1H), 6.97 - 6.91 (m, 1H), 3.50 - 3.39 (m, 1H), 1.27 (d, J = 6.9 Hz, 6H). The spectral data match those previously reported for this compound.[13]

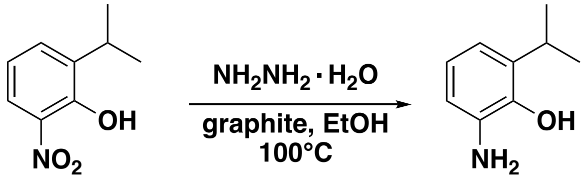

92 **13k (75%)**

2-Hydroxy-3-*iso*propylaniline **13k**: According to *general procedure B*, 2-*i*-propyl-6-nitrophenol **92** (0.65 g, 3.58 mmol, 1.00 equiv.), hydrazine (0.43 mL x 3, 2.5 equiv.) and graphite (0.51 g) were added to ethanol (10.3 mL). After purification by flash chromatography (silica gel, 20 mm x 160 mm, hexane/EtOAc 8:2) and crystallization with hexane/EtOAc (9:1), the reaction afforded the product **13k** as a orange solid (0.41 g, 2.69 mmol, 75% yield, mp: 75.6-77.3 °C). R_f= 0.4 (7:3 hexane/EtOAc). Spectral data for **13k**: ^{1}H NMR (500 MHz, Chloroform-*d*) δ 6.82 - 6.62 (m, 3H), 3.12 (hept, J = 6.9 Hz, 1H), 1.26 (d, J = 6.9 Hz, 6H). ^{13}C NMR (126 MHz, Chloroform-*d*) δ 142.85, 134.26, 133.60, 120.84, 117.64, 116.09, 27.23, 22.67. IR (thin film) 3385s, 3286s, 2961s, 1583s, 1475vs, 1194vs, 897s, 841s, 732vs cm^{-1}. HRMS (ESI-TOF) *m/z* found 152.1078 ([M+H]$^+$); calcd. 152.1075 for $C_9H_{14}NO$.

Preparation of aniline 13j

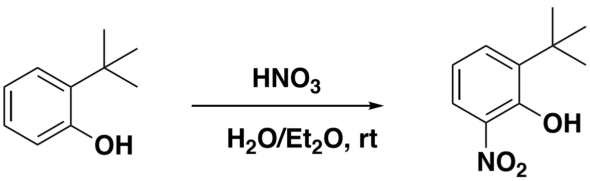

93 **94 (55%)**

2-*tert-Butyl*-6-nitrophenol ***94***: To an oven-dried 25 mL round bottomed flask were added 2-*tert-butyl*-phenol **93** (4.40 g, 29.2 mmol), nitric acid (4.4 0mL), water (40.0 mL) and diethyl ether (40.0 mL) at room temperature under nitrogen. Then the mixture was stirred for 2 days and the aqueous layer was extracted with diethyl ether (40.0 mL x 3). The combined organic layer was dried over $NaSO_4$ and concentrated under rotary evaporation. After purification of the crude product by flash chromatography (silica gel, 20 mm x 160 mm, hexane/EtOAc 95:5), the reaction afforded 2-*tert*-butyl-6-nitrophenol **94** (3.13 g, 16.6 mmol, 55% yield) as a yellow oil. R_f= 0.9 (7:3 hexane/EtOAc). Spectral data for **94**: ^{1}H NMR (500 MHz, Chloroform-*d*) δ 11.56 (d, *J* = 0.6 Hz, 1H, OH), 8.01 (dd, *J* = 8.5, 1.7 Hz, 1H), 7.59 (dd, *J* = 7.7, 1.7 Hz, 1H), 6.90 (dd, *J* = 8.5, 7.6 Hz, 1H), 1.45 (s, 9H). ^{13}C NMR (126 MHz, Chloroform-*d*) δ 155.00, 140.60, 134.63, 123.05, 119.09, 35.55, 29.28 (one sp^2 carbon is not located). These spectral data match those previously reported for this compound.[14]

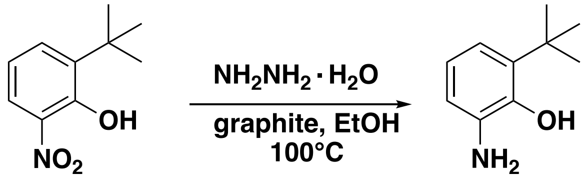

94 **13l (70%)**

2-Hydroxy-*tert-butyl*aniline ***13l***: According to *general procedure*, 2-*tert*-butyl-6-nitrophenol **94** (0.80 g, 4.1 mmol, 1.00 equiv.), hydrazine (0.49 mL x 3, 2.5 equiv.) and graphite (0.58 g) were added to ethanol (11.7 mL). After purification by flash chromatography (silica gel, 40 mm x 160 mm, hexane/EtOAc 8:2) and crystallization with hexane/EtOAc (9:1), the reaction afforded the product **13l** as a pink solid (0.47 g, 2.87 mmol, 70% yield, mp: 97.8-99.0 °C). R_f= 0.4 (7:3 hexane/EtOAc). Spectral data for **13l**: ^{1}H NMR (500 MHz, Chloroform-*d*) δ 6.91 (dd, *J* = 7.8, 1.7 Hz, 1H),

6.85 - 6.70 (m, 2H), 4.33 (br, NH_2, OH), 1.42 (s, 9H). ^{13}C NMR (126 MHz, Chloroform-*d*) δ 146.56, 136.91, 132.97, 120.33, 119.99, 118.69, 34.48, 29.74. IR (thin film) 3382s, 3308s, 2960vs, 1740s, 1595vs, 1448vs, 1273vs, 1198s, 788vs, 734vs cm^{-1}. HRMS (ESI-TOF) *m/z* found 166.1241 ($[M+H]^+$); calcd. 166.1232 for $C_{10}H_{16}NO$.

5. Procedure for preparation of aromatic aldehyde 51j

*Preparation of aldehyde **31j***

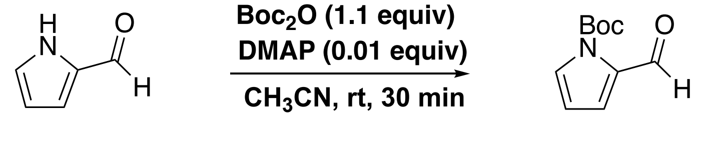

95 **31j (100%)**

t-Butyl 2-formyl-1*H*-pyrrole-1-carboxylate **31j**: To an oven-dried 50 mL round bottomed flask were added 1*H*-pyrrole-2-carbaldehyde **95** (0.50 g, 5.30 mmol, 1.00 equiv), Boc anhydride (1.30 g, 5.83 mmol, 1.10 equiv), 4-dimethylaminopyridine (DMAP, 6.40 mg, 0.01 equiv) and acetonitrile (10.0 mL) under nitrogen. After stirring for 30 minutes, the reaction was concentrated under rotary evaporation. The resulting crude product was purified with flash chromatography (silica gel, 20 mm x 160 mm, hexane/EtOAc 9:1) and afforded the product **31j** (1.03 g, 5.30 mmol, 100% yield) as a colorless oil. R_f= 0.8 (7:3 hexane/EtOAc). Spectral data for **31j**: ^1H NMR (500 MHz, Chloroform-*d*) δ 10.31 (d, *J* = 0.7 Hz, 1H), 7.42 (dd, *J* = 3.1, 1.7 Hz, 1H), 7.16 (dd, *J* = 3.8, 1.8 Hz, 1H), 6.26 (ddd, *J* = 3.8, 3.1, 0.7 Hz, 1H), 1.62 (s, 9H). ^{13}C NMR (126 MHz, Chloroform-*d*) δ 182.39, 148.38, 134.74, 127.36, 121.23, 111.72, 85.81, 27.96. IR (thin film) 1748 s, 1667 m, 1390 s, 427 vs cm^{-1}. These spectral data match those previously reported for this compound.[15]

4. Procedures for the preparation of the aliphatic aldehydes 55d-m

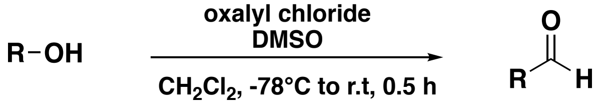

General procedure C for the Swern oxidation of alcohols: To a solution of oxalyl chloride in dichloromethane at -78 °C under nitrogen was added dimethyl sulfoxide. After 15 min, a solution of the corresponding alcohol in dichloromethane was added. The mixture was stirred for 30 min before triethylamine was added. The resulting mixture was then warmed up to room temperature and diluted with dichloromethane. The reaction was quenched by sat $NaHCO_3$. The organic phase was separated, dried with $NaSO_4$ and concentrated. Purification of the crude aldehyde by silica gel chromatography (hexane/EtOAc as eluent) afforded the resulting aldehyde.

*Preparation of aldehyde **55d***

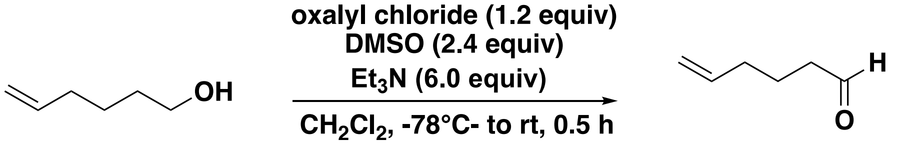

96 **55d (60%)**

Hex-5-enal **66d**: Following *general procedure C*, the reaction of hex-5-en-1-ol **96** (1.2 mL g, 10.0 mmol, 1.00 equiv), oxalyl chloride (1.0 mL, 12.0 mmol, 1.2 equiv), DMSO (1.68 mL, 24.0 mmol, 2.40 equiv) and triethylamine (8.30 mL, 60.0 mmol, 6.00 equiv) in dichloromethane (15.0 mL x 2) afforded product **55d** (0.69 g, 7.00 mmol, 70%) as a colorless oil. R_f= 0.7 (7:3 hexane/EtOAc). Spectral data for **55d**: ^{1}H NMR (500 MHz, Chloroform-*d*) δ 9.78 (d, *J* = 1.8 Hz, 1H), 5.90 - 5.64 (m, 1H), 5.08 - 4.93 (m, 2H), 2.45

(td, J = 7.3, 1.7 Hz, 2H), 2.12 - 2.06 (m, 2H), 1.74 (p, J = 7.4 Hz, 2H). The spectral data match those previously reported for this compound.[16]

*Preparation of aldehyde **55e***

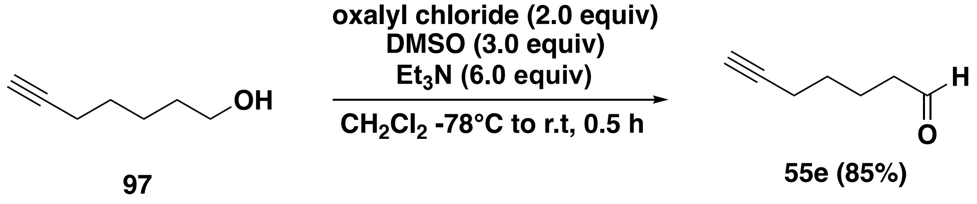

Hept-6-ynal **55e**: Following *general procedure C*, the reaction of hept-6-yn-1-ol **97** (1.00 g, 8.90 mmol, 1.00 equiv), oxalyl chloride (1.53 mL, 17.8 mmol, 2.0 equiv), DMSO (1.90 mL, 26.7 mmol, 3.0 equiv) and triethylamine (6.20 mL, 50.0 mmol, 5.00 equiv) in dichloromethane (35.0 mL x 2) afford the product **55e** (0.83 g, 7.60 mmol, 85%) as a colorless oil. R_f= 0.5 (7:3 hexane/EtOAc). Spectral data for **55e**: ^{1}H NMR (500 MHz, Chloroform-*d*) δ 9.78 (t, J = 1.7 Hz, 1H), 2.47 (td, J = 7.3, 1.7 Hz, 2H), 2.23 (td, J = 7.0, 2.7 Hz, 2H), 1.96 (t, J = 2.7 Hz, 1H), 1.82 - 1.71 (m, 2H), 1.64 - 1.46 (m, 2H). The spectral data match those previously reported for this compound.[17]

*Preparation of aldehyde **55f***

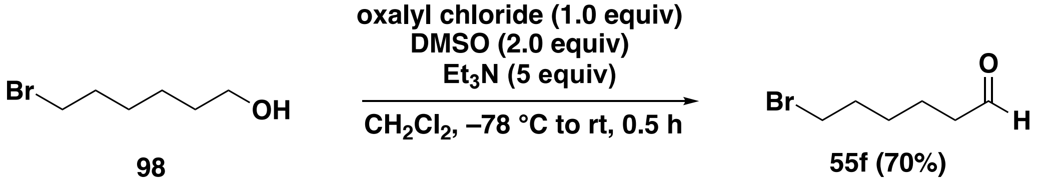

6-Bromohexanal **55f**: Following *General procedure C,* the reaction of 6-bromohexan-1-ol **98** (1.30 mL g, 10.0 mmol, 1.00 equiv.), oxalyl chloride (0.87 mL, 10.0 mmol,1.00 equiv.), DMSO (1.40 mL, 20.0 mmol, 2.00 equiv.) and triethylamine (6.90

mL, 50.0 mmol, 5.0 equiv.) in dichloromethane (20.0 mL x 2) afford the product **55f** (1.24 g, 7.00 mmol, 70%) as a colorless oil. R_f= 0.5 (7:3 hexane/EtOAc). Spectral data for **55f**: ^{1}H NMR (500 MHz, Chloroform-*d*) δ 9.76 (s, 1H), 3.39 (t, *J* = 6.7 Hz, 2H), 2.50 - 2.40 (m, 2H), 1.92 - 1.82 (m, 2H), 1.73 - 1.59 (m, 2H), 1.55 - 1.40 (m, 2H). These spectral data match those previously reported for this compound.[18]

*Preparation of aldehyde **55g***

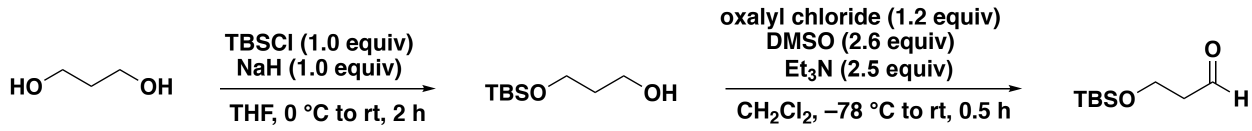

99 100 (96%) 55g (80%)

3-((*tert*-Butyldimethylsilyl)oxy)propanal **55g**: To a THF (12.5 mL) solution of propane-1,3-diol **99** (1.8 mL, 25 mmol, 1.00 equiv) was added a THF (25 mL) solution of NaH (1 g, 25 mmol, 1.00 equiv) drop wise at 0 °C. The mixture was stirred for 45 minutes at room temperature and then was added a TBSCl (3.75 g, 25.0 mmol, 1.00 equiv) solution in THF (12.5 mL). The reaction was stirred for 1 hour and quenched with sat $NaHCO_3$ at 0 °C. After the two layers were separated, the aqueous layer was extracted with diethyl ether (15.0 mL x 3). The combined organic layer was dried over $NaSO_4$ and concentrated under rotary evaporation. Flash chromatography (silica gel, 20 mm x 160 mm, hexane/EtOAc 75:25) afforded the pure mono-protected diol **100** (4.60 g, 24.0 mmol, 96%) as a colorless oil. R_f= 0.2 (7:3 hexane/EtOAc). Spectral data for **100**: ^{1}H NMR (500 MHz, Chloroform-*d*) δ 3.87 - 3.76 (m, 4H), 1.77 (p, *J* = 5.6 Hz, 2H), 0.89 (s, 9H), 0.10 - 0.05 (m, 6H). These spectral data match those previously reported for this compound.[19]

Following *General procedure C*, the reaction of **100** (4.60 g, 24.0 mmol, 1.00 equiv), oxalyl chloride (2.60 mL, 28.8 mmol,1.20 equiv), DMSO (4.3 mL, 62.4 mmol, 2.6 equiv) and triethylamine (8.50 mL, 62.4 mmol, 2.6 equiv) in dichloromethane (59.0 mL x 2) afford the product **55g** (3.60 g, 19.2 mmol, 80%) as a colorless oil. R_f= 0.8 (7:3 hexane/EtOAc). Spectral data for **55g**: ^{1}H NMR (500 MHz, Chloroform-*d*) δ 9.80 (s, 1H), 3.98 (t, *J* = 6.0 Hz, 2H), 2.59 (td, *J* = 6.1, 2.0 Hz, 2H), 0.88 (s, 9H), 0.06 (s, 6H). These spectral data match those previously reported for this compound.[19]

*Preparation of aldehyde **55h***

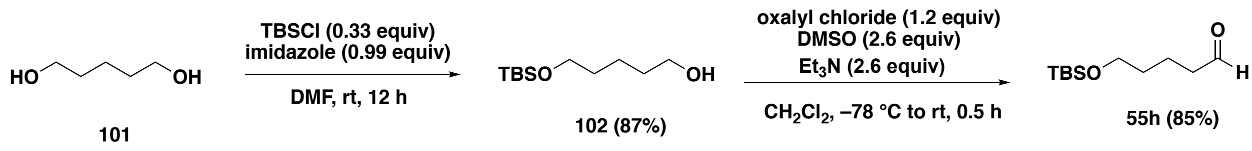

5-((*tert*-Butyldimethylsilyl)oxy)pentanal **55h**: To an oven-dried 250 mL round bottomed flask were added pentane-1,5-diol **101** (10.0 mL, 95.4 mmol, 1.00 equiv), imidazole (6.46 g, 94.8 mmol, 0.99 equiv) and DMF (40 mL). After all solids were dissolved, TBSCl (4.84 g, 32.0 mmol, 0.33 equiv) in 40 mL DMF was added drop wise to the solution of the diol. The whole mixture was stirred overnight at the room temperature and quenched with water (40.0 mL) and pentane (40.0 mL). The aqueous layer was extract with pentane (20.0 mL x 3) and the combined organic layer was dried over $NaSO_4$ and concentrated under rotary evaporation. Flash chromatography (silica gel, 60 mm x 200 mm, hexane/EtOAc 80:20) afforded the pure mono-protected diol **102** (18.1 g, 83.0 mmol, 87%) as a colorless oil. R_f= 0.6 (5:5 hexane/EtOAc). Spectral data for **102**: ^{1}H NMR (500 MHz, Chloroform-*d*) δ 3.60 (dt, *J* = 8.2, 6.6 Hz, 4H), 1.62 - 1.44

(m, 4H), 1.43 - 1.31 (m, 2H), 0.86 (d, *J* = 1.2 Hz, 9H), 0.05 - -0.04 (m, 6H). The spectral data match those previously reported for this compound.[20]

Following *General procedure C*, the reaction of **102** (6.0 g, 21.0 mmol, 1.00 equiv), oxalyl chloride (2.31 mL, 25.2 mmol,1.2 equiv), DMSO (3.76 mL, 54.6 mmol, 2.60 equiv) and triethylamine (7.50 mL, 54.6 mmol, 2.60 equiv) in dichloromethane (52.0 mL x 2) afford the product **55h** (3.85 g, 17.9 mmol, 85%) as a colorless oil. R_f= 0.8 (7:3 hexane/EtOAc). Spectral data for **55h**: ^{1}H NMR (500 MHz, Chloroform-*d*) δ 9.76 (s, 1H), 3.61 (td, *J* = 6.3, 1.1 Hz, 2H), 2.45 (td, *J* = 7.4, 1.8 Hz, 2H), 1.74 - 1.64 (m, 2H), 1.59 - 1.48 (m, 2H), 0.88 (s, 9H), 0.03 (s, 6H). These spectral data match those previously reported for this compound.[20]

*Preparation of aldehyde **55i***

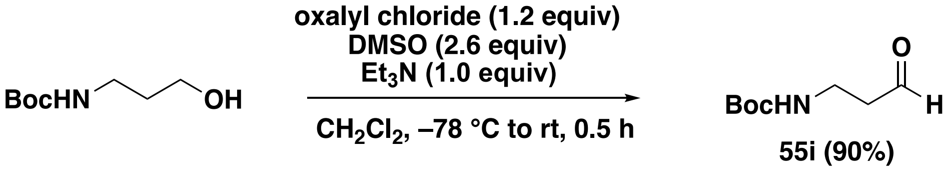

103

tert-Butyl (3-oxopropyl)carbamate **55i**: Following *General procedure C*, the reaction of **103** (3.50 g, 20.1 mmol, 1.00 equiv), oxalyl chloride (1.50 mL, 24.1 mmol, 1.20 equiv), DMSO (3.60 mL, 51.0 mmol, 2.00 equiv) and triethylamine (5.40 mL, 20.3 mmol, 1.00 equiv) in dichloromethane (20.0 mL x 2) afford the product **55i** (3.1 g, 18.1 mmol, 90%) as a colorless oil. R_f= 0.5 (5:5 hexane/EtOAc). Spectral data for **55i**: ^{1}H NMR (500 MHz, Chloroform-*d*) δ 9.77 (s, 1H), 3.38 (t, *J* = 5.9 Hz, 2H), 2.67 (t, *J* = 6.1 Hz, 2H), 1.39 (s, 9H). ^{13}C NMR (126 MHz, Chloroform-*d*) δ 201.45, 44.32, 33.98, 28.41

(one sp^2 and one sp^3 carbon are not located). These spectral data match those previously reported for this compound.[19]

*Preparation of aldehyde **55j***

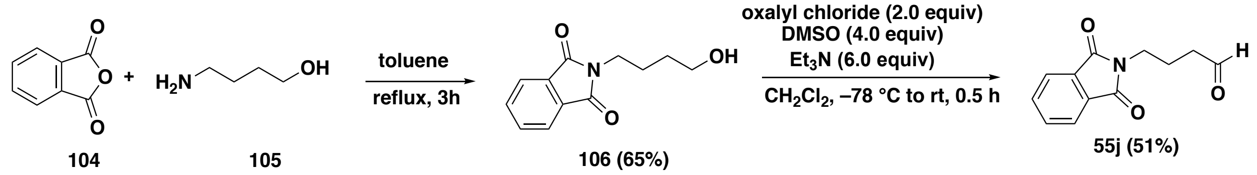

4-(1,3-Dioxoisoindolin-2-yl)butanal **55j**: To an oven-dried 50 mL round bottomed flask were added isobenzofuran-1,3-dione **104** (0.74 g, 5.00 mmol, 1.00 equiv), 4-aminobutan-1-ol **105** (0.45 g, 5.00 mmol, 1.00 equiv) and toluene (20.0 mL). The reaction was refluxed for 3 hours and concentrated under rotary evaporation. Flash chromatography (silica gel, 20 mm x 160 mm, pure EtOAc) afforded pure **106** (0.71 g, 3.30 mmol, 65%) as a white solid. R_f= 0.1 (pure EtOAc). Spectral data for **106**: ^{1}H NMR (500 MHz, Chloroform-*d*) δ 7.87 - 7.80 (m, 2H), 7.75 - 7.67 (m, 2H), 3.74 (t, *J* = 7.2 Hz, 2H), 3.69 (t, *J* = 6.4 Hz, 2H), 1.78 (p, *J* = 7.3 Hz, 2H), 1.62 (p, *J* = 7.3 Hz, 2H). The spectral data match those previously reported for this compound.[21]

Following *General procedure C*, the reaction of 2-(4-hydroxybutyl)isoindoline-1,3-dione **106** (0.72 g, 3.30 mmol, 1.00 equiv), oxalyl chloride (0.57 mL, 6.60 mmol, 2.0 equiv), DMSO (0.93 mL, 13.2 mmol, 4.0 equiv) and triethylamine (2.74 mL, 19.8 mmol, 6.0 equiv) in dichloromethane (15.0 mL x 2) afforded the product **55j** (0.37 g, 1.70 mmol, 51%) as a colorless oil. R_f= 0.8 (pure EtOAc). Spectral data for **55j**: ^{1}H NMR (500 MHz, Chloroform-*d*) δ 9.77 (s, 1H), 7.94 - 7.79 (m, 2H), 7.75 - 7.69 (m, 2H), 3.74 (t, *J* = 6.8 Hz, 2H), 2.54 (t, *J* = 7.2 Hz, 2H), 2.02 (p, *J* = 7.0 Hz, 2H). ^{13}C NMR (126 MHz, Chloroform-*d*)

δ 200.86, 168.37, 134.03, 131.97, 123.29, 41.09, 37.11, 21.16. These spectral data match those previously reported for this compound.[21]

*Preparation of aldehyde **55k***

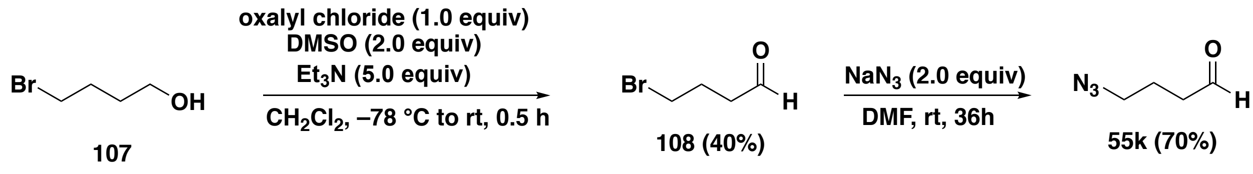

4-Azidobutanal **55k**: Following *General procedure C*, the reaction of 4-bromobutan-1-ol **107** (1.1 mL g, 10.0 mmol, 1.00 equiv), oxalyl chloride (0.87 mL, 10.0 mmol, 1.00 equiv), DMSO (1.4 mL, 20.0 mmol, 2.00 equiv) and triethylamine (6.9 mL, 50.0 mmol, 5.0 equiv) in dichloromethane (15 mL x 2) afforded the product **108** (0.6 g, 4.0 mmol, 40%) as a colorless oil. R_f= 0.5 (7:3 hexane/EtOAc). Spectral data for **108**: ^{1}H NMR (500 MHz, Chloroform-*d*) δ 9.82 (s, 1H), 3.46 (t, *J* = 6.4 Hz, 2H), 2.68 (t, *J* = 7.0 Hz, 2H), 2.18 (p, *J* = 6.7 Hz, 2H). The spectral data match those previously reported for this compound.[22]

To an oven-dried 50 mL round bo ttomed flask were added 4-bromobutanal **108** (0.6 g, 4.0 mmol, 1.00 equiv), sodium azide (0.51 g, 8.0 mmol, 2.00 equiv) and DMF (16.0 mL). The reaction was stirred at room temperature for 16 hours and washed with water/diethyl ether (20.0 mL x 3, 1:1). The combined organic layer was dried over $NaSO_4$ and concentrated under rotary evaporation. Flash chromatography (silica gel, 20 mm x 160 mm, hexane/EtOAc 3:7) afforded pure **55k** (0.32 g, 2.80 mmol, 70%) as a colorless oil. R_f= 0.2 (7:3 hexane/EtOAc). Spectral data for **55k**: ^{1}H NMR (500 MHz, Chloroform-*d*) δ 9.80 (s, 1H), 3.35 (d, *J* = 6.7 Hz, 2H), 2.58 (t, *J* = 7.1 Hz, 2H), 1.92 (p, *J* = 6.9 Hz, 2H). The spectral data match those previously reported for this compound.[23]

Preparation of aldehyde ***55l***

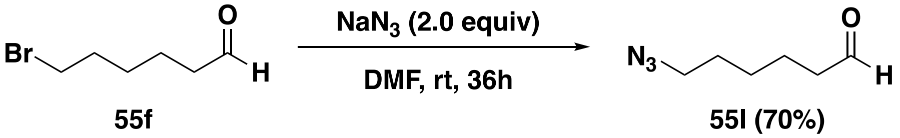

6-azidohexanal **55l**: To an oven-dried 50 mL round bottomed flask were added 6-bromohexanal **55f** (vide supra, 0.35 g, 2.00 mmol, 1.00 equiv), sodium azide (0.26 g, 4.00 mmol, 2.00 equiv) and DMF (8.0 mL). The reaction was stirred at room temperature for 16 hours and washed with water/diethyl ether (10.0 mL x 3, 1:1). The combined organic layer was dried over $NaSO_4$ and concentrated under rotary evaporation. Flash chromatography (silica gel, 20 mm x 160 mm, hexane/EtOAc 3:7) afforded the pure **55l** (0.20 g, 1.40 mmol, 70%) as a colorless oil. R_f= 0.2 (7:3 hexane/EtOAc). Spectral data for **55l**: ^{1}H NMR (500 MHz, Chloroform-*d*) δ 9.78 (s, 1H), 3.28 (t, *J* = 6.8 Hz, 2H), 2.50 - 2.43 (m, 2H), 1.71 - 1.59 (m, 4H), 1.47 - 1.36 (m, 2H). The spectral data match those previously reported for this compound.[24]

Preparation of aldehyde ***55m***

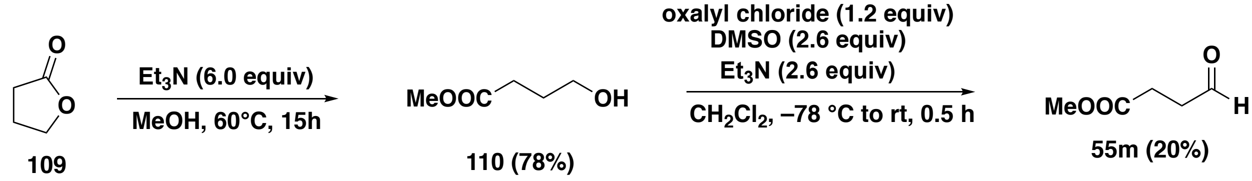

Methyl 4-oxobutanoate **55m**: To a solution of methanol (50 mL) and dihydrofuran-2(3*H*)-one **109** (0.76 mL, 10 mmol, 1.00 equiv) was added triethylamine (8.40 mL, 60.0 mmol, 6.0 equiv). The reaction was then heated up to 60°C and stirred

for 15 hours. After the mixture was cooled down to room temperature, it was diluted with hexane (50 mL) and concentrated under the rotary evaporation. The crude product **110** (0.90 g, 7.80 mmol, 78%) can be directly used into the next reaction without purification. R_f= 0.2 (5:5 hexane/EtOAc).

Following *General procedure C*, the reaction of **110** (0.92 g, 7.80 mmol, 1.00 equiv\), oxalyl chloride (0.68 mL, 9.40 mmol, 1.20 equi.), DMSO (1.10 mL, 20.3 mmol, 2.60 equiv) and triethylamine (5.4 mL, 20.3 mmol, 2.60 equiv) in dichloromethane (11.7 mL x 2) afforded the product **55m** (0.18 g, 1.6 mmol, 20%) as a colorless oil. The low yield was due to the loss of the product during the rotary evaporation given the low boiling point of the product. R_f= 0.7 (5:5 hexane/EtOAc). Spectral data for **55m**: ^{1}H NMR (500 MHz, Chloroform-*d*) δ 9.82 (s, 1H), 3.70 (s, 3H), 2.81 (t, *J* = 6.6 Hz, 2H), 2.64 (t, *J* = 6.6 Hz, 2H). ^{13}C NMR (126 MHz, Chloroform-*d*) δ 199.95, 172.70, 51.95, 38.52, 26.30. These spectral data match those previously reported for this compound.[19]

5. The Asymmetric Kabachnik-Fields Reactions with Aromatic Aldehydes 51a-j

5a. Effect of molecular Sieves

In Table 1 in the text in entries 2 and 3 it was observed that the asymmetric induction from the reaction of benzaldehyde with amine **13b** and diethyl phosphite the increased from 67% to 82% when the amount of molecular sieves was increased from 100 weight % relative to **13b** to 82% with 200 weight %. However, further increasing the amount of molecular sieves to 400% did not significant change the result, and further, increasing the amount of sieves to 600 weight % resulted in a sharp decrease in the asymmetric induction to 47% ee (Table S1, entries 3 and 4).

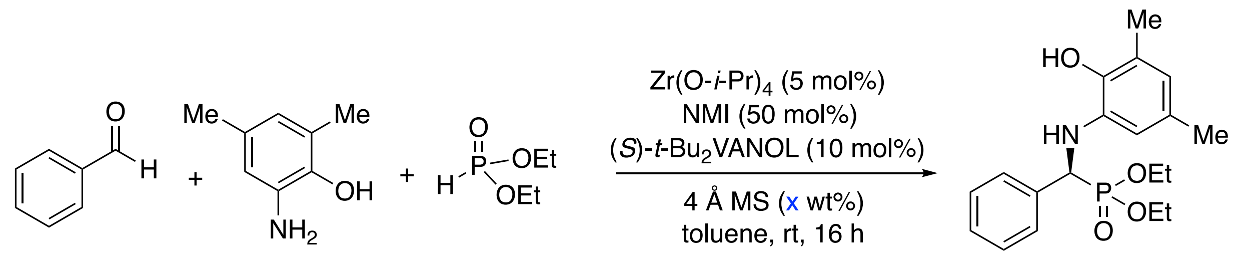

Table S1 Screen of equivalent of 4Å molecular sieves with 13b

entry	x wt% [a]	% yield [b]	% ee
1	100	55	67
2	200	87	82
3	400	88	79
4	600	84	47

[a] x wt % MS relative to the aniline. [b] Isolated yield.

5b. Effect of Mono-Substitued Hydroxy-Anilines on the Reaction of Benzaldehyde.

Given the fact that it was found that the mono-substituted aniline **13l** was optimal for the Kabachnik-Fields reaction of aliphatic aldehydes (Scheme 5 in text) and the fact that these anilines were not screened during the optimization of the reaction with aromatic aldehydes, the reaction of benzaldehyde was screened with these mono-substituted anilines and the results are given in Scheme S1. The optimal di-substituted aniline for the reaction of benzaldehyde was the 3,5-diisopropyl-2-hydroxyaniline **13c** (see Table 3 in the text) which gave phosphonate **51a** in 90% yield and 93% ee. The optimal mono-substituted hydroxyaniline found in this screen was 3-*t*-butyl-2-hydroxyaniline **13l** which gave the Kabachnik-Fields product with benzaldehyde with the

same induction (93%) as previously optimized di-substituted aniline **13c** but with a reduced yield of 62%.

Scheme S1

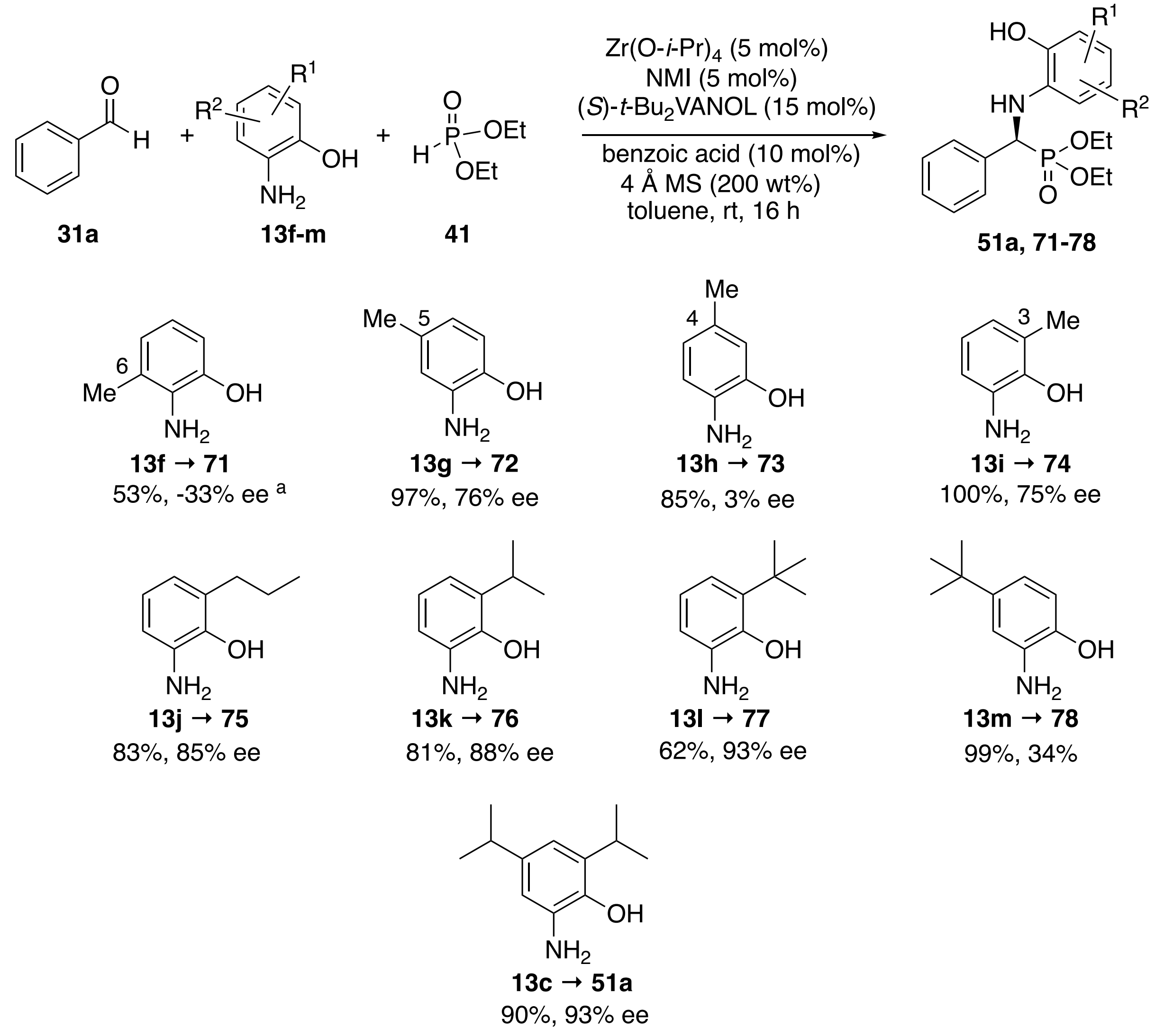

[a] (*R*)-*t*-Bu$_2$VANOL lignad was used and *ent*-**71** was obtained.

5c. Effect of Phosphite Esters on the Kabachnik-Fields Reaction of Benzaldehyde.

A number of phosphite esters were screened in the reaction of benzaldehyde with aniline **13c** and the results are presented in Table S2. These reactions were not

performed with benzoic acid. The results indicated that dimethyl phosphite gives the lowest asymmetric induction of those examined. The primary phosphite esters **41a** (ethyl) and **41d** (*n*-butyl) both gave 90% ee for the phosphonate product while the secondary ester **41c** (*i*-propyl) gave about the same induction (91%) but with a reduced yield of 45%. The tertiary ester **41e** was unreactive.

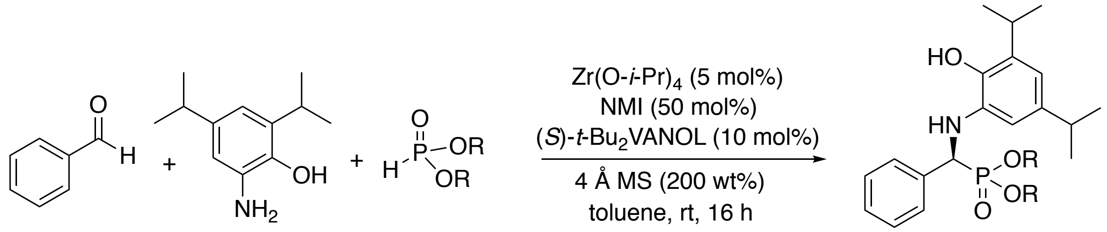

31a **13c** **41** **51**

Table S2 The Effect of Phosphite Estes on the Kabachnik-Fields Reaction. [a]

entry	R	phosphite	product	% yield [b]	% ee [c]
1	Me	**41b**	**51b**	76	79
2	Et	**41a**	**51a**	71	90
3	*i*-Pr	**41c**	**51c**	45	–91 [d]
4	*n*-Bu	**41d**	**51d**	65	–90 [d]
5	*t*-Bu	**41e**	**51e**	0	—

[a] Unless otherwise specified, all reactions were carried out on 0.1 mmol of aldehyde with 1.0 equiv of aniline and 1.0 equiv of phosphite and with 200 wt% of 4 Å MS relative to the aniline. The catalyst was prepared by stirring a mixture of 1 equiv of $Zr(O\text{-}i\text{-}Pr)_4$•HO-*i*-Pr, 10 equiv of *N*-methyl imidazole (NMI) and 2 equiv of (*S*)-*t*-Bu_2VANOL in dry toluene for 30 min at rt under air. [b] Isolated yield. [c] 100 wt % MS relative to the aniline. [d] (*R*)-*t*-Bu_2VANOL ligand was used.

5d *General procedure D for assembling the t-Bu_2VANOL-zirconium catalyst:*

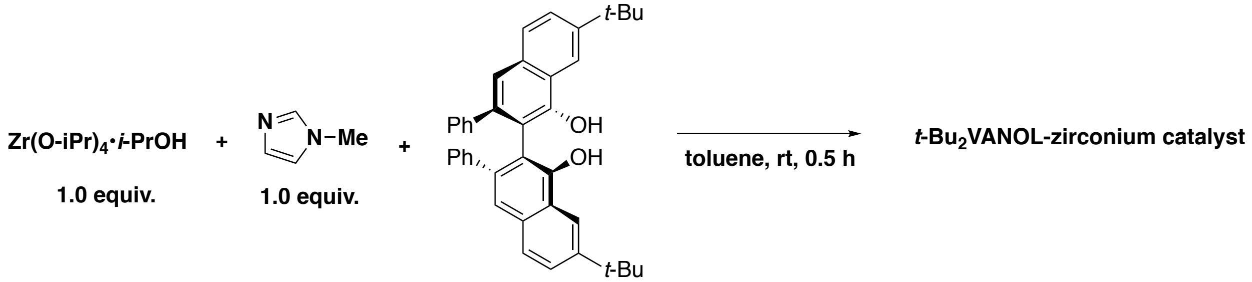

(*S*)-*t*-Bu_2VANOL 37
3.0 equiv

Zr(O*i*Pr)$_4$(HO*i*Pr) zirconium(IV) isopropoxide-isopropanol complex (38.8 mg, 0.1 mmol, 1.00 equiv), (*S*)-*t*-Bu$_2$VANOL **37** (165 mg, 0.30 mmol, 3.0 equiv), and 4 mL toluene were mixed under air at room temperature in an oven-dried 20-mL vial. After all of the solids were dissolved in the toluene, *N*-methylimidazole (8.00 µL, 0.1 mmol, 1.00 equiv) was added via a micro-syringe. The resulting mixture was stirred at room temperature under air for 30 min before being employed in the asymmetric catalytic Kabachnik-Fields reactions. The proper amount of catalyst is then removed for a given reaction and the remainder of the catalyst can be stored in toluene at –20°C under air for later use.

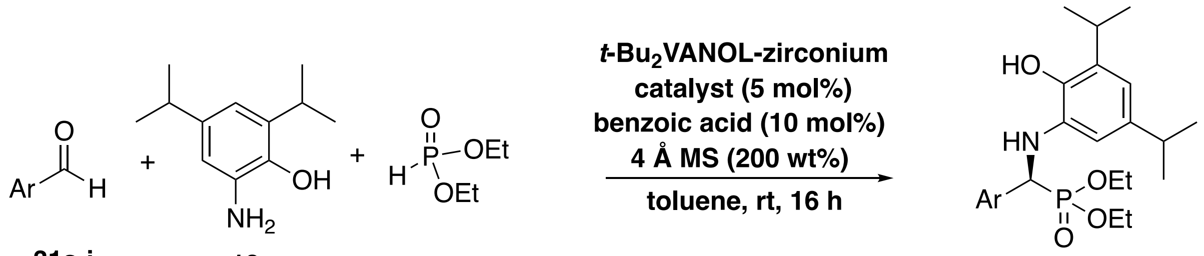

31a-j **13c** **41** **51a-j**

5e General procedure E for the asymmetric Kabachnik-Fields reaction of aromatic aldehydes: To a 10-mL flame-dried home-made Schlenk flask, prepared from a single necked 25 mL pear-shaped flask that had its 14/20 glass joint replaced with a high vacuum threaded Teflon valve, flushed with nitrogen was added 2-hydroxy-3,5-di*iso*propylaniline **13c** (19.3 mg, 0.10 mmol, 1.00 equiv), 4 Å molecular sieves (38.6 mg) and benzoic acid (1.20 mg, 0.01 mmol, 0.10 equiv). Under a nitrogen flow, the zirconium catalyst in toluene (0.20 mL, 5 mol%, 0.25 M, prepared from general procedure D), the desired aldehyde (0.10 mmol, 1.00 equiv) and diethyl phosphite (14.0 µL, 0.10 mmol, 1.00 equiv) were added to the mixture by syringe. The flask was sealed, and then placed into a liquid nitrogen bath. After the solvent was frozen, the Teflon

valve was opened to vacuum and the atmosphere was pumped off for 5 minutes. The flask was sealed again and removed from the liquid nitrogen bath. Gas bubbles could be seen to evolve from the solution during thawing of the solvent. When all the solvent melted, the flask was placed back into the liquid nitrogen bath and this Freeze-Pump-Thaw degassing process was repeated for another 2 times. The reaction was then stirring at room temperature for 16 hours and diluted with hexane (5 mL). The mixture was concentrated under rotary evaporation and purified by flash chromatography (silica gel, 10 mm x 100 mm, hexane/EtOAc, 7:3) to yield the corresponding α-amino phosphites. Each reaction was run twice, each with a different enantiomer of the catalyst. In this way we could determine the retention time for each enantiomer of the product and these are given in each experimental procedure. In each case, base-line separation of the enantiomers was realized.

Diethyl(S)-(((2-hydroxy-3,5-diisopropylphenyl)amino)(phenyl)methyl)phosphonate **51a**:

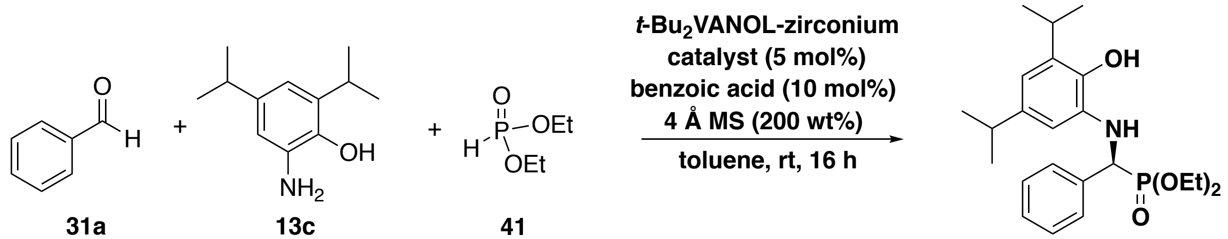

The reaction with benzaldehyde **31a** (12.0 μL, 0.10 mmol, 1.00 equiv) according to general procedure E afforded **51a** (38.6 mg, 0.092 mmol) in 92% yield and 93% *ee* as a yellow oil. R_f = 0.30 (hexane/EtOAc 7:3); Specific Rotation: $[\alpha]_D^{20}$= -12.8 (c 1.0, $CHCl_3$); Spectral data for **51a**: ^{1}H NMR (500 MHz, Chloroform-*d*) δ 7.48 (dd, *J* = 7.2, 3.0

Hz, 2H), 7.31 - 7.23 (m, 3H), 6.55 (d, *J* = 2.2 Hz, 1H), 6.29 (s, 1H), 4.69 (d, *J* = 22.7 Hz, 1H), 4.27 - 4.14 (m, 2H), 4.00 - 3.91 (m, 1H), 3.76 - 3.65 (m, 1H), 3.22 (hept, *J* = 7.0 Hz, 1H), 2.64 (hept, *J* = 7.0 Hz, 1H), 1.30 (t, *J* = 7.1 Hz, 3H), 1.21 (d, *J* = 6.8 Hz, 3H), 1.18 (d, *J* = 6.9 Hz, 3H), 1.11 (t, *J* = 7.1 Hz, 3H), 1.06 (d, *J* = 6.9 Hz, 3H), 1.03 (d, *J* = 6.9 Hz, 3H) ^{13}C NMR (126 MHz, Chloroform-*d*) δ 141.72, 140.66, 133.36, 130.15, 128.55, 128.53, 128.40, 128.34, 128.29, 128.03, 116.27, 112.37, 63.56 (d, *J* = 7.1 Hz), 58.42, 57.20, 33.68, 27.18, 24.15, 24.06, 22.86, 22.68, 16.42 (d, *J* = 6.0 Hz), 16.18 (d, *J* = 5.8 Hz). IR (thin film) 3372br, 2959s, 2868s, 1596vs, 1453s, 1200vs, 975s cm^{-1}; HRMS (ESI-TOF) *m/z* found 442.2130 ([M+Na]$^+$); calcd. 442.2123 for $C_{23}H_{34}NO_4PNa$. HPLC: DAICEL CHIRALPAK AD-H (Hexane/*i*PrOH = 95:5, 0.7 mL/min flow rate, 245 nm), 10.3 min for the minor peak and 16.9 min for the major peak.

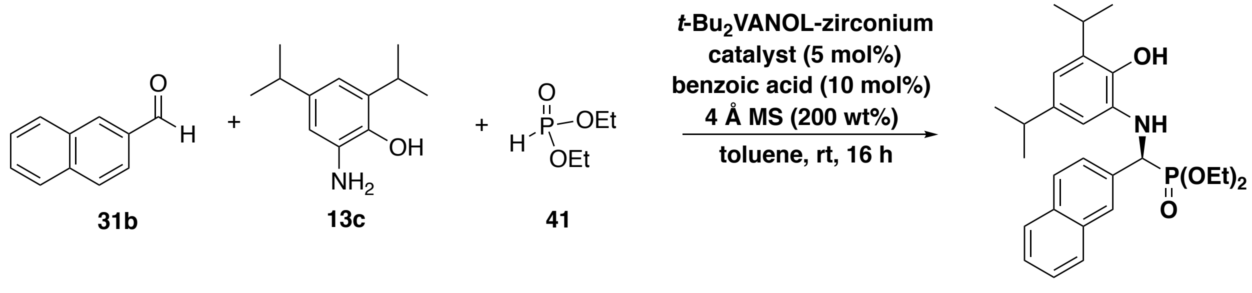

31b **13c** **41** **51b (81%)** **95% ee**

Diethyl (*S*)-(((2-hydroxy-3,5-diisopropylphenyl)amino)(naphthalen-2-yl)methyl) phosphonate **51b**: Reaction of 2-naphthaldehyde **31b** (15.6 mg, 0.10 mmol, 1.00 equiv) according to general procedure E afforded **51b** (38.0 mg, 0.081 mmol) in 81% yield and 95% *ee* as a yellow oil. R_f = 0.30 (hexane/EtOAc 7:3); Specific Rotation: $[\alpha]_D^{20}$= -25.3 (c 1.00, $CHCl_3$); Spectral data for **51b**: ^{1}H NMR (500 MHz, Chloroform-*d*) δ 7.92 (s, 1H), 7.74 (dd, *J* = 8.4, 3.9 Hz, 2H), 7.65 (d, *J* = 8.5 Hz, 1H), 7.59 (d, *J* = 8.2 Hz, 1H), 7.41 (t, *J* = 7.5 Hz, 1H), 7.35 (t, *J* = 7.5 Hz, 1H), 6.54 (d, *J* = 2.1 Hz, 1H), 6.38 (d, *J* = 2.1 Hz,

1H), 4.92 (d, J = 22.3 Hz, 1H), 4.30 - 4.15 (m, 2H), 4.01 - 3.91 (m, 1H), 3.77 - 3.66 (m, 1H), 3.22 (hept, J = 6.4 Hz, 1H), 2.58 (hept, J = 7.0 Hz, 1H), 1.32 (t, J = 7.1 Hz, 3H), 1.20 (d, J = 6.9 Hz, 3H), 1.13 - 1.04 (m, 6H), 0.97 (t, J = 6.7 Hz, 5H). ^{13}C NMR (126 MHz, Chloroform-*d*) δ 140.84, 133.14, 128.32, 128.06, 127.90, 127.85, 127.50, 126.15, 126.09, 126.03, 125.99, 63.73, 58.81, 57.58, 33.59, 27.16, 24.02, 22.90, 22.57, 16.45, 16.23 (five sp^2 and one sp^3 carbon are not located). IR (thin film) 3372br, 2959s, 1597vs, 1508s, 1198vs, 1024vs, 976s cm^{-1}; HRMS (ESI-TOF) *m/z* found 470.2462 ($[M+H]^+$); calcd. 470.2460 for $C_{27}H_{37}NO_4P$. HPLC: DAICEL CHIRALPAK AD-H (Hexane/*i*PrOH = 5:95, 0.7 mL/min flow rate, 245 nm), 15.5 min for the minor peak and 26,7 min for the major peak.

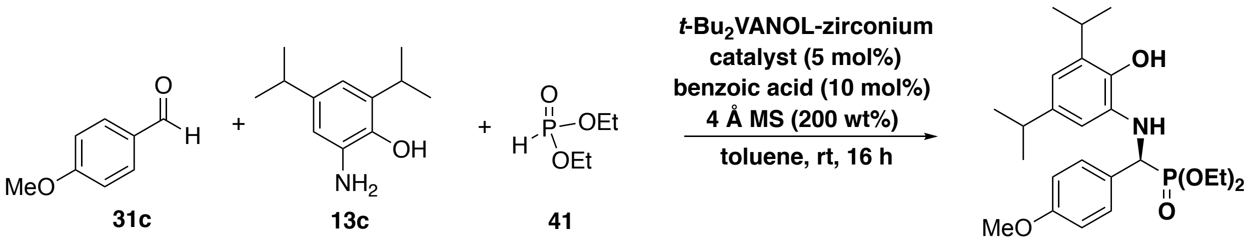

31c **13c** **41**

51c (90%)
90% ee

Diethyl (*S*)-(((2-hydroxy-3,5-diisopropylphenyl)amino)(4methoxyphenyl)methyl) phosphonate **51c**: Reaction of 4-methoxybenzaldehyde **31c** (12.4 μL, 0.100 mmol, 1.00 equiv) according to general procedure E afforded **51c** (40.4 mg, 0.09 mmol) in 90% yield and 90% *ee* as a yellow oil. R_f = 0.30 (hexane/EtOAc 7:3); Specific Rotation: $[\alpha]_D^{20}$= -37.6 (c 1.00, $CHCl_3$); Spectral data for **51c**: ^{1}H NMR (500 MHz, Chloroform-*d*) δ 7.38 (dd, J = 8.7, 2.2 Hz, 2H), 6.76 (d, J = 8.4 Hz, 2H), 6.53 (d, J = 2.1 Hz, 1H), 6.29 (d, J = 2.1 Hz, 1H), 4.62 (d, J = 22.6 Hz, 1H), 4.18 (tqd, J = 10.3, 7.2, 3.4 Hz, 2H), 3.95 (dp, J = 10.6, 7.1 Hz, 1H), 3.73 (s, 4H), 3.21 (hept, J = 6.8 Hz, 1H), 2.66 (p, J = 6.9 Hz, 1H), 1.30 (t, J = 7.1 Hz, 3H), 1.21 (d, J = 6.8 Hz, 3H), 1.17 - 1.10 (m, 6H), 1.08 (d, J = 6.9 Hz,

3H), 1.05 (d, J = 6.9 Hz, 3H). ^{13}C NMR (126 MHz, Chloroform-d) δ 159.30, 141.66, 140.54, 129.47, 129.42, 127.42, 113.99, 113.97, 113.94, 113.92, 63.45 (dd, J = 7.2, 4.2 Hz), 57.63, 56.39, 55.20, 33.70, 27.11, 24.20, 24.10, 22.91, 22.68, 16.44 (d, J = 5.8 Hz), 16.26 (d, J = 5.7 Hz). IR (thin film) 3408br, 2959s, 2065s, 1636vs, 1510s, 1205vs, 974s cm^{-1}; HRMS (ESI-TOF) *m/z* found 450.2410 ($[M+H]^+$); calcd. 450.2409 for $C_{23}H_{37}NO_5P$. HPLC: DAICEL CHIRALPAK AD-H (Hexane/*i*PrOH = 95:5, 0.7 mL/min flow rate, 245 nm), 16.4 min for the minor peak and 29.8 min for the major peak.

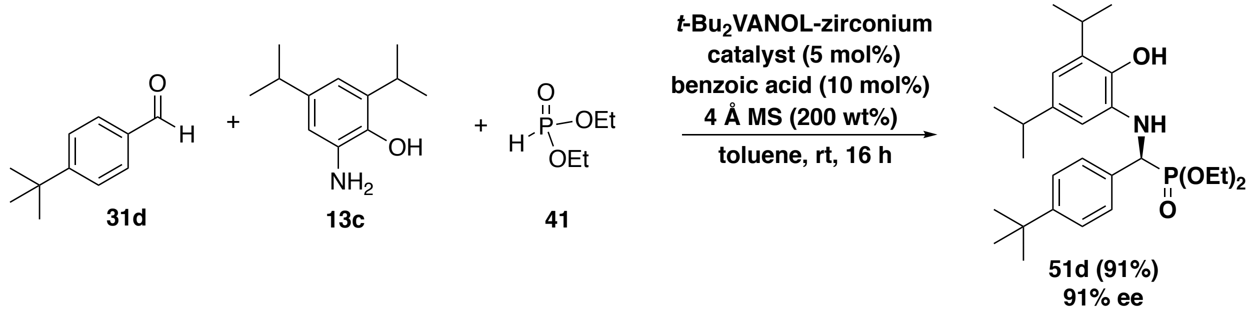

Diethyl (*S*)-((4-(*tert*-butyl)phenyl)((2-hydroxy-3,5 diisopropylphenyl)amino)methyl) phosphonate **51d**: Reaction of 4-(*tert*-butyl)benzaldehyde **31d** (16.8 μL, 0.10 mmol, 1.00 equiv) according to general procedure E afforded **51d** (43.2 mg, 0.091 mmol) in 91% yield and 91% *ee* as a yellow oil. R_f = 0.30 (hexane/EtOAc 7:3); Specific Rotation: $[\alpha]_D^{20}$= -2.5 (c 1.00, $CHCl_3$); Spectral data for **51d**: ^1H NMR (500 MHz, Chloroform-d) δ 7.38 (d, J = 6.3 Hz, 2H), 7.21 (d, J = 7.9 Hz, 2H), 6.50 (s, 1H), 6.26 (s, 1H), 4.67 (d, J = 23.2 Hz, 1H), 4.27 - 4.12 (m, 2H), 4.00 - 3.89 (m, 1H), 3.75 - 3.64 (m, 1H), 3.21 (hept, J = 6.8 Hz, 1H), 2.65 (hept, J = 6.8, 5.7 Hz, 1H), 1.31 (t, J = 7.1 Hz, 3H), 1.23 (s, 9H), 1.19 (d, J = 7.1 Hz, 3H), 1.11 (d, J = 7.0 Hz, 6H), 1.07 (d, J = 6.8 Hz, 3H), 1.03 (d, J = 6.7 Hz, 3H). ^{13}C NMR (126 MHz, Chloroform-d) δ 150.77, 141.41, 140.34, 134.37, 132.48, 127.90, 127.85, 125.36, 115.37, 111.51, 63.48 (dd, J = 24.4, 7.0 Hz), 57.69, 56.46, 34.45, 33.69, 31.27 (3C), 27.00, 24.21, 24.00, 22.97, 22.65, 16.45 (d, J = 5.9 Hz),

16.13 (d, J = 5.7 Hz). IR (thin film) 3420br, 2962s, 1636vs, 1186s, 1038s cm^{-1}; HRMS (ESI-TOF) m/z found 498.2716 ([M+Na]$^+$); calcd. 498.2749 for $C_{27}H_{42}NO_4PNa$. HPLC: DAICEL CHIRALPAK AD-H (Hexane/*i*PrOH = 95:5, 0.7 mL/min flow rate, 245 nm), 9.1 min for the minor peak and 16.7 min for the major peak.

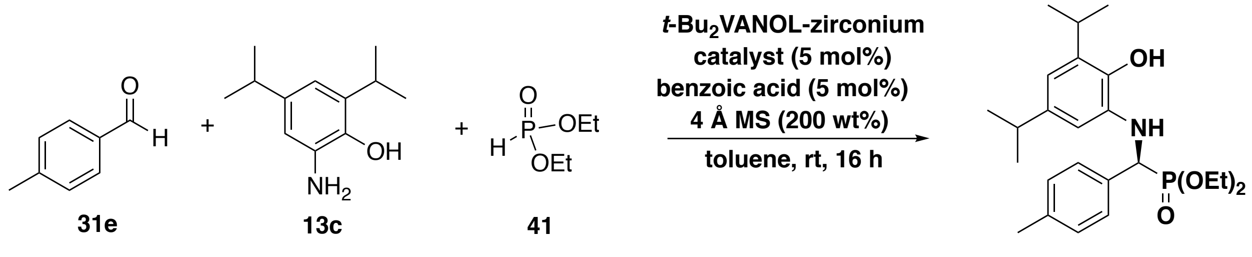

51d (96%)
81% ee

Diethyl (*S*)-(((2-hydroxy-3,5-diisopropylphenyl)amino)(*p*-tolyl)methyl) phosphonate **51e**: Reaction of 4-methylbenzaldehyde **31e** (11.8 μL, 0.10 mmol, 1.00 equiv) according to general procedure E with the exception that only 5 mol% benzoic acid (0.60 mg, 0.005 mmol, 0.05 equiv) was used afforded **51e** (41.6 mg, 0.096 mmol) in 96% yield and 81% *ee* as a yellow oil; R_f = 0.30 (hexane/EtOAc 7:3); Specific Rotation: $[\alpha]_D^{20}$= -40.2 (c 1.00, $CHCl_3$); Spectral data for **51e**: ^{1}H NMR (500 MHz, Chloroform-*d*) δ 7.40 - 7.29 (m, 2H), 7.02 (d, J = 7.7 Hz, 2H), 6.51 (d, J = 2.0 Hz, 1H), 6.28 (d, J = 2.0 Hz, 1H), 4.68 (d, J = 24.0 Hz, 1H), 4.30 - 4.13 (m, 2H), 4.02 - 3.89 (m, 1H), 3.77 - 3.66 (m, 1H), 3.22 (hept, J = 6.9 Hz, 1H), 2.66 (hept, J = 6.9 Hz, 1H), 2.28 (s, 3H), 1.30 (t, J = 7.1 Hz, 3H), 1.20 (d, J = 6.9 Hz, 3H), 1.13 (t, J = 7.3 Hz, 6H), 1.09 (d, J = 6.9 Hz, 3H), 1.06 (d, J = 6.9 Hz, 3H). ^{13}C NMR (126 MHz, Chloroform-*d*) δ 141.32, 140.41, 137.46, 134.42, 132.71, 129.18, 128.09, 128.04, 115.16, 111.27, 63.56 (dd, J = 18.2, 7.1 Hz), 57.70, 56.47, 33.73, 27.02, 24.21, 24.12, 22.95, 22.69, 21.11, 16.45 (d, J = 6.0 Hz), 16.23 (d, J = 5.7 Hz).). IR (thin film) 3374br, 2959s, 1598s, 1511s, 1206vs, 975s cm^{-1}. HRMS (ESI-TOF) m/z found 434.2457 ([M+H]$^+$); calcd 434.2460 for

$C_{24}H_{37}NO_4P$. HPLC: DAICEL CHIRALPAK AD-H (Hexane/*i*PrOH = 95:5, 0.7 mL/min flow rate, 245 nm), 11.6 min for the minor peak and 20.1 min for the major peak.

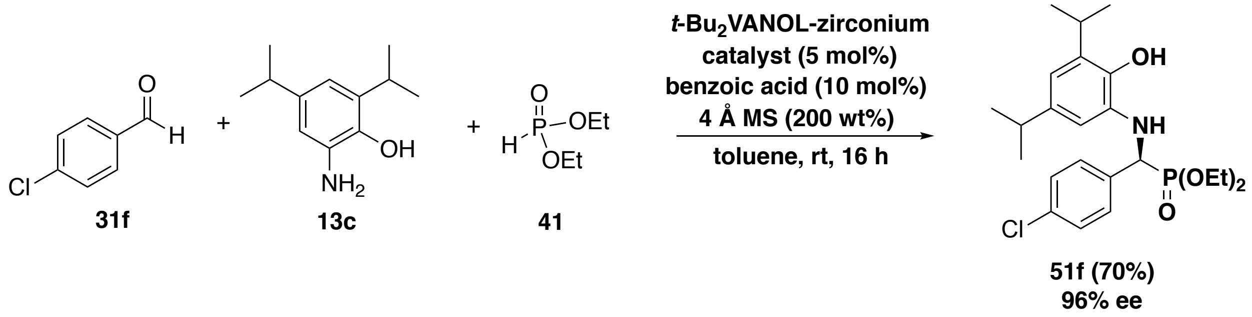

Diethyl (*S*)-((4-chlorophenyl)((2-hydroxy-3,5-diisopropylphenyl)amino)methyl) phosphonate **51f**: Reaction of 4-chlorobenzaldehyde **31f** (11.7 μL, 0.10 mmol, 1.00 equiv) according to general procedure E afforded **51f** (31.7 mg, 0.07 mmol) in 70% yield and 96% *ee* as a yellow oil. R_f = 0.30 (hexane/EtOAc 7:3); Specific Rotation: $[\alpha]_D^{20}$= -18.6 (c 1.00, $CHCl_3$); Spectral data for **51f**: ^1H NMR (500 MHz, Chloroform-*d*) δ 7.50 - 7.45 (m, 1H), 7.42 - 7.37 (m, 1H), 7.28 - 7.18 (m, 4H), 6.58 (d, *J* = 2.1 Hz, 1H), 6.27 (d, *J* = 2.2 Hz, 1H), 4.68 (d, *J* = 22.7 Hz, 1H), 4.23 - 4.12 (m, 2H), 4.06 - 3.96 (m, 1H), 3.88 - 3.77 (m, 1H), 3.19 (hept, *J* = 6.9 Hz, 1H), 2.65 (hept, *J* = 6.9 Hz, 1H), 1.30 (t, *J* = 7.1 Hz, 3H), 1.21 (dd, *J* = 11.7, 6.9 Hz, 6H), 1.16 (t, *J* = 7.1 Hz, 3H), 1.07 (d, *J* = 6.9 Hz, 3H), 1.05 (d, *J* = 6.8 Hz, 3H). ^{13}C NMR (126 MHz, Chloroform-*d*) δ 141.65, 141.06, 134.45, 129.84, 128.51, 128.47, 126.59, 126.55, 117.01, 112.70, 63.78 (d, *J* = 5.9 Hz), 58.11, 56.90, 33.67, 27.26, 24.12, 24.06, 22.81, 22.64, 16.38 (d, *J* = 5.8 Hz), 16.28 (d, *J* = 5.7 Hz) (one sp^3 carbon is not located). IR (thin film) 3405br, 2960s, 1653vs, 1507s, 1207s, 1024s cm^{-1}; HRMS (ESI-TOF) *m/z* found 476.1727 ($[M+H]^+$); calcd. 476.1733 for $C_{23}H_{33}NO_4PClNa$. HPLC: DAICEL CHIRALPAK AD-H (Hexane/*i*PrOH = 95:5, 0.7 mL/min flow rate, 245 nm), 11.5 min for the minor peak and 19.4 min for the major peak.

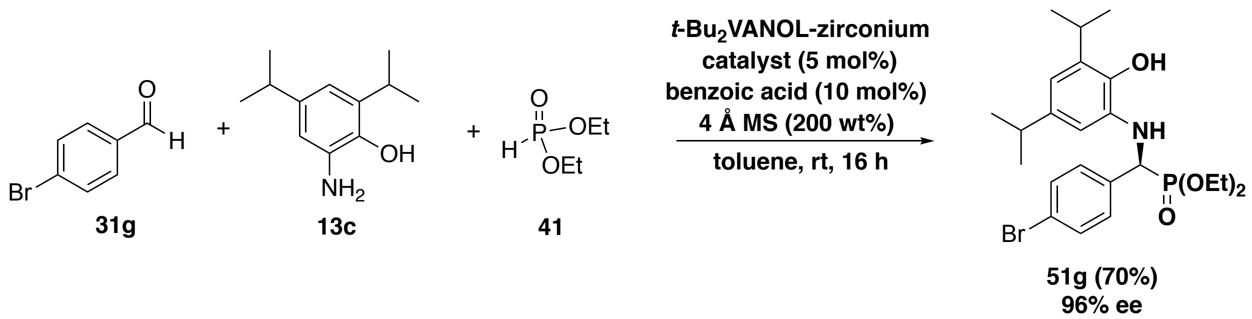

Diethyl (*S*)-((4-bromophenyl)((2-hydroxy-3,5-diisopropylphenyl)amino)methyl) phosphonate **51g**: Reaction of 4-bromobenzaldehyde **31g** (18.3 mg, 0.10 mmol, 1.00 equiv) according to general procedure E afforded **51g** (34.8 mg, 0.07 mmol) in 70% yield and 96% *ee* as a yellow oil. R_f = 0.30 (hexane/EtOAc 7:3); Specific Rotation: $[\alpha]_D^{20}$= -27.5 (c 1.00, $CHCl_3$); Spectral data for **51g**: ^{1}H NMR (500 MHz, Chloroform-*d*) δ 7.34 - 7.21 (m, 4H), 6.50 (d, *J* = 2.0 Hz, 1H), 6.18 (d, *J* = 2.1 Hz, 1H), 4.69 (d, *J* = 24.0 Hz, 1H), 4.30 - 4.16 (m, 2H), 3.98 (dp, *J* = 10.2, 7.1 Hz, 1H), 3.79 (ddq, *J* = 10.2, 8.7, 7.1 Hz, 1H), 3.18 (hept, *J* = 6.9 Hz, 1H), 2.67 (hept, *J* = 6.9 Hz, 1H), 1.31 (t, *J* = 7.0 Hz, 3H), 1.23 - 1.12 (m, 6H), 1.13 - 1.03 (m, 9H). ^{13}C NMR (126 MHz, Chloroform-*d*) δ 140.79, 140.60, 134.84, 134.64, 131.54, 131.52, 129.78, 129.74, 121.94, 121.82, 115.00, 110.26, 63.70, 57.03, 55.81, 33.77, 26.92, 24.22, 24.13, 23.05, 22.59, 16.47, 16.25. IR (thin film) 3422br, 1652vs, 1219s, 1053s, 771s cm^{-1} HRMS (ESI-TOF) *m/z* found 520.1243 ($[M+Na]^+$); calcd. 520.1228 for $C_{23}H_{33}NO_4PBrNa$. HPLC: DAICEL CHIRALPAK AD-H (Hexane/*i*PrOH = 95:5, 0.7 mL/min flow rate, 245 nm), 10.0 min for the minor peak and 29.7 min for the major peak.

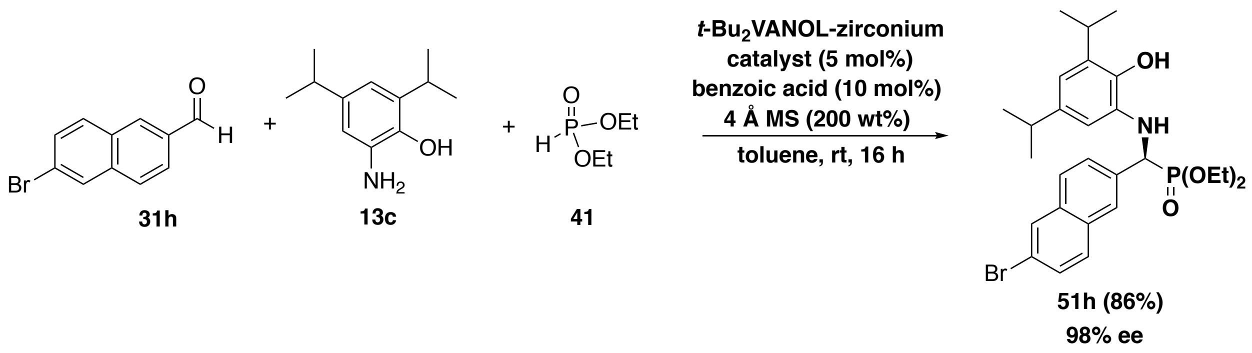

Diethyl (*S*)-((6-bromonaphthalen-2-yl)((2-hydroxy-3,5-diisopropylphenyl) amino)methyl)phosphonate **51h**: Reaction of 6-bromo-2-naphthaldehyde **31h** (23.5 mg, 0.10 mmol, 1.00 equiv) according to general procedure E afforded **51h** (47.1 mg, 0.086 mmol) in 86% yield and 98% *ee* as a yellow oil. R_f = 0.30 (hexane/EtOAc 7:3); Specific Rotation: $[\alpha]_D^{20}$ = -54.9 (c 1.00, $CHCl_3$); Spectral data for **51h**: ^1H NMR (500 MHz, Chloroform-*d*) δ 7.73 (d, *J* = 17.0 Hz, 2H), 7.56 (d, *J* = 8.5 Hz, 1H), 7.44 (d, *J* = 8.5 Hz, 1H), 7.02 (d, *J* = 8.8 Hz, 1H), 6.65 (d, *J* = 8.7 Hz, 1H), 6.41 (s, 1H), 6.34 (s, 1H), 4.96 (d, *J* = 24.5 Hz, 1H), 4.41 - 4.25 (m, 2H), 3.97 - 3.85 (m, 1H), 3.72 - 3.57 (m, 1H), 3.21 (hept, *J* = 7.0 Hz, 1H), 2.66 (hept, *J* = 7.3 Hz, 1H), 1.35 (t, *J* = 7.2 Hz, 3H), 1.16 (d, *J* = 6.8 Hz, 3H), 1.15 - 1.01 (m, 9H), 0.87 (d, *J* = 6.9 Hz, 3H). ^{13}C NMR (126 MHz, Chloroform-*d*) δ 140.54, 134.38, 133.76, 131.44, 129.44, 129.11, 129.04, 127.33, 127.17, 127.12, 127.01, 119.84, 114.83, 109.81, 63.79 (dd, *J* = 110.9, 7.0 Hz), 57.25, 56.03, 33.76, 26.58, 24.33, 24.14, 23.33, 22.49, 16.50 (d, *J* = 5.9 Hz), 16.17 (d, *J* = 5.6 Hz) (two sp^2 carbon are not located). IR (thin film) 3408br, 2960s, 1653vs, 1507s, 1197vs,1053vs, 975s cm^{-1}; HRMS (ESI-TOF) *m/z* found 570.1372 ($[M+Na]^+$); calcd. 570.1358 for $C_{237}H_{35}NO_4PBrNa$. HPLC: DAICEL CHIRALPAK AD-H (Hexane/*i*PrOH = 95:5, 0.7 mL/min flow rate, 245 nm), 17.6 min for the minor peak and 26.3 min for the major peak.

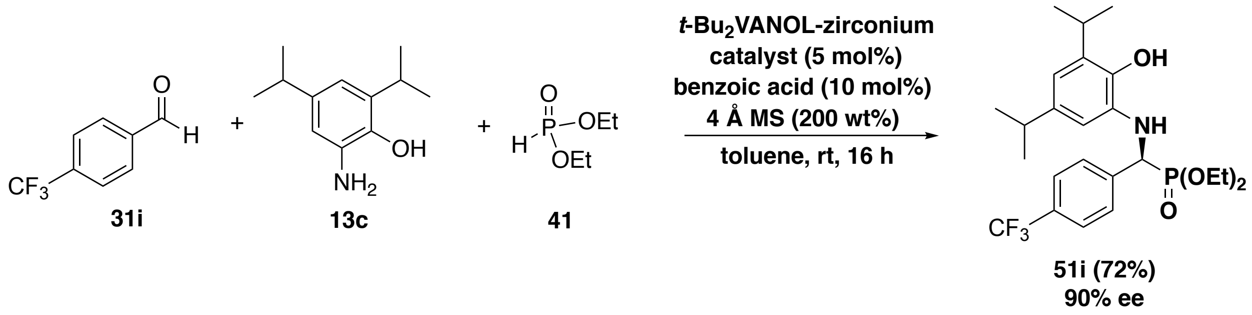

Diethyl (*S*)-(((2-hydroxy-3,5-diisopropylphenyl)amino)(4-(trifluoromethyl) phenyl)methyl) phosphonate **51i**: Reaction of 4-trifluoromethylbenzaldehyde **31i** (11.2 μL, 0.10 mmol, 1.00 equiv) according to general procedure E afforded **51i** (35.1 mg, 0.072 mmol) in 72% yield and 90% *ee* as a yellow oil. R_f = 0.30 (hexane/EtOAc 7:3); Specific Rotation: $[\alpha]_D^{20}$= -12.3 (c 1.00, $CHCl_3$); Spectral data for **51i**: ^1H NMR (500 MHz, Chloroform-*d*) δ 7.56 (d, *J* = 8.1 Hz, 2H), 7.46 (d, *J* = 8.0 Hz, 2H), 6.51 (s, 1H), 6.16 (s, 1H), 4.74 (d, *J* = 23.8 Hz, 1H), 4.28 - 4.10 (m, 2H), 4.10 - 3.94 (m, 1H), 3.94 - 3.81 (m, 1H), 3.12 (hept, *J* = 7.1 Hz, 1H), 2.64 (hept, *J* = 6.8 Hz, 1H), 1.31 (t, *J* = 7.1 Hz, 3H), 1.21 (d, *J* = 6.8 Hz, 3H), 1.17 (t, *J* = 7.0 Hz, 3H), 1.14 (d, *J* = 6.8 Hz, 3H), 1.06 (d, *J* = 7.0 Hz, 3H), 1.03 (d, *J* = 6.9 Hz, 3H). ^{13}C NMR (126 MHz, Chloroform-*d*) δ 141.04, 140.80, 128.46, 127.69, 125.89, 125.40, 122.37, 115.54, 114.73, 111.03, 76.75, 63.61 (dd, *J* = 52.8, 7.0 Hz), 57.70, 56.49, 33.70, 29.70, 27.17, 24.13, 23.98, 22.56, 16.43 (d, *J* = 5.6 Hz), 16.21 (d, *J* = 5.8 Hz); IR (thin film) 3422br, 2095s, 1653vs, 1457 m cm^{-1}; HRMS (ESI-TOF) *m/z* found 488.2180 ($[M+H]^+$); calcd. 488.2178 for $C_{24}H_{34}NO_4F_3P$. HPLC: DAICEL CHIRALPAK AD-H (Hexane/*i*PrOH = 95:5, 0.7 mL/min flow rate, 245 nm), 10.8 min for the minor peak and 28.8 min for the major peak.

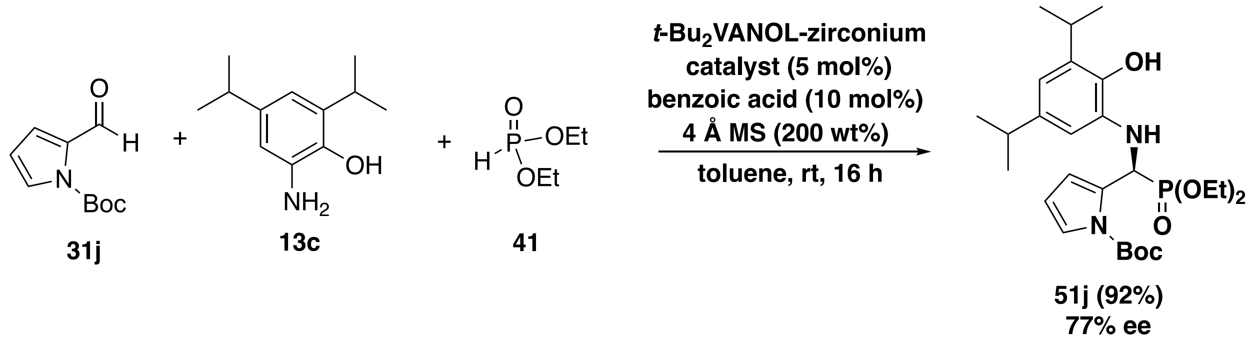

tert-Butyl (*S*)-2-((diethoxyphosphoryl)((2-hydroxy-3,5-diisopropylphenyl)amino) methyl)-1*H*-pyrrole-1-carboxylate **51j**: Reaction of *tert*-butyl 2-formyl-1*H*-pyrrole-1-carboxylate **31j** (18.1 mg, 0.1 mmol, 1.00 equiv) according to general procedure E fforded **51j** (46.7 mg, 0.092 mmol) in 92% yield and 77% *ee* as a yellow oil. R_f = 0.30 (hexane/EtOAc 7:3); Specific Rotation: $[\alpha]_D^{20}$= -1.1 (c 1.00, $CHCl_3$); Spectral data for **51j**: ^1H NMR (500 MHz, Chloroform-*d*) δ 7.25 (s, 1H), 6.56 - 6.51 (m, 2H), 6.41 (s, 1H), 6.10 - 6.06 (m, 1H), 4.80 (br, 1H, NH), 4.18 (dt, *J* = 14.1, 6.9 Hz, 2H), 3.98 (dt, *J* = 9.9, 6.9 Hz, 1H), 3.76 (dt, *J* = 14.5, 7.4 Hz, 1H), 3.26 (hept, *J* = 7.0 Hz, 1H), 2.71 (hept, *J* = 7.0 Hz, 1H), 1.60 (s, 9H), 1.31 - 1.24 (m, 3H), 1.23 - 1.17 (m, 6H), 1.16 - 1.10 (m, 9H). ^{13}C NMR (126 MHz, Chloroform-*d*) δ 149.51, 141.89, 140.41, 134.37, 130.18, 122.35, 115.80, 114.13, 111.71, 110.66, 84.32, 63.29, 63.23, 63.17, 33.75, 27.90, 27.07, 24.26, 24.21, 22.93, 22.79, 16.43 (d, *J* = 5.9 Hz), 16.28 (d, *J* = 5.7 Hz) (one sp^2 carbon is not located). IR (thin film) 3420br, 2091s, 1645vs, 1320vs, 1023s, 973s cm^{-1}. HRMS (ESI-TOF) *m/z* found 509.2802 ($[M+H]^+$); calcd. 509.2780 for $C_{26}H_{42}N_2O_6P$. HPLC: DAICEL CHIRALPAK AD-H (Hexane/*i*PrOH = 95:5, 0.7 mL/min flow rate, 245 nm), 12.4 min for the minor peak and 24.1 min for the major peak.

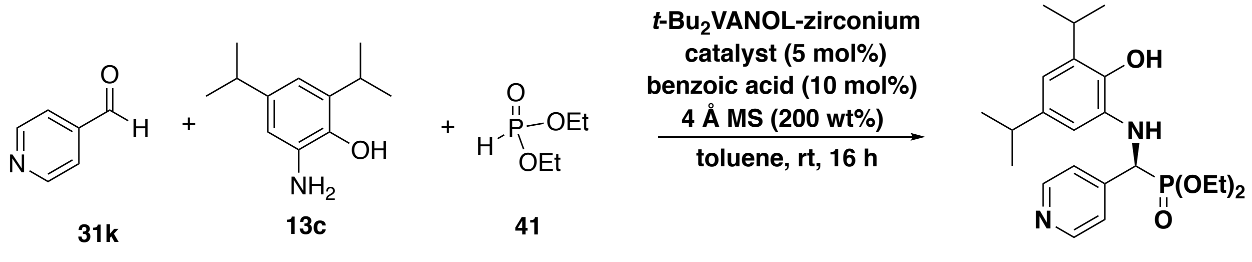

31k 13c 41

51k (35%)
17% ee

Diethyl (*S*)-(((2-hydroxy-3,5-diisopropylphenyl)amino)(pyridin-4-yl)methyl) phosphonate **51k**: Reaction of isonicotinaldehyde **31k** (9.90 μL, 0.10 mmol, 1.00 equiv) according to general procedure E afforded **51k** (14.7 mg, 0.035 mmol) in 35% yield and 17% *ee* as a yellow oil; R_f = 0.30 (hexane/EtOAc 7:3); Specific Rotation: $[\alpha]_D^{20}$= -7.9 (c 1.00, $CHCl_3$); Spectral data for **51k**: ^{1}H NMR (500 MHz, Chloroform-*d*) δ 8.70 (s, 1H), 8.46 (dt, *J* = 3.6, 1.8 Hz, 1H), 7.86 (dq, *J* = 8.0, 2.1 Hz, 2H), 7.13 (dd, *J* = 8.0, 4.9 Hz, 1H), 6.49 (d, *J* = 2.1 Hz, 1H), 6.18 (d, *J* = 2.0 Hz, 1H), 4.80 (d, *J* = 24.1 Hz, 1H), 4.36 - 4.13 (m, 2H), 4.08 - 3.96 (m, 1H), 3.88 (m, *J* = 10.1, 8.4, 7.0 Hz, 1H), 3.20 (p, *J* = 6.9 Hz, 1H), 2.65 (p, *J* = 6.9 Hz, 1H), 1.31 (t, *J* = 7.0 Hz, 3H), 1.20 (d, *J* = 6.9 Hz, 3H), 1.17 (t, *J* = 7.1 Hz, 3H), 1.13 (d, *J* = 6.9 Hz, 3H), 1.08 (d, *J* = 6.9 Hz, 3H), 1.05 (d, *J* = 6.9 Hz, 3H). ^{13}C NMR (126 MHz, Chloroform-*d*) δ 150.43, 148.88, 123.26, 122.94, 122.45, 122.10, 121.39, 120.98, 114.97,109.93, 63.73, 57.17, 55.98, 30.00, 27.20, 24.43, 24.18, 22.78, 22.59, 16.32, 16.19 (one sp^2 carbon is not located). IR (thin film) 3423br, 2100s, 1646vs, 1208vs,1041vs cm^{-1}; HRMS (ESI-TOF) *m/z* found 421.2278 ([M+H]$^+$); calcd. 421.2256 for $C_{22}H_{34}N_2O_4P$. HPLC: DAICEL CHIRALPAK AS-H (Hexane/*i*PrOH = 5:95, 1 mL/min flow rate, 245 nm), 11.4 min for the minor peak and 18.9 min for the major peak.

6. The Asymmetric Kabachnik-Fields Reactions with Aliphatic Aldehydes 66a-r

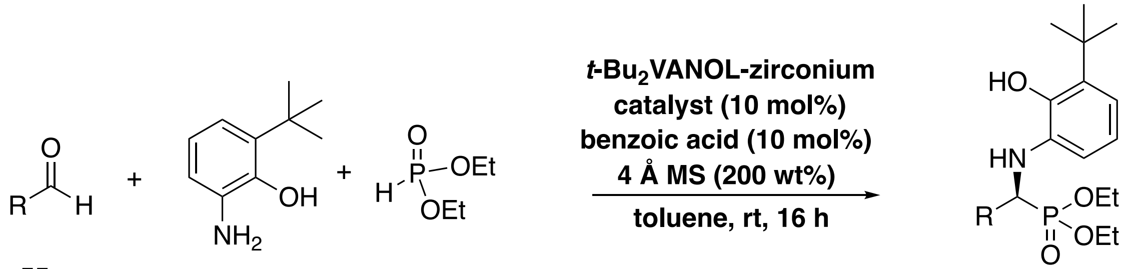

55a-r **13l** **41** **66a-r**

General procedure F for asymmetric Kabachnik-Fields reactions of aliphatic aldehydes: To a 10-mL flame-dried home-made Schlenk flask, prepared from a single necked 25 mL pear-shaped flask that had its 14/20 glass joint replaced with a high vacuum threaded Teflon valve, flushed with nitrogen was added 2-hydroxy-3-*tert*-propylaniline **13l** (16.5 mg, 0.1 mmol, 1.00 equiv), 4 Å molecular sieves (33.0 mg) and benzoic acid (1.20 mg, 0.01 mmol, 0.10 equiv). Under a nitrogen flow, the zirconium catalyst in toluene (0.40 mL, 10 mol%, 0.25 M, prepared from general procedure D), aldehyde (0.10 mmol, 1.00 equiv) and diethyl phosphite (14.0 μL, 0.10 mmol, 1.00 equiv) were added to the mixture by syringe. The flask was sealed, and then placed into a liquid nitrogen bath. After the solvent was frozen, the Teflon valve was opened to vacuum and the atmosphere was pumped off for 5 minutes. The flask was sealed again and removed from the liquid nitrogen bath. Gas bubbles could be seen to evolve from the solution during thawing the solvent at the room temperature. When all the solvent melted, the flask was placed back into the liquid nitrogen bath and this Freeze-Pump-Thaw degassing process was repeated for another 2 times. The reaction was then stirred at room temperature for 16-72 hours and diluted with hexane (5.00 mL). The mixture was concentrated under rotary evaporation and purified by flash

chromatography (silica gel, 10 mm x 100 mm, hexane/EtOAc, 7:3) to yield corresponding α-amino phosphites. Each reaction was run twice, each with a different enantiomer of the catalyst. In this way we could determine the retention time for each enantiomer of the product and these are given in each experimental procedure. In each case, base-line separation of the enantiomers was realized.

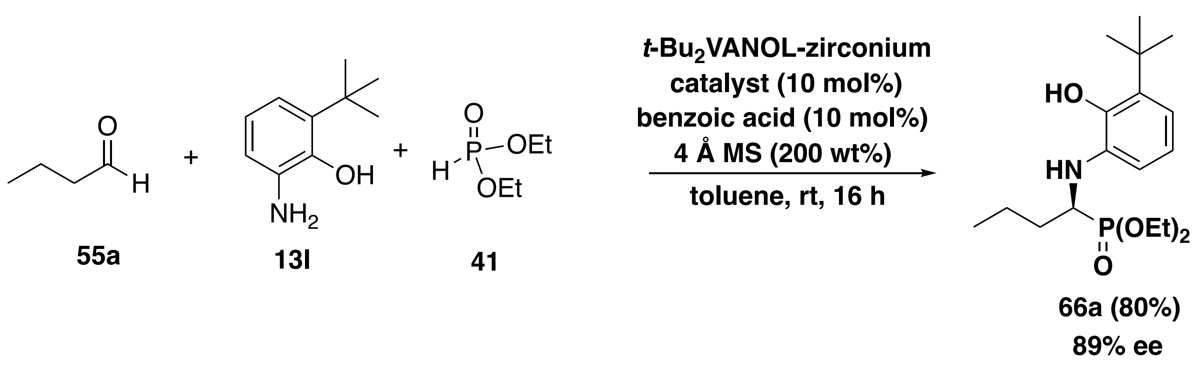

Diethyl (*S*)-(1-((3-(*tert*-butyl)-2-hydroxyphenyl)amino)butyl)phosphonate **66a**: Reaction of butyraldehyde **55a** (9.00 μL, 0.10 mmol, 1.00 equiv) according to general procedure F afforded **66a** (28.6 mg, 0.080 mmol) after 16 hours in 80% yield and 89% *ee* as a yellow oil; R_f = 0.30 (hexane/EtOAc 7:3); Spectral data for **66a**: ^{1}H NMR (500 MHz, Chloroform-*d*) δ 7.24 - 7.03 (m, 1H, NH), 6.89 (d, *J* = 7.8 Hz, 1H), 6.80 (d, *J* = 7.7 Hz, 1H), 6.74 (t, *J* = 7.8 Hz, 1H), 4.21 - 3.95 (m, 4H), 3.51-3.45 (m, 1H), 1.89-1.82 (m, 1H), 1.736-1.71 (m, 1H), 1.65 - 1.55 (m, 1H), 1.53-1.48 (m, 1H), 1.38 (s, 9H), 1.32 (t, *J* = 7.0 Hz, 3H), 1.21 (t, *J* = 7.1 Hz, 3H), 0.92 (t, *J* = 7.4 Hz, 3H). ^{13}C NMR (126 MHz, Chloroform-*d*) δ 147.17, 137.30, 135.46, 120.09, 119.57, 117.55, 62.37 (dd, *J* = 36.2, 7.5 Hz), 54.65, 53.42, 34.67, 32.78, 29.71 (3C), 19.39 16.46, 16.40, 13.94; IR (thin film) 3091br, 2399s, 1708s, 1213s, 748vs cm^{-}1; HRMS (ESI-TOF) *m/z* found 358.2147 ([M+H]$^+$); calcd. 358.2147 for $C_{18}H_{33}NO_4P$; HPLC: DAICEL CHIRALPAK AS-H

(Hexane/*i*PrOH = 99:1, 1 mL/min flow rate, 245 nm), 8.4 min for the minor peak and 11.0 min for the major peak.

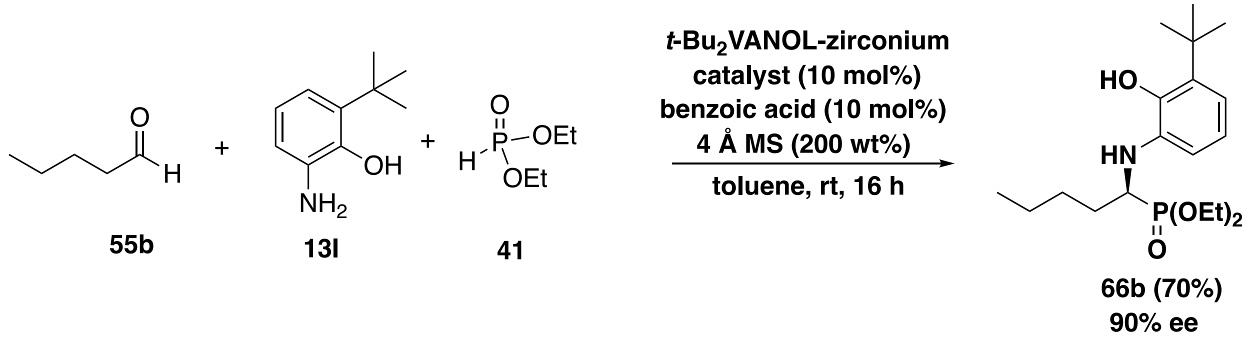

Diethyl (*S*)-(1-((3-(*tert*-butyl)-2-hydroxyphenyl)amino)pentyl)phosphonate **66b**: Reaction of pentanal **55b** (10.6 μL, 0.10 mmol, 1.00 equiv) according to general procedure F afforded **66b** (26.0 mg, 0.07 mmol) after 16 hours in 70% yield and 90% *ee* as a yellow oil. R_f = 0.30 (hexane/EtOAc 7:3); Spectral data for **66b**: ^{1}H NMR (500 MHz, Chloroform-*d*) δ 7.09 (br, 1H, NH), 6.93 (dd, *J* = 7.9, 1.6 Hz, 1H), 6.85 (dd, *J* = 7.7, 1.5 Hz, 1H), 6.74 (t, *J* = 7.8 Hz, 1H), 4.19 - 3.96 (m, 4H), 3.46 (ddd, *J* = 13.6, 8.0, 5.4 Hz, 1H), 1.98 - 1.84 (m, 1H), 1.81 - 1.68 (m, 1H), 1.59 - 1.50 (m, 1H), 1.40 (s, 9H), 1.33-1.30 (m, 3H), 1.30 (t, *J* = 7.1 Hz, 3H), 1.22 (t, *J* = 7.1 Hz, 3H), 0.88 (t, *J* = 7.3 Hz, 3H). ^{13}C NMR (126 MHz, Chloroform-*d*) δ 147.68, 137.44, 134.80, 120.93, 119.61, 118.61, 62.54 (dd, *J* = 18.6, 7.3 Hz), 55.28, 54.05, 34.67, 30.14, 29.69, 28.24, 28.17, 22.54, 16.44, 16.40, 16.34, 13.85. IR (thin film) 3373br, 3306br, 2924vs, 1698vs, 1442s, 1206s,1166s, 1020vs, 754s cm^-1; HRMS (ESI-TOF) *m/z* found 372.2294 ($[M+H]^+$); calcd. 372.2304 for $C_{19}H_{35}NO_4P$; HPLC: DAICEL CHIRALPAK AD-H (Hexane/*i*PrOH = 99:1, 1 mL/min flow rate, 245 nm), 8.5 min for the minor peak and 9.9 min for the major peak.

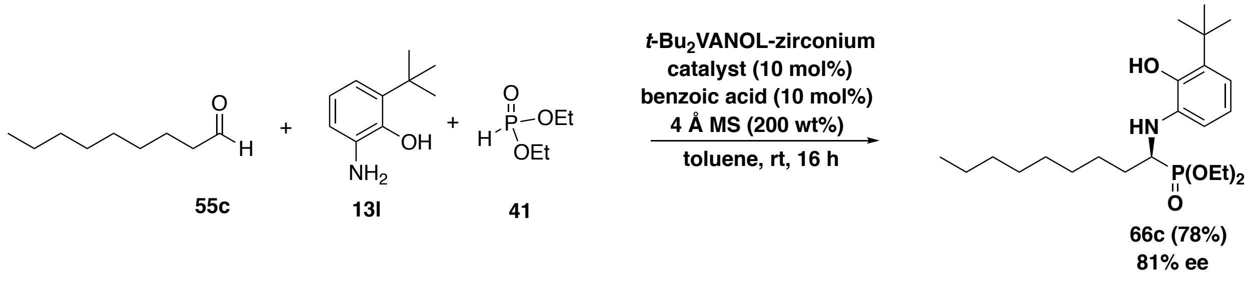

Diethyl (*S*)-(1-((3-(*tert*-butyl)-2-hydroxyphenyl)amino)nonyl)phosphonate **66c**: Reaction of nonanal **55c** (17.2 μL, 0.10 mmol, 1.00 equiv) according to general procedure F afforded **66c** (33.0 mg, 0.078 mmol) after 16 hours in 78% yield and 81% *ee* as a yellow oil. R_f = 0.30 (hexane/EtOAc 7:3); Spectral data for **66c**: ^{1}H NMR (500 MHz, Chloroform-*d*) δ 7.17 - 7.06 (br, 1H, NH), 6.95 (dd, *J* = 7.9, 1.6 Hz, 1H), 6.86 (dd, *J* = 7.8, 1.6 Hz, 1H), 6.76 (t, *J* = 7.8 Hz, 1H), 4.22 - 3.98 (m, 4H), 3.50 - 3.44 (m, 1H), 1.97 - 1.83 (m, 1H), 1.81 - 1.71 (m, 1H), 1.60 - 1.52 (m, 1H), 1.42 (s, 9H), 1.31 (t, *J* = 7.0 Hz, 3H), 1.30 - 1.17 (m, 14H), 0.88 (t, *J* = 7.0 Hz, 3H). ^{13}C NMR (126 MHz, Chloroform-*d*) δ 147.74, 121.06, 119.65, 118.74, 62.59 (dd, *J* = 20.2, 7.3 Hz), 55.33, 54.11, 34.70, 31.82, 30.40, 29.70 (3C), 29.44, 29.31, 29.21, 26.09, 22.65, 16.41 (2C), 14.11 (two sp^2 are not located). IR (thin film) 3370br, 2924vs, 2096vs, 1713s, 1205s, 1021vs, 754s cm^{-}1; HRMS (ESI-TOF) *m/z* found 428.2935 ([M+H]$^+$); calcd. 428.2930 for $C_{23}H_{43}NO_4P$; HPLC: DAICEL CHIRALPAK IA-H (Hexane/*i*PrOH = 99:1, 1 mL/min flow rate, 245 nm), 16.3 min for the minor peak and 18.8 min for the major peak.

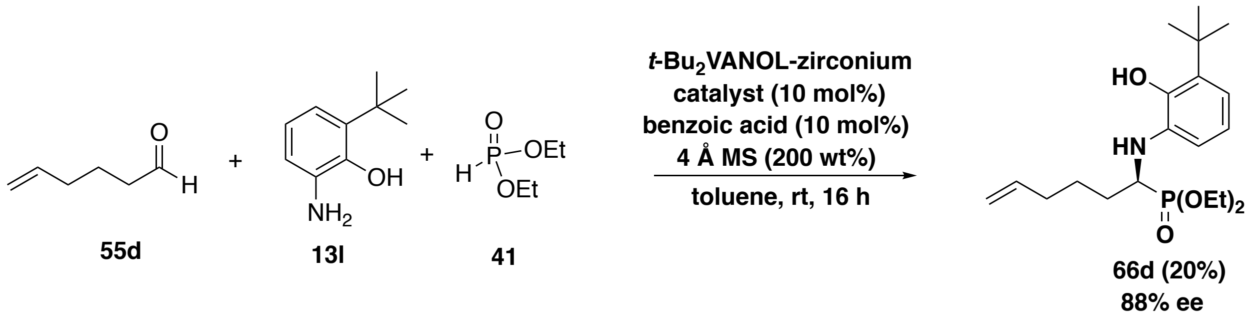

Diethyl (*S*)-(1-((3-(*tert*-butyl)-2-hydroxyphenyl)amino)hex-5-en-1-yl)phosphonate **66d**: Reaction of hex-5-enal **55d** (12.0 μL, 0.10 mmol, 1.00 equiv) according to general procedure F afforded **66d** (7.6 mg, 0.020 mmol) after 16 hours in 20% yield and 88% *ee* as a yellow oil. R_f = 0.30 (hexane/EtOAc 7:3); Spectral data for **66d**: ^{1}H NMR (500 MHz, Chloroform-*d*) δ 6.91 (d, *J* = 7.8 Hz, 1H), 6.84 - 6.78 (m, 1H), 6.74 (t, *J* = 7.9 Hz, 1H), 5.76 (ddt, *J* = 17.0, 10.2, 6.7 Hz, 1H), 5.08 - 4.83 (m, 2H), 4.19 - 3.97 (m, 4H), 3.40 (m, 1H), 2.13 - 2.01 (m, 2H), 1.95 - 1.84 (m, 1H), 1.80 - 1.61 (m, 2H), 1.61 - 1.51 (m, 1H), 1.40 (s, 9H), 1.32 (q, *J* = 7.4 Hz, 3H), 1.24 (t, *J* = 7.0 Hz, 3H). ^{13}C NMR (126 MHz, Chloroform-*d*) δ 147.72, 138.06, 136.99, 135.36, 120.64, 119.55, 118.60, 115.03, 62.48, 55.14, 53.91, 34.61, 33.48, 30.10, 29.70 (3C), 25.37, 16.37 (2C). IR (thin film) 3372br, 3308br, 2926s, 1586s, 1458s, 1213vs, 1024vs, 747vs cm^{-}1; HRMS (ESI-TOF) *m/z* found 384.2307 ($[M+H]^+$); calcd. 384.2304 for $C_{20}H_{35}NO_4P$; HPLC: DAICEL CHIRALPAK IA-H (Hexane/*i*PrOH = 95: 5, 1 mL/min flow rate, 245 nm), 8.8 min for the minor peak and 9.8 min for the major peak.

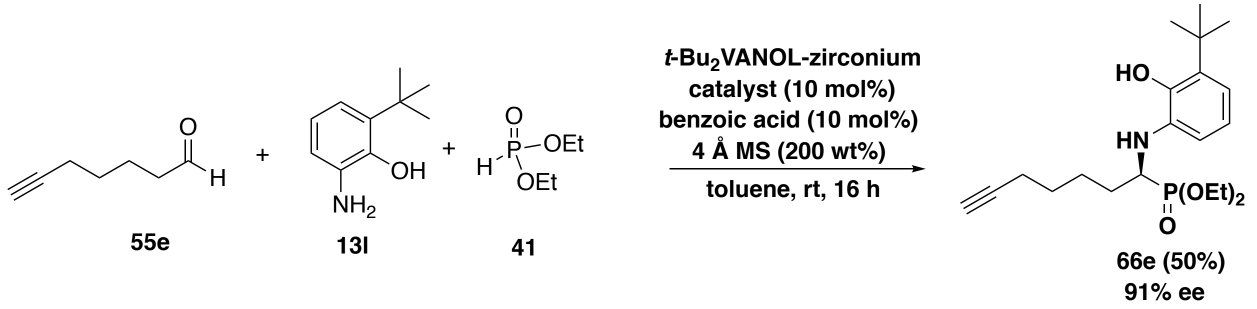

Diethyl (*S*)-(1-((3-(*tert*-butyl)-2-hydroxyphenyl)amino)hept-6-yn-1-yl)phosphonate **66e**: Reaction of hept-6-ynal **55e** (11.0 μL, 0.10 mmol, 1.00 equiv) according to general procedure F afforded **66e** (18.9 mg, 0.050 mmol) after 16 hours in 50% yield and 91% *ee* as a yellow oil. R_f = 0.30 (hexane/EtOAc 7:3); Spectral data for **66e**: ^{1}H NMR (500 MHz, Chloroform-*d*) δ 6.98 (dd, *J* = 7.9, 1.6 Hz, 1H), 6.91 (dd, *J* = 7.8, 1.5 Hz, 1H), 6.77 (t, *J* = 7.9 Hz, 1H), 4.26 - 3.96 (m, 4H), 3.57 - 3.48 (m, 1H), 2.17 (td, *J* = 7.0, 2.7 Hz, 2H), 1.93 (t, *J* = 2.6 Hz, 1H), 1.80 - 1.60 (m, 4H), 1.57 - 1.50 (m, 2H), 1.41 (s, 9H), 1.30 (t, *J* = 7.1 Hz, 3H), 1.24 (t, *J* = 7.1 Hz, 3H). ^{13}C NMR (126 MHz, Chloroform-*d*) δ 147.74, 138.21, 129.65, 121.73, 119.93, 119.06, 84.13, 68.51, 62.75, 55.33, 54.10, 34.75, 29.71, 29.61, 28.17, 25.22, 25.15, 18.14, 16.43, 16.39, 16.34. IR (thin film) 3375br, 3308br, 2923vs, 2399s, 1722s, 1585s, 1443s, 1210s, 1022vs, 751s cm$^-$1; HRMS (ESI-TOF) *m/z* found 396.2303 ([M+H]$^+$); calcd. 396.2304 for $C_{21}H_{35}NO_4P$; HPLC: DAICEL CHIRALPAK IA-H (Hexane/*i*PrOH = 98:2, 1 mL/min flow rate, 245 nm), 16.2 min for the minor peak and 18.3 min for the major peak.

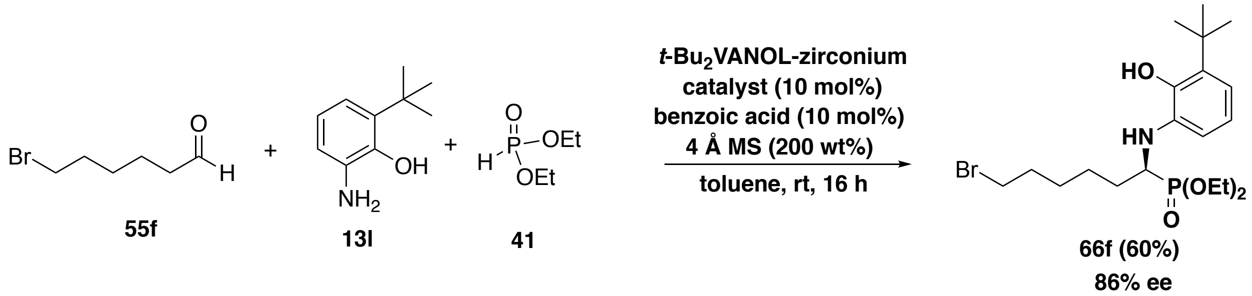

Diethyl (*S*)-(5-bromo-1-((3-(*tert*-butyl)-2-hydroxyphenyl)amino)pentyl) phosphonate **66f**: Reaction of 6-bromohexanal **55f** (17.8 mg, 0.10 mmol, 1.00 equiv) according to general procedure F afforded the **66f** (27.8 mg, 0.060 mmol) after 16 hours in 60% yield and 86% *ee* as a yellow oil. R_f = 0.30 (hexane/EtOAc 7:3); Spectral data for **66f**: ^{1}H NMR (500 MHz, Chloroform-*d*) δ 6.96 (d, *J* = 7.9 Hz, 1H), 6.86 (t, *J* = 10.2 Hz, 1H), 6.82 - 6.74 (m, 1H), 4.21 - 3.98 (m, 4H), 3.52 (dt, *J* = 14.2, 6.7 Hz, 1H), 3.42 - 3.31 (m, 2H), 1.82 (td, *J* = 12.9, 11.6, 5.8 Hz, 3H), 1.63 - 1.42 (m, 5H), 1.38 (s, 9H), 1.30 (t, *J* = 7.0 Hz, 3H), 1.23 (t, *J* = 7.1 Hz, 3H). ^{13}C NMR (126 MHz, Chloroform-*d*) δ 147.52, 121.48, 119.92, 118.64, 62.76 (dd, *J* = 27.5, 7.1 Hz), 55.16, 53.93, 34.73, 33.68, 32.36, 30.00, 29.71, 27.89, 25.25, 25.18, 16.44, 16.39, 16.35 (two sp^2 carbons are not located). IR (thin film) 3373br, 2953s, 1735vs, 1439s, 1203vs, 1020vs, 753s cm$^-$1; HRMS (ESI-TOF) *m/z* found 464.1573 ($[M+H]^+$); calcd. 464.1565 for $C_{20}H_{36}NO_4PBr$; HPLC: DAICEL CHIRALPAK AD-H (Hexane/*i*PrOH = 98:2, 1 mL/min flow rate, 245 nm), 17.9 min for the minor peak and 27.8 min for the major peak.

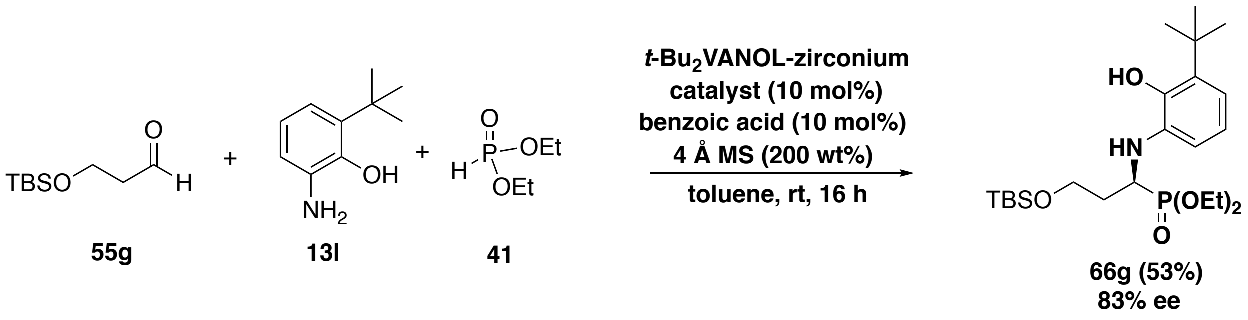

Diethyl (*S*)-(1-((3-(*tert*-butyl)-2-hydroxyphenyl)amino)-3-((*tert*-butyldimethyl silyl)oxy) propyl)phosphonate **66g**: Reaction of 3-((*tert*-butyldimethylsilyl)oxy)propane **55g** (18.8 mg, 0.10 mmol, 1.00 equiv) according to general procedure F afforded **66g** (25.1 mg, 0.053 mmol) after 24 hours in 53% yield and 83% *ee* as a yellow oil. R_f = 0.30 (hexane/EtOAc 7:3); Spectral data for **66g**: ^{1}H NMR (500 MHz, Chloroform-*d*) δ 7.00 - 6.89 (m, 2H), 6.73 (t, *J* = 7.8 Hz, 1H), 4.11 - 3.89 (m, 5H), 3.89 - 3.74 (m, 2H), 2.24 (m, 1H), 1.91 - 1.80 (m, 1H), 1.39 (s, 9H), 1.21 (t, *J* = 7.1 Hz, 3H), 1.18 (t, *J* = 7.1 Hz, 3H), 0.90 (s, 9H), 0.09 (s, 3H), 0.07 (s, 3H). ^{13}C NMR (126 MHz, Chloroform-*d*) δ 148.09, 128.89, 127.41, 126.37, 120.24, 119.43, 62.43, 59.67, 59.58, 34.74, 32.20, 29.63 (3C), 25.91 (3C), 18.33, 16.24 (2C), -5.36, -5.51. IR (thin film) 3374br, 3307br, 2926vs, 1701vs, 1442s, 1208s, 1021vs, 753vs cm^{-}1; HRMS (ESI-TOF) *m/z* found 474.2813 ([M+H]$^+$); calcd. 474.2805 for $C_{23}H_{45}NO_5SiP$; HPLC: DAICEL CHIRALPAK IA-H (Hexane/*i*PrOH = 99:1, 1 mL/min flow rate, 245 nm), 17.4 min for the minor peak and 19.6 min for the major peak.

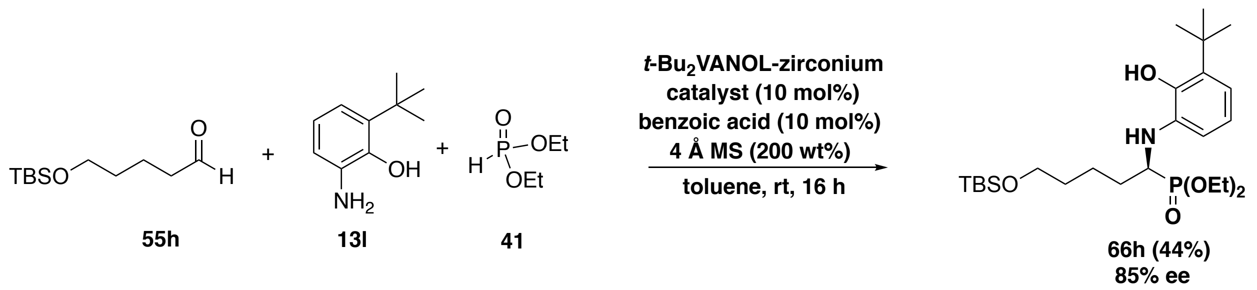

Diethyl (*S*)-(1-((3-(*tert*-butyl)-2-hydroxyphenyl)amino)-5-((*tert* butyldimethyl silyl)oxy) pentyl) phosphonate **66h**: Reaction of 5-((*tert*-butyldimethylsilyl)oxy)pentanal **55h** (21.6 mg, 0.1 mmol, 1.00 equiv) according to general procedure F afforded **66h** (22.0 mg, 0.044 mmol) after 48 hours in 44% yield and 85% *ee* as a yellow oil. R_f = 0.30 (hexane/EtOAc 7:3); Specific Rotation: $[\alpha]_D^{20}$= -7.19 (c 1.05, $CHCl_3$); Spectral data for **66h**: ^{1}H NMR (500 MHz, Chloroform-*d*) δ 7.00 (br, 1H, NH), 6.93 (dd, *J* = 7.9, 1.6 Hz, 1H), 6.84 (dd, *J* = 7.8, 1.5 Hz, 1H), 6.74 (t, *J* = 7.8 Hz, 1H), 4.20 - 3.98 (m, 4H), 3.59 (t, *J* = 6.1 Hz, 2H), 3.50 - 3.38 (m, 1H), 1.99 - 1.83 (m, 1H), 1.83 - 1.68 (m, 1H), 1.67 - 1.56 (m, 1H), 1.56 - 1.48 (m, 3H), 1.41 (s, 9H), 1.31 (t, *J* = 7.1 Hz, 3H), 1.24 (t, *J* = 7.1 Hz, 3H), 0.88 (s, 9H), 0.03 (s, 6H). ^{13}C NMR (126 MHz, Chloroform-*d*) δ 147.77, 137.30, 134.97, 120.90, 119.60, 118.76, 62.79, 62.52 (dd, *J* = 20.2, 7.3 Hz), 55.38, 54.15, 34.66, 32.61, 30.38, 29.70 (3C), 25.96, 22.51, 18.34, 16.40 (2C), -5.29 (2C). IR (thin film) 3373br, 3307br, 2926vs, 1702s, 1441s, 1213s, 1022vs, 752s cm$^-$1; HRMS (ESI-TOF) *m/z* found 502.3120 ($[M+H]^+$); calcd. 502.3118 for $C_{25}H_{49}NO_5SiP$; HPLC: DAICEL CHIRALPAK IA-H (Hexane/*i*PrOH = 99:1, 1 mL/min flow rate, 245 nm), 12.3 min for the minor peak and 13.7 min for the major peak.

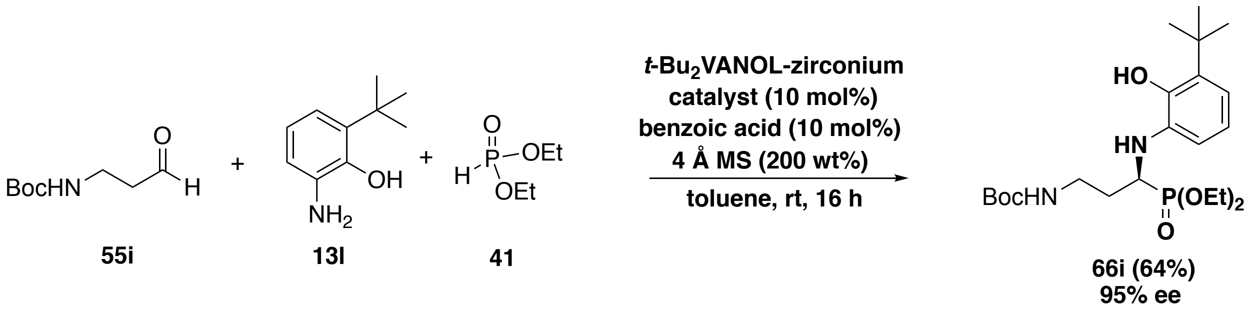

tert-Butyl (*S*)-(3-((3-(*tert*-butyl)-2-hydroxyphenyl)amino)-3-(diethoxyphosphoryl) propyl) carbamate **66i**: Reaction of *tert*-butyl (3-oxopropyl)carbamate **55i** (17.3 mg, 0.1 mmol, 1.00 equiv) according to general procedure F afforded **66i** (29.3 mg, 0.064 mmol) after 16 hours in 64% yield and 95% *ee* as a yellow oil. R_f = 0.30 (hexane/EtOAc 7:3); Spectral data for **66i**: ^{1}H NMR (500 MHz, Chloroform-*d*) δ 6.89 (d, *J* = 7.6 Hz, 1H), 6.80 (d, *J* = 7.7 Hz, 1H), 6.75 (t, *J* = 7.8 Hz, 1H), 4.93 (br, 1H, NH), 4.19 - 3.95 (m, 4H), 3.63 - 3.53 (m, 1H), 3.37 - 3.28 (m, 2H), 2.18 - 2.07 (m, 1H), 1.95 - 1.85 (m, 1H), 1.41 (s, 9H), 1.39 (s, 9H), 1.29 (t, *J* = 7.0 Hz, 3H), 1.20 (t, *J* = 7.1 Hz, 4H). ^{13}C NMR (126 MHz, Chloroform-*d*) δ 156.06, 146.85, 137.99, 135.66, 127.43, 120.09, 117.56, 62.72 (dd, *J* = 35.1, 7.0 Hz), 52.82, 51.59, 37.42, 34.69, 30.93 (3C), 29.75 (3C), 28.37, 16.42, 16.37, 16.32. IR (thin film) 3373br, 2925vs, 2096vs, 1713s, 1585s, 1442s, 1210vs, 1022vs, 751vs cm^{-}1; HRMS (ESI-TOF) *m/z* found 459.2634 ($[M+H]^+$); calcd. 459.2624 for $C_{22}H_{40}N_2O_6P$; HPLC: DAICEL CHIRALPAK AS-H (Hexane/*i*PrOH = 98:2, 1 mL/min flow rate, 245 nm), 11.4 min for the minor peak and 14.8 min for the major peak.

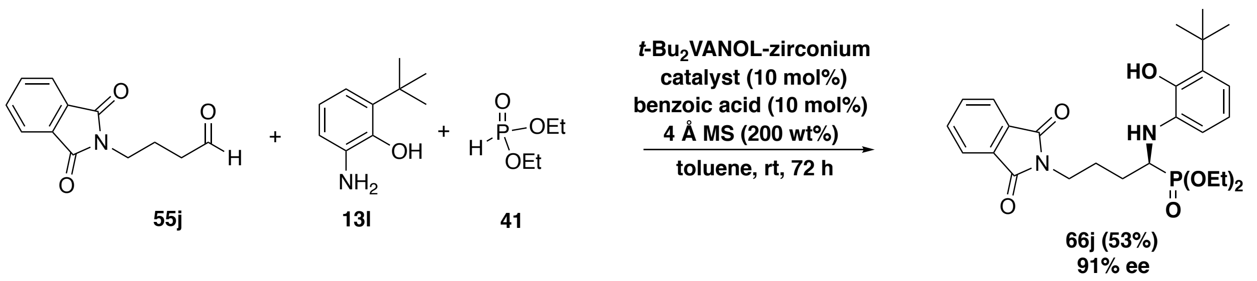

Diethyl (*S*)-(1-((3-(*tert*-butyl)-2-hydroxyphenyl)amino)-4-(1,3-dioxoisoindolin-2-yl)butyl) phosphonate **66j**: Reaction of 4-(1,3-dioxoisoindolin-2-yl)butanal **55j** (21.9 mg, 0.1 mmol, 1.00 equiv) according to general procedure F afforded **66j** (26.6 mg, 0.053 mmol) after 72 hours in 53% yield and 91% *ee* as a yellow oil. R_f = 0.30 (hexane/EtOAc 7:3); Spectral data for **66j**: ^{1}H NMR (500 MHz, Chloroform-*d*) δ 7.82 (dt, *J* = 7.7, 3.8 Hz, 2H), 7.80 - 7.66 (m, 2H), 6.85 (d, *J* = 7.8 Hz, 1H), 6.82 - 6.66 (m, 3H), 4.19 - 4.01 (m, 4H), 3.70 (t, *J* = 6.6 Hz, 2H), 3.56 - 3.44 (m, 1H), 2.02 - 1.86 (m, 3H), 1.85 - 1.72 (m, 1H), 1.36 (d, *J* = 7.1 Hz, 9H), 1.28 (d, *J* = 7.1 Hz, 3H), 1.20 (d, *J* = 7.1 Hz, 3H). ^{13}C NMR (126 MHz, Chloroform-*d*) δ 168.33, 147.18, 137.22, 135.52, 135.46, 133.93, 132.03, 123.21, 120.26, 119.78, 117.96, 62.56 (dd, *J* = 18.2, 7.3 Hz), 54.53, 53.30, 37.56, 34.59, 29.72 (3C), 27.78, 25.19, 25.12, 16.38. IR (thin film) 3374br, 2926vs, 2095vs, 1713s, 1442s, 1210vs, 1022vs, 752s cm^{-}1; HRMS (ESI-TOF) *m/z* found 503.2325 ($[M+H]^+$); calcd. 503.2311 for $C_{26}H_{36}N_2O_6P$; HPLC: DAICEL CHIRALPAK AS-H (Hexane/*i*PrOH = 5:95, 1 mL/min flow rate, 245 nm), 11.4 min for the minor peak and 18.9 min for the major peak.

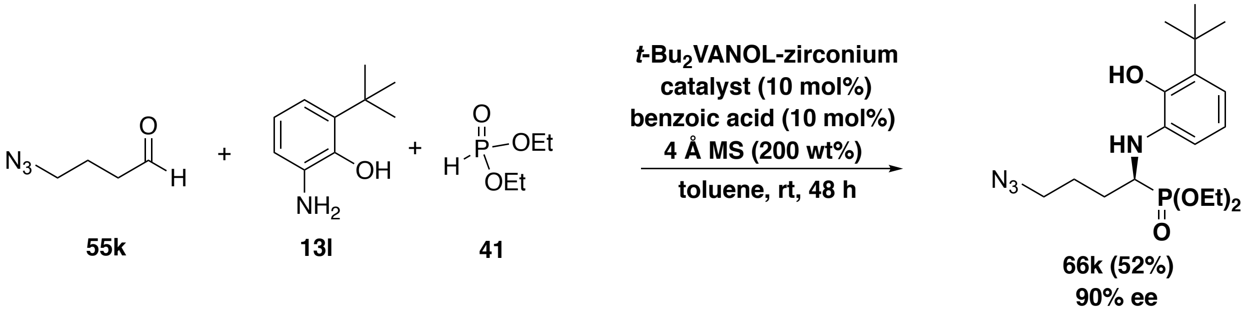

Diethyl (*S*)-(4-azido-1-((3-(*tert*-butyl)-2-hydroxyphenyl)amino)butyl)phosphonate **66k**: Reaction of 4-azidobutanal **55k** (11.3 mg, 0.10 mmol, 1.00 equiv) according to general procedure F afforded **66k** (19.9 mg, 0.052mmol) after 48 hours in 52% yield and 90% *ee* as a yellow oil. R_f = 0.30 (hexane/EtOAc 7:3); Spectral data for **66k**: ^{1}H NMR (500 MHz, Chloroform-*d*) δ 6.94 (d, *J* = 7.9 Hz, 1H), 6.84 (d, *J* = 7.8 Hz, 1H), 6.77 (q, *J* = 7.7 Hz, 1H), 4.19 – 3.99 (m, 4H), 3.58 – 3.49 (m, 1H), 3.29 (t, *J* = 6.3 Hz, 2H), 2.06 – 1.94 (m, 1H), 1.90 – 1.75 (m, 3H), 1.40 (s, 9H), 1.34 – 1.28 (m, 3H), 1.24 – 1.19 (m, 3H). ^{13}C NMR (126 MHz, Chloroform-*d*) δ 147.22, 137.80, 134.64, 120.91, 119.97, 117.99, 62.81 (dd, *J* = 25.1, 7.1 Hz), 54.64, 53.40, 51.16, 34.68, 27.69 (3C), 25.65, 16.40 (two sp3 carbons are not located). IR (thin film) 3370br, 2927vs, 2095vs, 1712vs, 1212vs, 1021vs, 751vs cm^{-}1; HRMS (ESI-TOF) *m/z* found 399.2166 ([M+H]$^+$); calcd. 399.2161 for $C_{18}H_{32}N_4O_4P$; HPLC: DAICEL CHIRALPA IA-H (Hexane/*i*PrOH = 98:2, 1 mL/min flow rate, 245 nm), 17.9 min for the minor peak and 26.2 min for the major peak.

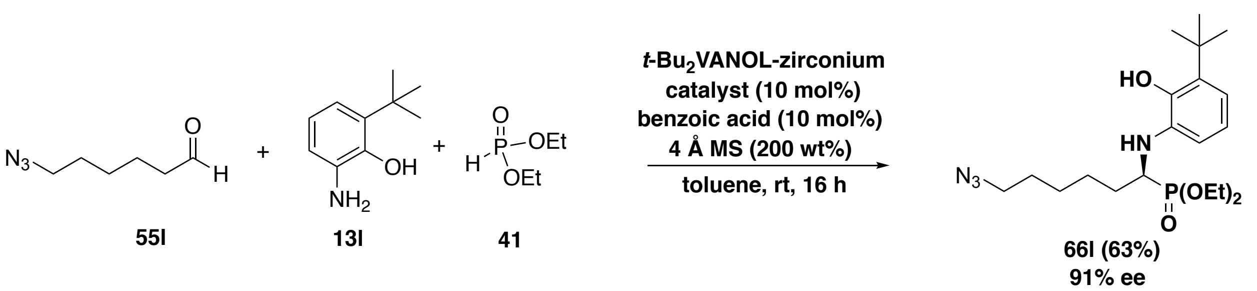

Diethyl (*S*)-(5-azido-1-((3-(*tert*-butyl)-2-hydroxyphenyl)amino)pentyl)phosphonate **66l**: Reaction of 5-azidopentanal **55l** (14.0 mg, 0.10 mmol, 1.00 equiv) according to general procedure F afforded **66l** (25.8 mg, 0.063 mmol) after 16 hours in 63% yield and 91% *ee* as a yellow oil. R_f = 0.30 (hexane/EtOAc 7:3); Specific Rotation: $[\alpha]_D^{20}$= -7.19 (c 1.05, $CHCl_3$); Spectral data for **66l**: ^{1}H NMR (500 MHz, Chloroform-*d*) δ 7.00 (d, *J* = 7.9 Hz, 1H), 6.94 (d, *J* = 7.9 Hz, 1H), 6.77 (t, *J* = 7.9 Hz, 1H), 4.18 - 4.00 (m, 4H), 3.56 (dt, *J* = 14.0, 6.6 Hz, 1H), 3.22 (t, *J* = 6.9 Hz, 2H), 1.95 (m, 1H), 1.87 - 1.76 (m, 1H), 1.63 - 1.48 (m, 4H), 1.40 (s, 9H), 1.37 - 1.33 (m, 2H), 1.31 - 1.21 (m, 6H). ^{13}C NMR (126 MHz, Chloroform-*d*) δ 147.81, 120.14, 119.45, 62.87 (dd, *J* = 17.6, 7.0 Hz), 55.51, 54.29, 51.24, 34.80, 29.71 (3C), 28.50, 26.44, 25.59, 16.39, 16.34 (three sp^2 and one sp^3 carbons are not located). IR (thin film) 3375br, 2959vs, 2096vs, 1602s, 1441s, 1206vs, 1022vs, 752s cm$^-$1; HRMS (ESI-TOF) *m/z* found 427.2475 ($[M+H]^+$); calcd. 427.2474 for $C_{20}H_{36}N_4O_4P$; HPLC: DAICEL CHIRALPAK AD-H (Hexane/*i*PrOH = 98:2, 1 mL/min flow rate, 245 nm), 18.4 min for the minor peak and 27.0 min for the major peak.

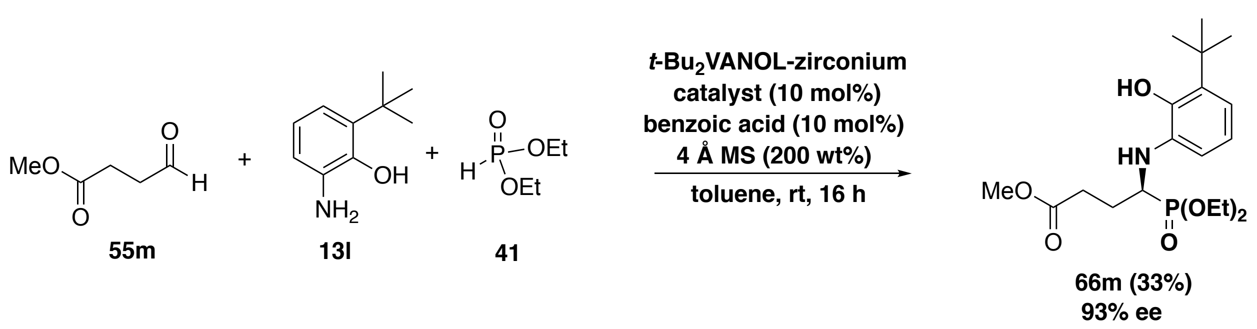

Methyl (*S*)-4-((3-(*tert*-butyl)-2-hydroxyphenyl)amino)-4-(diethoxyphosphoryl) butanoate **66m**: Reaction of methyl 4-oxobutanoate **55m** (11.6 mg, 0.10 mmol, 1.00 equiv) according to general procedure F afforded **66m** (13.2 mg, 0.033 mmol) after 16 hours in 33% yield and 93% *ee* as a yellow oil. R_f = 0.30 (hexane/EtOAc 7:3); Spectral data for **66m**: ^{1}H NMR (500 MHz, Chloroform-*d*) δ 6.99 (dd, *J* = 7.9, 1.6 Hz, 1H), 6.92 (dd, *J* = 7.9, 1.5 Hz, 1H), 6.79 (t, *J* = 7.9 Hz, 1H), 4.16 - 3.93 (m, 4H), 3.78 - 3.71 (m, 1H), 3.66 (s, 3H), 2.70 - 2.57 (m, 2H), 2.36 - 2.24 (m, 1H), 2.18 - 2.06 (m, 1H), 1.41 (s, 9H), 1.29 (t, *J* = 7.1 Hz, 3H), 1.22 (t, *J* = 7.1 Hz, 3H). ^{13}C NMR (126 MHz, Chloroform-*d*) δ 173.71, 147.54, 127.43, 126.39, 122.00, 120.29, 119.01, 62.91 (d, *J* = 6.9 Hz), 54.44, 53.23, 51.84, 34.78, 30.53, 29.75 (3C), 25.12, 16.34 (one sp^3 carbon is not located). IR (thin film) 3376br, 2956s, 1707s, 1213vs, 1023s, 748vs cm$^-$1; HRMS (ESI-TOF) *m/z* found 402.2048 ($[M+H]^+$); calcd. 402.2045 for $C_{19}H_{33}NO_6P$; HPLC: DAICEL CHIRALPAK IA-H (Hexane/*i*PrOH = 99:1, 1 mL/min flow rate, 245 nm), 14.9 min for the minor peak and 15.9 min for the major peak.

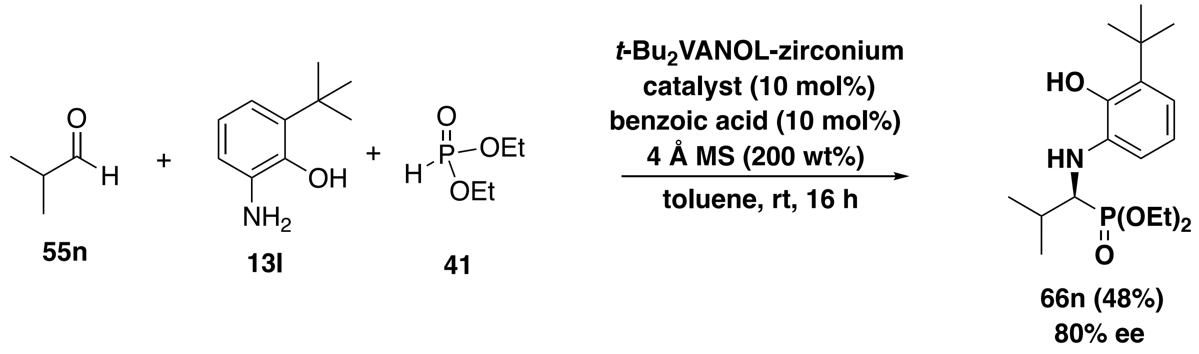

Diethyl (*S*)-(1-((3-(*tert*-butyl)-2-hydroxyphenyl)amino)-2-methylpropyl) phosphonate **66n**: Reaction of *iso*butyraldehyde **55n** (9.10 μL, 0.10 mmol, 1.00 equiv) according to general procedure F afforded **66n** (17.1 mg, 0.048 mmol) after 16 hours in 48% yield and 80% *ee* as a yellow oil. R_f = 0.30 (hexane/EtOAc 7:3); Spectral data for **66n**: ^{1}H NMR (500 MHz, Chloroform-*d*) δ 6.87 (dd, *J* = 7.6, 1.9 Hz, 1H), 6.82 (br, 1H, NH), 6.80 - 6.73 (m, 2H), 4.17 - 4.09 (m, 2H), 4.06 - 3.86 (m, 2H), 3.38 (dd, *J* = 16.3, 3.7 Hz, 1H), 2.33 - 2.20 (m, 1H), 1.40 (s, 9H), 1.30 (t, *J* = 7.1 Hz, 3H), 1.17 (t, *J* = 7.1 Hz, 3H), 1.12 (d, *J* = 6.9 Hz, 6H). ^{13}C NMR (126 MHz, Chloroform-*d*) δ 147.10, 139.83, 137.33, 136.56, 119.88, 118.04, (dd, *J* = 15.8, 7.0 Hz), 62.20, 60.9, 59.73, 34.61, 29.77 (3C), 20.38, 20.29, 18.24, 16.41 (d, *J* = 5.7 Hz), 16.28 (d, *J* = 5.7 Hz). IR (thin film) 3375br, 3308br, 2923vs, 2356s, 1585s, 1422vs, 1204vs, 1021vs, 732s cm$^-$1; HRMS (ESI-TOF) *m/z* found 380.1967 ([M+Na]$^+$); calcd. 380.1967 for $C_{18}H_{32}NO_4PNa$; HPLC: DAICEL CHIRALPAK IA-H (Hexane/*i*PrOH = 99:1, 1 mL/min flow rate, 245 nm), 11.0 min for the minor peak and 13.0 min for the major peak.

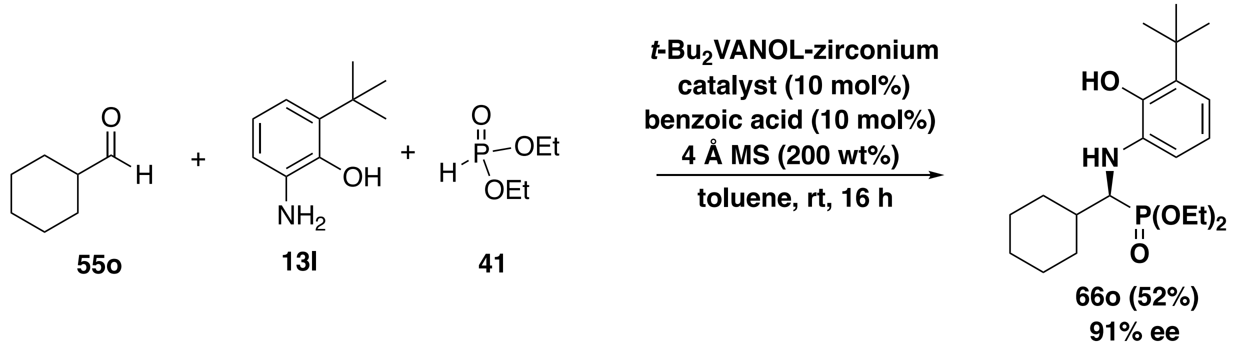

Diethyl (*S*)-(((3-(*tert*-butyl)-2-hydroxyphenyl)amino)(cyclohexyl)methyl) phosphonate **66o**: Reaction of cyclohexanecarbaldehyde **55o** (12.0 μL, 0.10 mmol, 1.00 equiv) according to general procedure F afforded **66o** (20.6 mg, 0.052 mmol) after 16 hours in 52% yield and 91% *ee* as a yellow oil. R_f = 0.30 (hexane/EtOAc 7:3). Spectral data for **66o**:^{1}H NMR (500 MHz, Chloroform-*d*) δ 7.02 (br, 1H, NH), 6.89 - 6.79 (m, 1H), 6.79 - 6.69 (m, 2H), 4.13 (p, *J* = 7.2 Hz, 2H), 4.08 - 4.00 (m, 1H), 3.97 - 3.87 (m, 1H), 3.42 (dd, *J* = 17.1, 3.8 Hz, 1H), 1.98 - 1.82 (m, 2H), 1.82 - 1.68 (m, 3H), 1.69 - 1.61 (m, 1H), 1.40 (s, 9H), 1.31 (t, *J* = 7.1 Hz, 5H), 1.27 - 1.21 (m, 3H), 1.14 (t, *J* = 7.1 Hz, 3H). ^{13}C NMR (126 MHz, Chloroform-*d*) δ 146.39, 137.51, 136.44, 119.81, 119.28, 116.75, 62.37 (dd, *J* = 36.2, 7.5 Hz), 60.06, 58.87, 39.70, 34.65, 30.59, 30.50, 29.78, 28.67, 28.64, 26.46, 26.35, 26.07,16.34 (d, *J* = 5.8 Hz), 16.31 (d, *J* = 5.8 Hz). IR (thin film) 3370br, 2924vs, 2096vs, 1713s, 1205s, 1021vs, 754s cm^{-}1; HRMS (ESI-TOF) *m/z* found 398.2432 ([M+H]$^+$); calcd. 398.2460 for $C_{21}H_{37}NO_4P$; HPLC: DAICEL CHIRALPAK AD-H (Hexane/*i*PrOH = 99:1, 1 mL/min flow rate, 245 nm), 8.3 min for the minor peak and 10.9 min for the major peak.

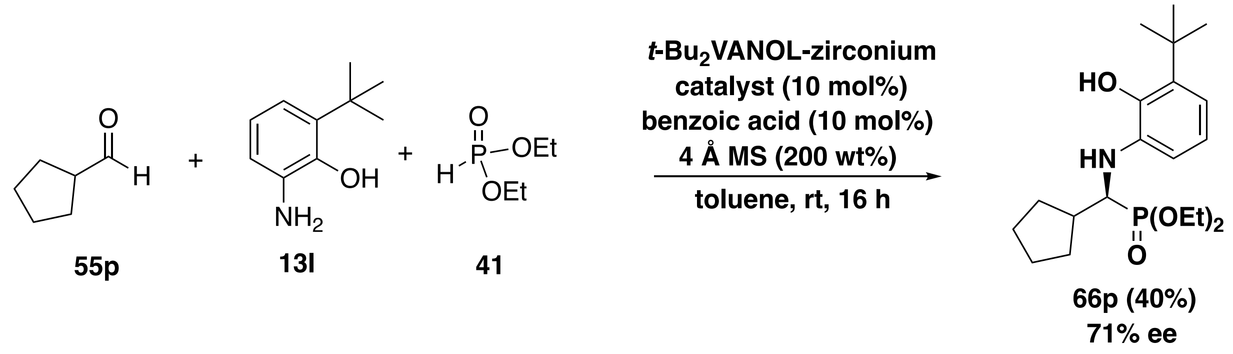

Diethyl (*S*)-(((3-(*tert*-butyl)-2-hydroxyphenyl)amino)(cyclopentyl)methyl) phosphonate **66p**: Reaction of cyclopentanecarbaldehyde **55p** (11.0 μL, 0.10 mmol, 1.00 equiv) according to general procedure F afforded **66p** (15.3 mg, 0.040 mmol) after 16 hours in 40% yield and 71% *ee* as a yellow oil. R_f = 0.30 (hexane/EtOAc 7:3); Spectral data for **66p**: ^{1}H NMR (500 MHz, Chloroform-*d*) δ 6.90 (dd, *J* = 7.9, 1.6 Hz, 1H), 6.84 (dd, *J* = 7.9, 1.6 Hz, 1H), 6.74 (td, *J* = 7.8, 1.6 Hz, 1H), 4.15 - 4.02 (m, 3H), 4.01 - 3.88 (m, 1H), 3.54 (dd, *J* = 14.0, 6.3 Hz, 1H), 2.38 (dq, *J* = 16.3, 8.2 Hz, 1H), 1.94 - 1.84 (m, 2H), 1.70 - 1.61 (m, 2H), 1.61 - 1.46 (m, 2H), 1.40 (s, 9H), 1.32 - 1.23 (m, 5H), 1.20 (t, *J* = 7.1 Hz, 3H). ^{13}C NMR (126 MHz, Chloroform-*d*) δ 149.20, 147.24, 134.53, 120.14, 118.86, 118.74, 62.64, 59.30, 58.00, 40.99, 34.74, 30.08, 29.79, 29.17 (3C), 25.30, 25.17, 16.35, 16.31. IR (thin film) 3370br, 2923vs, 1715s, 1443s, 1258s, 1017vs, 756vs cm^{-}1; HRMS (ESI-TOF) *m/z* found 406.2133 ([M+Na]$^{+}$); calcd. 406.2123 for $C_{20}H_{34}NO_4PNa$; HPLC: DAICEL CHIRALPAK AD-H (Hexane/*i*PrOH = 99:1, 1 mL/min flow rate, 245 nm), 18.2 min for the minor peak and 23.0 min for the major peak.

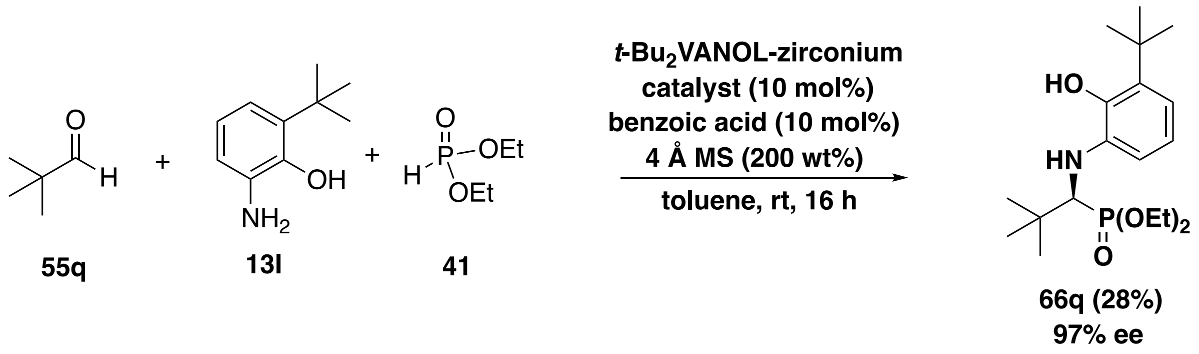

Diethyl (*S*)-(1-((3-(*tert*-butyl)-2-hydroxyphenyl)amino)-2,2-dimethylpropyl) phosphonate **66q**: Reaction of pivalaldehyde **55q** (10.7 μL, 0.10 mmol, 1.00 equiv) according to general procedure F afforded **66q** (10.4 mg, 0.028 mmol) after 16 hours in 28% yield and 97% *ee* as a yellow oil. R_f = 0.30 (hexane/EtOAc 7:3); Spectral data for **66q**: ^{1}H NMR (500 MHz, Chloroform-*d*) δ 6.82 - 6.70 (m, 3H), 4.09 (p, *J* = 7.3 Hz, 2H), 4.06 - 3.98 (m, 1H), 3.97 - 3.87 (m, 1H), 3.37 (d, *J* = 16.9 Hz, 1H), 1.41 (s, 9H), 1.27 - 1.23 (m, 6H), 1.15 (s, 9H). ^{13}C NMR (126 MHz, Chloroform-*d*) δ 120.65, 62.54, 62.20, 61.83, 34.56, 29.97, 29.70 (3C), 27.81, 16.31, 16.26 (five sp^2 and three sp^3 carbons are not located). IR (thin film) 3378br, 2958s, 2280s, 1712s, 1213vs, 1021vs, 751s cm^{-}1; HRMS (ESI-TOF) *m/z* found 372.2299 ($[M+H]^+$); calcd. 372.2304 for $C_{19}H_{34}NO_4P$; HPLC: DAICEL CHIRALPAK IA-H (Hexane/*i*PrOH = 99:1, 1 mL/min flow rate, 245 nm), 9.8 min for the minor peak and 11.9 min for the major peak.

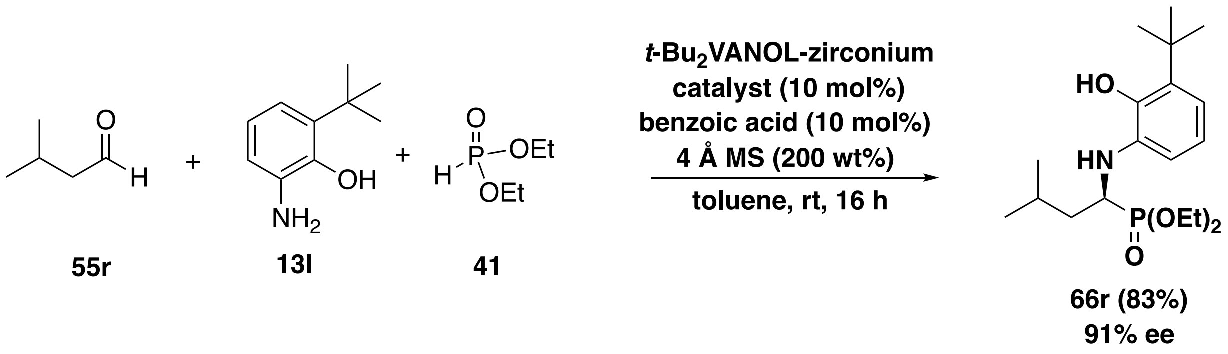

Diethyl (*S*)-(1-((3-(*tert*-butyl)-2-hydroxyphenyl)amino)-3-methylbutyl)phosphonate **66r**: Reaction of 3-methylbutanal **55r** (10.7 μL, 0.10 mmol, 1.00 equiv) according to general procedure F afforded **66r** (30.6 mg, 0.083 mmol) after 16 hours in 83% yield and 91% *ee* as a yellow oil. R_f = 0.30 (hexane/EtOAc 7:3); Spectral data for **66r**: ^{1}H NMR (500 MHz, Chloroform-*d*) δ 6.88 (br, 1H, NH), 6.82 (dd, *J* = 7.8, 1.6 Hz, 1H), 6.74 (t, *J* = 7.8 Hz, 1H), 4.17 - 3.91 (m, 4H), 3.61 - 3.52 (m, 1H), 1.97 - 1.84 (m, 1H), 1.72 - 1.64 (m, 2H), 1.40 (s, 9H), 1.30 (t, *J* = 7.1 Hz, 3H), 1.19 (t, *J* = 7.1 Hz, 3H), 0.92 (d, *J* = 6.6 Hz, 3H), 0.87 (d, *J* = 6.6 Hz, 3H). ^{13}C NMR (126 MHz, Chloroform-*d*) δ 146.81, 137.29, 135.87, 119.71, 119.66, 117.12, 62.52 (dd, *J* = 45.4, 7.6 Hz), 52.87, 51.64, 39.94, 34.62, 29.74 (3C), 24.46, 23.16, 21.65, 6.43 (d, *J* = 5.7 Hz), 16.34 (d, *J* = 5.6 Hz). IR (thin film) 3380br, 2927s, 2097vs, 1711s, 1213vs, 1022s, 750vs cm^{-}1; HRMS (ESI-TOF) *m/z* found 394.2138 ($[M+Na]^+$); calcd. 394.2123 for $C_{19}H_{34}NO_4PNa$; HPLC: DAICEL CHIRALPAK AD-H (Hexane/*i*PrOH = 99:1, 1 mL/min flow rate, 222 nm), 17.8 min for the minor peak and 20.6 min for the major peak.

7. Procedure for the deprotection of 51a from benzaldehyde

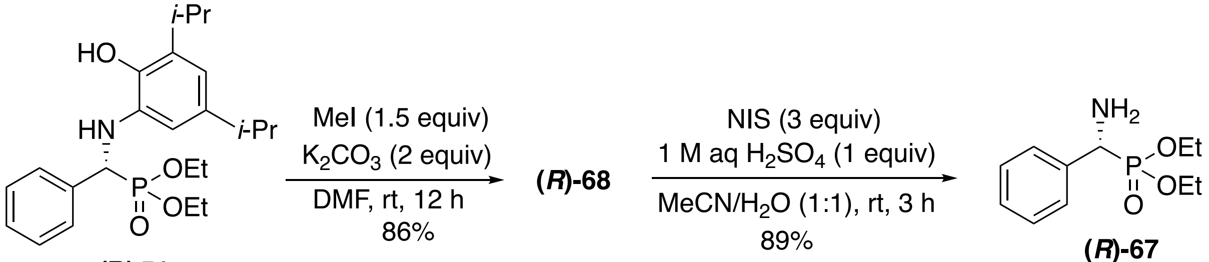

Diethyl (((3,5-diisopropyl-2-methoxyphenyl)amino)(phenyl)methyl)phosphonates **67**: To an oven-dried 10 mL round bottom flask were added (*R*)-**51a** (42.1 mg, 0.100 mmol, 1.00 equiv), potassium carbonate (280 mg, 0.200 mmol, 2.00 equiv) and dried DMF (1.0 mL). The mixture was stirred for 5 minutes followed by the addition of methyl iodine (9.3 μL, 0.15 mmol, 1.5 equiv). The reaction was stirred for 12 hours at room temperature and quenched with water (5.0 mL). The aqueous layer was extracted with diethyl ether (5.0 mL x 3). The combined organic layer was dried over $NaSO_4$ and concentrated under rotary evaporation. Flash chromatography (silica gel, 10 mm x 100 mm, hexane/EtOAc, 7:3) afforded the (*R*)-**68** (37.2 mg, 0.086 mmol, 86%) as a light yellow oil. R_f = 0.30 (hexane/EtOAc 7:3); Specific Rotation: $[\alpha]_D^{20}$= +5.6 (c 1.0, $CHCl_3$); Spectral data for (*R*)-**68**: ^1H NMR (500 MHz, Chloroform-*d*) δ 7.54 - 7.47 (m, 2H), 7.38 - 7.29 (m, 2H), 7.29 - 7.21 (m, 1H), 6.44 (d, *J* = 2.0 Hz, 1H), 6.17 (d, *J* = 2.1 Hz, 1H), 4.75 (d, *J* = 23.7 Hz, 1H), 4.18 - 3.93 (m, 3H), 3.79 (s, 3H), 3.78 - 3.74 (m, 1H), 3.28 (hept, *J* = 6.9 Hz, 1H), 2.66 (hept, *J* = 6.9 Hz, 1H), 1.27 (t, *J* = 7.1 Hz, 3H), 1.24 - 1.17 (m, 6H), 1.15 (t, *J* = 7.1 Hz, 3H), 1.09 (d, *J* = 6.9 Hz, 3H), 1.04 (d, *J* = 6.9 Hz, 3H). ^{13}C NMR (126 MHz, Chloroform-*d*) δ 144.88, 142.61, 140.65, 139.14, 139.03, 136.27, 128.52, 127.94, 127.90, 127.83, 113.34, 108.00, 63.12 (t, *J* = 7.6 Hz), 60.50, 56.64, 55.44, 33.98, 26.39,

24.11, 23.93, 23.84, 23.82, 6.46 (d, *J* = 5.8 Hz), 16.25 (d, *J* = 5.9 Hz). IR (thin film) 2956s, 1740vs, 1591s, 1442s, 1366vs, 1263vs, 733vs $cm^{-}1$; HRMS (ESI-TOF) *m/z* found 434.2460 ($[M+H]^+$); calcd. 434.2460 for $C_{24}H_{37}NO_4P$.

Diethyl (amino(phenyl)methyl)phosphonates (*R*)-**67**: To an oven-dried 5 mL round bottom flask were added (*R*)-**68** (22.0 mg, 0.0500 mmol, 1.00 equiv), NIS (33.7 mg, 0.150 mmol, 3.00 equiv), sulfuric acid (1 M, 0.05 mL, 0.05 mmol, 1.00 equiv) and acetonitrile/water (1.00 mL, 1:1). The reaction was stirred for 3 hours at room temperature and quenched with 5 M potassium hydroxide solution (2.0 mL). The aqueous layer was extracted with diethyl ether (3.0 mL x 3). The combined organic layer was dried over $NaSO_4$ and concentrated under rotary evaporation. Flash chromatography (silica gel, 10 mm x 100 mm, Hexane/EtOAc 1:1, then $CHCl_3$/MeOH, 9:1) afforded the (*R*)-**67** (10.8 mg, 0.0444 mmol, 89%) as a light yellow oil. R_f = 0.1 (EtOAc); Specific Rotation: $[\alpha]_D^{20}$ =+10.1 (c 0.9, $CHCl_3$); Spectral data for (*R*)-**67**: ^1H NMR (500 MHz, Chloroform-*d*) δ 7.47 - 7.43 (m, 2H), 7.38 - 7.33 (m, 2H), 7.32 - 7.27 (m, 1H), 4.26 (d, *J* = 17.1 Hz, 1H), 4.09 - 3.96 (m, 3H), 3.91 - 3.83 (m, 1H),2.49-2.19 (br, 1H, NH^2), 1.28 (t, *J* = 7.1, 0.5 Hz, 3H), 1.18 (t, *J* = 7.1, 0.6 Hz, 3H). ^{13}C NMR (126 MHz, Chloroform-*d*) δ 128.46, 128.44, 127.87, 127.84, 127.72, 127.67, 62.77 (dd, *J* = 18.8, 7.2 Hz), 54.65, 53.47, 16.44 (d, *J* = 5.7 Hz), 16.32 (d, *J* = 5.7 Hz). IR (thin film) 3400br, 2925vs, 2359vs, 1650s, 1258s, 1026vs, 798s $cm^{-}1$; HRMS (ESI-TOF) *m/z* found 266.0924 $[(M+Na)^+$; calcd. 266.0922 for $C_{11}H_{18}NO_3PNa$. The absolute configuration was determined to be (*R*) by comparing the optical rotation with lthe iterature value reported for this compound[25] and by the ECCD spectrum (vide infra).

7a. Deprotection of 51a by ceric ammonium nitrate (CAN)

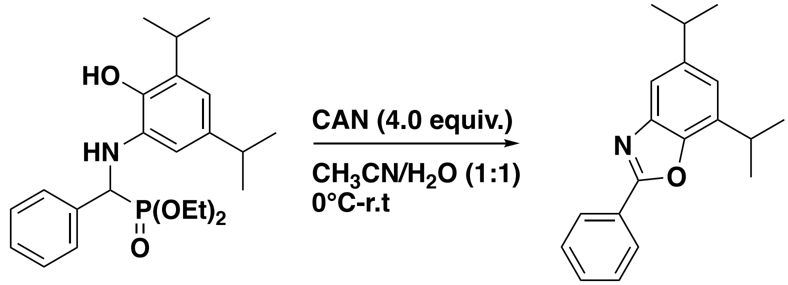

51a

111
34% (major side product)

5,7-Di*iso*propyl-2-phenylbenzo[*d*]oxazole **111**: To an oven-dried 5 mL round bottom flask were added **51a** (42.8 mg, 0.10 mmol, 1.00 equiv), CAN (0.22 g, 0.40 mmol, 4.0 equiv), and acetonitrile/water (0.80 mL, 1:1). The reaction was stirred for 6 hours at room temperature and quenched with aq sodium bicarbonate (1.0 mL). The aqueous layer was extracted with diethyl ether (3 x 2.00 mL). The combined organic layer was dried over $NaSO_4$ and concentrated under rotary evaporation. Flash chromatography (silica gel, 10 mm x 100 mm, EtOAc/Hexane, 2:8) afforded the **111** (9.4mg, 0.034 mmol, 34%) as a light yellow oil. R_f = 0.7 (hexane/EtOAc 7:3); Spectral data for **111**: ^{1}H NMR (500 MHz, Chloroform-*d*) δ 8.30 - 8.25 (m, 2H), 7.56 - 7.51 (m, 3H), 7.48 (d, *J* = 1.6 Hz, 1H), 7.08 - 7.05 (m, 1H), 3.38 (hept, *J* = 7.0 Hz, 1H), 3.03 (hept, *J* = 6.9 Hz, 1H), 1.45 (d, *J* = 7.0 Hz, 6H), 1.32 (d, *J* = 6.9 Hz, 6H). ^{13}C NMR (126 MHz, Chloroform-*d*) δ 162.71, 147.21, 145.86, 142.10, 131.50, 131.25, 128.86, 127.47, 121.66, 114.43, 34.35, 30.08, 24.50, 22.62 (one sp2 carbon is not located). IR (thin film) 2960vs, 2925vs, 1555s, 1450s, 1058s, 1026vs, 798s cm^-1; HRMS (ESI-TOF) *m/z* found 280.1705 [$(M+H)^+$; calcd. 280.1701 for $C_{19}H_{22}NO$.

8. General procedure for deprotection of 66r from 3-methylbutanal

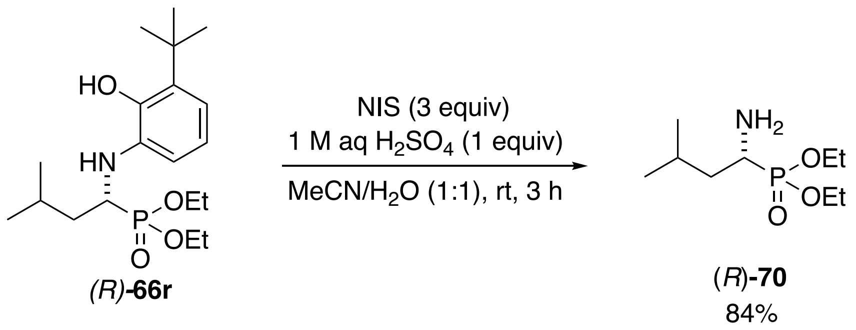

Diethyl (1-amino-3-methylbutyl)phosphonates **70**: To an oven-dried 25 mL round bottom flask were added (*R*)-**66r** (74.3 mg, 0.200 mmol, 1.00 equiv), NIS (135 mg, 0.600 mmol, 3.00 equiv), sulfuric acid (1 M, 0.200 mL, 0.200 mmol, 1.00 equiv) and acetonitrile/water (4.00 mL, 1:1). The reaction was stirred for 3 hours at room temperature and quenched with 5 M potassium hydroxide solution (5.0 mL). The aqueous layer was extracted with diethyl ether (5.0 mL x 2). The combined organic layer was dried over $NaSO_4$ and concentrated under rotary evaporation. Flash chromatography (silica gel, 10 mm x 100 mm, Hexane/EtOAc 1:1, then $CHCl_3$/MeOH, 9:1) afforded the (*R*)-**70** (37.5 mg, 0.168 mmol, 84%) as a light yellow oil. $[\alpha]_D^{20}$= -36.7 (c 0.72, $CHCl_3$); R_f = 0.1 (EtOAc). Spectral data for (*R*)-**70**: ^{1}H NMR (500 MHz, Chloroform-*d*) δ 4.17 (s, 4H), 3.10 (s, 1H), 1.93 (s, 1H), 1.54 (s, 2H), 1.35 (t, *J* = 6.5 Hz, 6H), 0.98 (d, *J* = 6.5 Hz, 3H), 0.92 (d, *J* = 6.4 Hz, 3H). ^{13}C NMR (126 MHz, Chloroform-*d*) δ 63.33, 46.00, 39.08, 24.37, 23.24, 21.37, 16.60 (2C). ^{31}P NMR (Chloroform-*d*) δ 26.24. IR (thin film) 3396br, 2921vs, 1653s, 1223s, 1023vs; HRMS (ESI-TOF) *m/z* found 246.1238 ($[M+Na]^+$); calcd. 246.1235 for $C_9H_{22}NO_3PNa$; The absolute configuration was determined to be (*R*) by comparing the optical rotation with a literature value reported for this compound[26] and by ECCD spectrum (vide infra).

9. Determination of the absolute configuration of 67 and 70 by ECCD

Exciton Coupled Circular Dichroism (ECCD)[27] offers a non-empirical chiroptical approach to assign the absolute stereochemistry of chiral molecules. In particular, bis-porphyrin tweezer methodology has been exploited over the years to determine the absolute stereochemistry of chiral molecules via the formation of a strong host-guest complex.[28] As shown in the scheme below, C_3-Zn-TPFPtz **112**[29] binds with **67** in a bidentate fashion. The steric interactions imposed by the asymmetric center induce a specific helicity of the host-guest complex. As illustrated, the porphyrin bound to the amino group differentiates between the H and Ph substituents, sliding away from the larger Ph group, and thus favoring the formation of *M*-helical complex. Since the observed handedness (negative ECCD spectra, Figure S1) is the direct consequence of the chirality of the guest molecule, the absolute stereochemistry of compound **67** is assigned as (*R*). Similarly, the absolute configuration of compound **70** was determined to be (*R*) as a result of its negative ECCD spectrum when bound to C_3-Zn-TPFPtz **112** (Figure S2). For the CD measurements, spectra were recorded on a JASCO J-810 spectropolarimeter, equipped with a temperature controller (Neslab 111), and the data are reported as Mol. CD / λ [nm].

C_3-Zn-TPFPtz **112** (1.0 μL of a 0.001 M solution in anhydrous dichloromethane) was added to hexane (1.0 mL) in a 1.0 cm CD cell (cooled to 0 °C) to obtain a 1.0 μM solution. The background spectrum was recorded from 350 nm to 480 nm with a scan rate of 100 nm/min at 0 °C. Chiral aminophosphonates **67** and **70** (5 μL, 5 equiv with

respect to the host) from a stock solution in anhydrous dichloromethane (0.001 M) were added to the prepared host solution to afford the host-guest complex. CD spectra were measured immediately (10 scans). The resultant ECCD spectra recorded in millidegrees were converted the molecular CD (Mol. CD), assuming the host concentration of 1.0 μM.

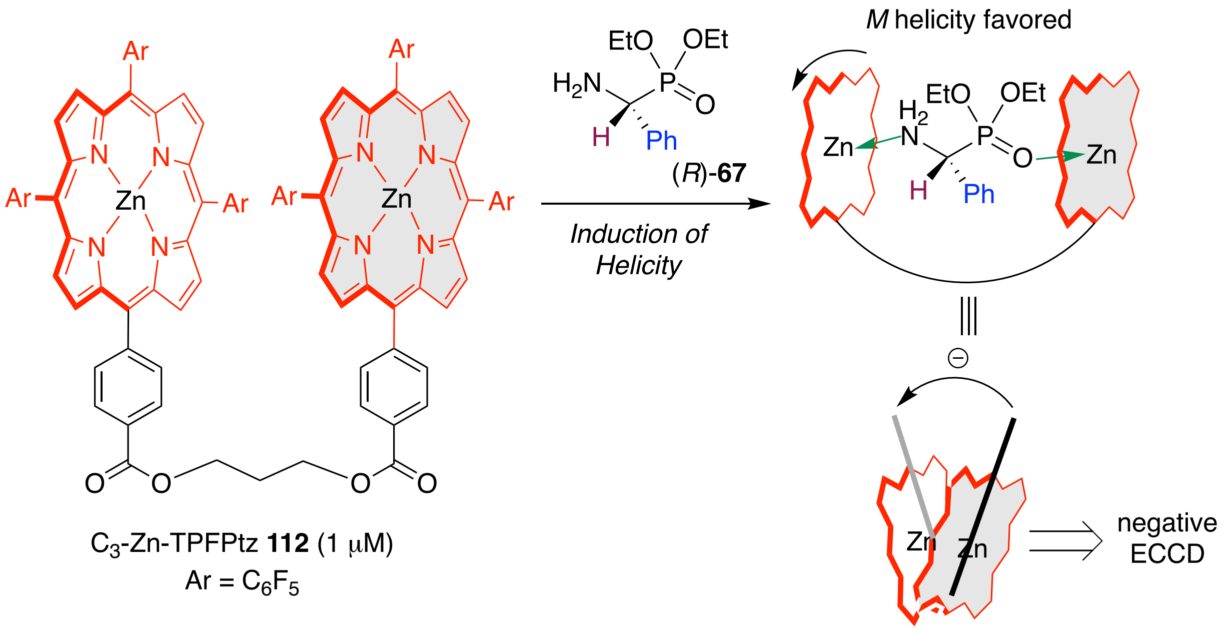

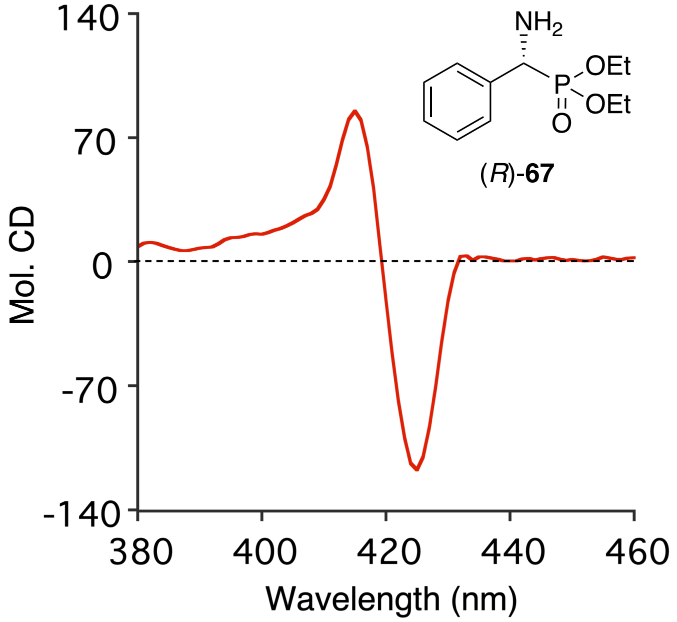

Figure S1 Negative ECCD spectrum of C_3-Zn-TPFPtz **112** complexed with 5 equiv of **67** at 0 °C in hexane.

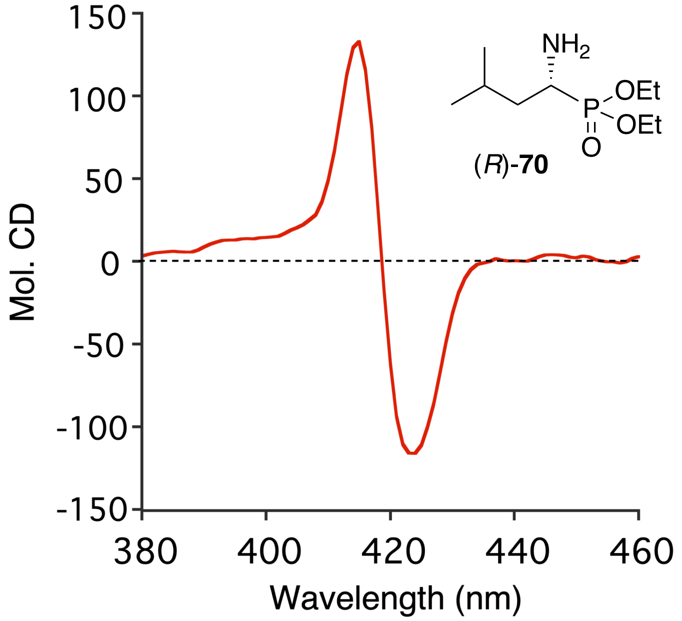

Figure S2 Negative ECCD spectrum of C_3-Zn-TPFPtz **112** complexed with 5 equiv of **70** at 0 °C in hexane.

10. Reference

1. Li, X. Y.; Tanasova, M.; Vasileiou, C.; Borhan, B., Fluorinated porphyrin tweezer: A powerful reporter of absolute configuration for erythro and threo diols, amino alcohols, and diamines. *J Am Chem Soc* **2008,** *130* (6), 1885-1893.

2. Zhang, Y.; Yeung, S. M.; Wu, H. Q.; Heller, D. P.; Wu, C. R.; Wulff, W. D., Highly enantioselective deracemization of linear and vaulted biaryl ligands. *Org Lett* **2003,** *5* (11), 1813-1816.

3. Su, Y.; Rabalakos, C.; Mitchell, W. D.; Wulff, W. D., New synthesis of vaulted biaryl ligands via the Snieckus phenol synthesis. *Org Lett* **2005,** *7* (3), 367-369.

4. Desai, A. A.; Huang, L.; Wulff, W. D.; Rowland, G. B.; Antilla, J. C., Gram-Scale Preparation of VAPOL Hydrogenphosphate: A Structurally Distinct Chiral Bronsted Acid. *Synthesis-Stuttgart* **2010,** (12), 2106-2109.

5. Ding, Z. S.; Osminski, W. E. G.; Ren, H.; Wulff, W. D., Scalable Syntheses of the Vaulted Biaryl Ligands VAPOL and VANOL via the Cycloaddition/Electrocyclization Cascade. *Org Process Res Dev* **2011,** *15* (5), 1089-1107.

6. Guan, Y.; Ding, Z. S.; Wulff, W. D., Vaulted Biaryls in Catalysis: A Structure-Activity Relationship Guided Tour of the Immanent Domain of the VANOL Ligand. *Chem-Eur J* **2013,** *19* (46), 15565-15571.

7. Guan, Y., PhD Dissertation. *Michigan State University* **2012**.

8. Vasudevan, J.; Beard, R. L.; Yuan, H.; Chandratratna, R. A. Preparation of 3,5-diisopropylphenyl-heptatrienoic acid derivatives having serum glucose reducing activity. US6759546B1, 2004.

9. Zhou, Y.; Xia, X.; Gao, Z., Synthesis and physical properties of some 1,4-bis(1,3-benzoxazol-2-yl)benzene derivatives. *Gaodeng Xuexiao Huaxue Xuebao* **1988,** *9* (11), 1122-5.

10. Alonso, F.; Yus, M., Easy Synthesis of 2,4-Dialkyl Substituted Phenols and Anisoles from Para-Benzoquinone. *Tetrahedron* **1992,** *48* (13), 2709-2714.

11. Vinsova, J.; Cermakova, K.; Tomeckova, A.; Ceckova, M.; Jampilek, J.; Cermak, P.; Kunes, J.; Dolezal, M.; Staud, F. Synthesis and antimicrobial evaluation of new 2-substituted 5,7-di-tert-butylbenzoxazoles. *Bioorg. Med. Chem*. **2006**, *14*, 5850-5865.

12. Benson, G. M.; Hertzberg, R. P.; Jurewicz, A. J.; Rutledge, M. C.; Veber, D. F.; Widdowson, K. L. Preparation of N,N'-diphenyl ureas as IL-8 receptor antagonists. WO2000076495A1, 2000.

13. Kano, F.; Yamane, T.; Yamashita, K.; Hosoe, K.; Watanabe, K. Alkyl-substituted benzoxazinorifamycin derivative, process for preparing the same and antibacterial agent containing the same. EP253340A1, 1988.

14. Shibuya, K.; Kawamine, K.; Sato, Y.; Miura, T.; Ozaki, C.; Edano, T.; Hirata, M.; Ohgiya, T. Preparation of novel cyclic diamine compounds as inhibitors of acyl CoA cholesterol acyltransferase (ACAT). US20040038987A1, 2004.

15. Waser, J.; Gaspar, B.; Nambu, H.; Carreira, E. M. Hydrazines and Azides via the Metal-Catalyzed Hydrohydrazination and Hydroazidation of Olefins. *J. Am. Chem. Soc*. **2006**, *128*, 11693-11712.

16. Liniger, M.; Neuhaus, C.; Hofmann, T.; Fransioli-Ignazio, L.; Jordi, M.; Drueckes, P.; Trappe, J.; Fabbro, D.; Altmann, K.-H. Kinase Inhibition by Deoxy Analogues of the Resorcylic Lactone L-783277. *ACS Med. Chem. Lett*. **2011**, *2*, 22-27.

17. Pirali, T.; Tron, G. C.; Zhu, J. P., One-pot synthesis of macrocycles by a tandem three-component reaction and intramolecular [3+2] cycloaddition. *Org Lett* **2006,** *8* (18), 4145-4148.

18. Sultane, P. R.; Bielawski, C. W., Burgess Reagent Facilitated Alcohol Oxidations in DMSO. *J Org Chem* **2017,** *82* (2), 1046-1052.

19. Zhou, Y. B.; Gupta, A. K.; Mukherjee, M.; Zheng, L.; Wulff, W. D., Multicomponent Catalytic Asymmetric Synthesis of trans-Aziridines. *J Org Chem* **2017,** *82* (24), 13121-13140.

20. Withall, D. M.; Haynes, S. W.; Challis, G. L., Stereochemistry and Mechanism of Undecylprodigiosin Oxidative Carbocyclization to Streptorubin B by the Rieske Oxygenase RedG. *J Am Chem Soc* **2015,** *137* (24), 7889-7897.

21. Attaba, N.; Taylor, J. E.; Slawin, A. M. Z.; Smith, A. D., Enantioselective NHC-Catalyzed Redox [4+2]-Hetero-Diels-Alder Reactions Using alpha,beta-Unsaturated Trichloromethyl Ketones as Amide Equivalents. *J Org Chem* **2015,** *80* (19), 9728-9739.

22. Wu, B.I Li, K.; Toy, P. H. Synthesis of Hydroxy-a-sanshool. *Synlett* **2012**, *23*, 2564-2566.

23. Firdous, S.; Banert, K.; Auer, A. A., Viability of 4,5-Dihydro-1,2,3,4-oxatriazoles Reinvestigated. *Chem-Eur J* **2011,** *17* (20), 5539-5543.

24. Shaikh, T. M.; Sudalai, A., Enantioselective Synthesis of (+)-alpha-Conhydrine and (-)-Sedamine by L-Proline-Catalysed alpha-Aminooxylation. *Eur J Org Chem* **2010,** (18), 3437-3444.

25. Davis, F. A.; Lee, S.; Yan, H. X.; Titus, D. D., Asymmetric synthesis of quaternary alpha-amino phosphonates using sulfinimines. *Org Lett* **2001,** *3* (11), 1757-1760.

26. Smith, A. B.; Yager, K. M.; Phillips, B. W.; Taylor, C. M., Asymmetric synthesis of diethyl (R)-(-)-(1-amino-3-methylbutyl)phosphonate. *Org Synth* **1998,** *75*, 19-30.

27. Harada, N.; Nakanishi, K.: *Circular Dichroic Spectroscopy - Exciton Coupling in Organic Stereochemistry*; University Science Books: Mill Valley, CA, 1983.

28. Gholami, H.; Chakraborty, D.; Zhang, J.; Borhan, B., Absolute stereochemical determination of organic molecules through induction of helicity in host-guest complexes. *Acc. Chem. Res.*, **2021**, *54*, 654-667.

29. Li, X.; Burrell, C. E.; Staples, R. J.; Borhan, B. Absolute configuration for 1,n-glycols: a nonempirical approach to long-range stereochemical determination. *J. Am. Chem. Soc.* **2012**, *134*, 9026-9029.
