# Supplementary material for: Zirconium-catalyzed asymmetric Kabachnik–Fields reactions of aromatic and aliphatic aldehydes
Source: Chem Sci. 2021 Aug 3;12(37):12333–45. doi: 10.1039/d1sc03222d (PMC8480333; doi:10.1039/d1sc03222d)
Supplement: SC-012-D1SC03222D-s002 [file SC-012-D1SC03222D-s002.pdf]

Electronic Supplementary Material (ESI) for Chemical Science.
This journal is © The Royal Society of Chemistry 2021

Supporting Information (NMR spectra) For:
Zirconium-Catalyzed Asymmetric Kabachnik-Fields Reactions of Aromatic and Aliphatic Aldehydes

Yijing Dai, Li Zheng, Debarshi Chakraborty,, Babak Borhan and William D. Wulff,*
Department of Chemistry, Michigan State University, East Lansing, MI 48824 wulff@chemistry.msu.edu

Table of Content

Compound	Page	Compound	Page
80	1, 2	55g	36
81	3, 4	102	37
13c	5, 6	55h	38
83	7, 8	55i	39, 40
84	9, 10	106	41
13d	11, 12	55j	42, 43
86	13, 14	108	44
13e	15, 16	55k	45
88	17	55l	46
13i	18, 19	55m	47, 48
90	20	51a	49, 50, 51
13j	21, 22	51b	52, 53
92	23	51c	54, 55
13k	24, 25	51d	56, 57
94	26, 27	51f	60, 61
13l	28, 29	51g	62, 63
31j	30, 31	51h	64, 65
55d	32	51i	66, 67, 68
55e	33	51j	69, 70, 71
55f	34	51k	72, 73
100	35	66a	74, 75

Table of Content (Continue)

Compound	Page	Compound	Page
66b	76, 77	66m	98, 99
66c	78, 79	66n	100, 101
66d	80, 81	66o	102, 103
66e	82, 83	66p	104, 105
66f	84, 85	66q	106, 107
66g	86, 87	66r	108, 109
66h	88, 89	68	110, 111
66i	90, 91	67	112, 113
66j	92, 93	111	114, 115
66k	94, 95	70	116, 117, 118
66l	96, 97		

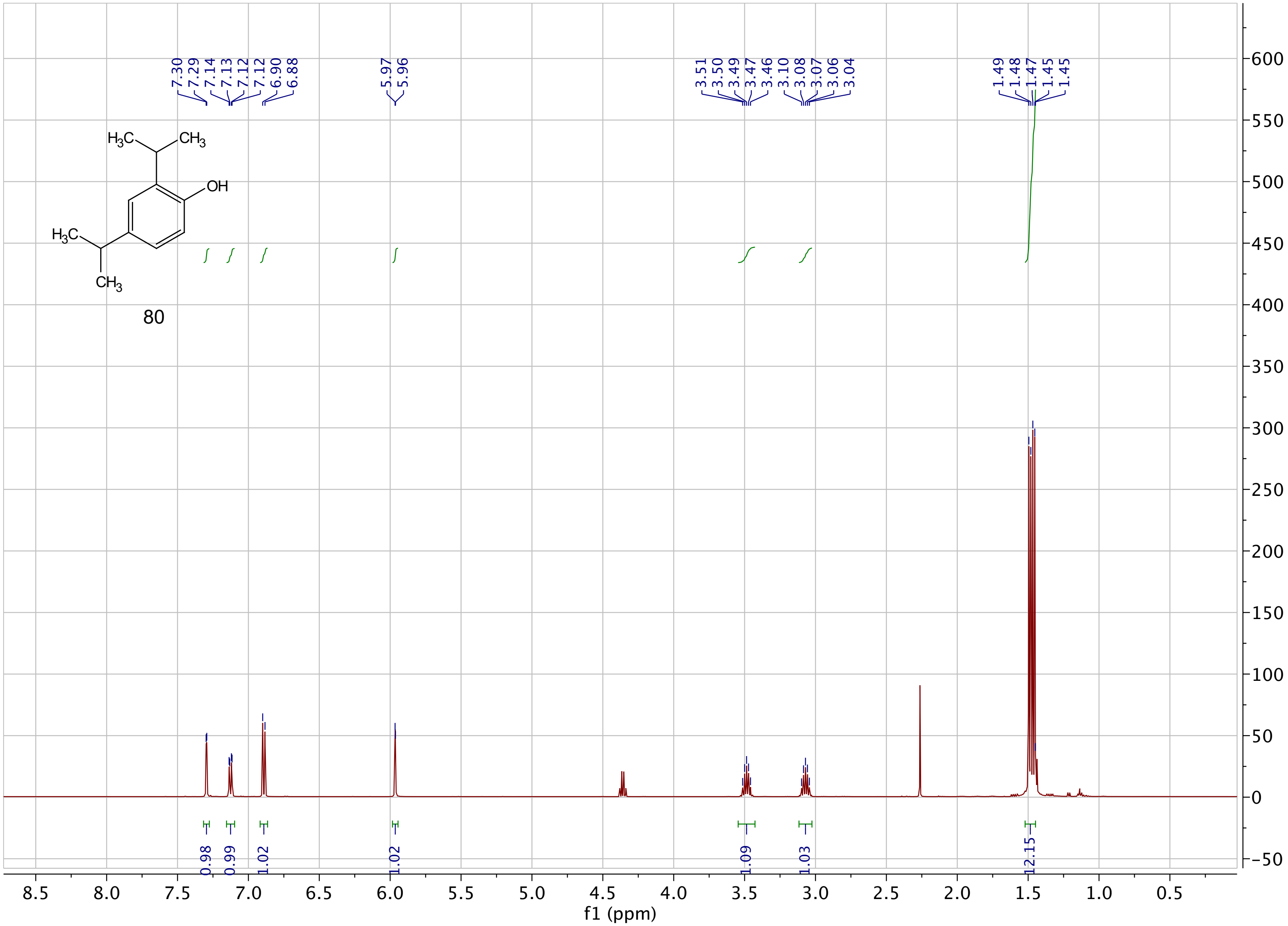

7.30
7.29
7.14
7.13
7.12
7.12
6.90
6.88
5.97
5.96
3.51
3.50
3.49
3.47
3.46
3.10
3.08
3.07
3.06
3.04
1.49
1.48
1.47
1.45
1.45
H3C
CH3
OH
H3C
CH3
0.98
0.99
1.02
1.02
1.09
1.03
12.15
f1 (ppm)

80

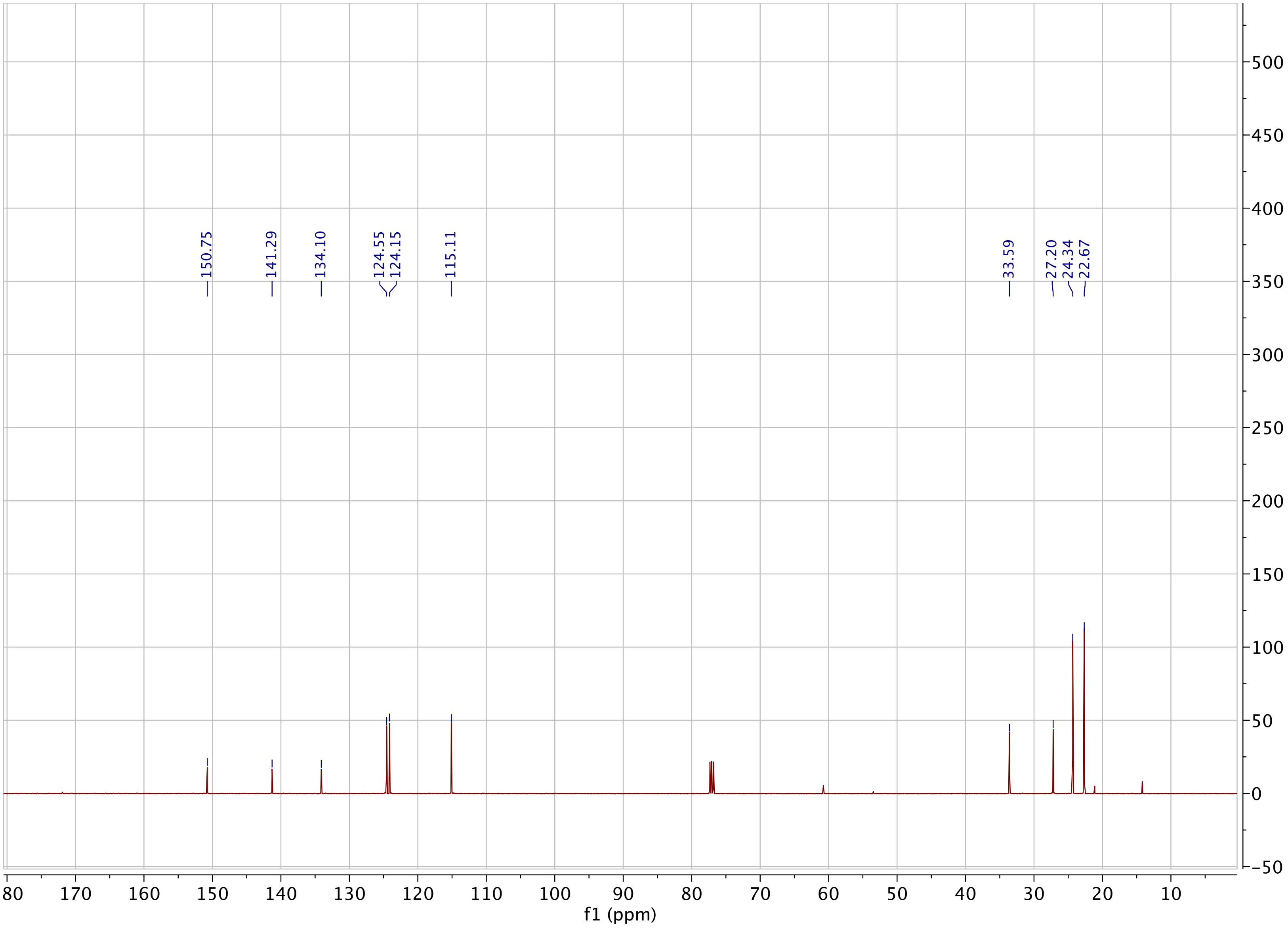
150.75
141.29
134.10
124.55
124.15
115.11
33.59
27.20
24.34
22.67
f1 (ppm)

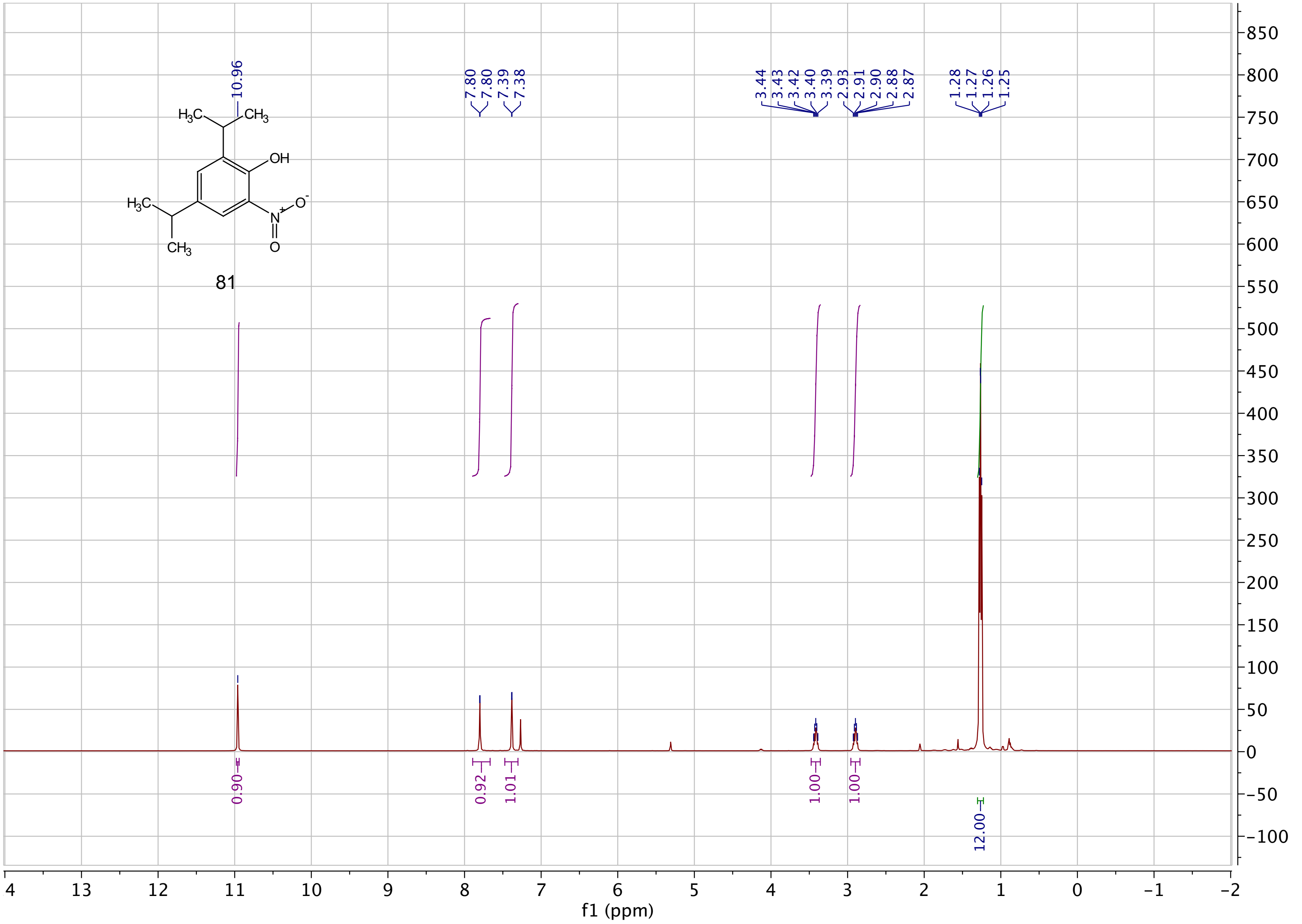

10.96
7.80
7.80
7.39
7.38
3.44
3.43
3.42
3.40
3.39
2.93
2.91
2.90
2.88
2.87
1.28
1.27
1.26
1.25
H3C
CH3
OH
H3C
CH3
N
O
O
81
0.90
0.92
1.01
1.00
1.00
12.00
f1 (ppm)

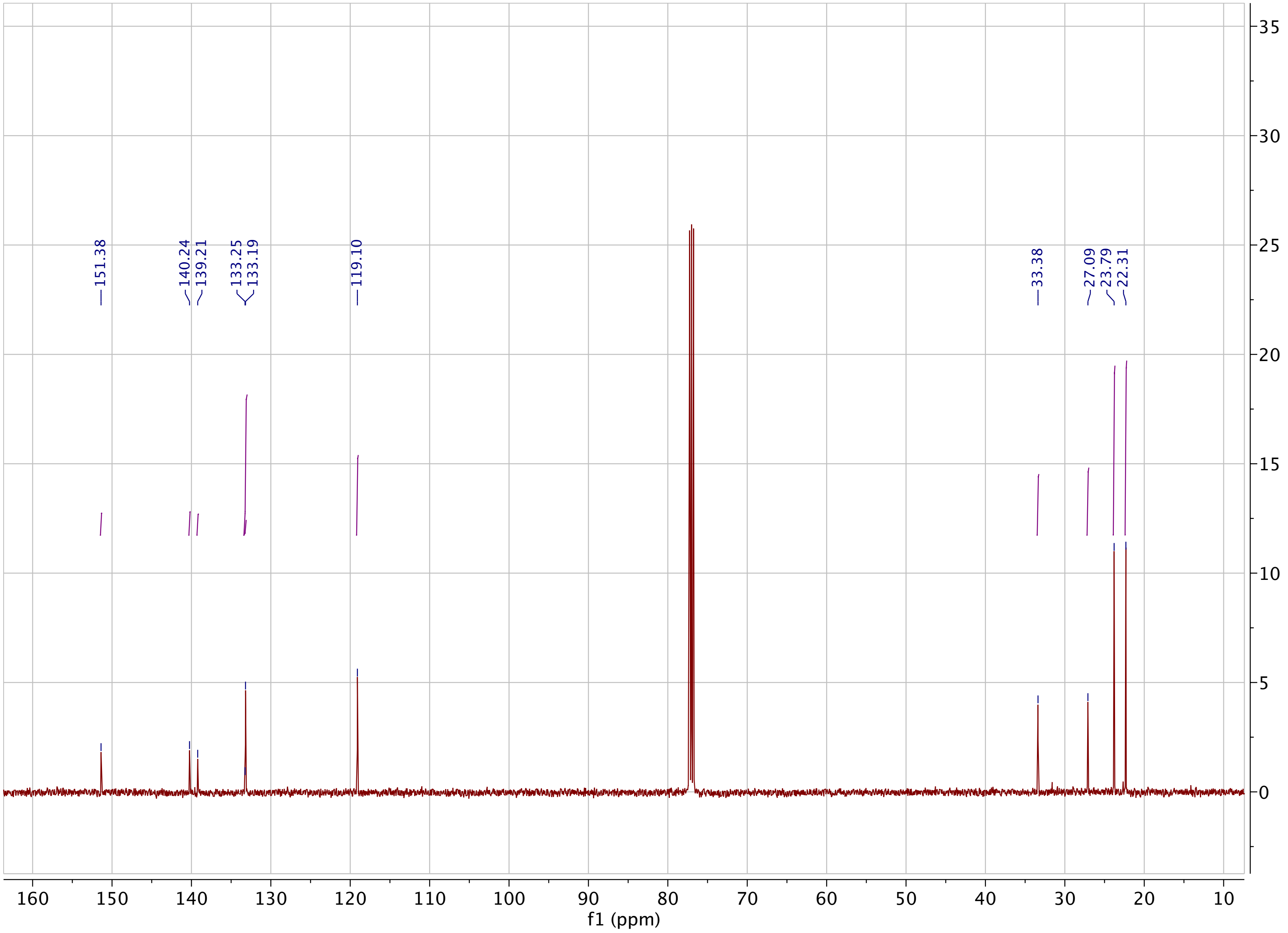

151.38
140.24
139.21
133.25
133.19
119.10
33.38
27.09
23.79
22.31
f1 (ppm)

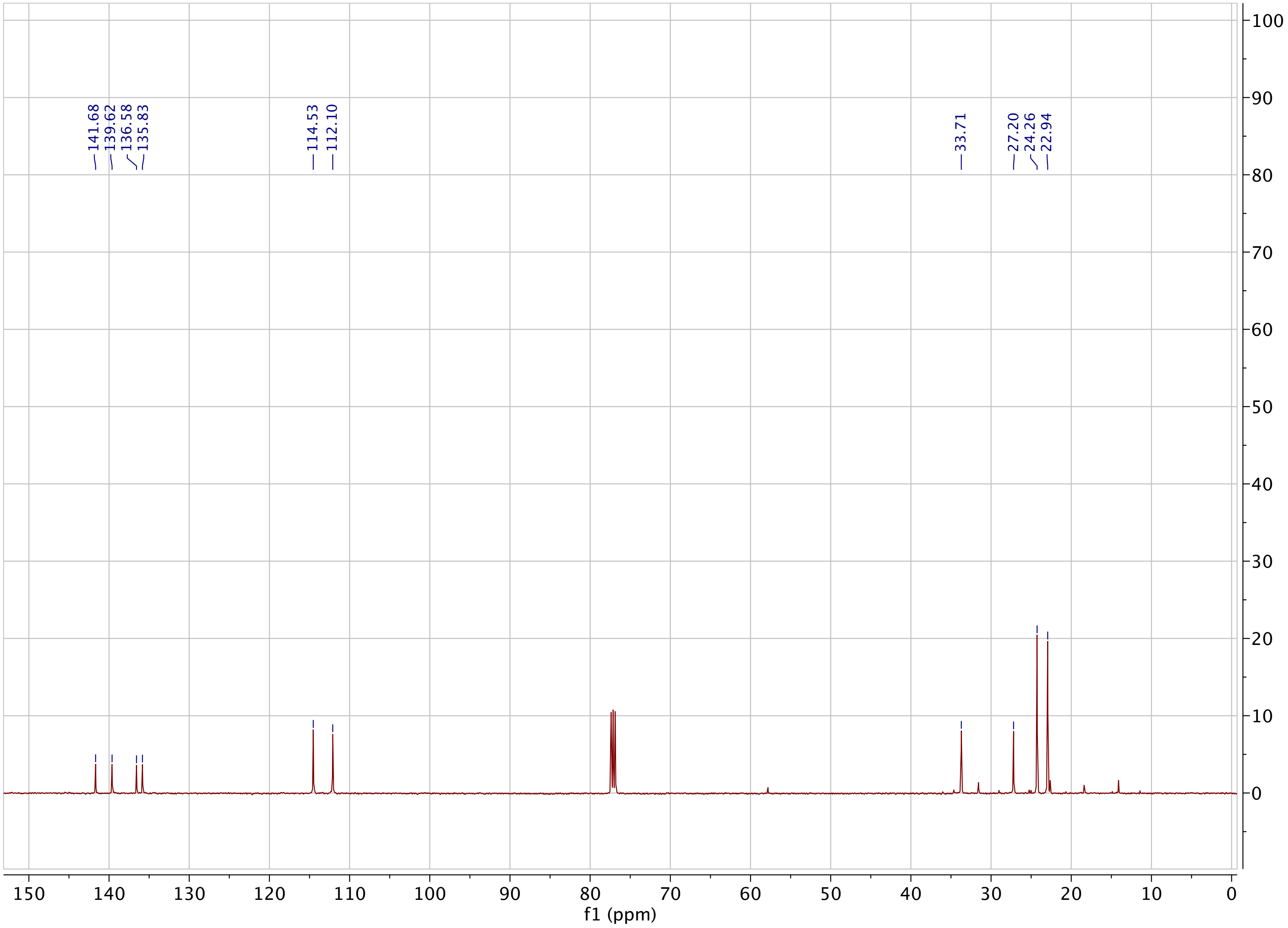

141.68
139.62
136.58
135.83
114.53
112.10
33.71
27.20
24.26
22.94
f1 (ppm)

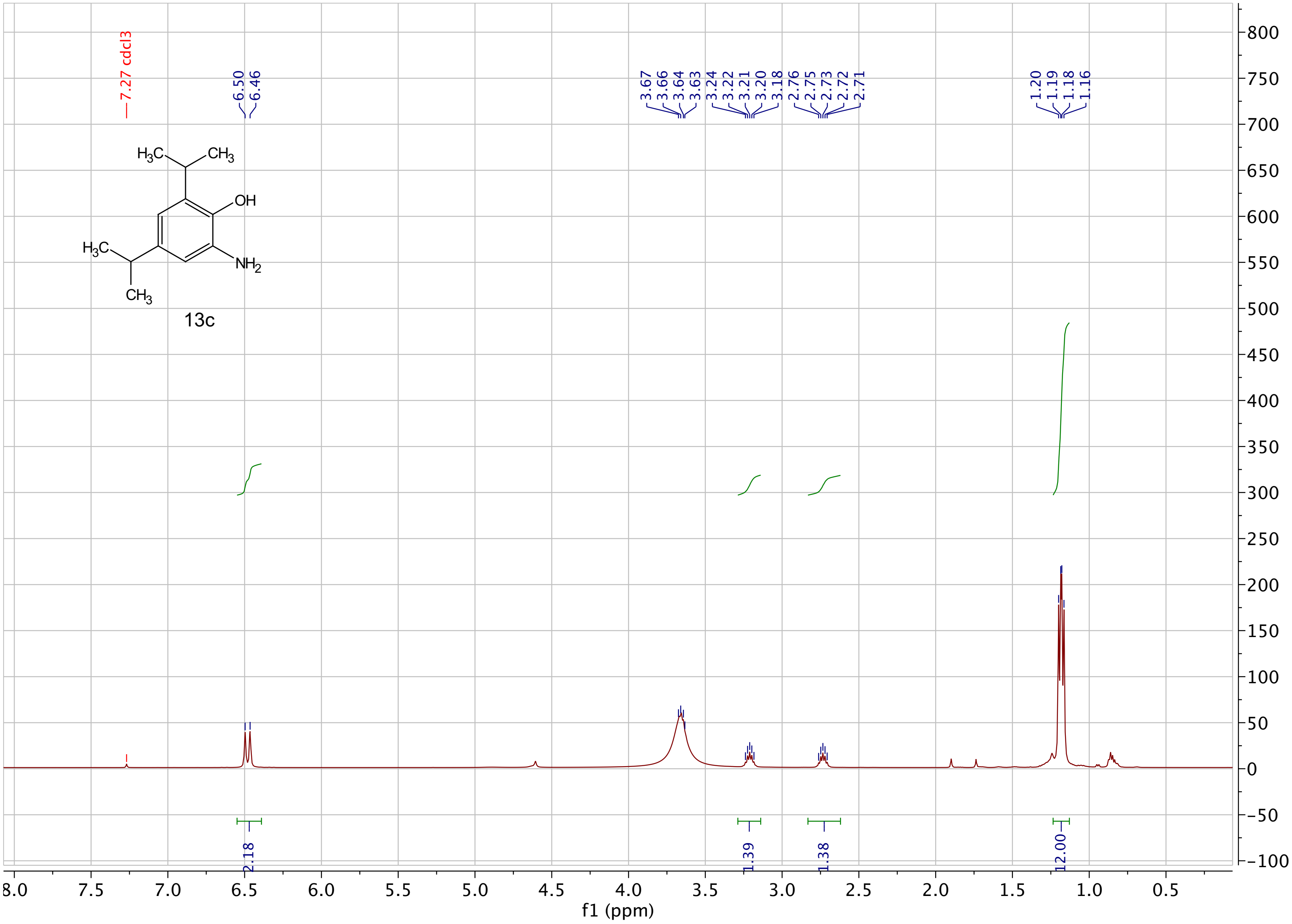
7.27 cdcl3
6.50
6.46
3.67
3.66
3.64
3.63
3.24
3.22
3.21
3.20
3.18
2.76
2.75
2.73
2.72
2.71
1.20
1.19
1.18
1.16
13c
2.18
1.39
1.38
12.00
f1 (ppm)

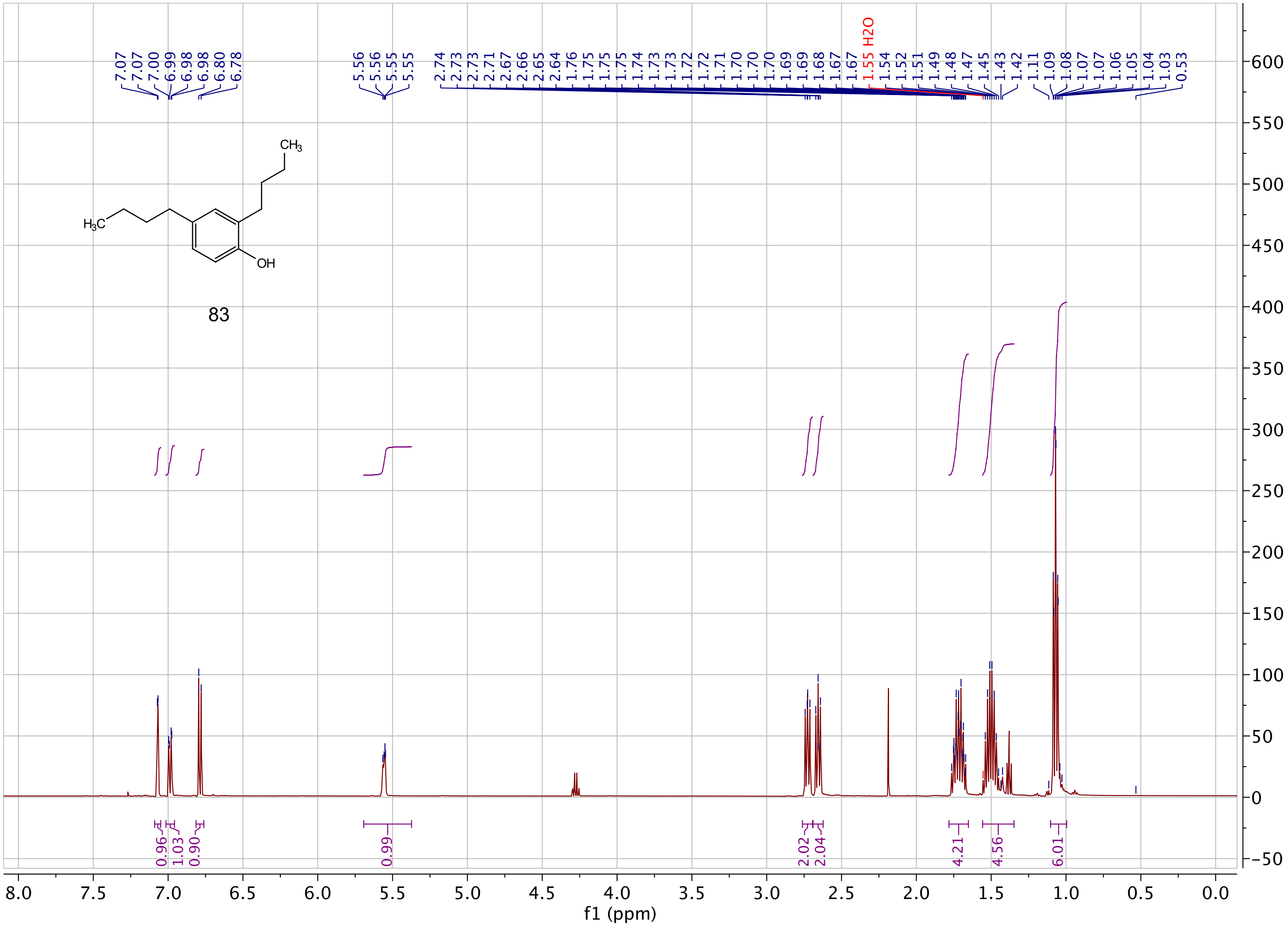

7.07 7.07 7.00 6.99 6.98 6.98 6.80 6.78
5.56 5.56 5.55 5.55
2.74 2.73 2.73 2.71 2.67 2.66 2.65 2.64 1.76 1.75 1.75 1.75 1.74 1.73 1.73 1.72 1.72 1.71 1.70 1.70 1.70 1.69 1.69 1.68 1.67 1.67 1.55 H2O 1.54 1.52 1.51 1.49 1.48 1.47 1.45 1.43 1.42 1.11 1.09 1.08 1.07 1.07 1.06 1.05 1.04 1.03 0.53
CH3
H3C
OH
83
0.96
1.03
0.90
0.99
2.02
2.04
4.21
4.56
6.01
f1 (ppm)

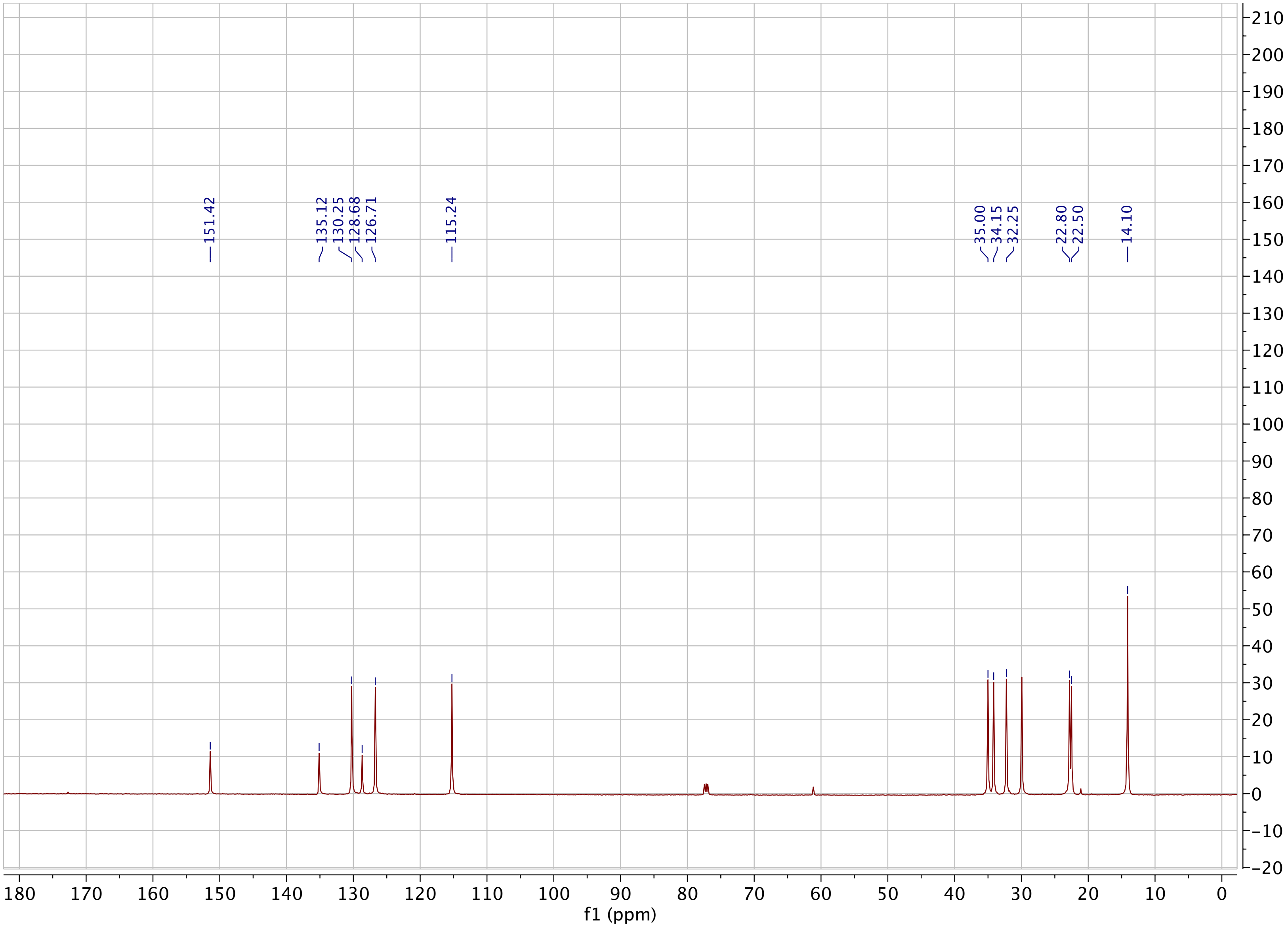
151.42
135.12
130.25
128.68
126.71
115.24
35.00
34.15
32.25
22.80
22.50
14.10
f1 (ppm)

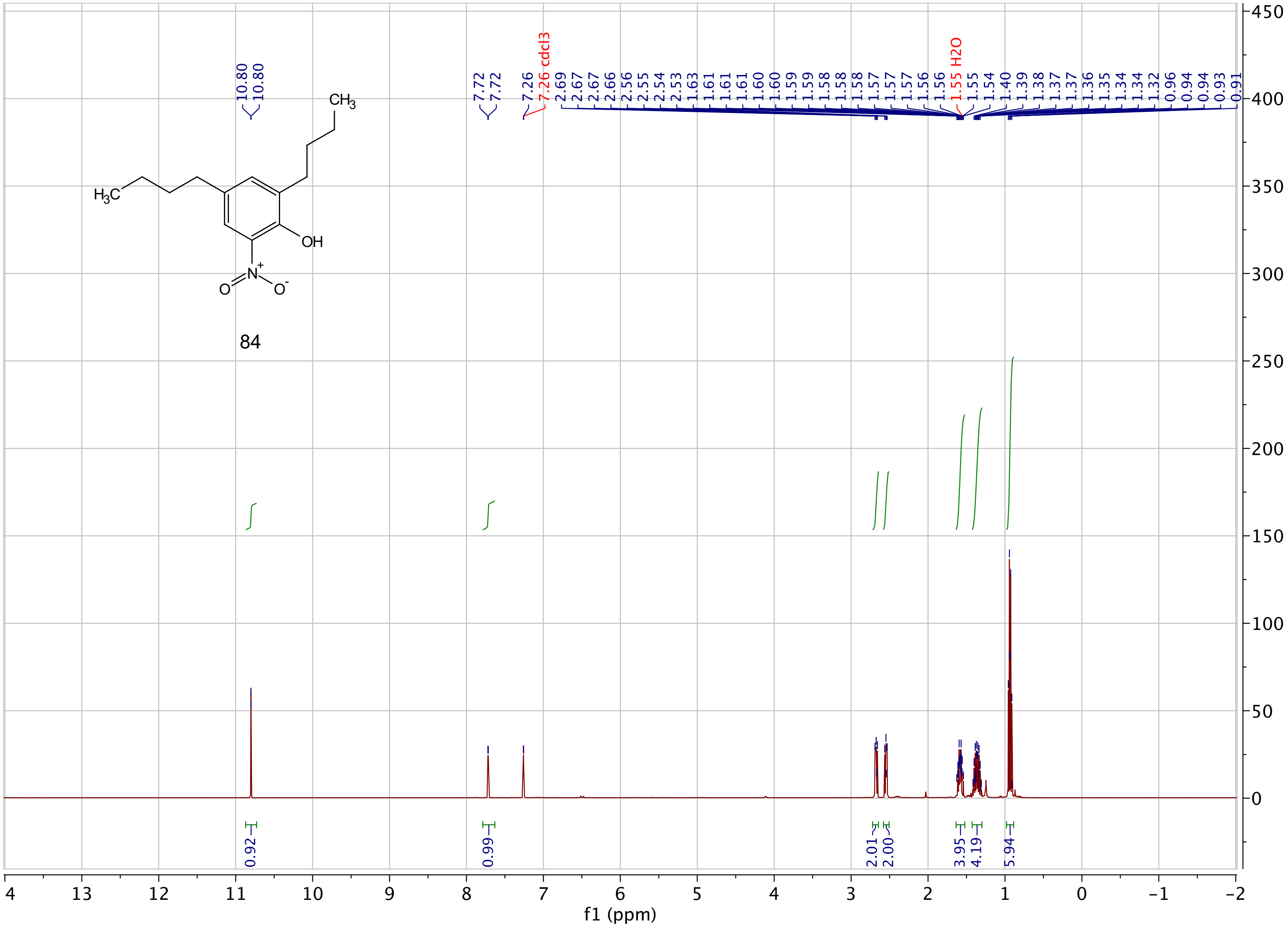

84

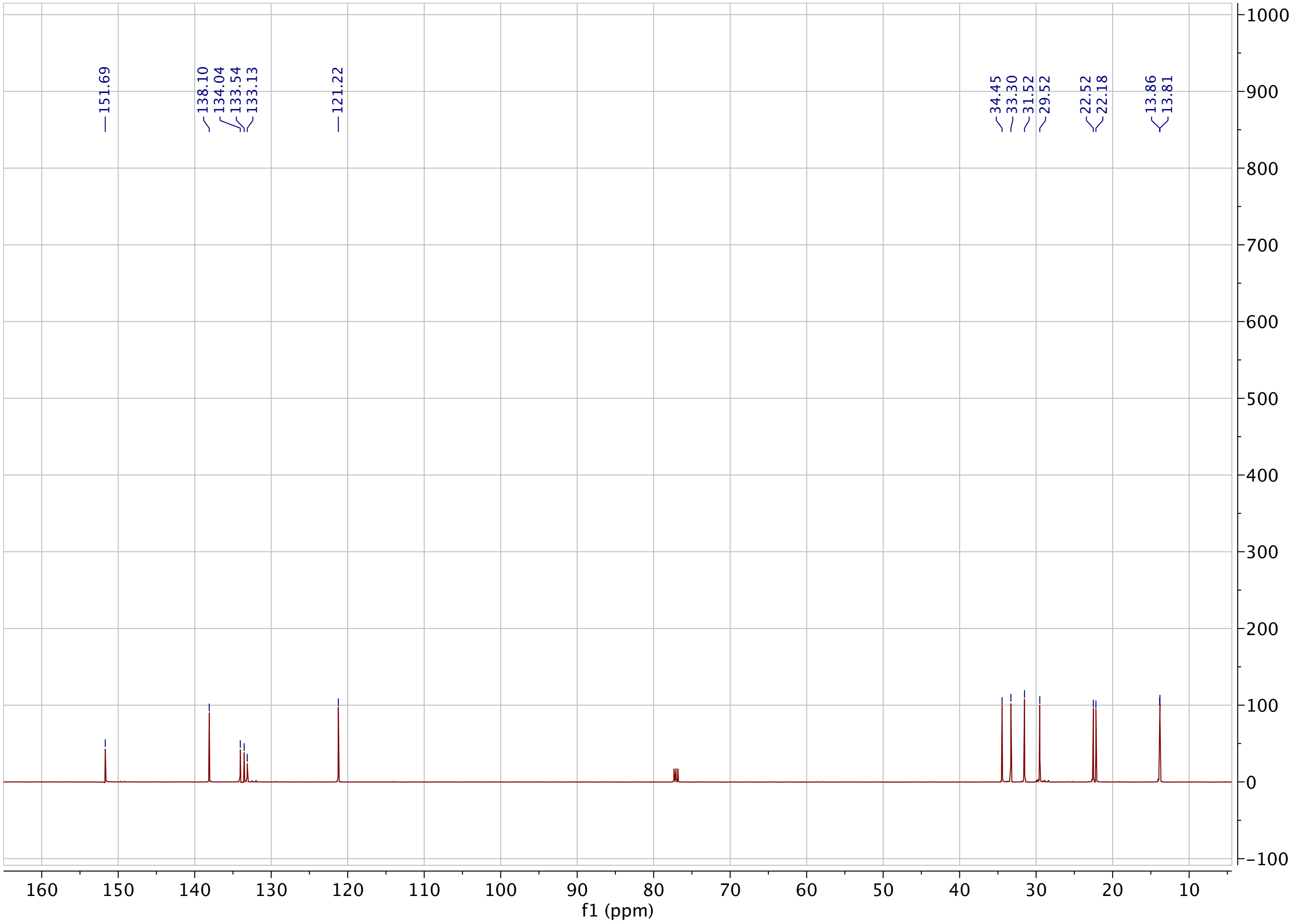
151.69
138.10
134.04
133.54
133.13
121.22
34.45
33.30
31.52
29.52
22.52
22.18
13.86
13.81
f1 (ppm)

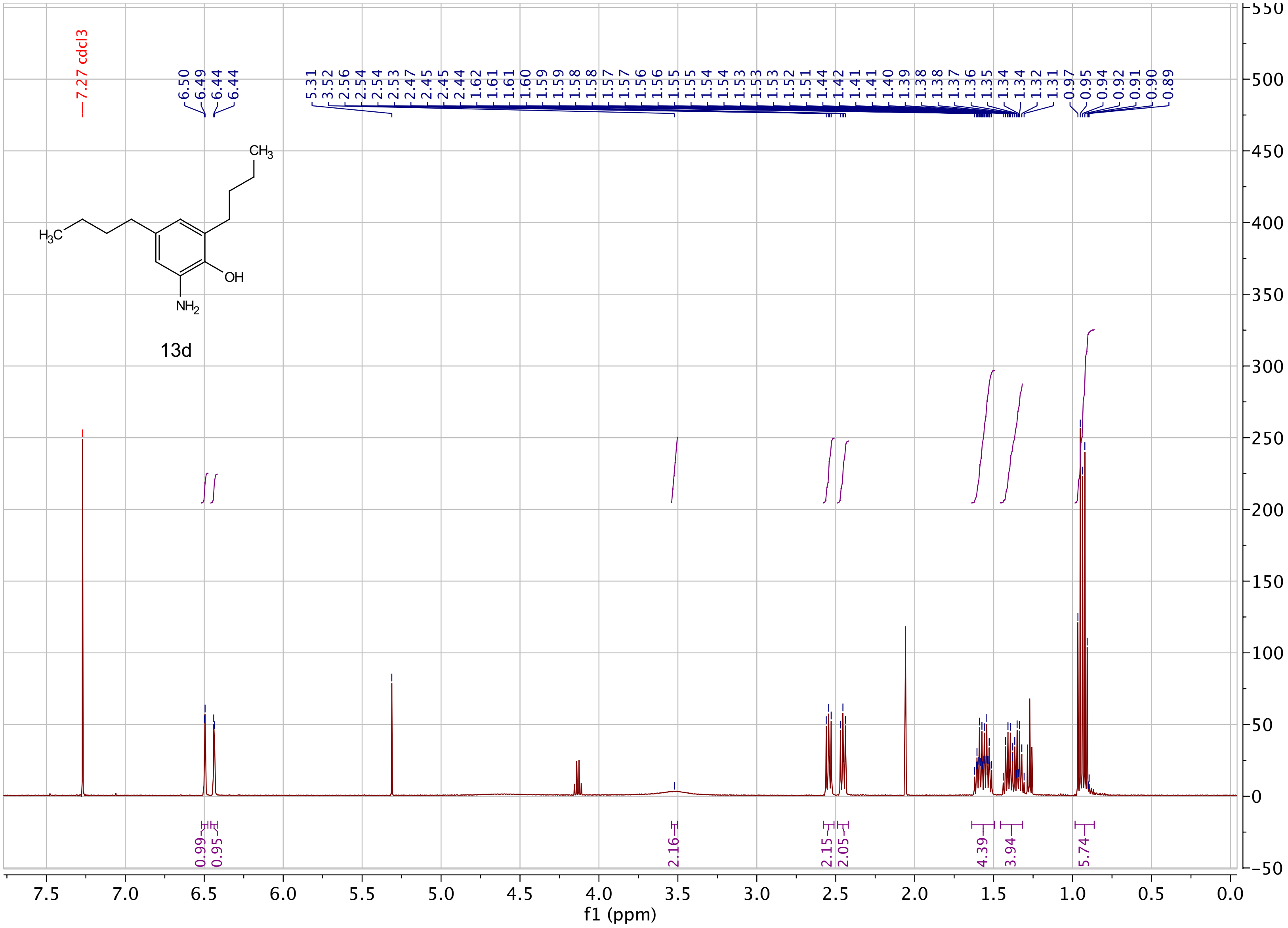

7.27 cdcl3
CH3
H3C
OH
NH2
13d
f1 (ppm)

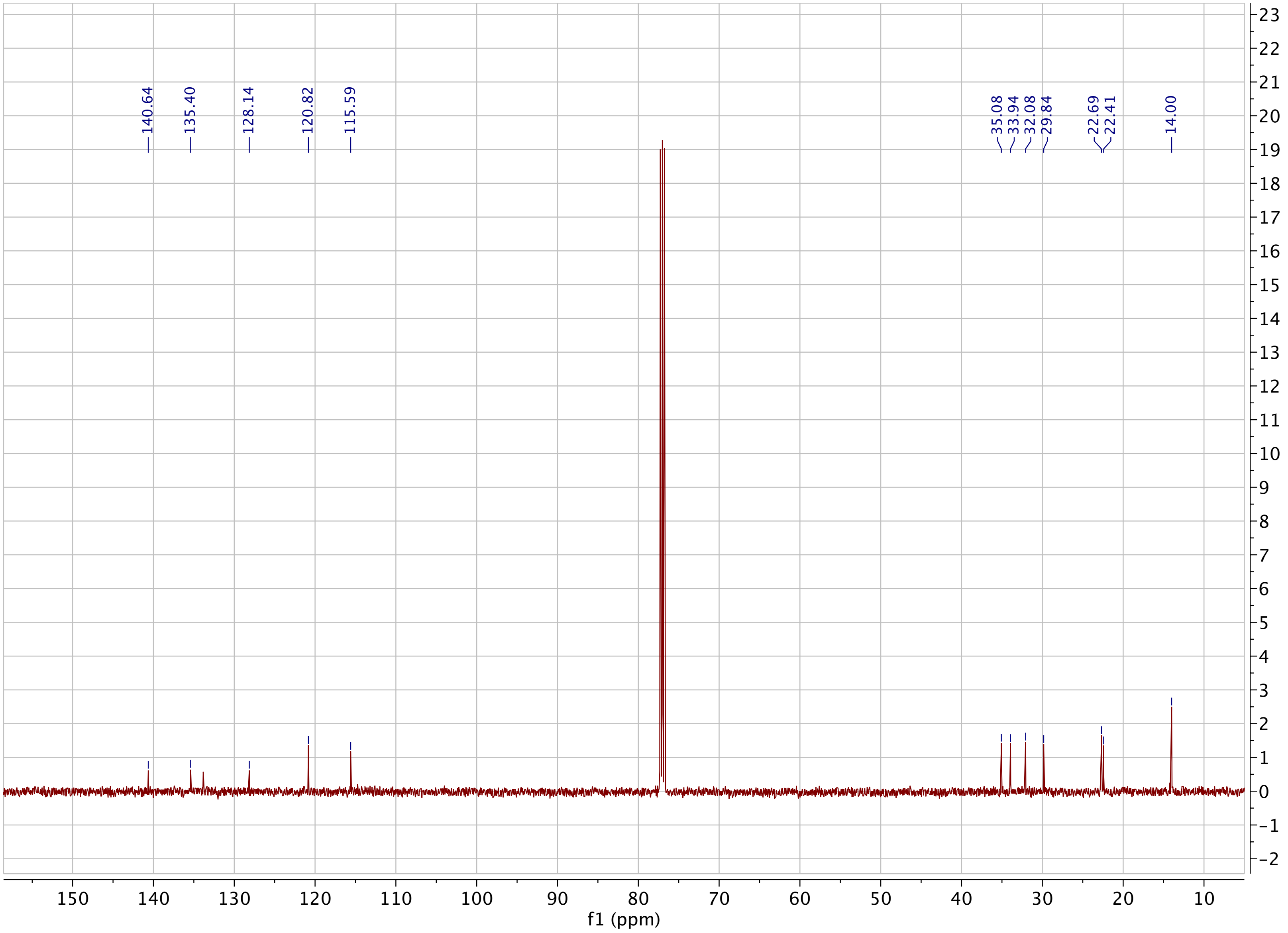

140.64
135.40
128.14
120.82
115.59
35.08
33.94
32.08
29.84
22.69
22.41
14.00
f1 (ppm)

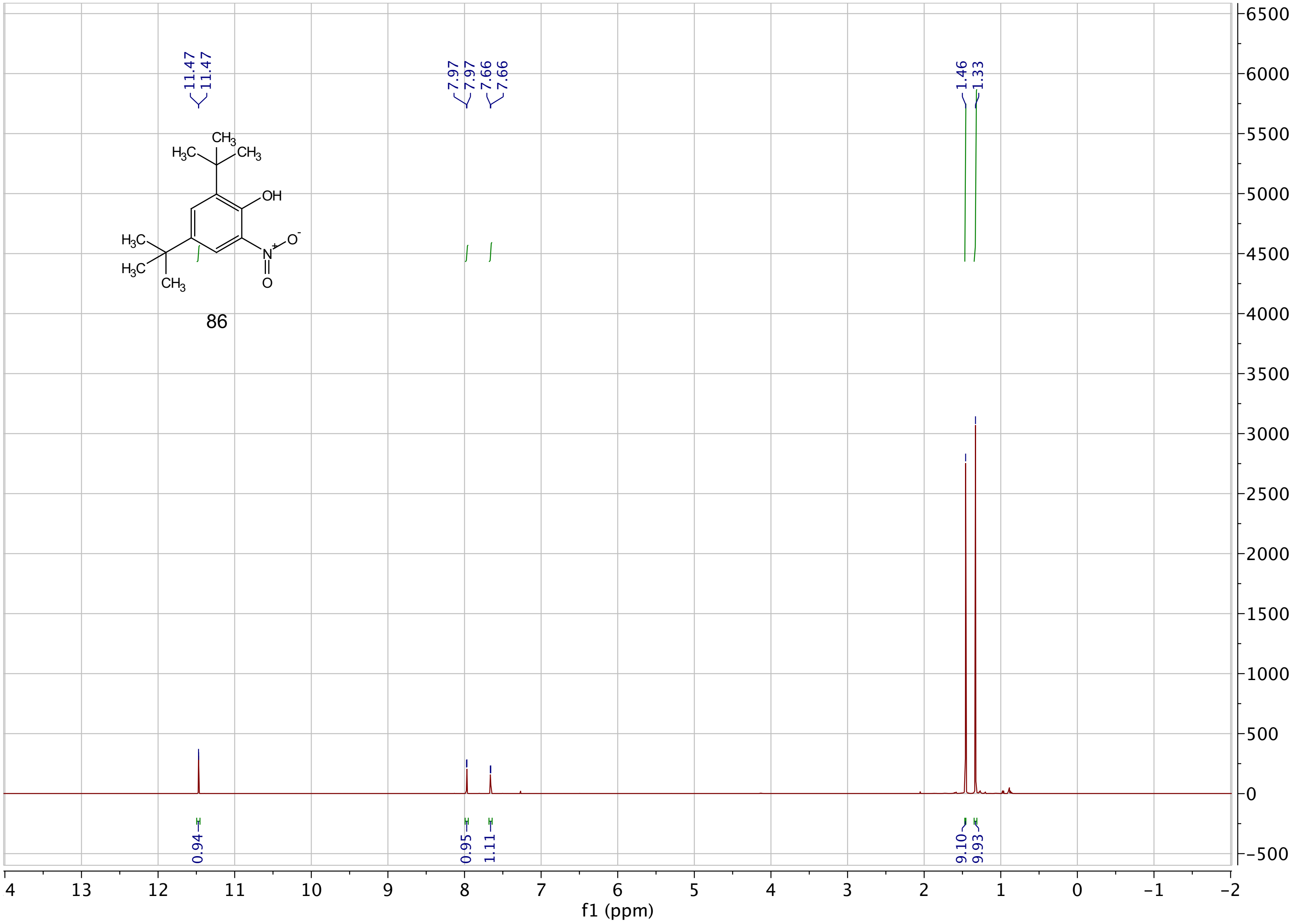

11.47
11.47
7.97
7.97
7.66
7.66
1.46
1.33
OH
86
0.94
0.95
1.11
9.10
9.93
f1 (ppm)

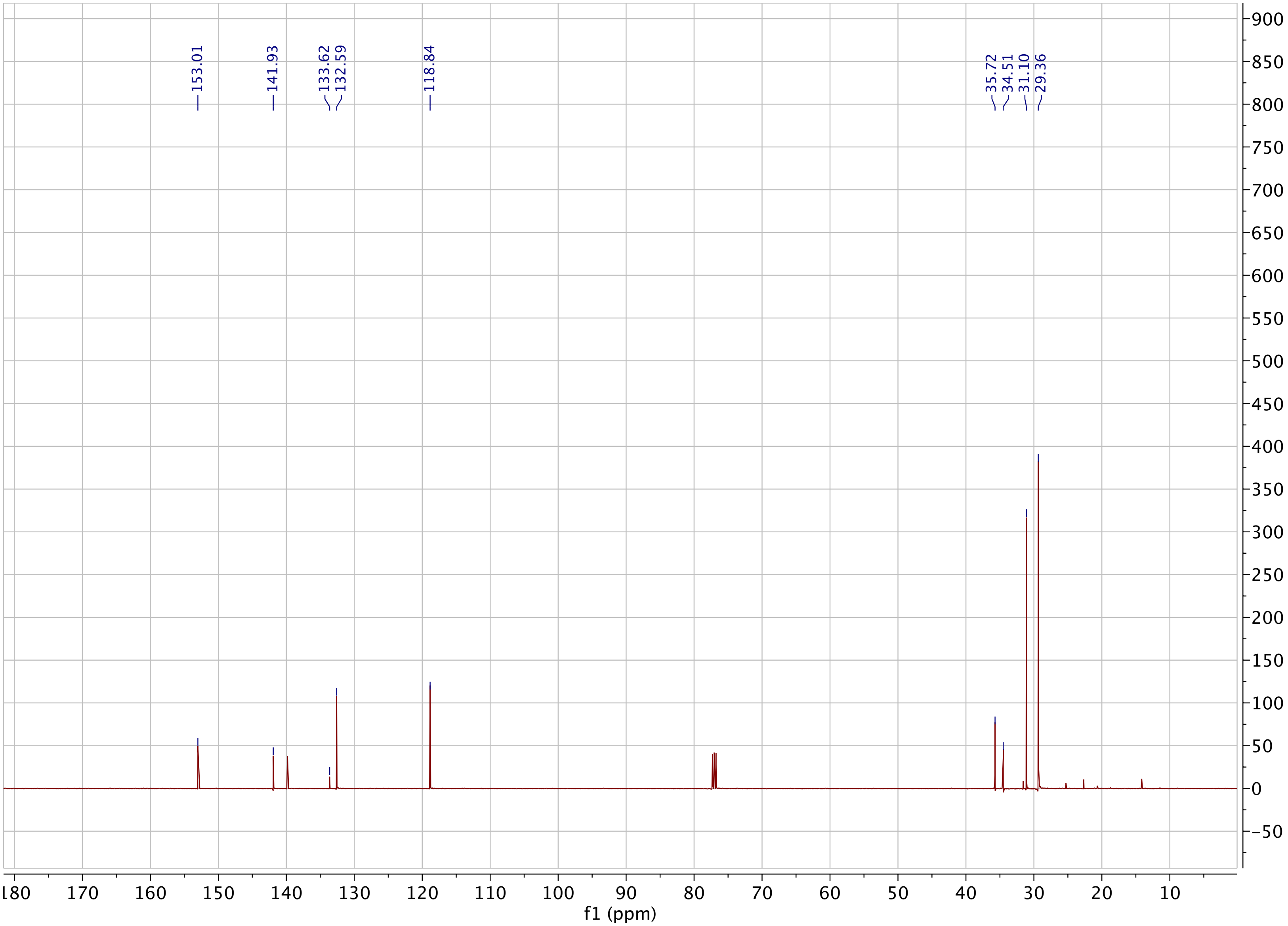
153.01
141.93
133.62
132.59
118.84
35.72
34.51
31.10
29.36
f1 (ppm)

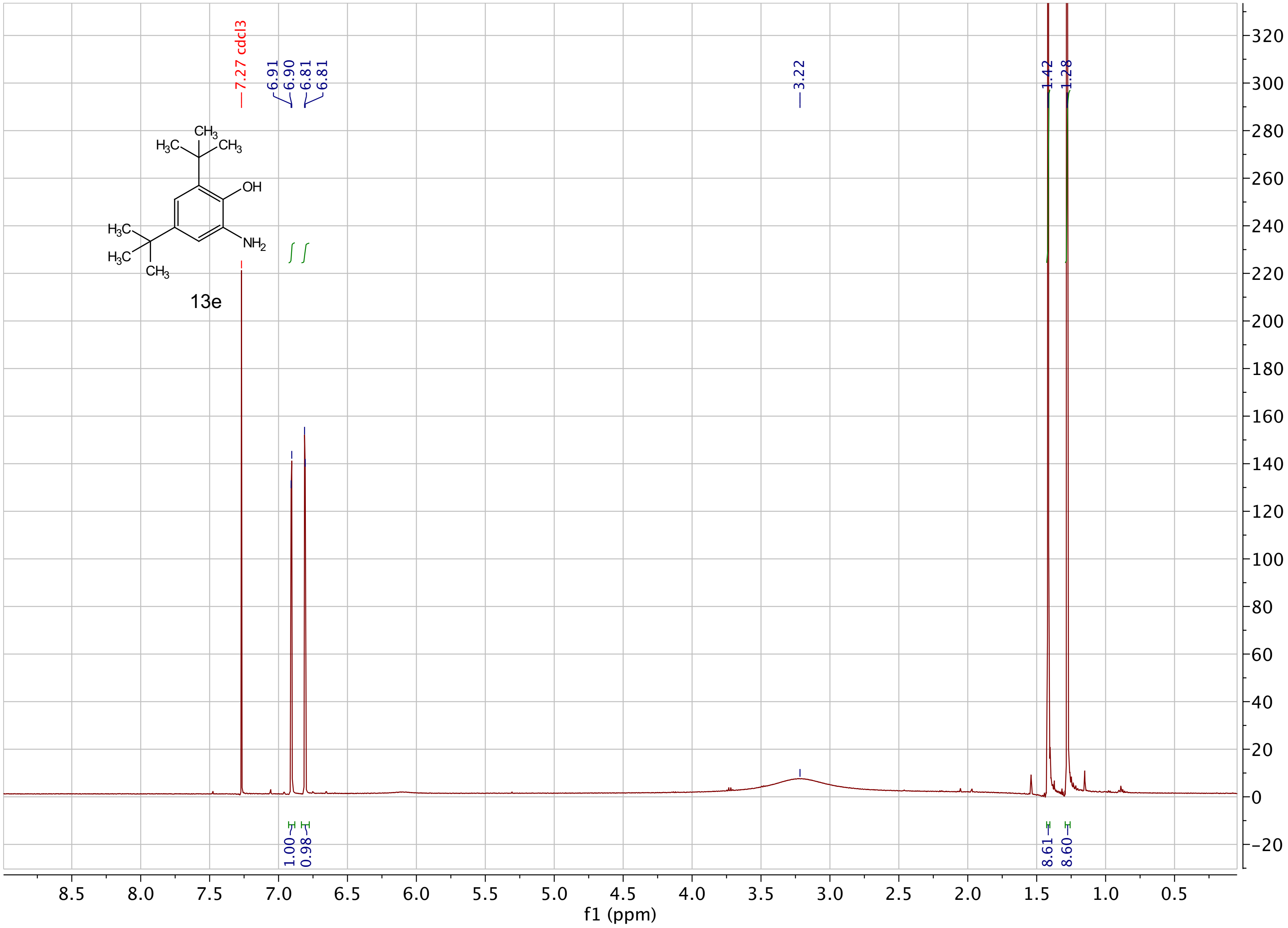

7.27 cdcl3
6.91
6.90
6.81
6.81
3.22
1.42
1.28
OH
NH2
13e
1.00
0.98
8.61
8.60
f1 (ppm)

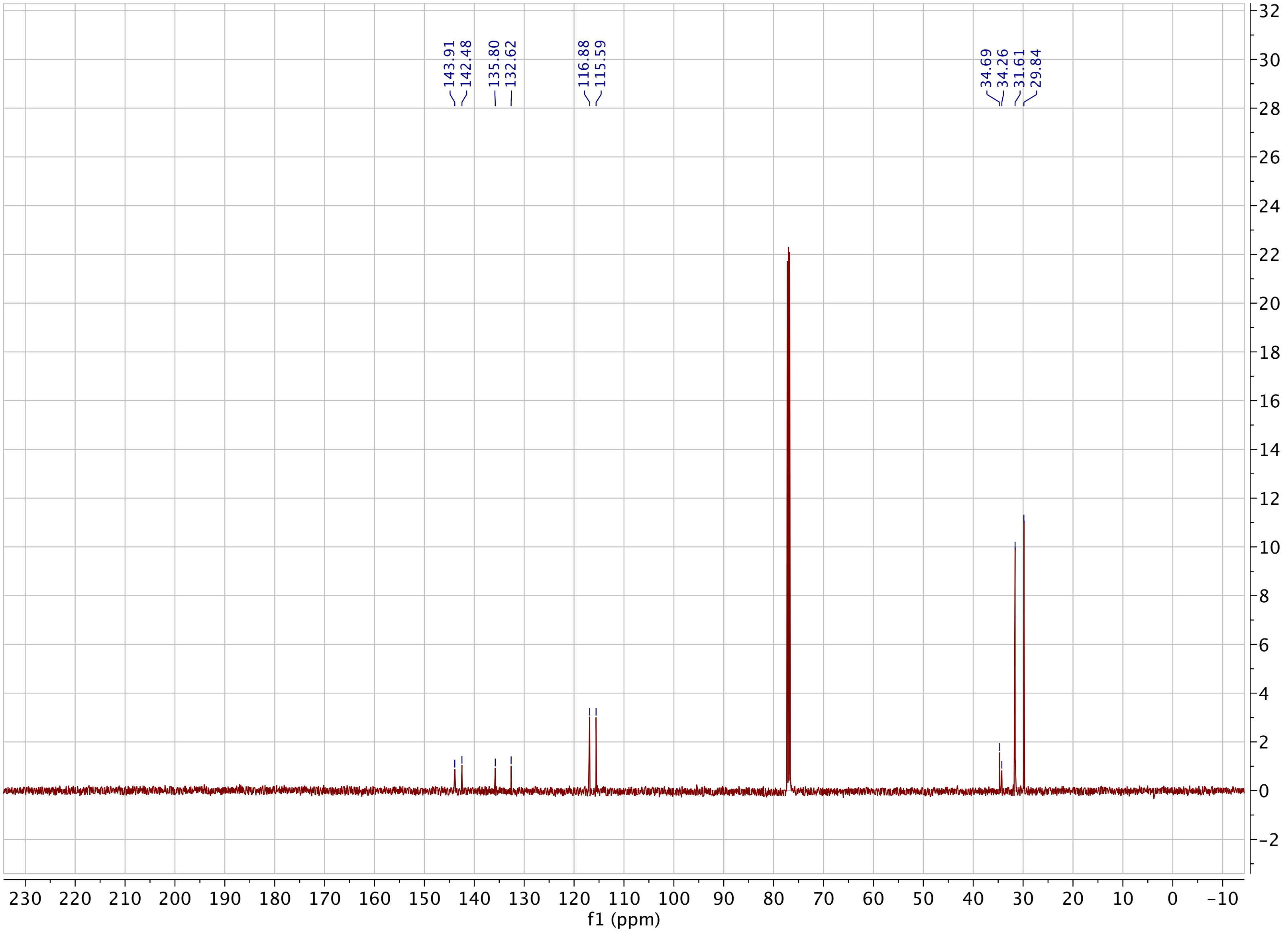
143.91
142.48
135.80
132.62
116.88
115.59
34.69
34.26
31.61
29.84
f1 (ppm)

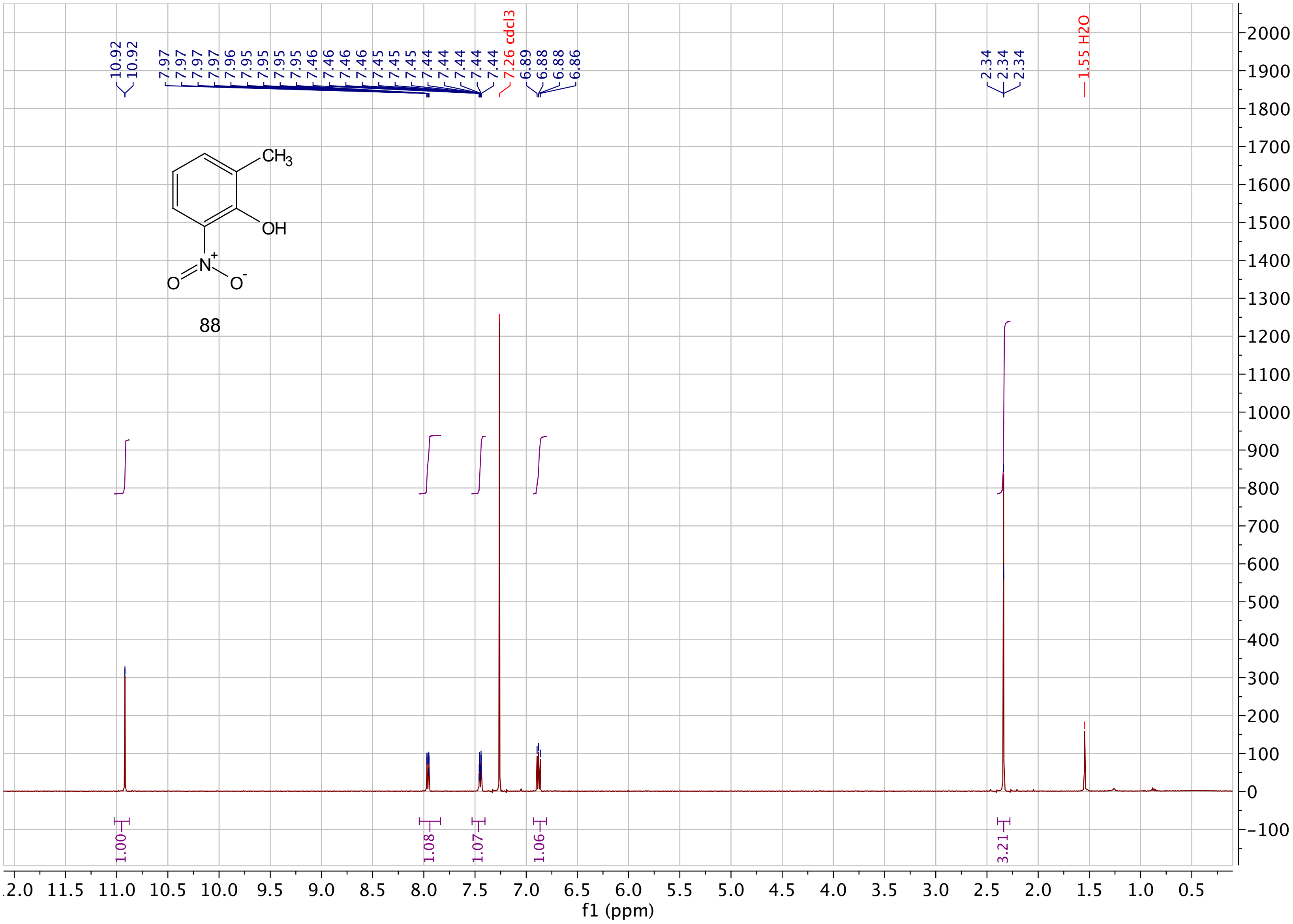

10.92
10.92
7.97
7.97
7.97
7.97
7.96
7.95
7.95
7.95
7.95
7.46
7.46
7.46
7.45
7.45
7.45
7.44
7.44
7.44
7.44
7.44
7.26 cdcl3
6.89
6.88
6.88
6.86
2.34
2.34
2.34
1.55 H2O
CH3
OH
N+
O
O-
88
1.00
1.08
1.07
1.06
3.21
f1 (ppm)

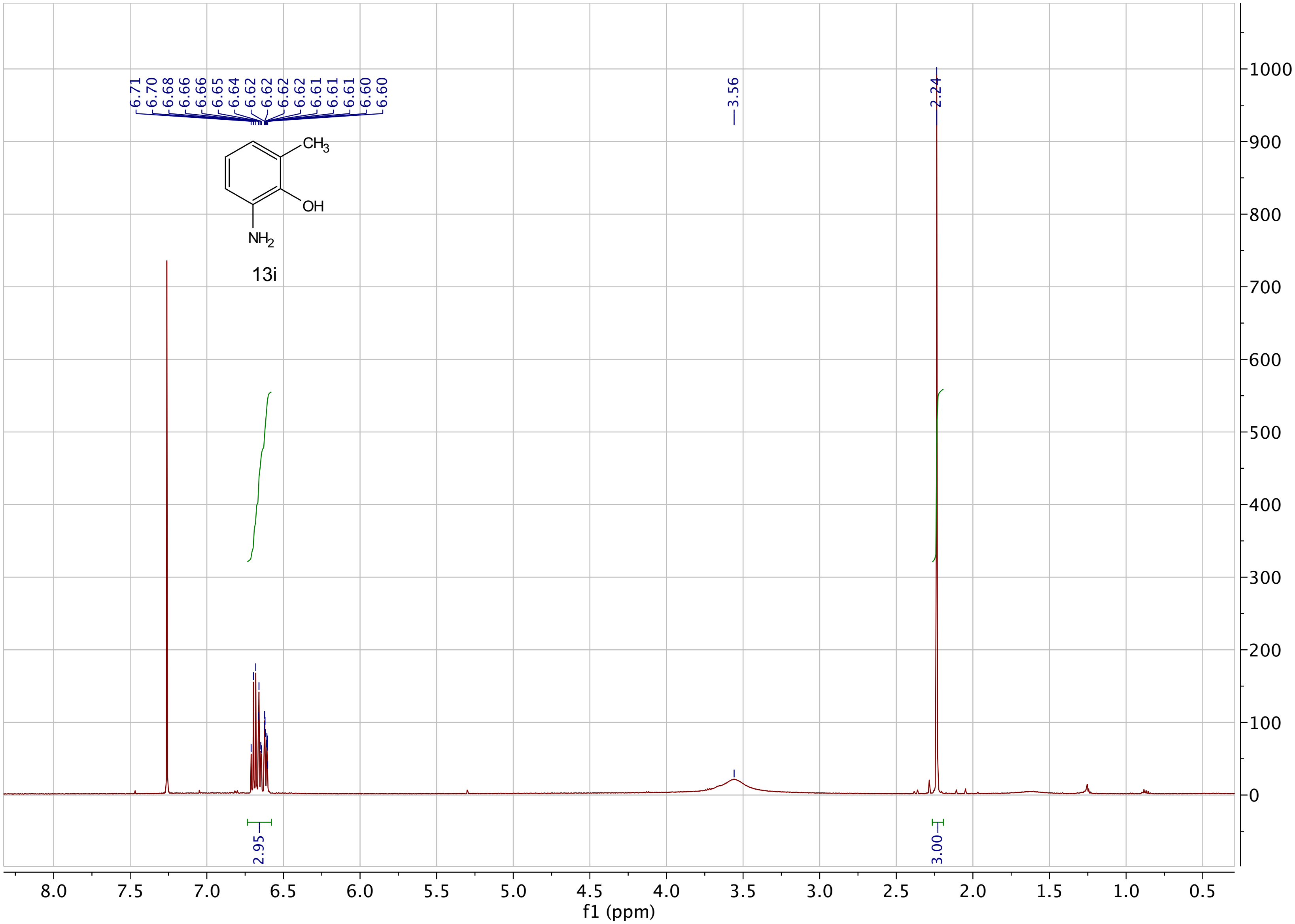
6.71
6.70
6.68
6.66
6.66
6.65
6.64
6.62
6.62
6.62
6.62
6.61
6.61
6.61
6.60
6.60
3.56
2.24
CH3
OH
NH2
13i
2.95
3.00
8.0
7.5
7.0
6.5
6.0
5.5
5.0
4.5
4.0
3.5
3.0
2.5
2.0
1.5
1.0
0.5
f1 (ppm)
1000
900
800
700
600
500
400
300
200
100
0

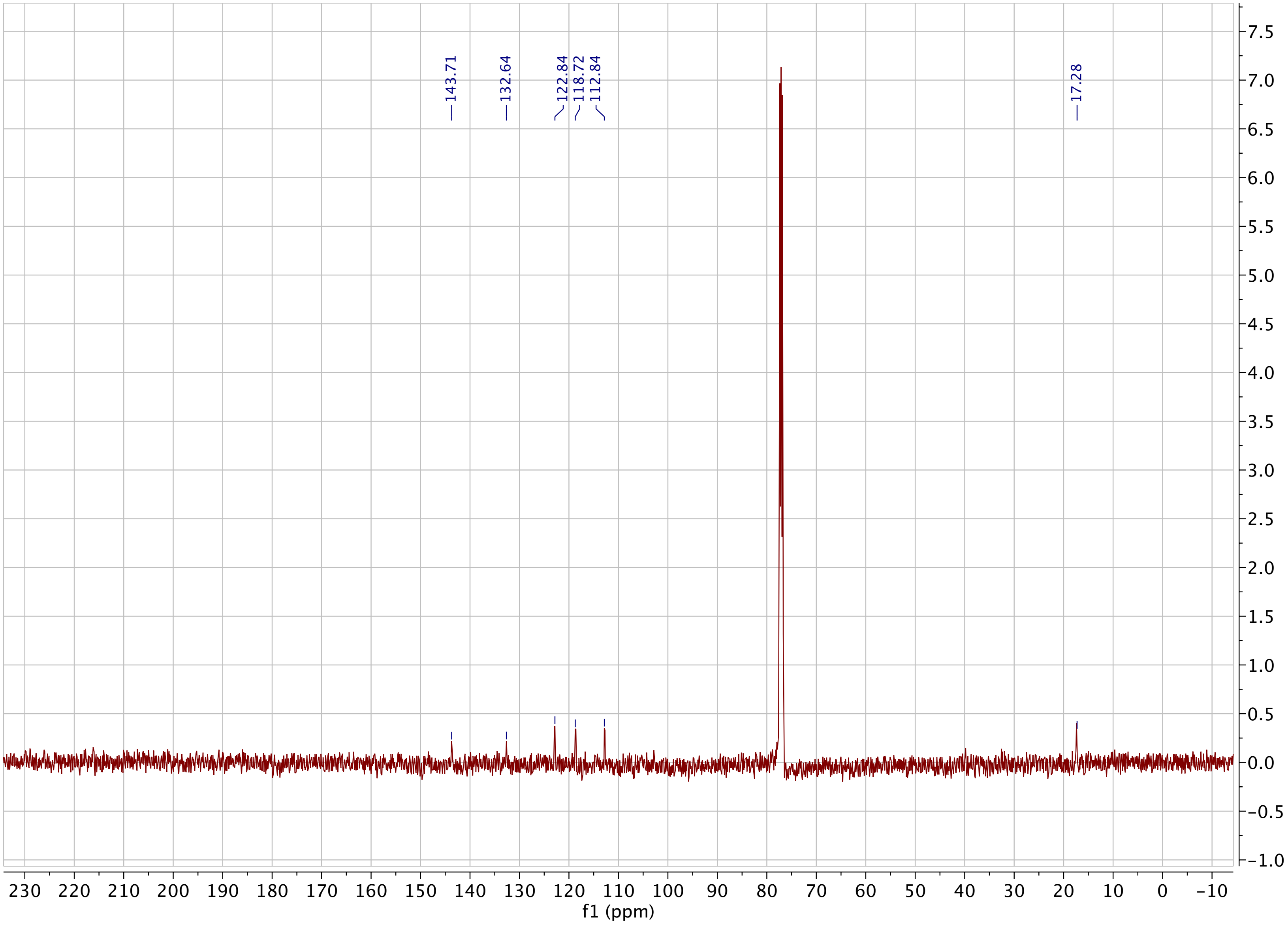

143.71
132.64
122.84
118.72
112.84
17.28
f1 (ppm)

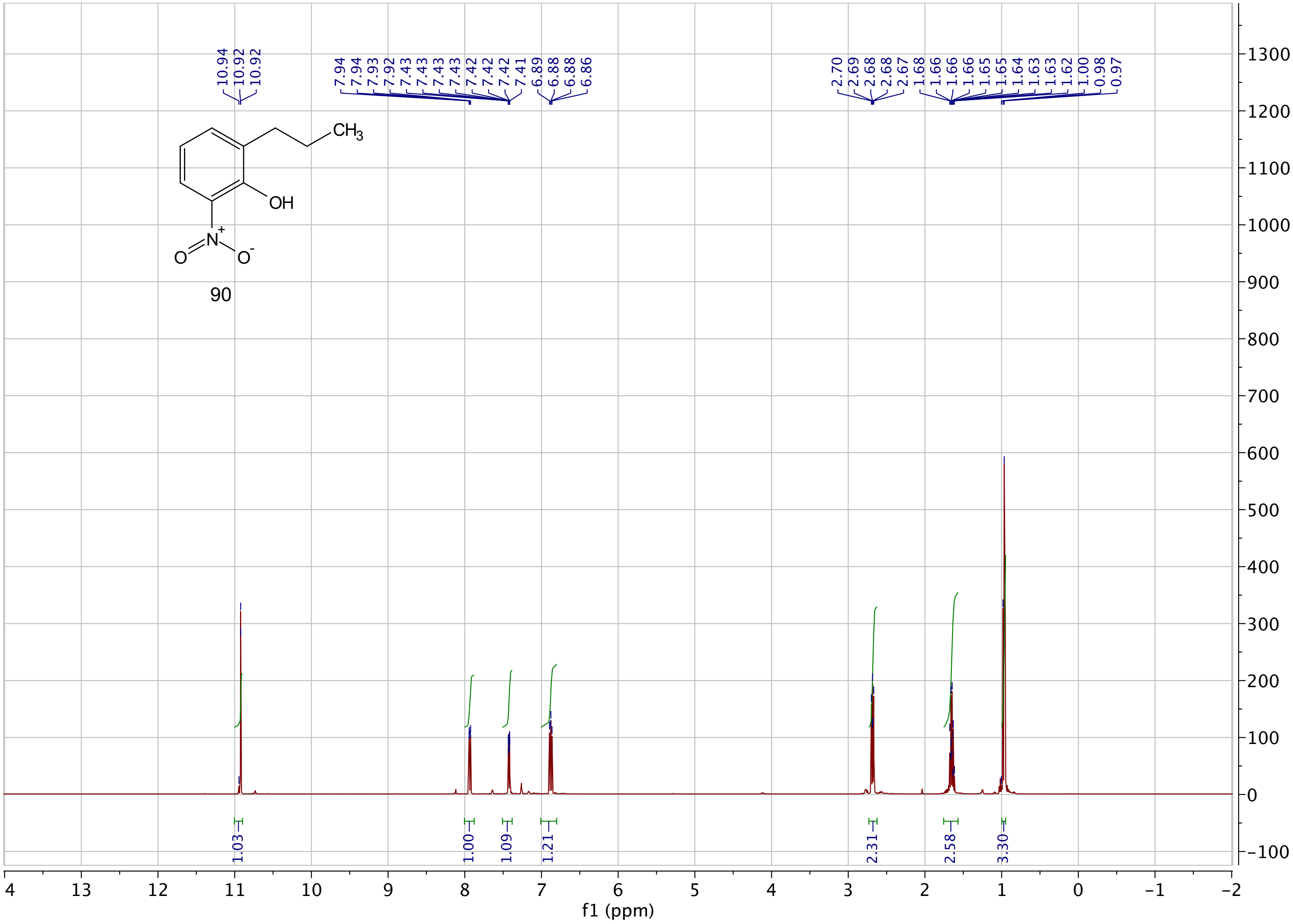
10.94
10.92
10.92
7.94
7.94
7.93
7.92
7.43
7.43
7.43
7.42
7.42
7.42
7.41
6.89
6.88
6.88
6.86
2.70
2.69
2.68
2.68
2.67
1.68
1.66
1.66
1.66
1.65
1.65
1.64
1.63
1.63
1.62
1.00
0.98
0.97
CH3
OH
N+
O
O-
90
1.03
1.00
1.09
1.21
2.31
2.58
3.30
f1 (ppm)

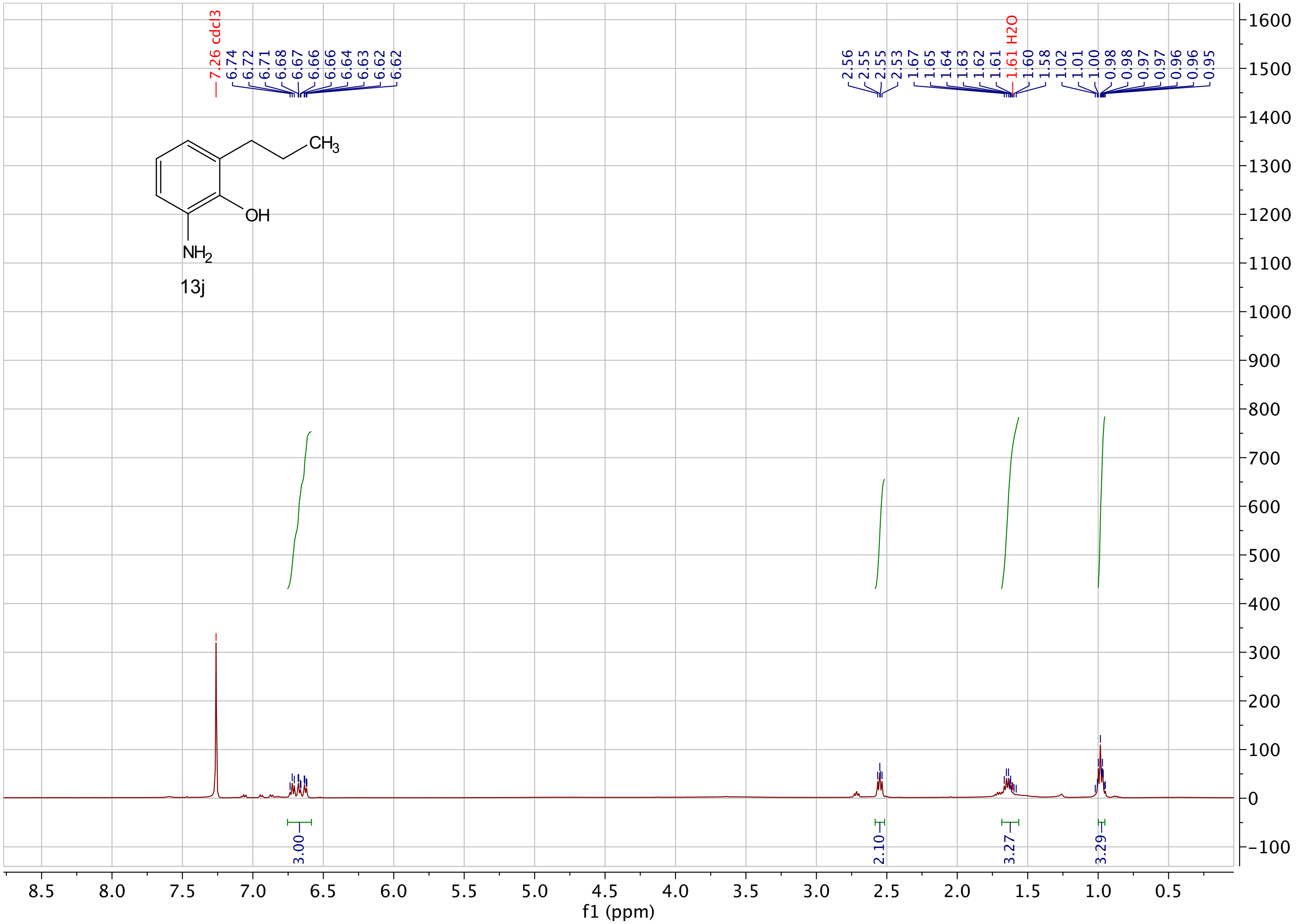
7.26 cdcl3
6.74
6.72
6.71
6.68
6.67
6.66
6.66
6.64
6.63
6.62
6.62
2.56
2.55
2.55
2.53
1.67
1.65
1.64
1.63
1.62
1.61
1.61 H2O
1.60
1.58
1.02
1.01
1.00
0.98
0.98
0.97
0.97
0.96
0.96
0.95
CH3
OH
NH2
13j
3.00
2.10
3.27
3.29
f1 (ppm)

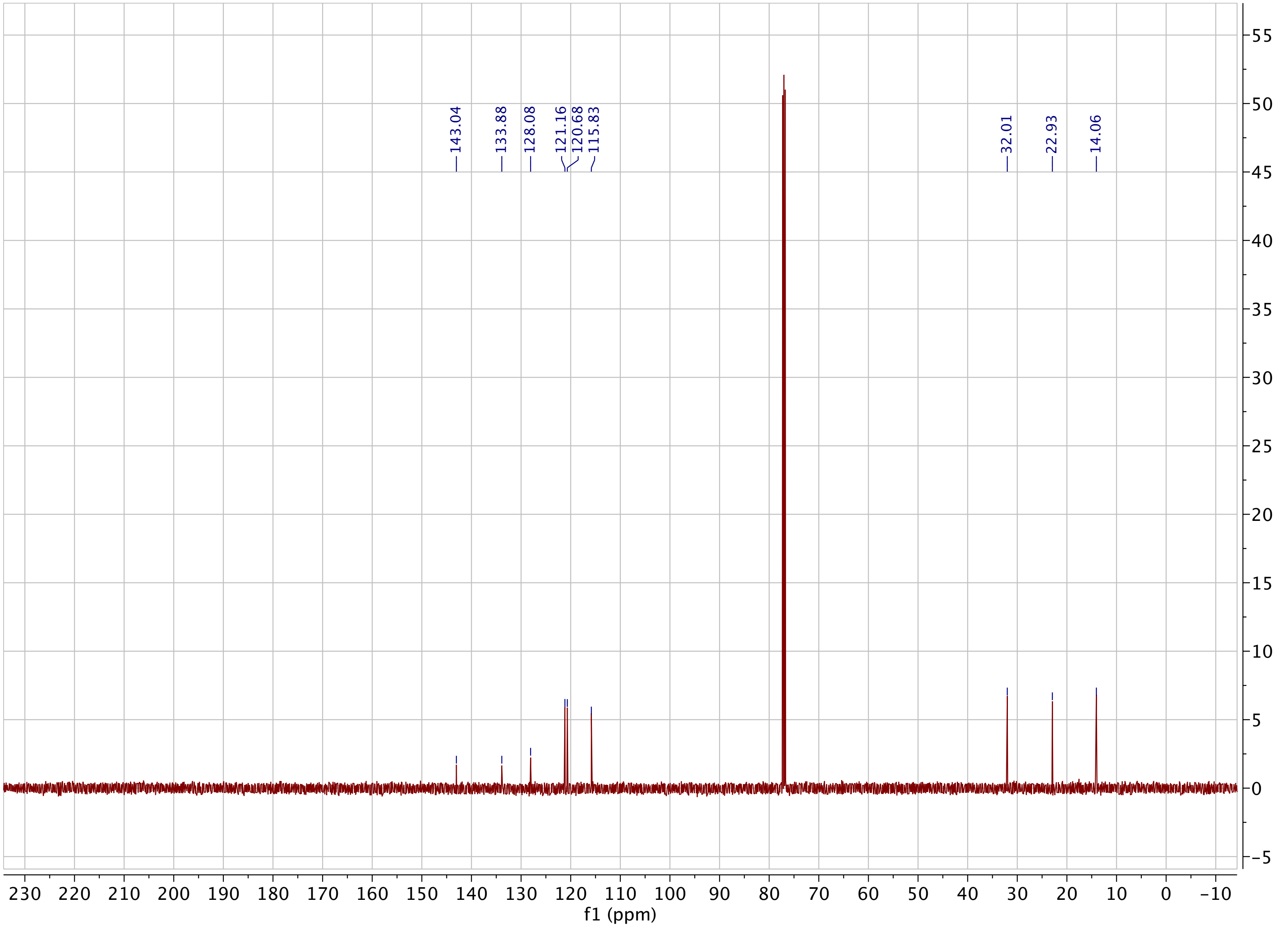
143.04
133.88
128.08
121.16
120.68
115.83
32.01
22.93
14.06
f1 (ppm)

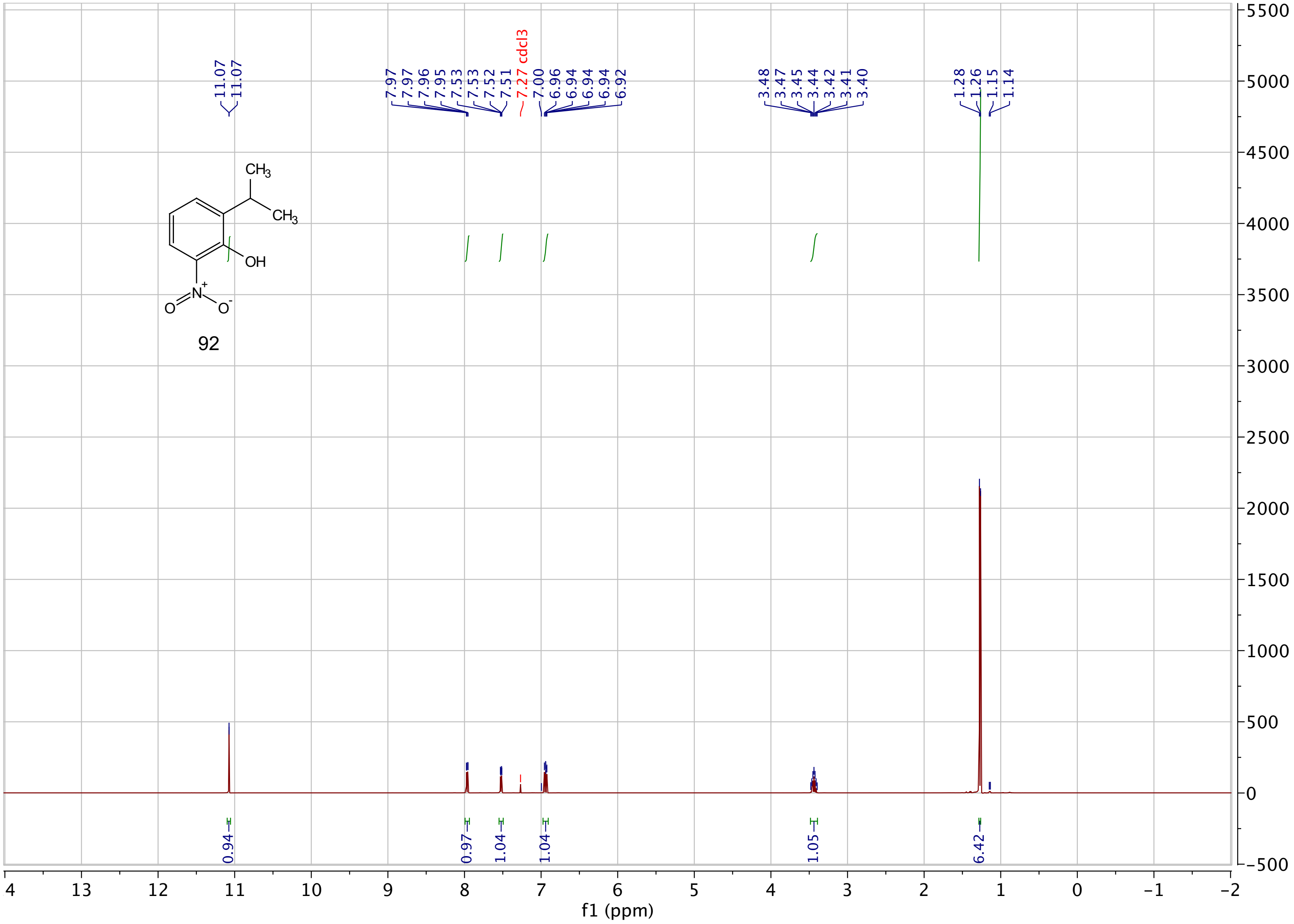

11.07
11.07
7.97
7.97
7.96
7.95
7.53
7.53
7.52
7.51
7.27 cdcl3
7.00
6.96
6.94
6.94
6.94
6.92
3.48
3.47
3.45
3.44
3.42
3.41
3.40
1.28
1.26
1.15
1.14
CH3
CH3
OH
N+
O
O-
92
0.94
0.97
1.04
1.04
1.05
6.42
f1 (ppm)

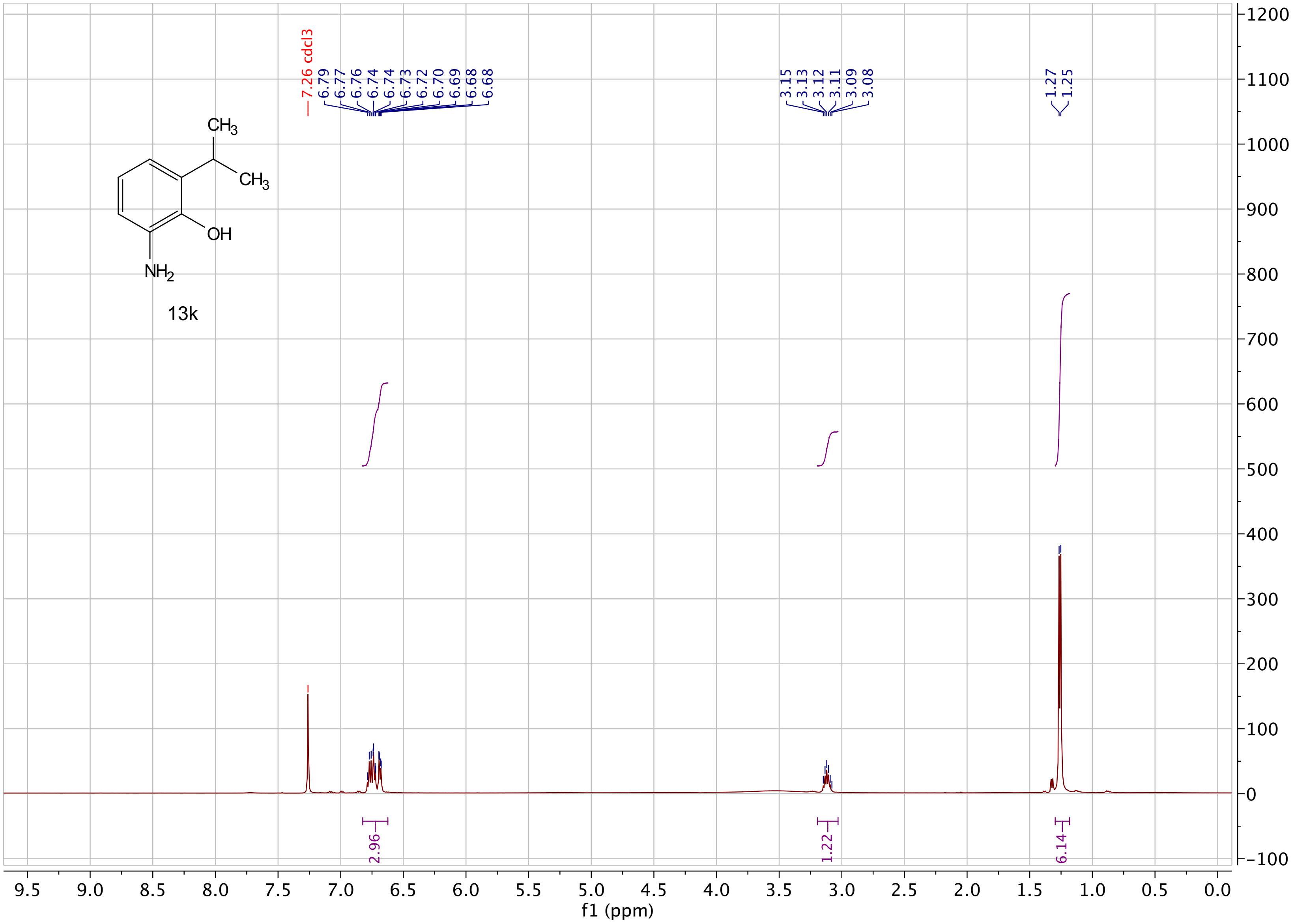
7.26 cdcl3
6.79
6.77
6.76
6.74
6.74
6.73
6.72
6.70
6.69
6.68
6.68
3.15
3.13
3.12
3.11
3.09
3.08
1.27
1.25
2.96
1.22
6.14
f1 (ppm)

13k

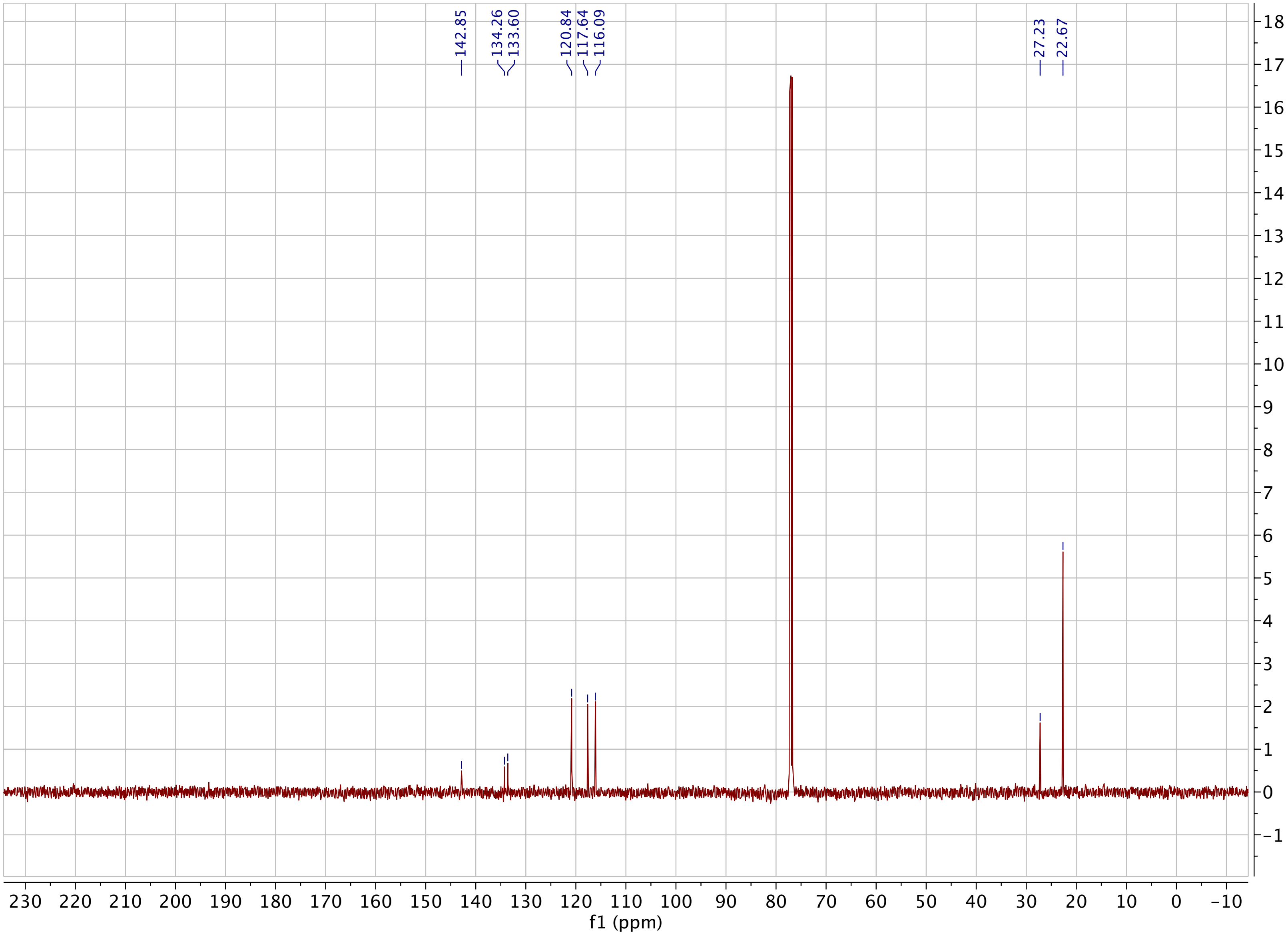

142.85
134.26
133.60
120.84
117.64
116.09
27.23
22.67
230 220 210 200 190 180 170 160 150 140 130 120 110 100 90 80 70 60 50 40 30 20 10 0 −10
f1 (ppm)
18 17 16 15 14 13 12 11 10 9 8 7 6 5 4 3 2 1 0 −1

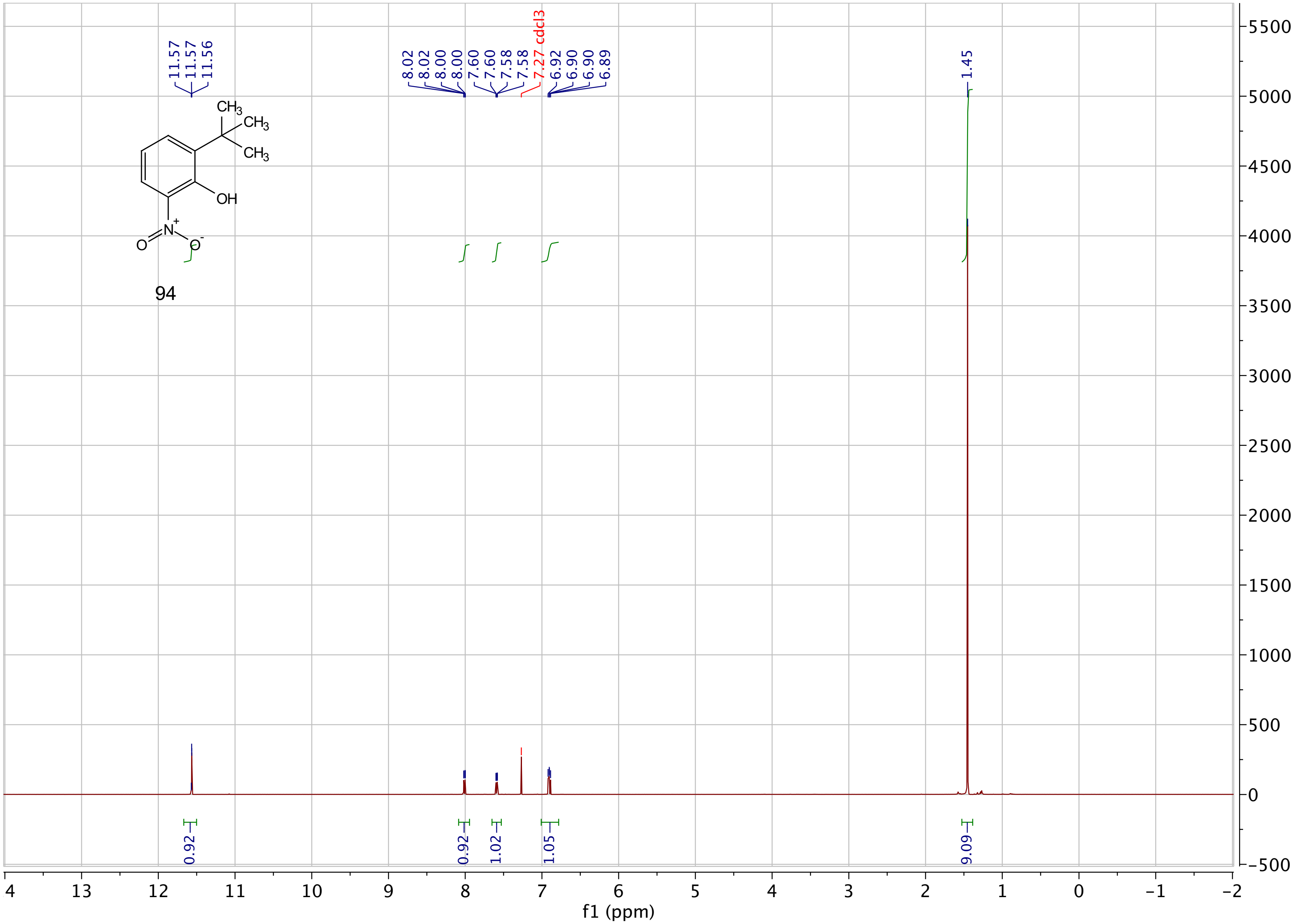
11.57
11.57
11.56
8.02
8.02
8.00
8.00
7.60
7.60
7.58
7.58
7.27 cdcl3
6.92
6.90
6.90
6.89
1.45
CH3
CH3
CH3
OH
N+
O
O-
94
0.92
0.92
1.02
1.05
9.09
f1 (ppm)

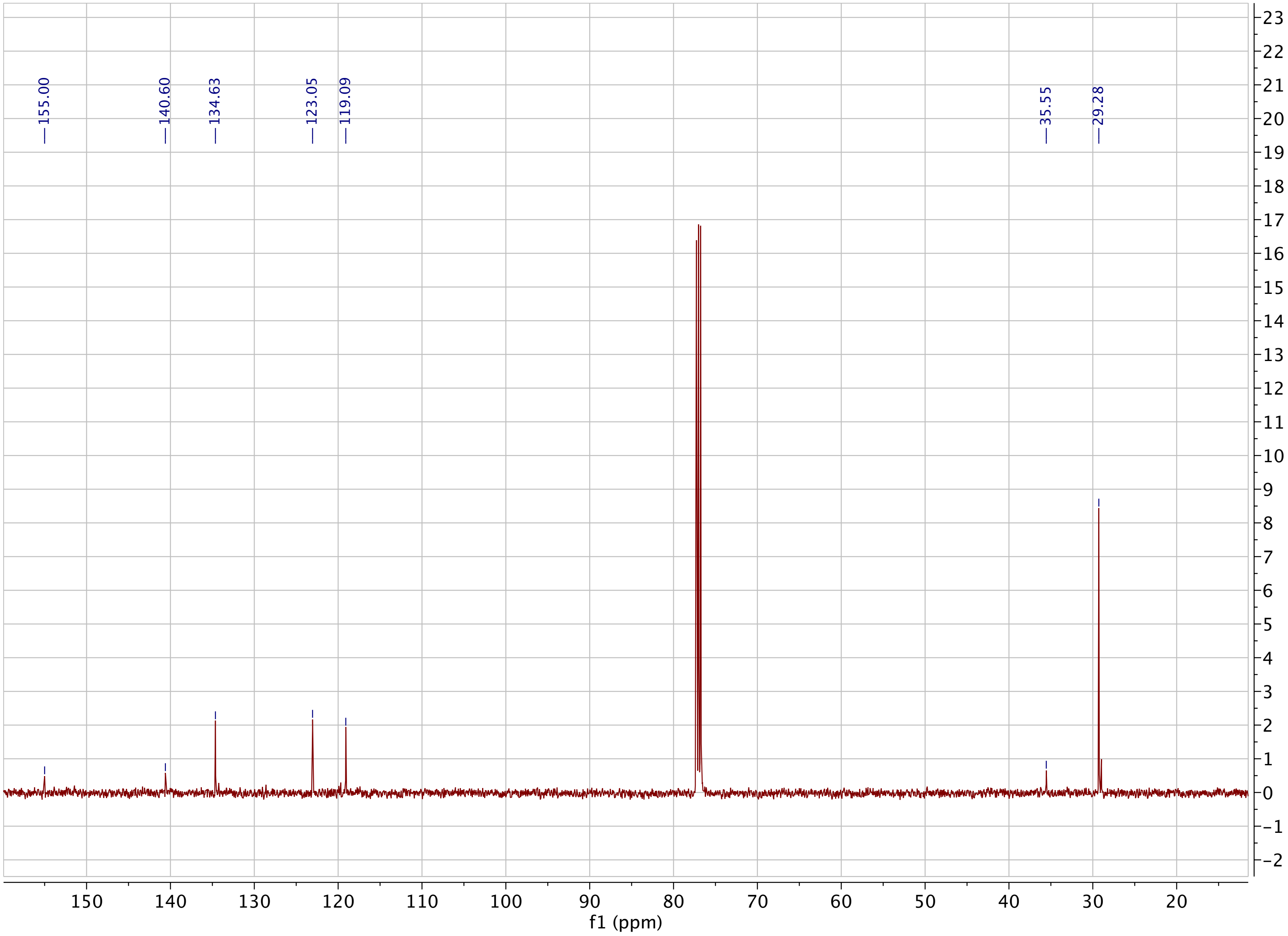

155.00
140.60
134.63
123.05
119.09
35.55
29.28
f1 (ppm)

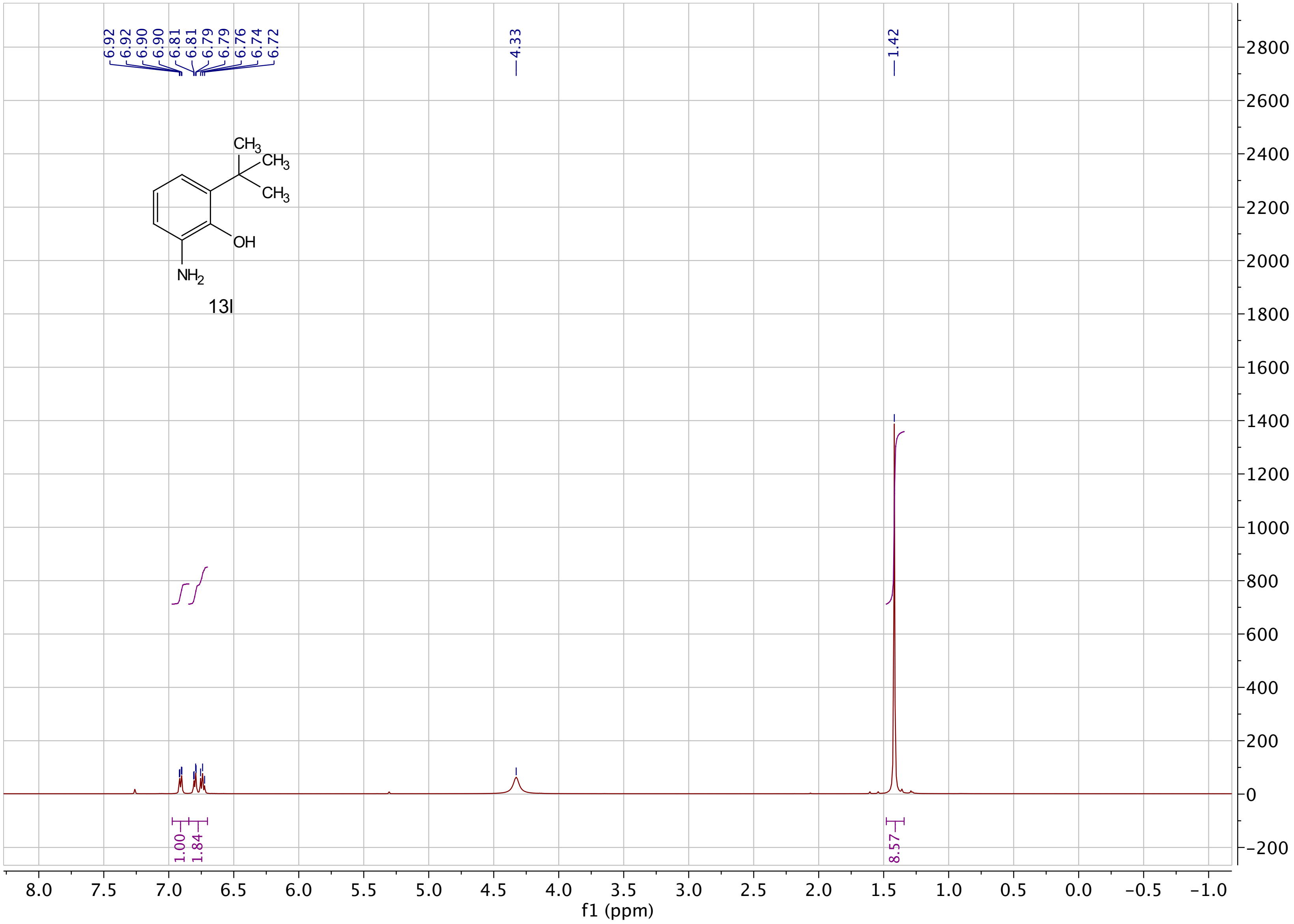
6.92
6.92
6.90
6.90
6.81
6.81
6.79
6.79
6.76
6.74
6.72
4.33
1.42
CH3
CH3
CH3
OH
NH2
13l
1.00
1.84
8.57
f1 (ppm)

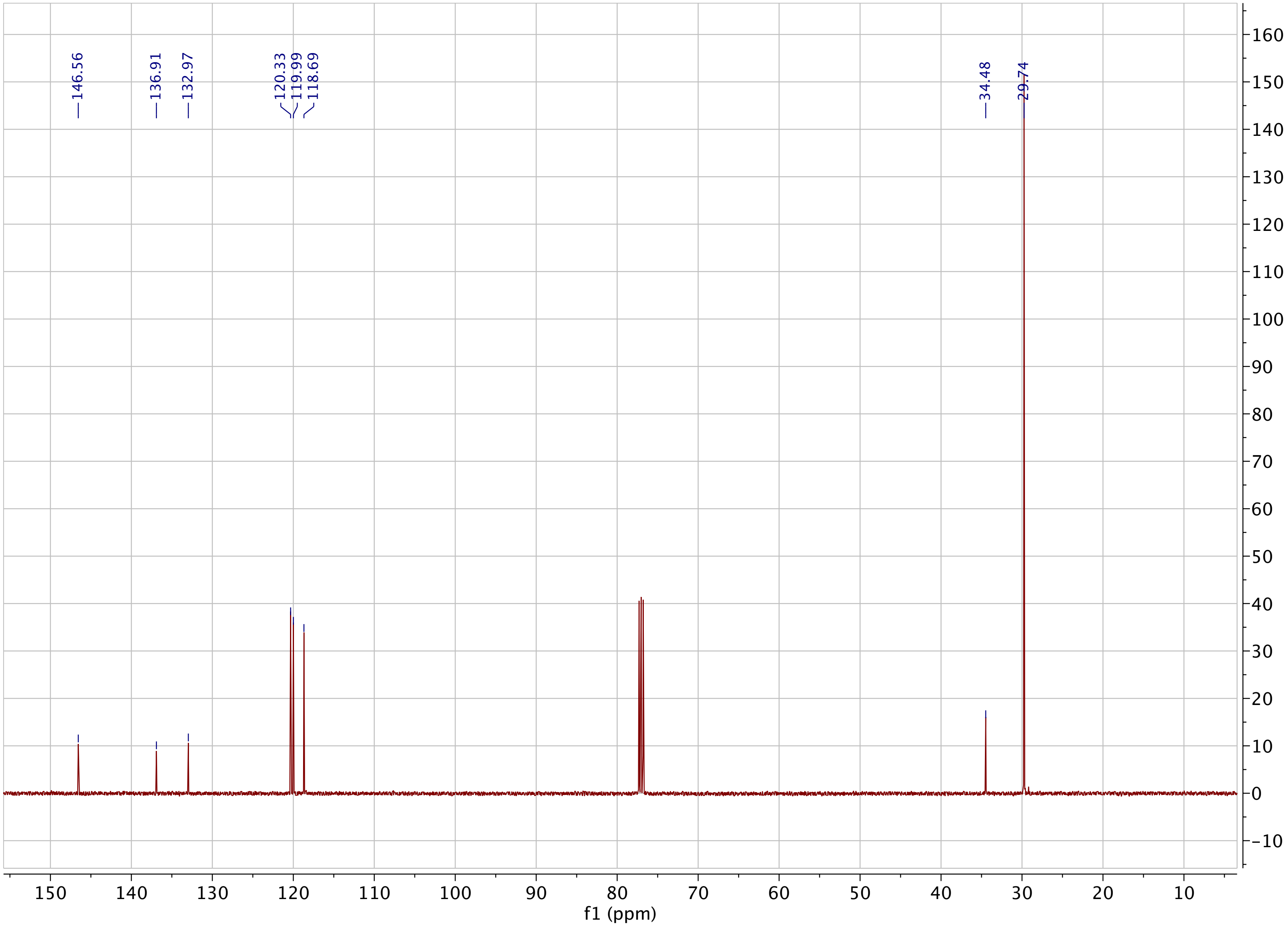
146.56
136.91
132.97
120.33
119.99
118.69
34.48
29.74
f1 (ppm)

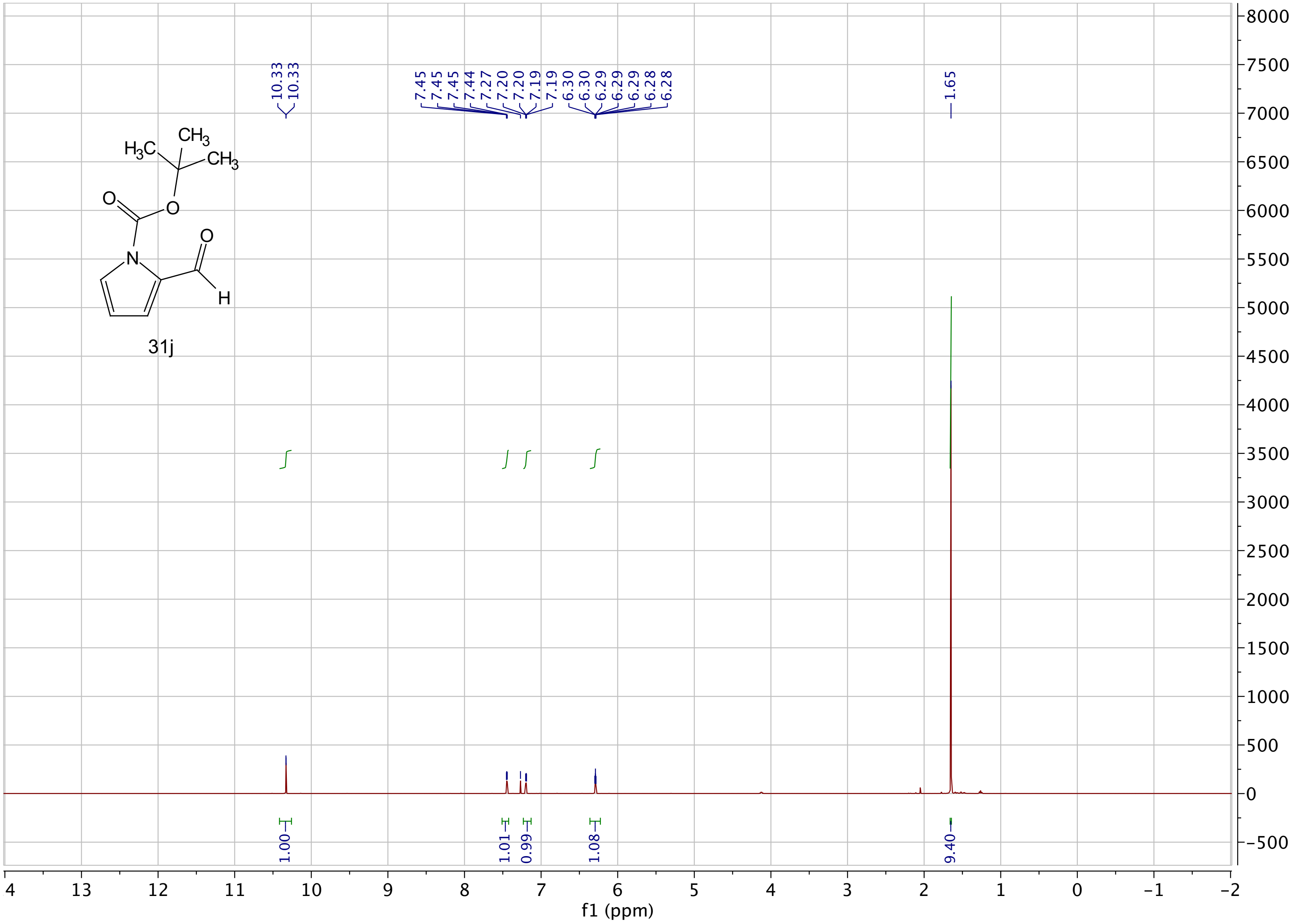

31j
10.33
10.33
7.45
7.45
7.45
7.44
7.27
7.20
7.20
7.19
7.19
6.30
6.30
6.29
6.29
6.29
6.28
6.28
1.65
1.00
1.01
0.99
1.08
9.40
f1 (ppm)

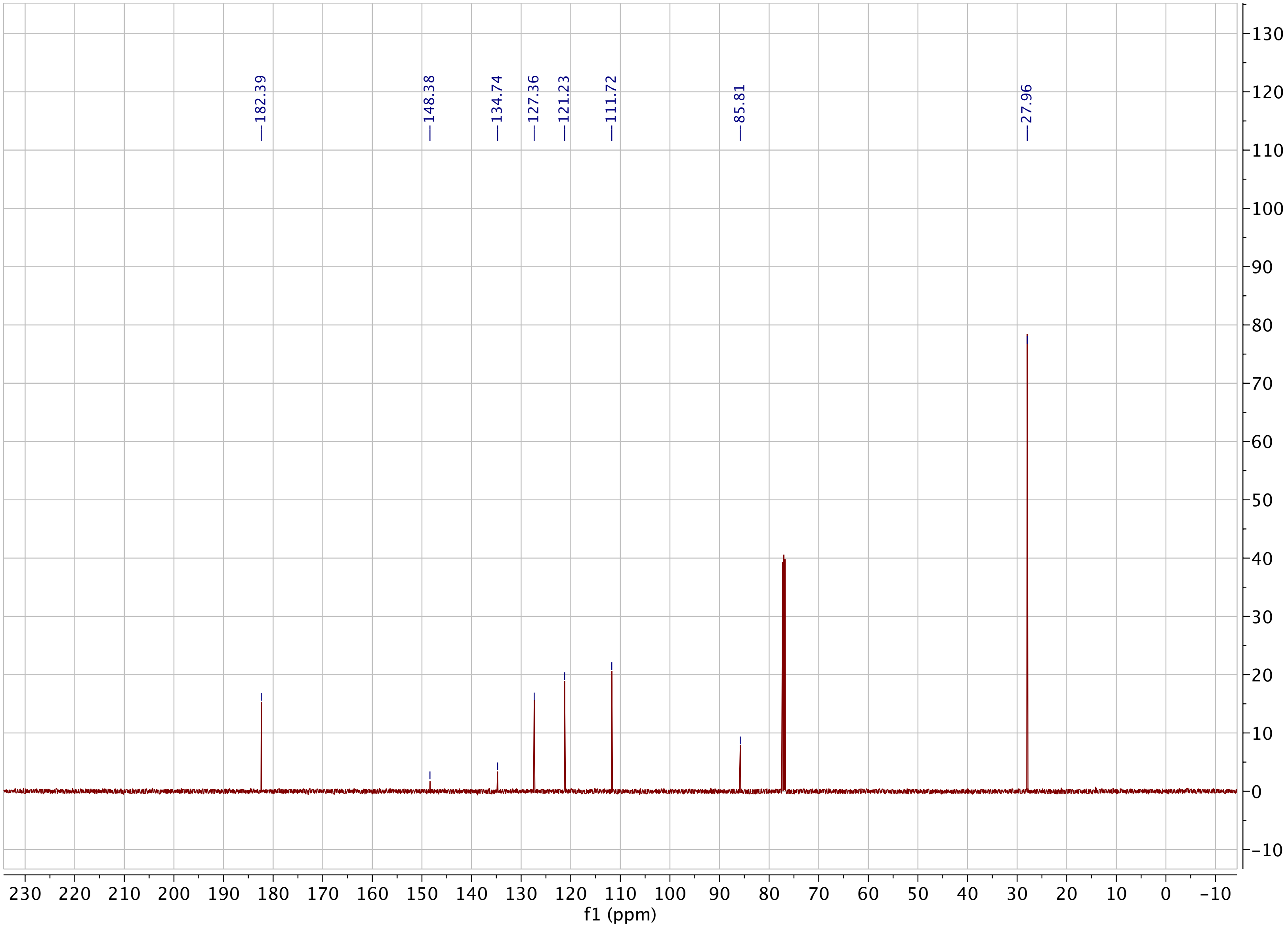
182.39
148.38
134.74
127.36
121.23
111.72
85.81
27.96
f1 (ppm)

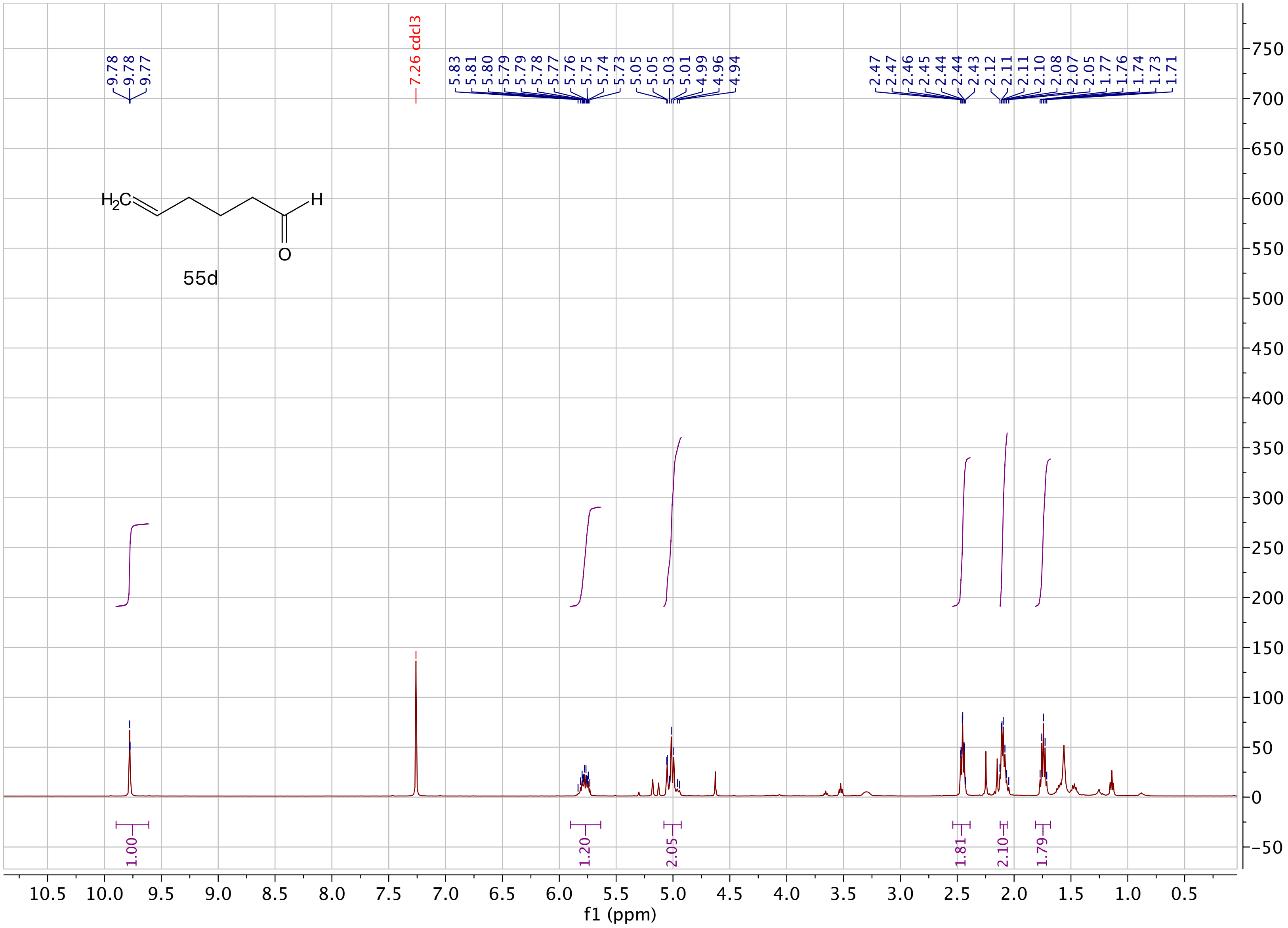

9.78
9.78
9.77
7.26 cdcl3
5.83
5.81
5.80
5.79
5.79
5.78
5.77
5.76
5.75
5.74
5.73
5.05
5.05
5.03
5.01
4.99
4.96
4.94
2.47
2.47
2.46
2.45
2.44
2.44
2.43
2.12
2.11
2.11
2.10
2.08
2.07
2.05
1.77
1.76
1.74
1.73
1.71
H2C
H
O
55d
1.00
1.20
2.05
1.81
2.10
1.79
f1 (ppm)

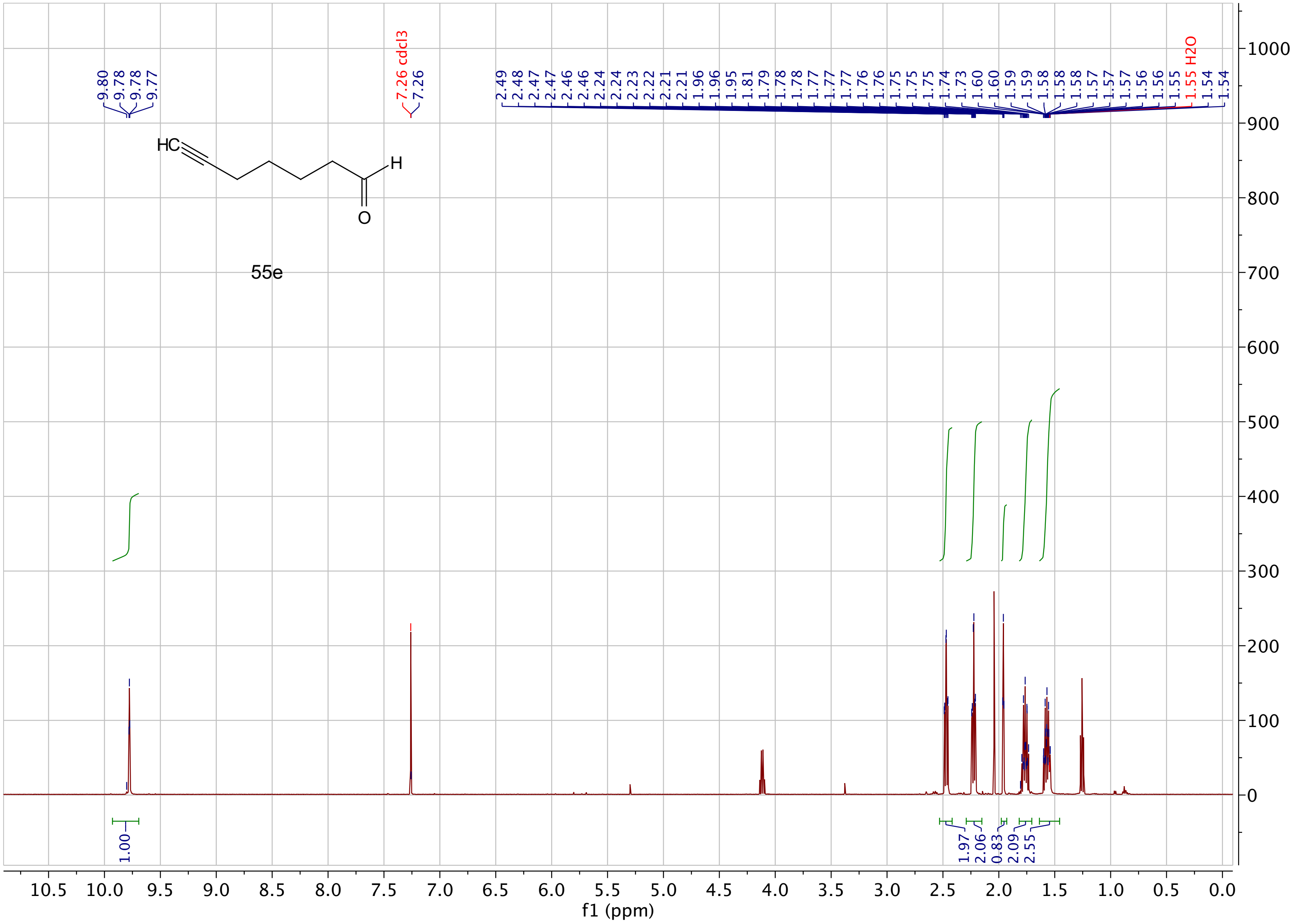
9.80
9.78
9.78
9.77
7.26 cdcl3
7.26
2.49
2.48
2.47
2.47
2.46
2.46
2.24
2.24
2.23
2.22
2.22
2.21
2.21
1.96
1.96
1.95
1.81
1.79
1.78
1.78
1.77
1.77
1.77
1.76
1.76
1.75
1.75
1.75
1.74
1.73
1.60
1.60
1.59
1.59
1.58
1.58
1.58
1.57
1.57
1.57
1.56
1.56
1.55
1.55 H2O
1.54
1.54
HC
H
O
55e
1.00
1.97
2.06
0.83
2.09
2.55
f1 (ppm)

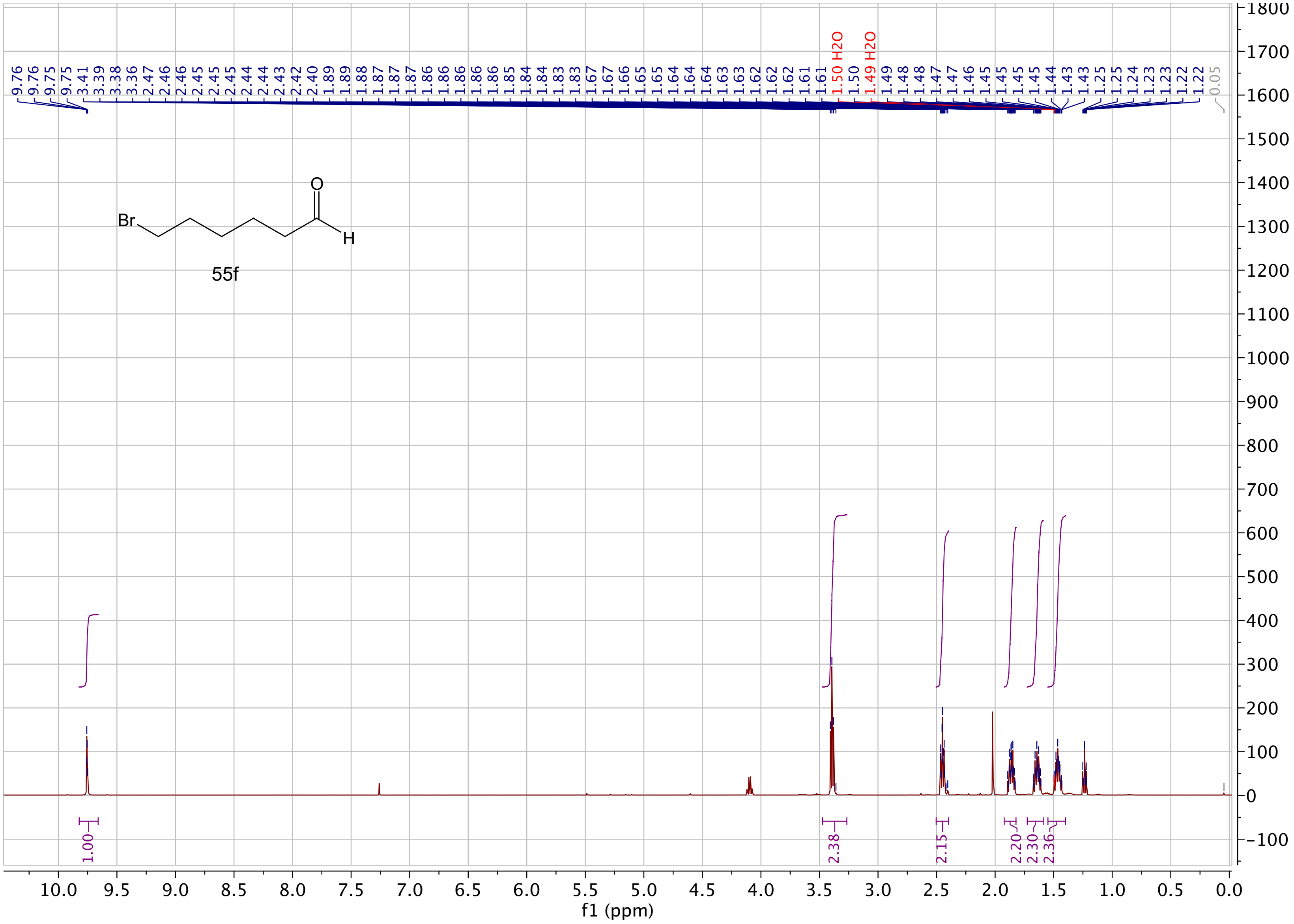

Br
O
H
55f
f1 (ppm)

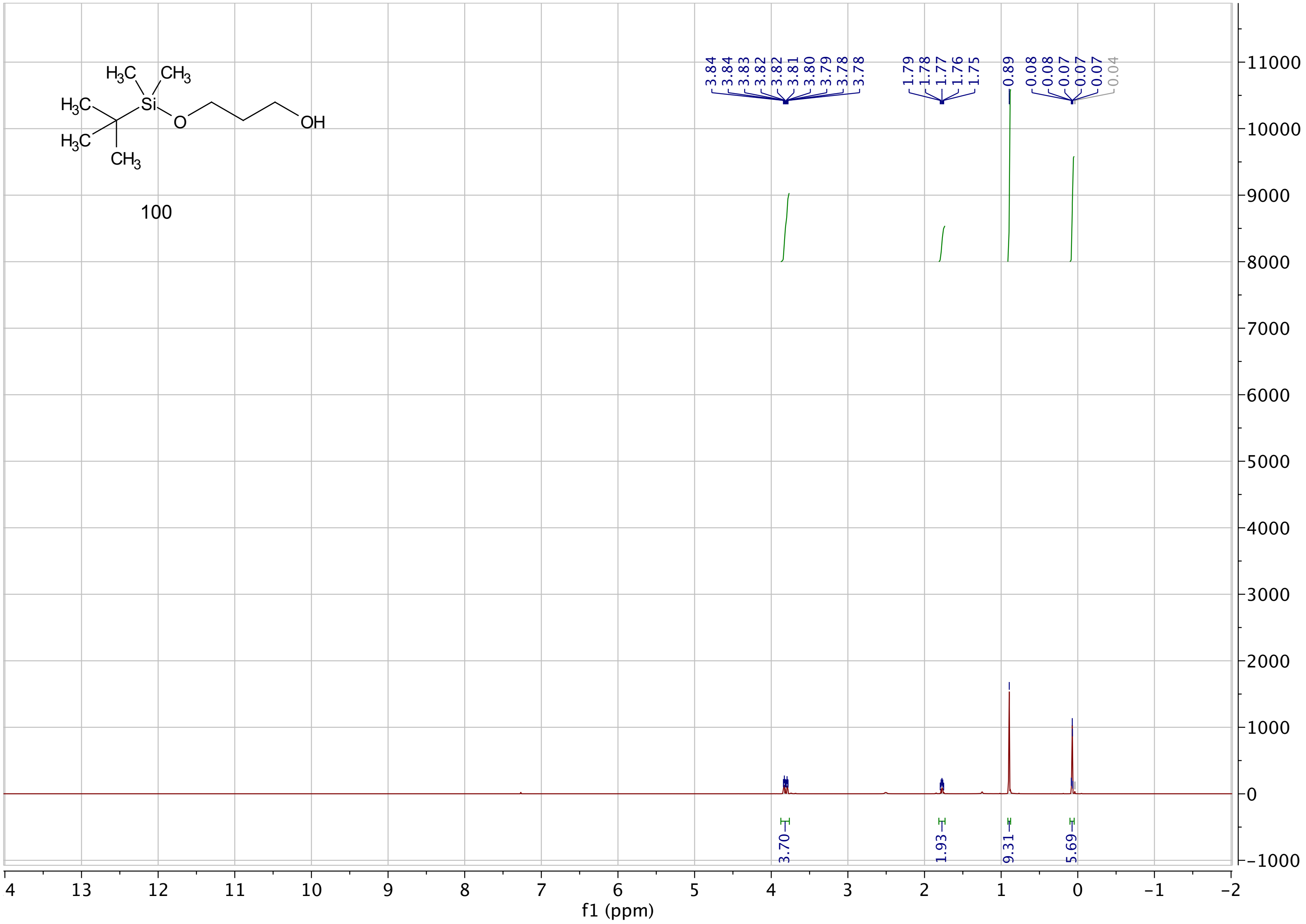

3.84 3.84 3.83 3.82 3.82 3.81 3.80 3.79 3.78 3.78
1.79 1.78 1.77 1.76 1.75
0.89
0.08 0.08 0.07 0.07 0.07 0.04
3.70
1.93
9.31
5.69
f1 (ppm)
100

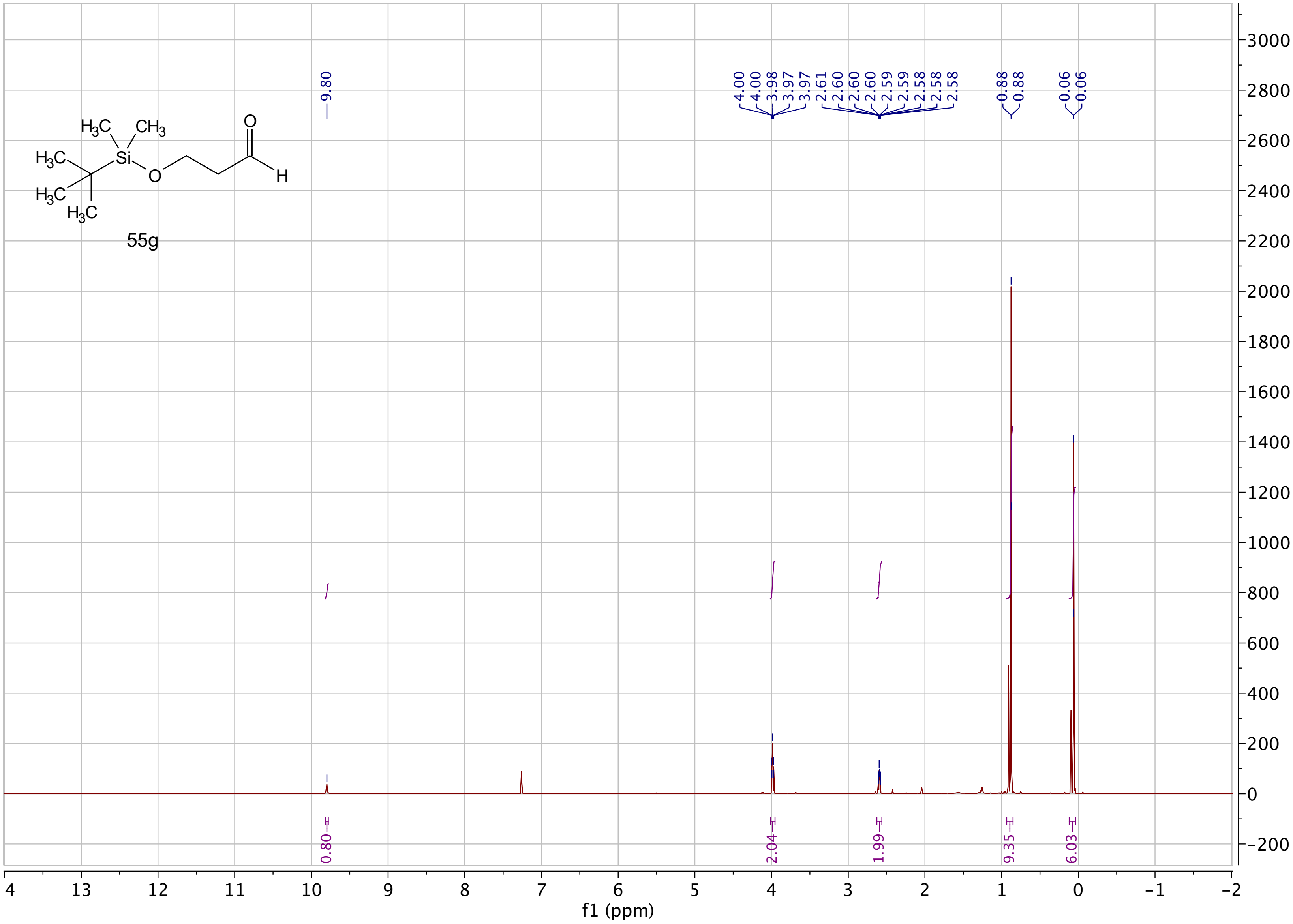

55g
9.80
4.00 4.00 3.98 3.97 3.97
2.61 2.60 2.60 2.60 2.59 2.59 2.58 2.58 2.58
0.88 0.88
0.06 0.06
0.80
2.04
1.99
9.35
6.03
f1 (ppm)

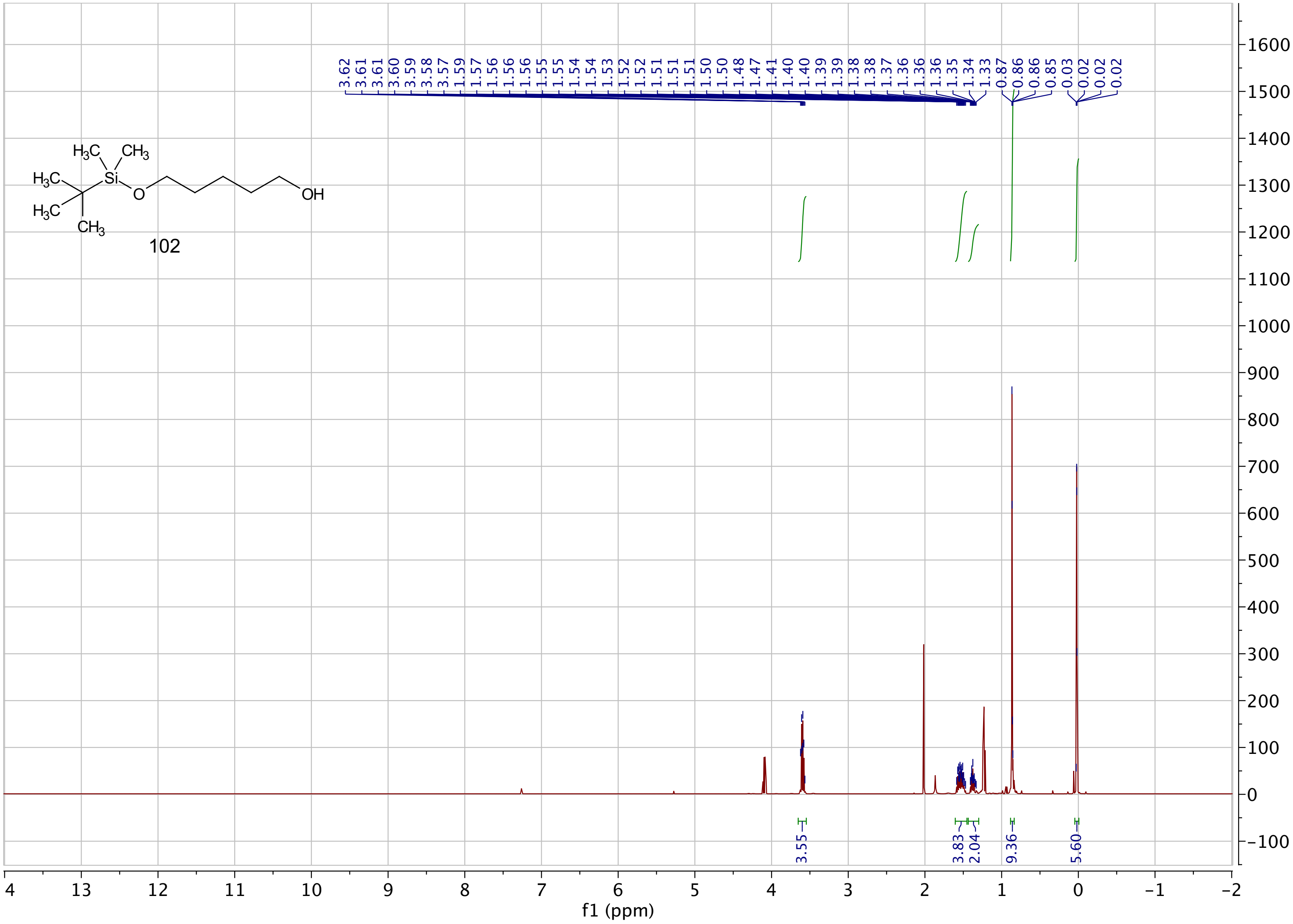
3.62 3.61 3.61 3.60 3.59 3.58 3.57 1.59 1.57 1.56 1.56 1.56 1.55 1.55 1.54 1.54 1.53 1.52 1.52 1.51 1.51 1.51 1.50 1.50 1.48 1.47 1.41 1.40 1.40 1.39 1.39 1.38 1.38 1.37 1.36 1.36 1.35 1.34 1.33 0.87 0.86 0.86 0.85 0.03 0.02 0.02 0.02
H3C CH3 H3C Si O OH H3C CH3
102
3.55
3.83
2.04
9.36
5.60
f1 (ppm)

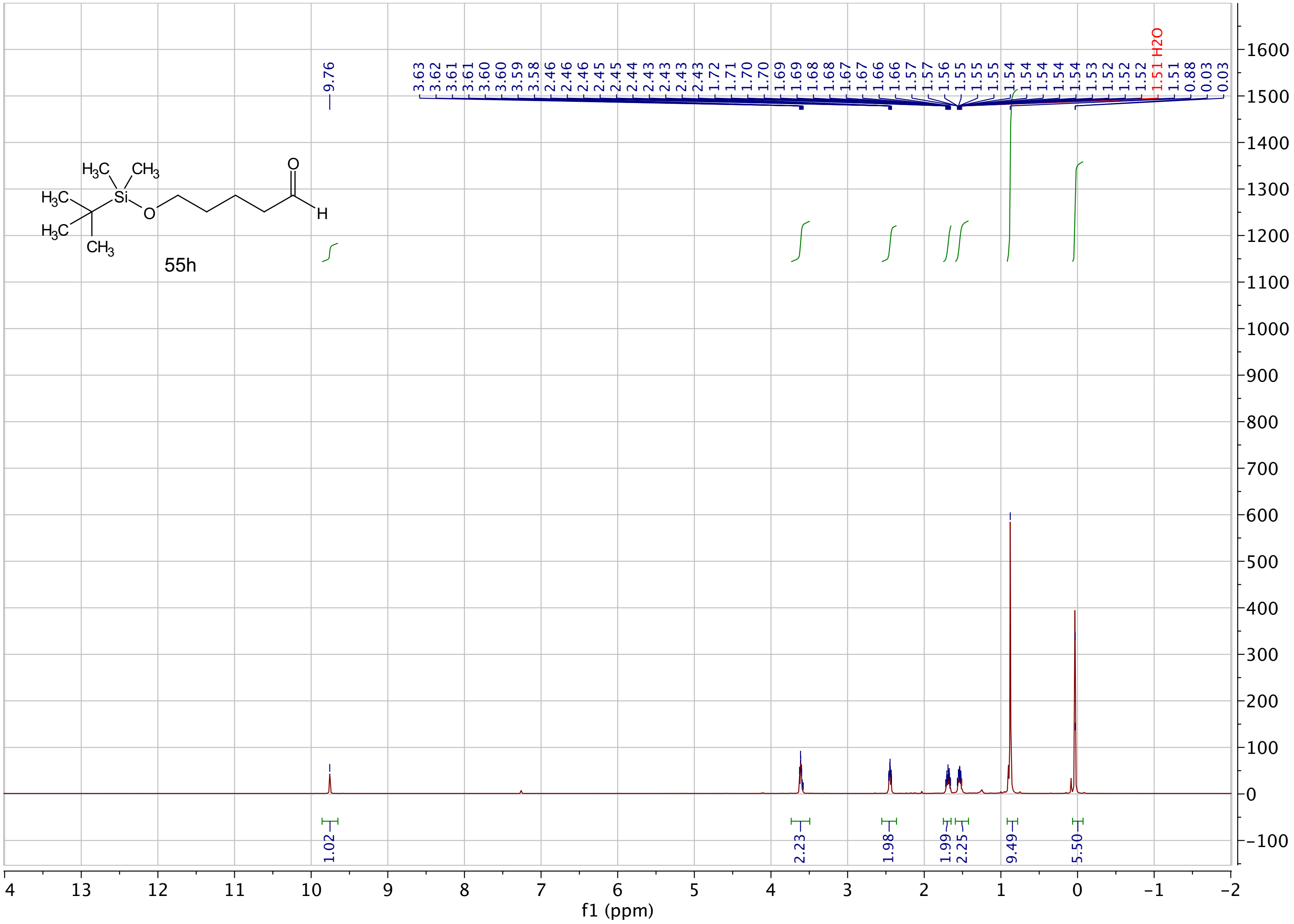

55h
9.76
3.63 3.62 3.61 3.61 3.60 3.60 3.59 3.58 2.46 2.46 2.46 2.45 2.45 2.44 2.43 2.43 2.43 2.43 2.43 1.72 1.71 1.70 1.70 1.69 1.69 1.68 1.68 1.67 1.67 1.66 1.66 1.57 1.57 1.56 1.55 1.55 1.55 1.54 1.54 1.54 1.54 1.54 1.53 1.52 1.52 1.52 1.51 H2O 1.51 0.88 0.03 0.03
1.02 2.23 1.98 1.99 2.25 9.49 5.50
f1 (ppm)

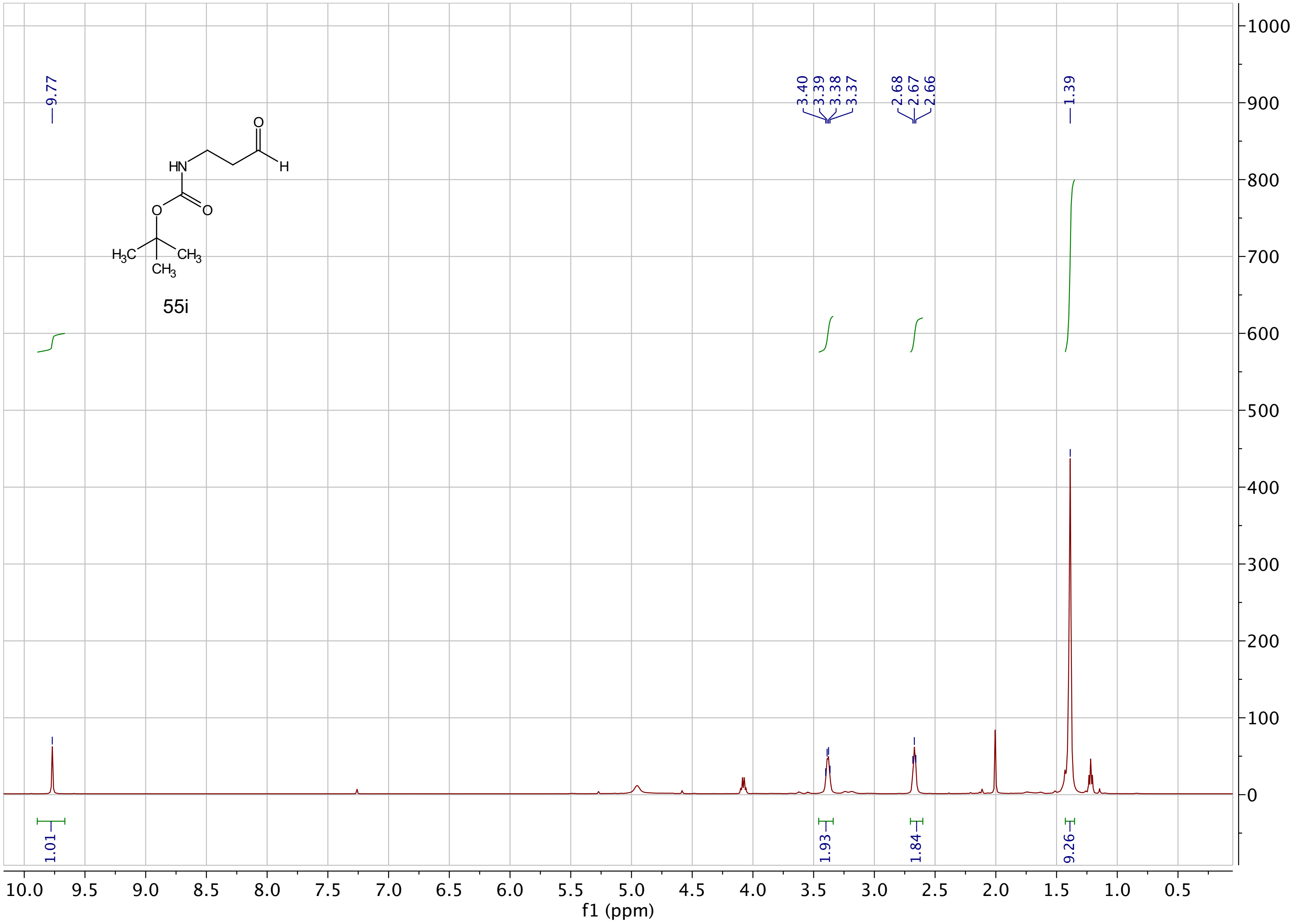

9.77
3.40
3.39
3.38
3.37
2.68
2.67
2.66
1.39
HN
O
H
O
O
H3C
CH3
CH3
55i
1.01
1.93
1.84
9.26
1000
900
800
700
600
500
400
300
200
100
0
10.0 9.5 9.0 8.5 8.0 7.5 7.0 6.5 6.0 5.5 5.0 4.5 4.0 3.5 3.0 2.5 2.0 1.5 1.0 0.5
f1 (ppm)

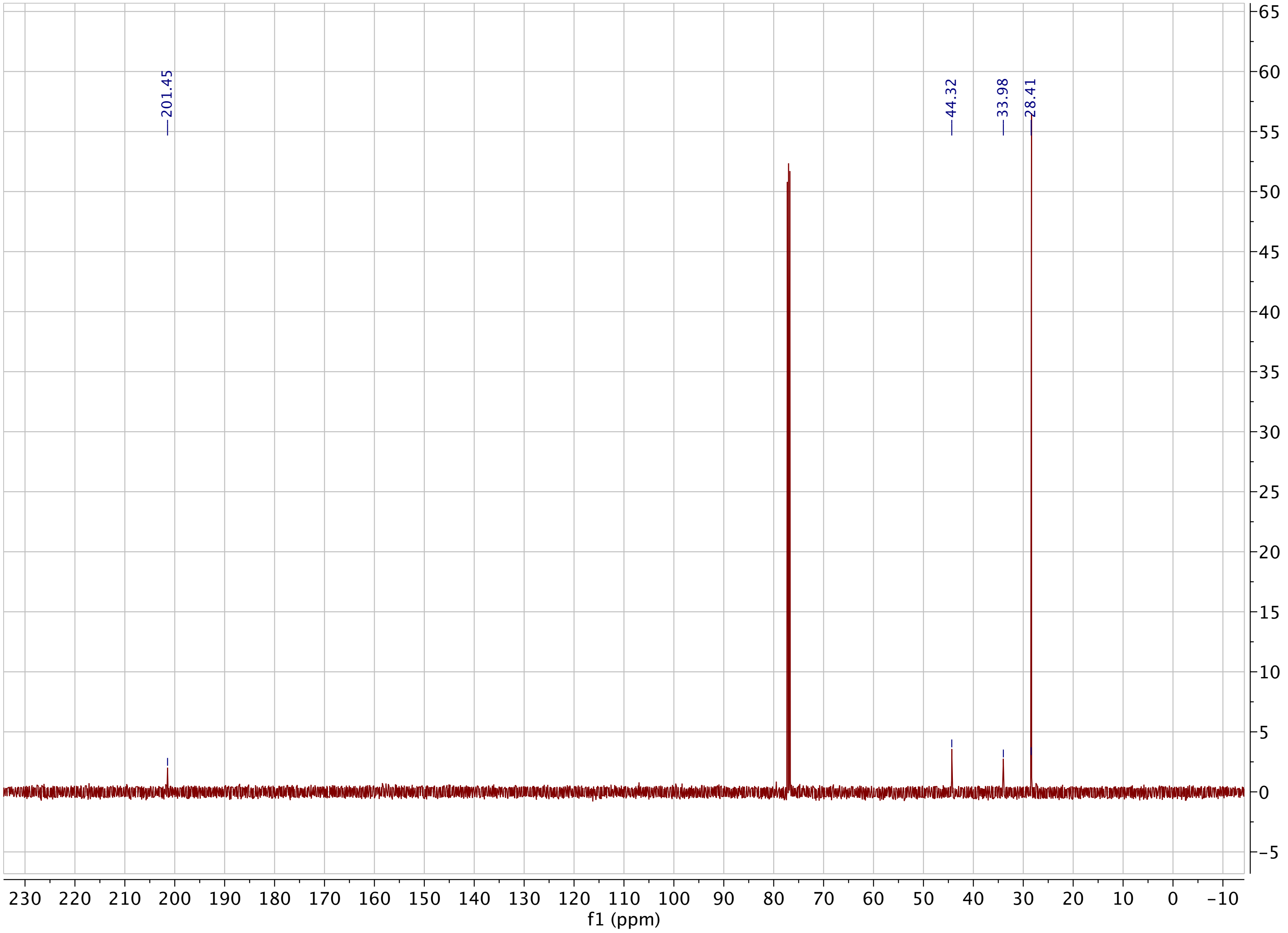
201.45
44.32
33.98
28.41
f1 (ppm)

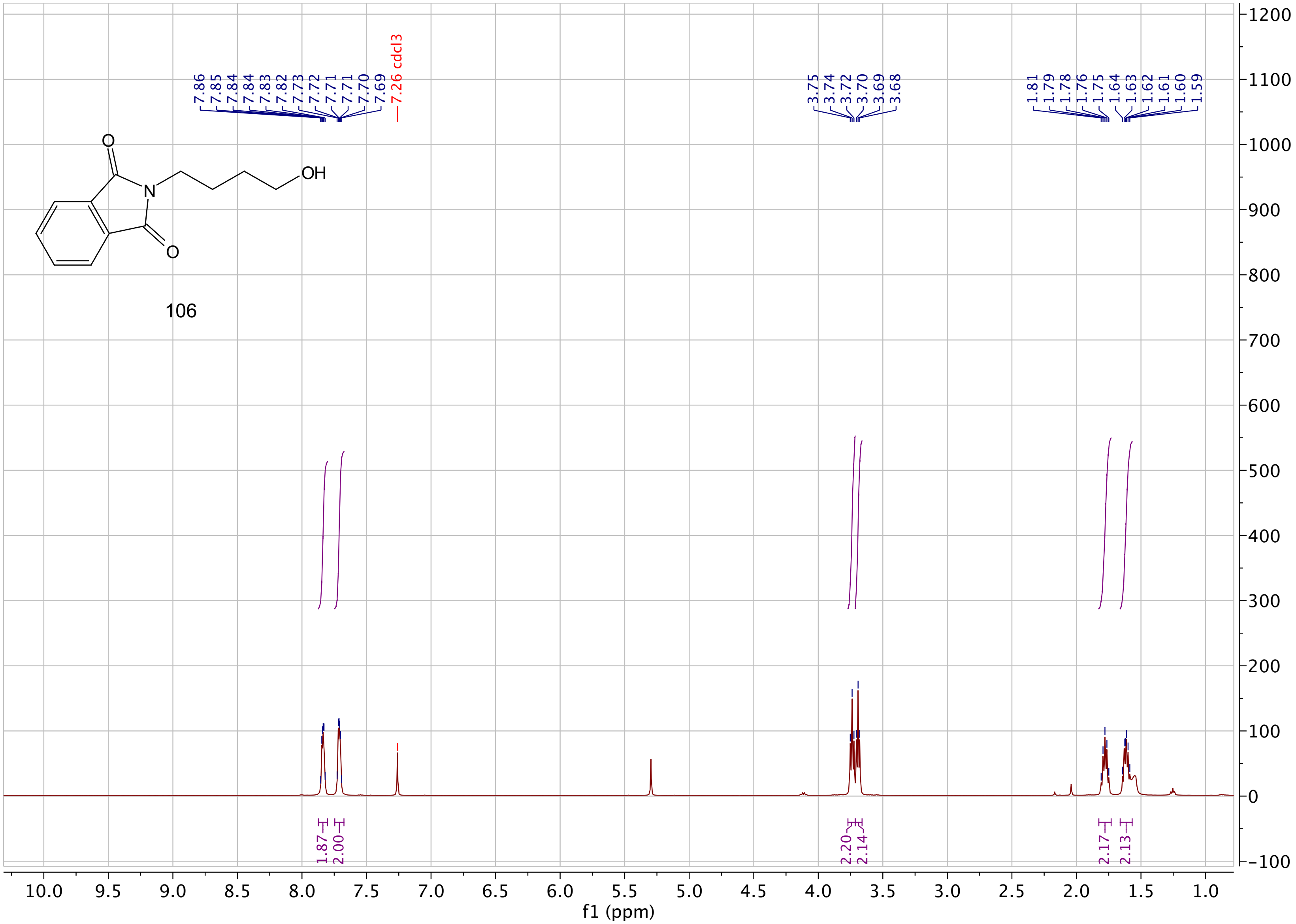

106
OH
N
O
7.86
7.85
7.84
7.84
7.83
7.82
7.73
7.72
7.71
7.71
7.70
7.69
7.26 cdcl3
3.75
3.74
3.72
3.70
3.69
3.68
1.81
1.79
1.78
1.76
1.75
1.64
1.63
1.62
1.61
1.60
1.59
1.87
2.00
2.20
2.14
2.17
2.13
f1 (ppm)

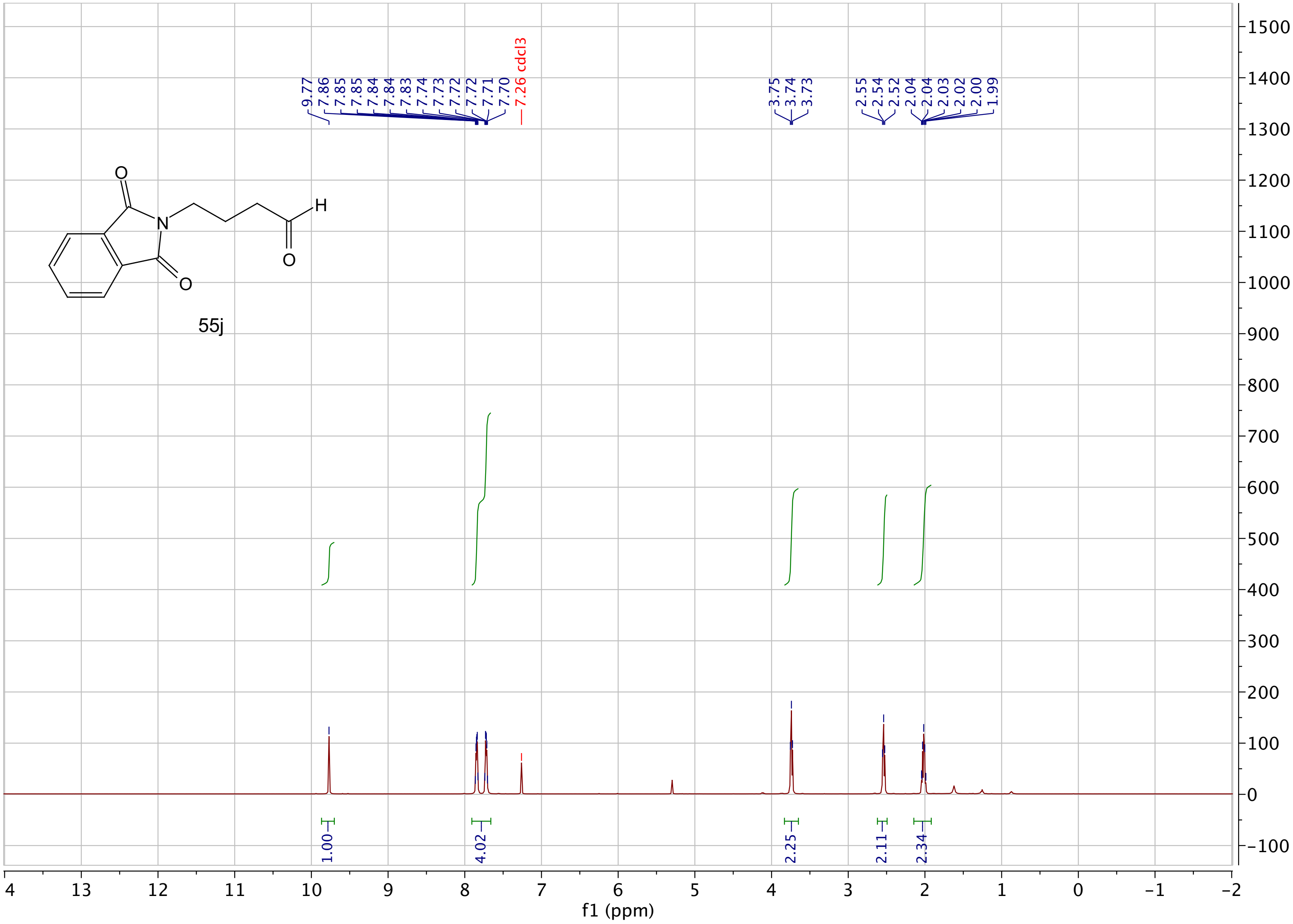
9.77
7.86
7.85
7.85
7.84
7.84
7.83
7.74
7.73
7.72
7.72
7.71
7.70
7.26 cdcl3
3.75
3.74
3.73
2.55
2.54
2.52
2.04
2.04
2.03
2.02
2.00
1.99
O
N
H
O
O
55j
1.00
4.02
2.25
2.11
2.34
f1 (ppm)

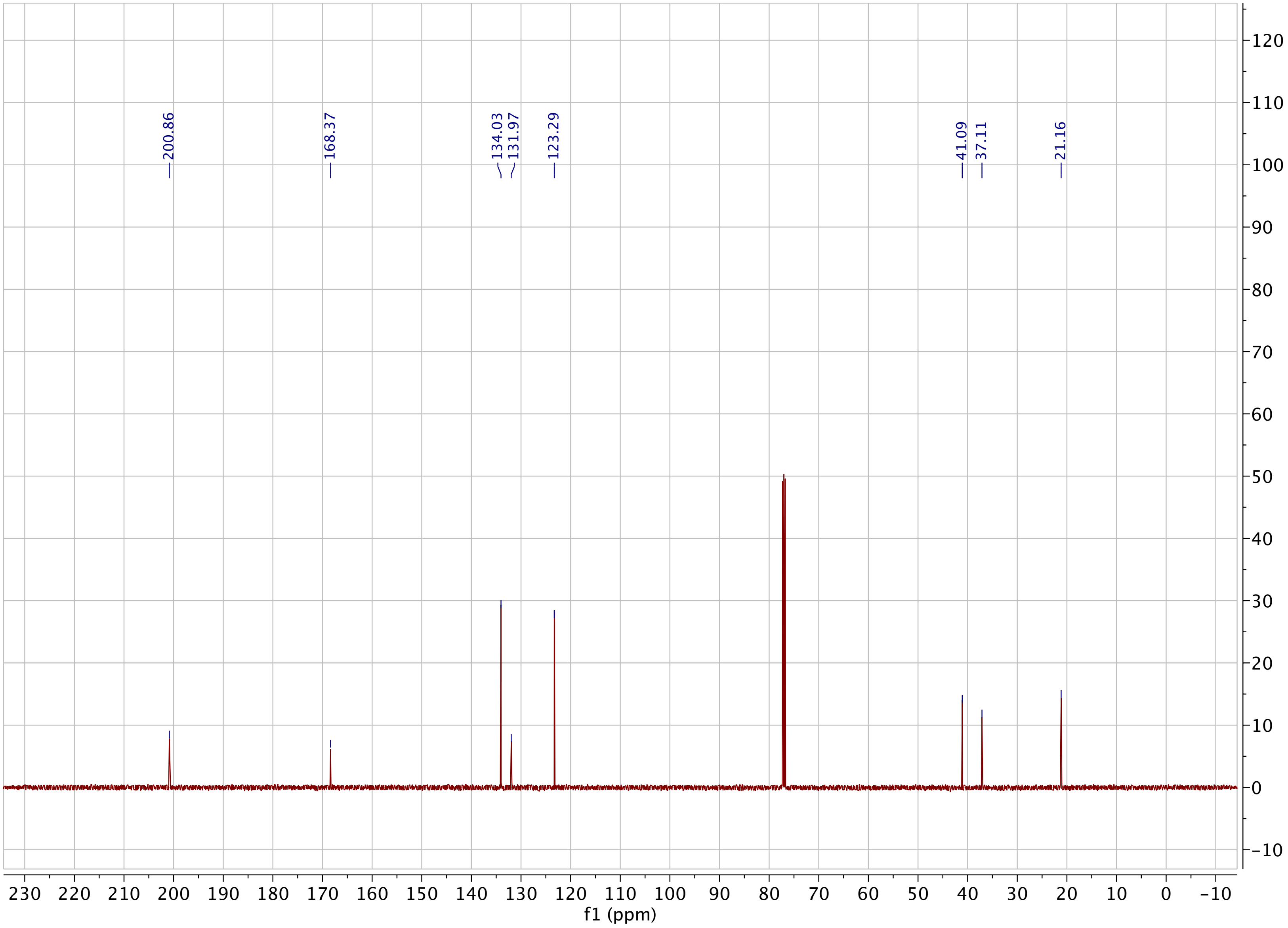
200.86
168.37
134.03
131.97
123.29
41.09
37.11
21.16
f1 (ppm)

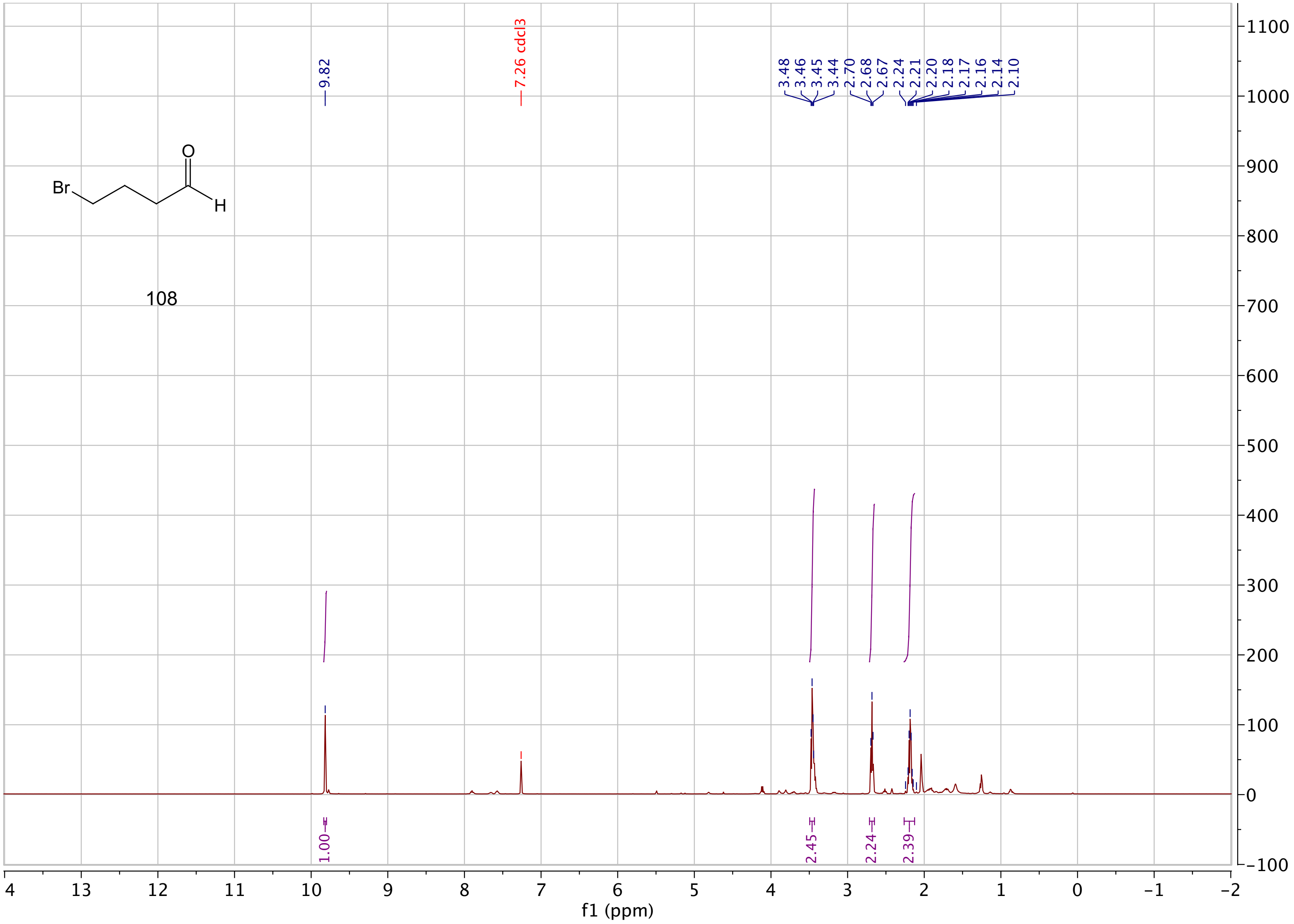

Br
O
H
9.82
7.26 cdcl3
3.48
3.46
3.45
3.44
2.70
2.68
2.67
2.24
2.21
2.20
2.18
2.17
2.16
2.14
2.10
1.00
2.45
2.24
2.39
f1 (ppm)

108

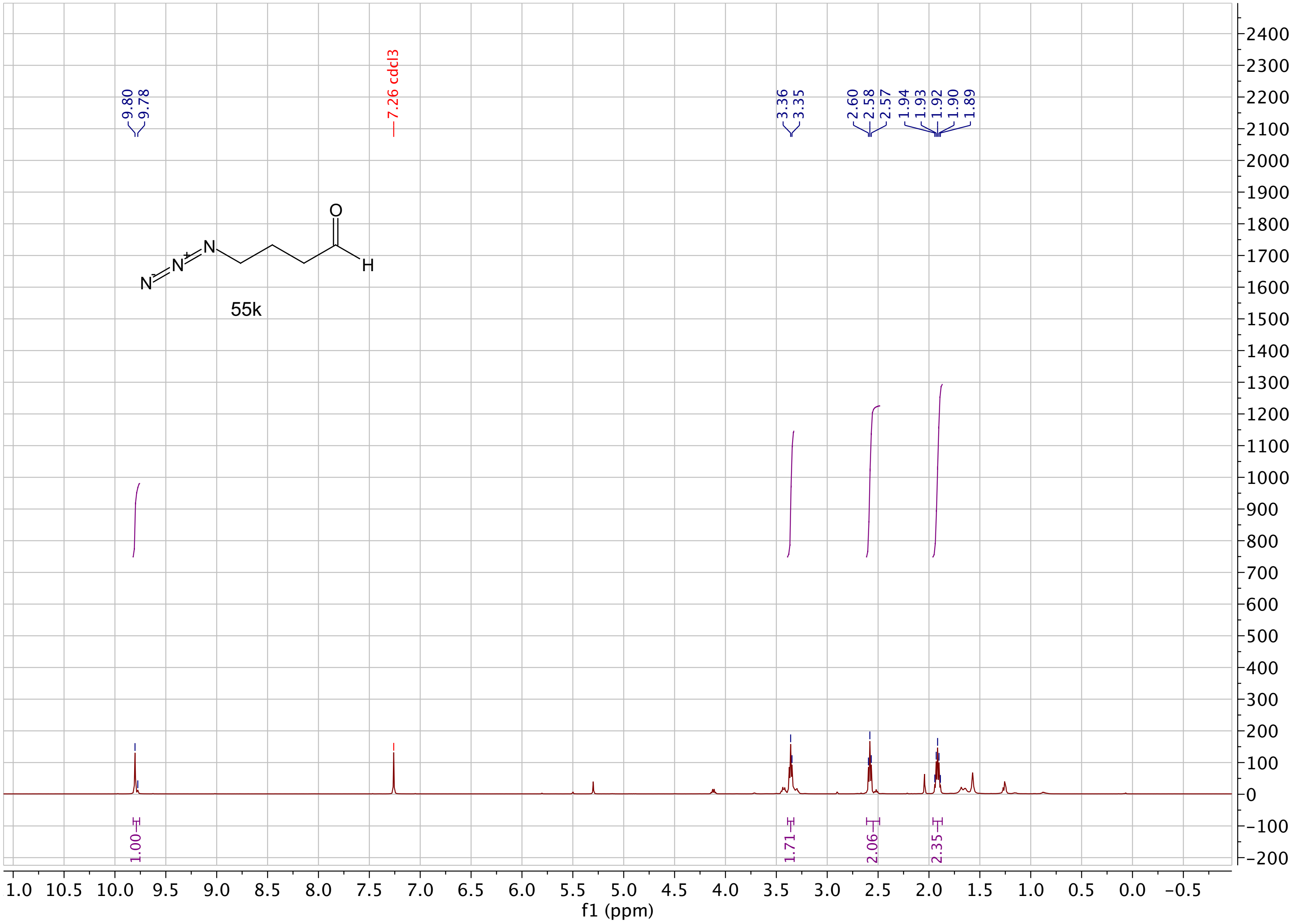
55k
9.80
9.78
7.26 cdcl3
3.36
3.35
2.60
2.58
2.57
1.94
1.93
1.92
1.90
1.89
1.00
1.71
2.06
2.35
f1 (ppm)

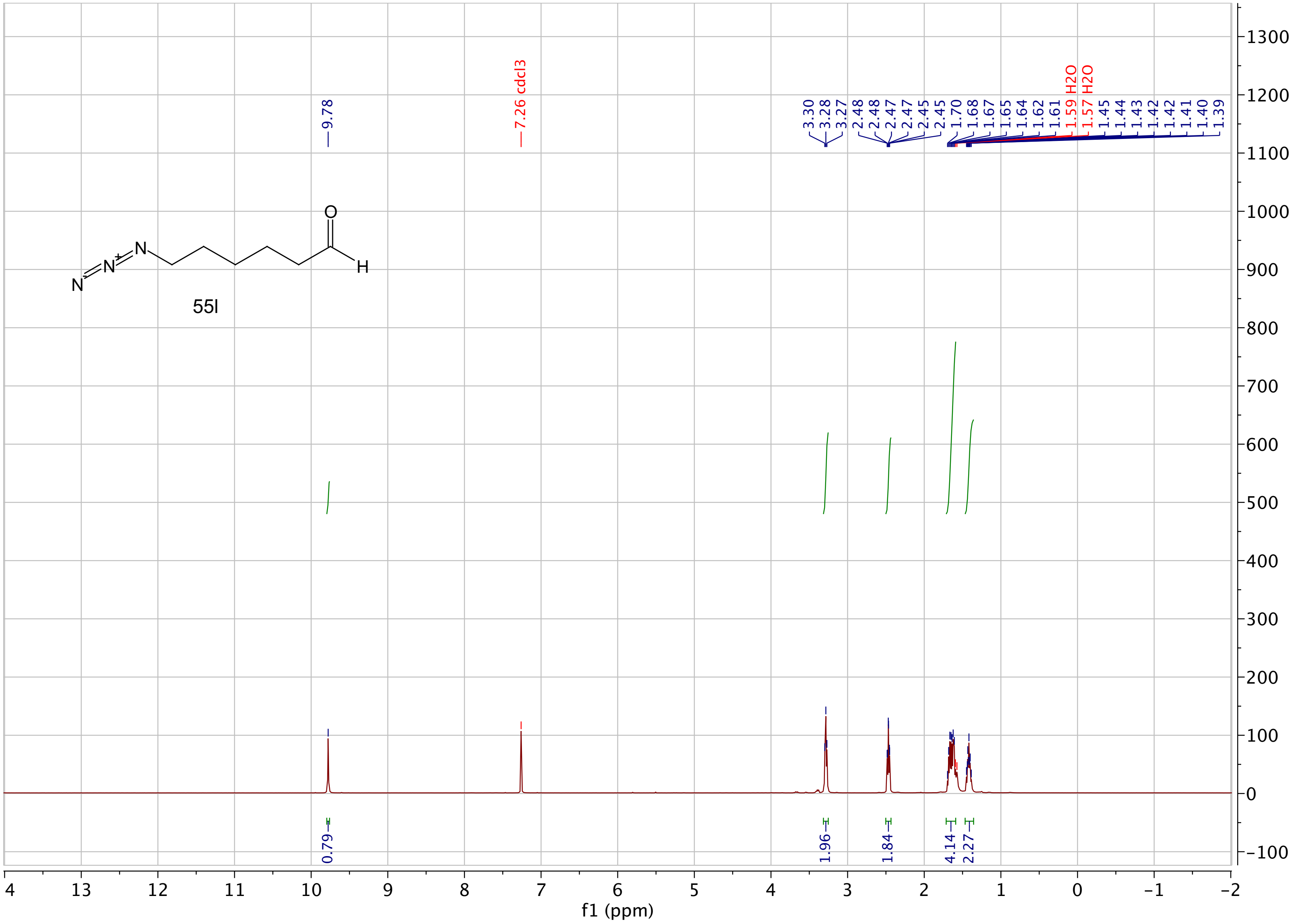

55l
9.78
7.26 cdcl3
3.30
3.28
3.27
2.48
2.48
2.47
2.47
2.45
2.45
1.70
1.68
1.67
1.65
1.64
1.62
1.61
1.59 H2O
1.57 H2O
1.45
1.44
1.43
1.42
1.42
1.41
1.40
1.39
0.79
1.96
1.84
4.14
2.27
f1 (ppm)

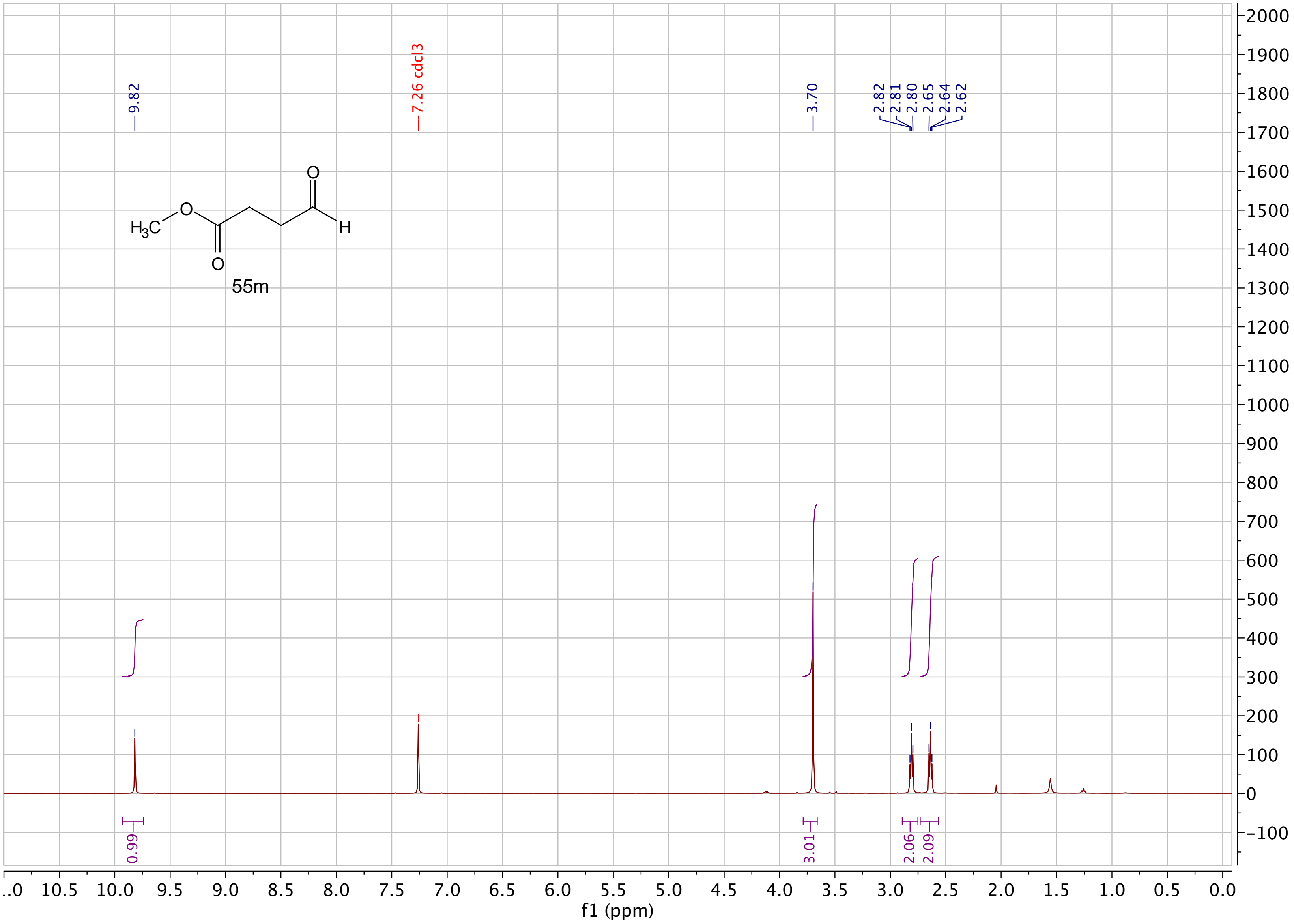

9.82
7.26 cdcl3
3.70
2.82
2.81
2.80
2.65
2.64
2.62
H_3C
O
O
O
H
55m
0.99
3.01
2.06
2.09
f1 (ppm)

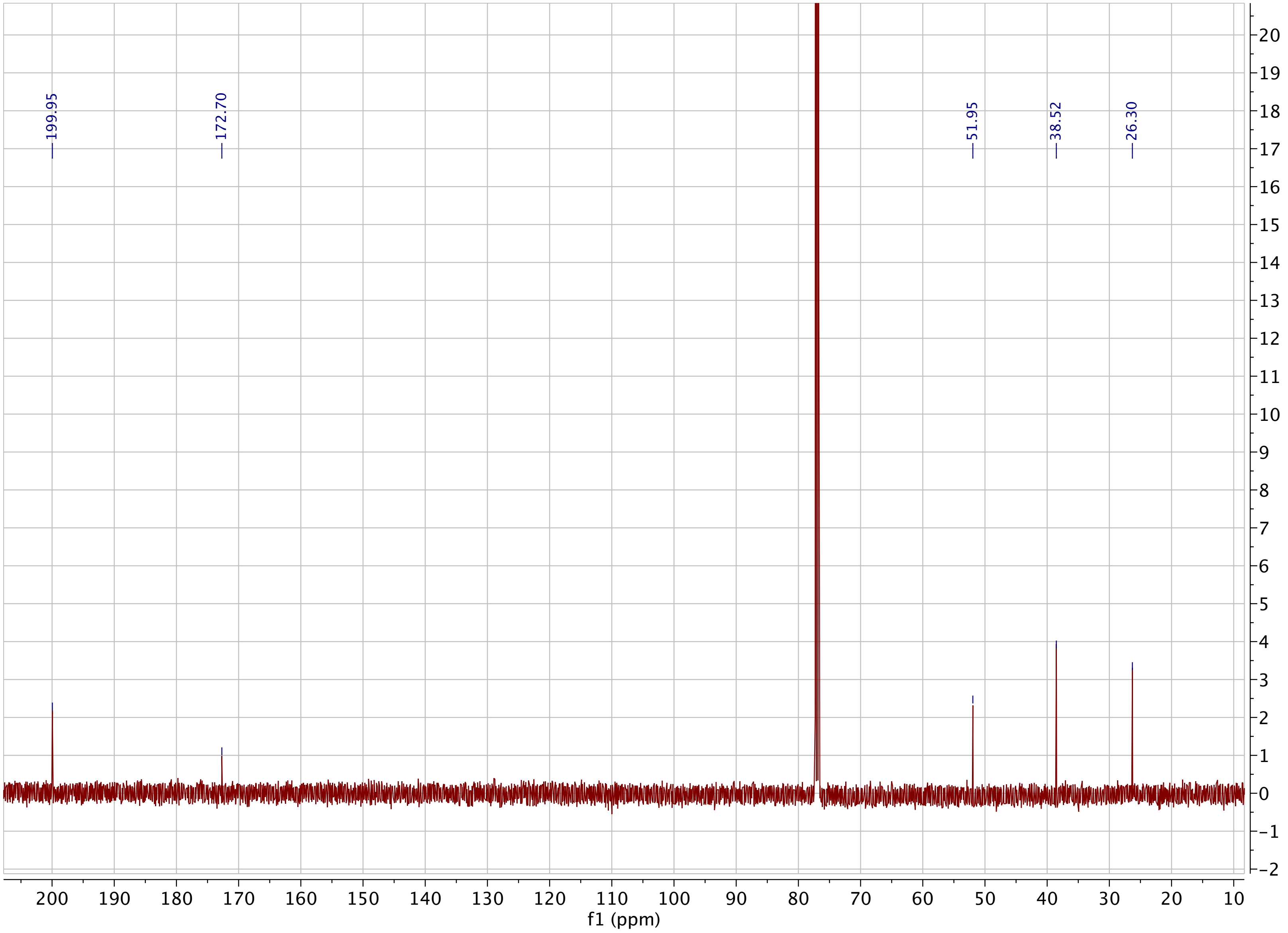

199.95
172.70
51.95
38.52
26.30
f1 (ppm)

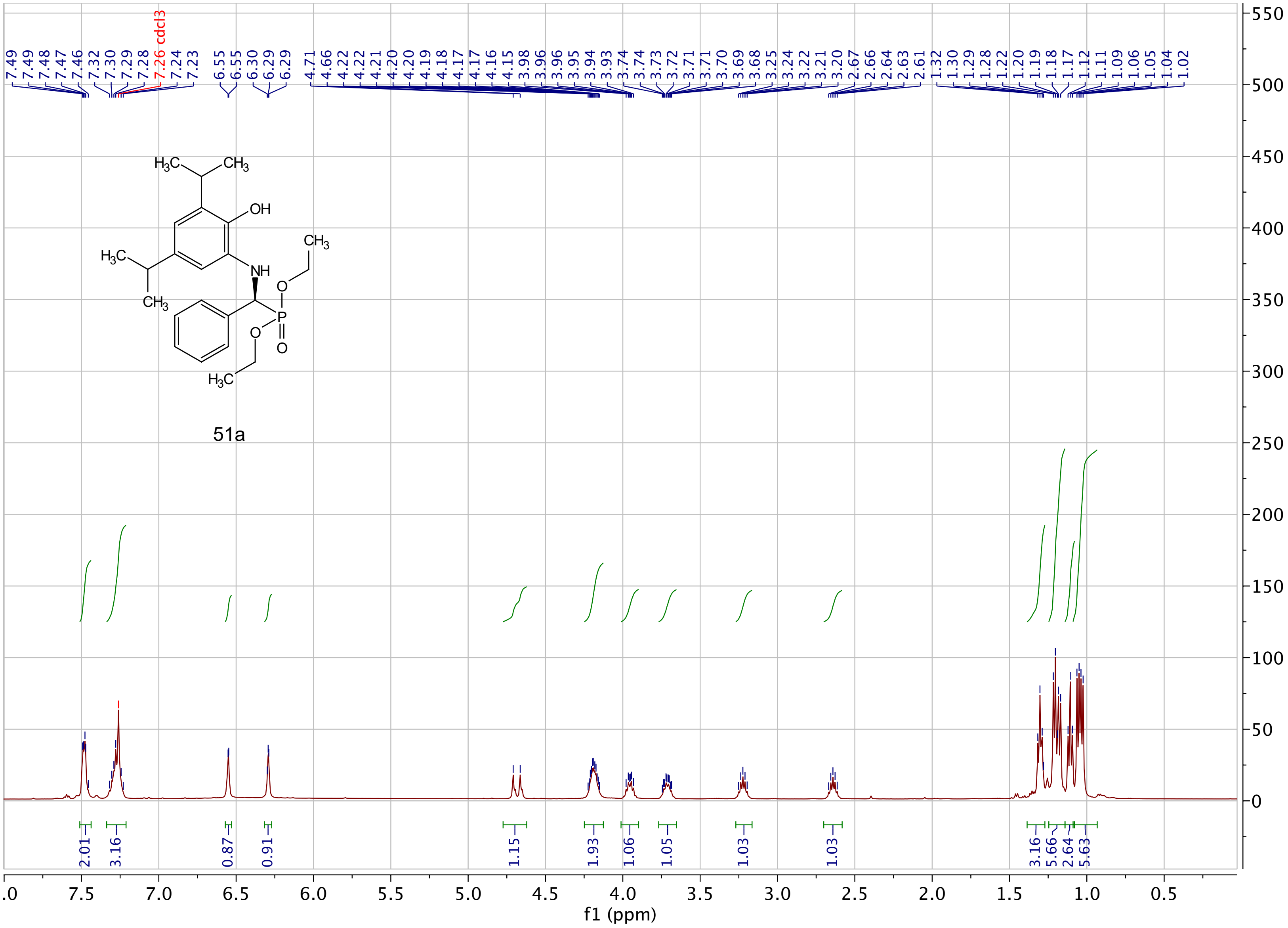

51a

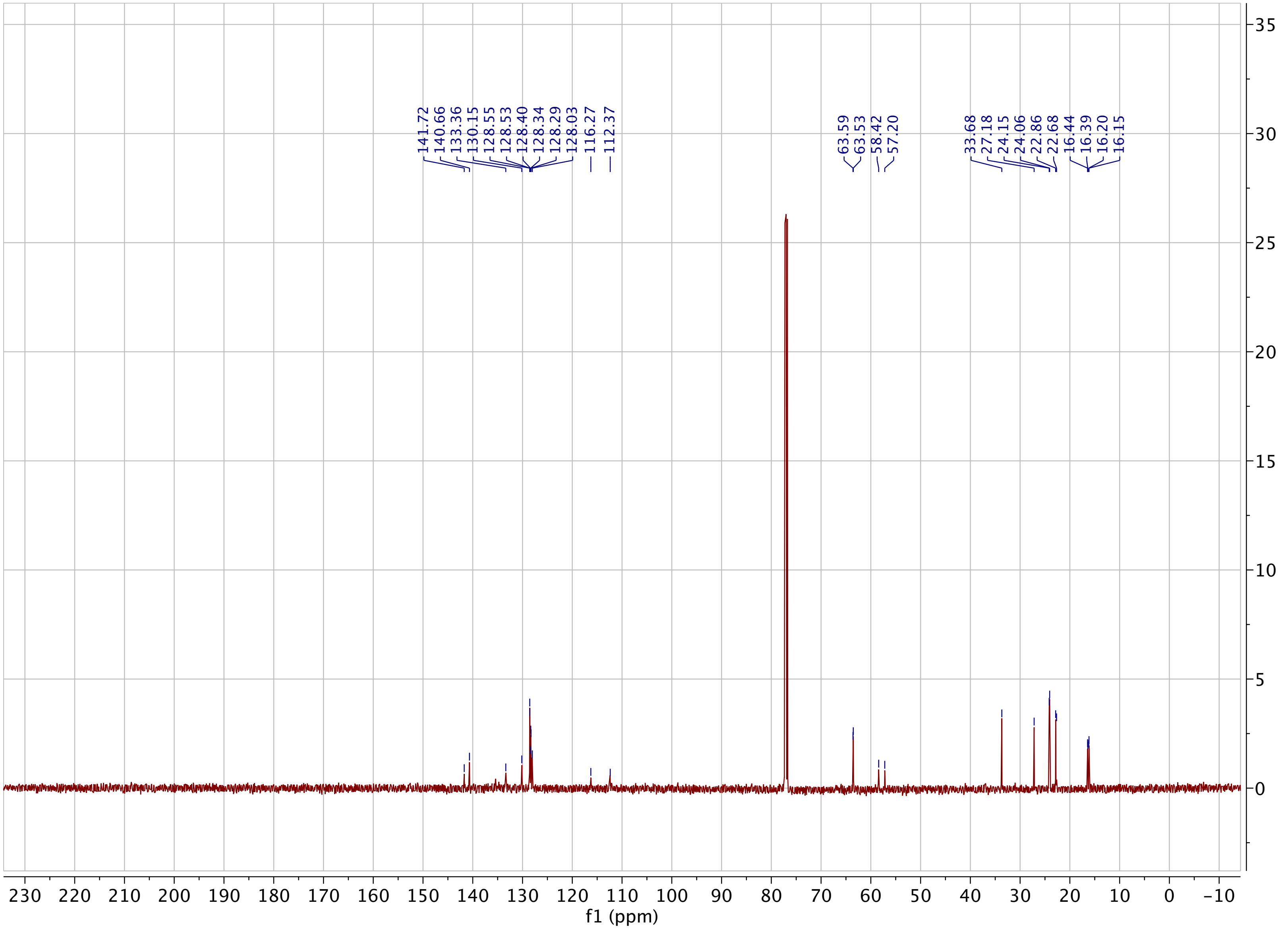
141.72
140.66
133.36
130.15
128.55
128.53
128.40
128.34
128.29
128.03
116.27
112.37
63.59
63.53
58.42
57.20
33.68
27.18
24.15
24.06
22.86
22.68
16.44
16.39
16.20
16.15
f1 (ppm)

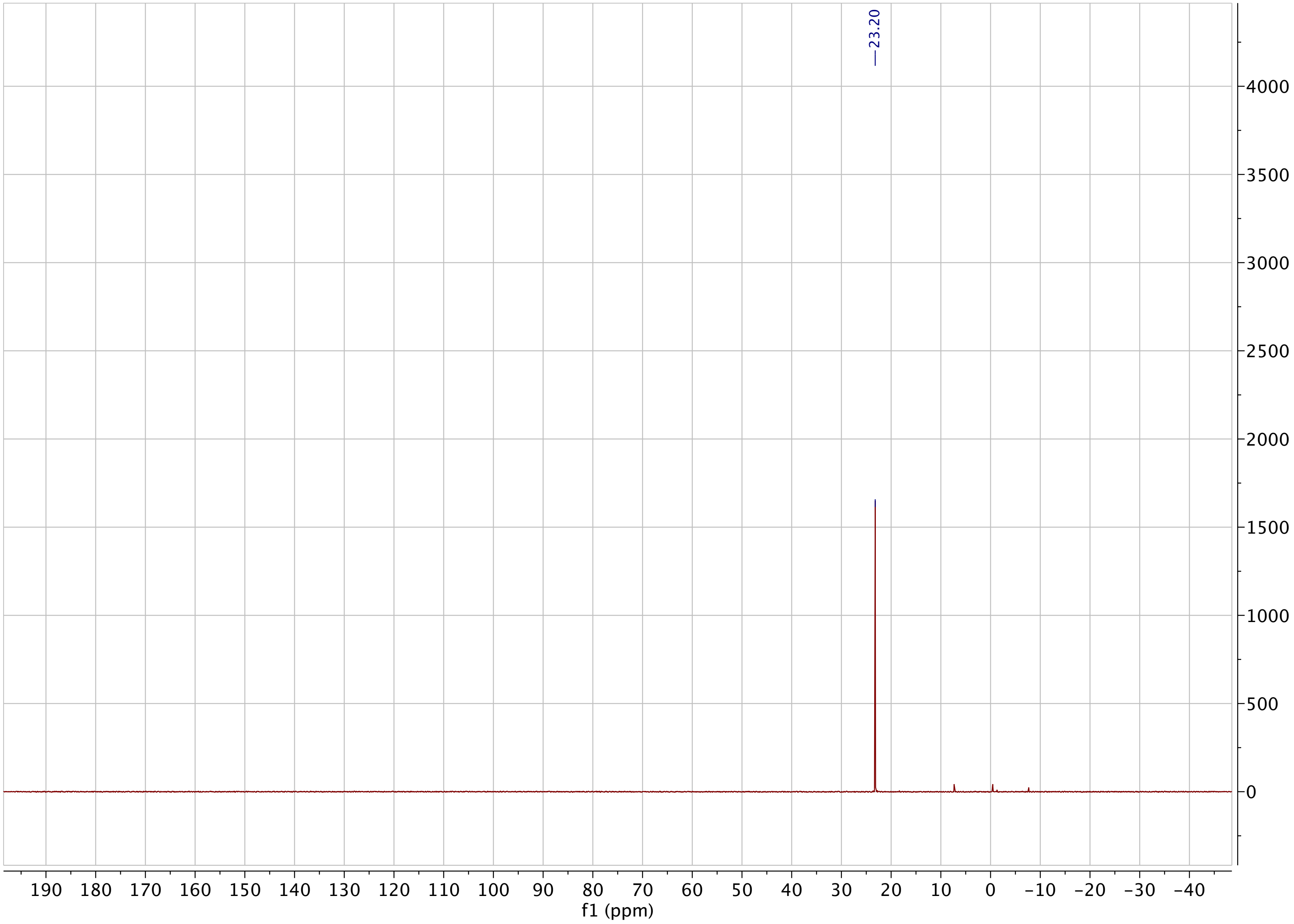
23.20
4000
3500
3000
2500
2000
1500
1000
500
0
190 180 170 160 150 140 130 120 110 100 90 80 70 60 50 40 30 20 10 0 −10 −20 −30 −40
f1 (ppm)

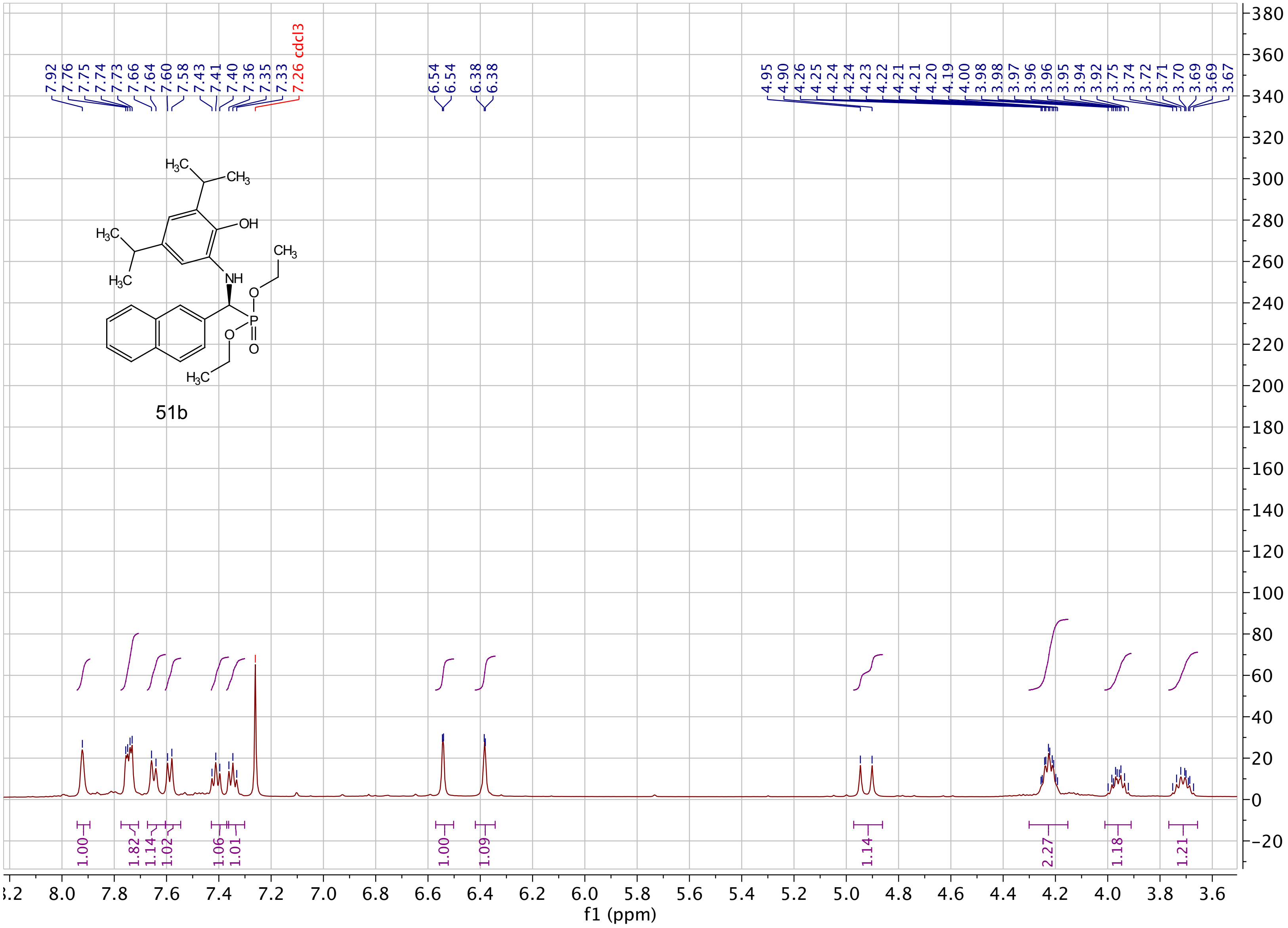
7.92
7.76
7.75
7.74
7.73
7.66
7.64
7.60
7.58
7.43
7.41
7.40
7.36
7.35
7.33
7.26 cdcl3
6.54
6.54
6.38
6.38
4.95
4.90
4.26
4.25
4.24
4.24
4.23
4.22
4.21
4.21
4.20
4.19
4.00
3.98
3.98
3.97
3.96
3.96
3.95
3.94
3.92
3.75
3.74
3.72
3.71
3.70
3.69
3.69
3.67
H3C
CH3
OH
H3C
CH3
H3C
NH
O
P
O
O
H3C
51b
1.00
1.82
1.14
1.02
1.06
1.01
1.00
1.09
1.14
2.27
1.18
1.21
f1 (ppm)

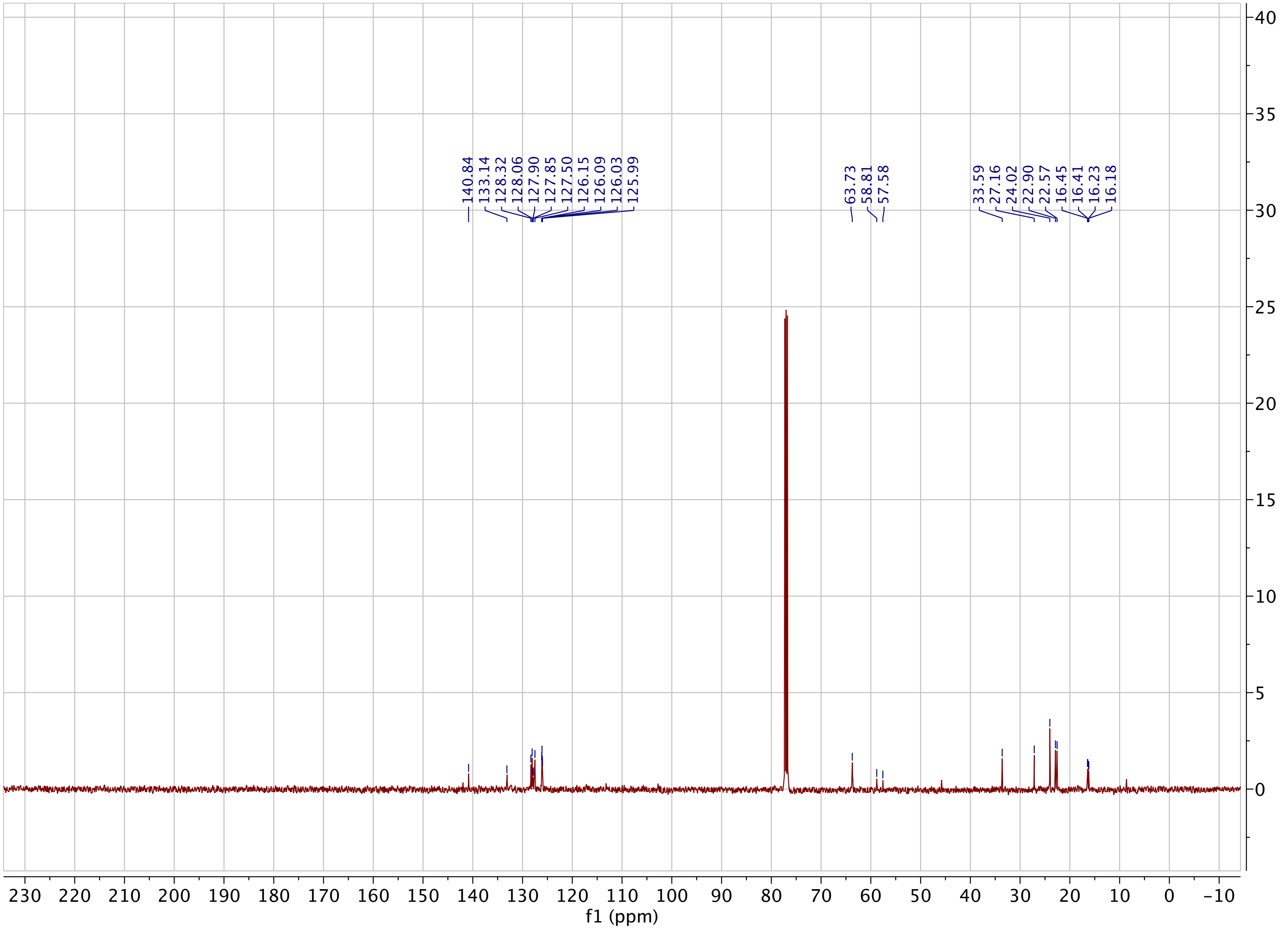

140.84
133.14
128.32
128.06
127.90
127.85
127.50
126.15
126.09
126.03
125.99
63.73
58.81
57.58
33.59
27.16
24.02
22.90
22.57
16.45
16.41
16.23
16.18
f1 (ppm)

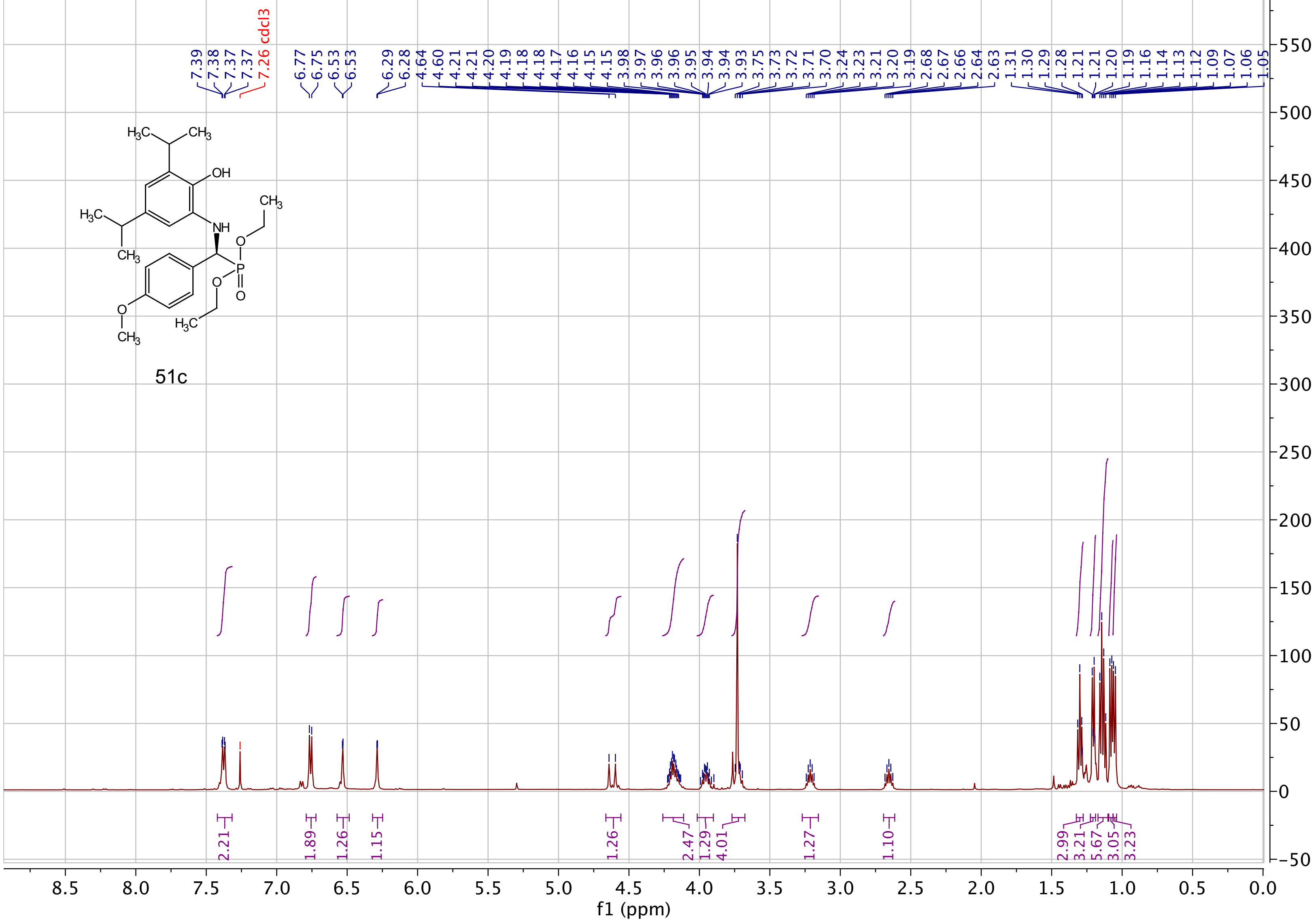
7.39 7.38 7.37 7.37 7.26 cdcl3 6.77 6.75 6.53 6.53 6.29 6.28 4.64 4.60 4.21 4.21 4.20 4.19 4.18 4.18 4.17 4.16 4.15 4.15 3.98 3.97 3.96 3.96 3.95 3.94 3.94 3.93 3.75 3.73 3.72 3.71 3.70 3.24 3.23 3.21 3.20 3.19 2.68 2.67 2.66 2.64 2.63 1.31 1.30 1.29 1.28 1.21 1.21 1.20 1.19 1.16 1.14 1.13 1.12 1.09 1.07 1.06 1.05
51c
2.21 1.89 1.26 1.15 1.26 2.47 1.29 4.01 1.27 1.10 2.99 3.21 5.67 3.05 3.23
f1 (ppm)

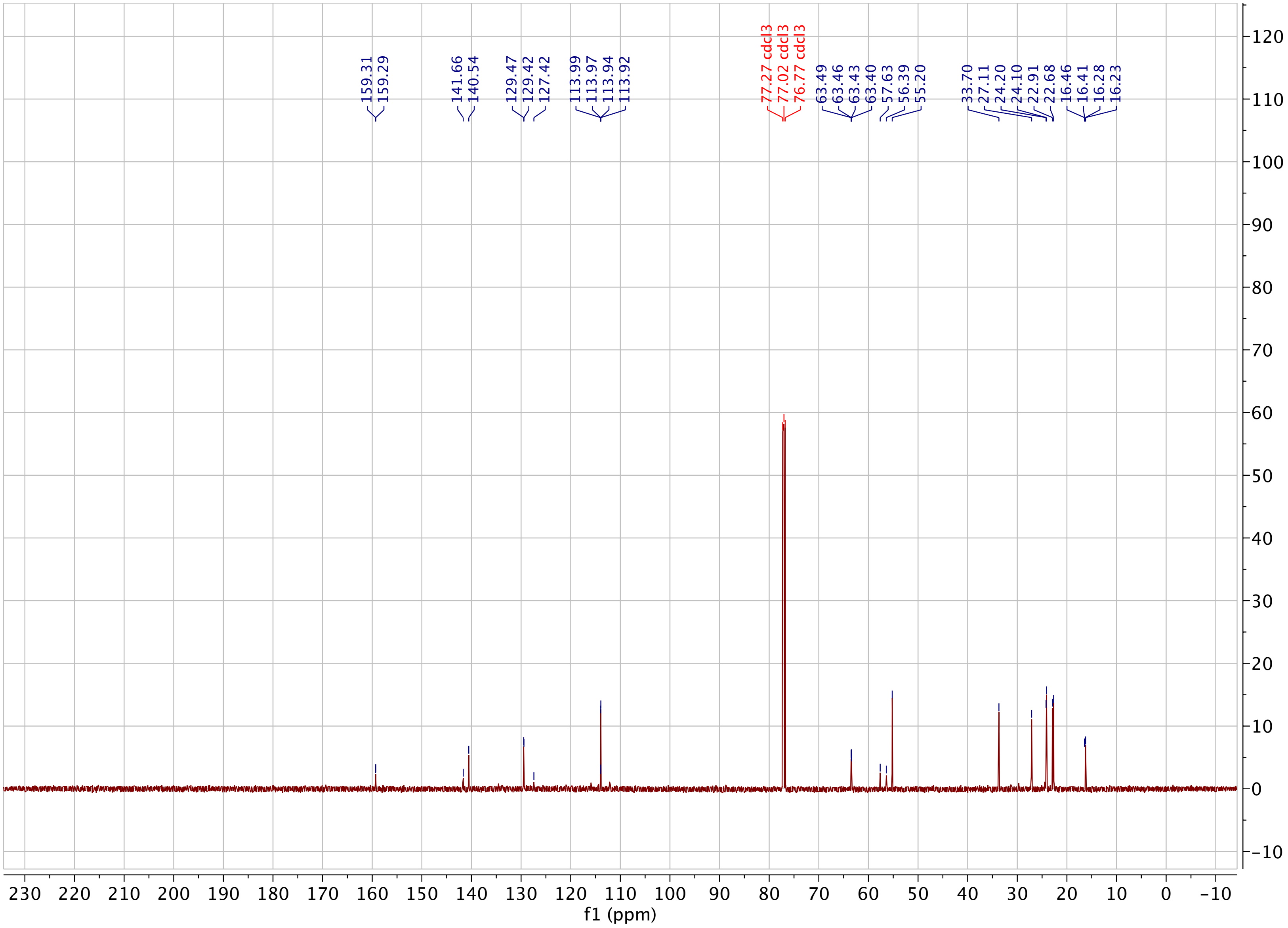
159.31
159.29
141.66
140.54
129.47
129.42
127.42
113.99
113.97
113.94
113.92
77.27 cdcl3
77.02 cdcl3
76.77 cdcl3
63.49
63.46
63.43
63.40
57.63
56.39
55.20
33.70
27.11
24.20
24.10
22.91
22.68
16.46
16.41
16.28
16.23
f1 (ppm)

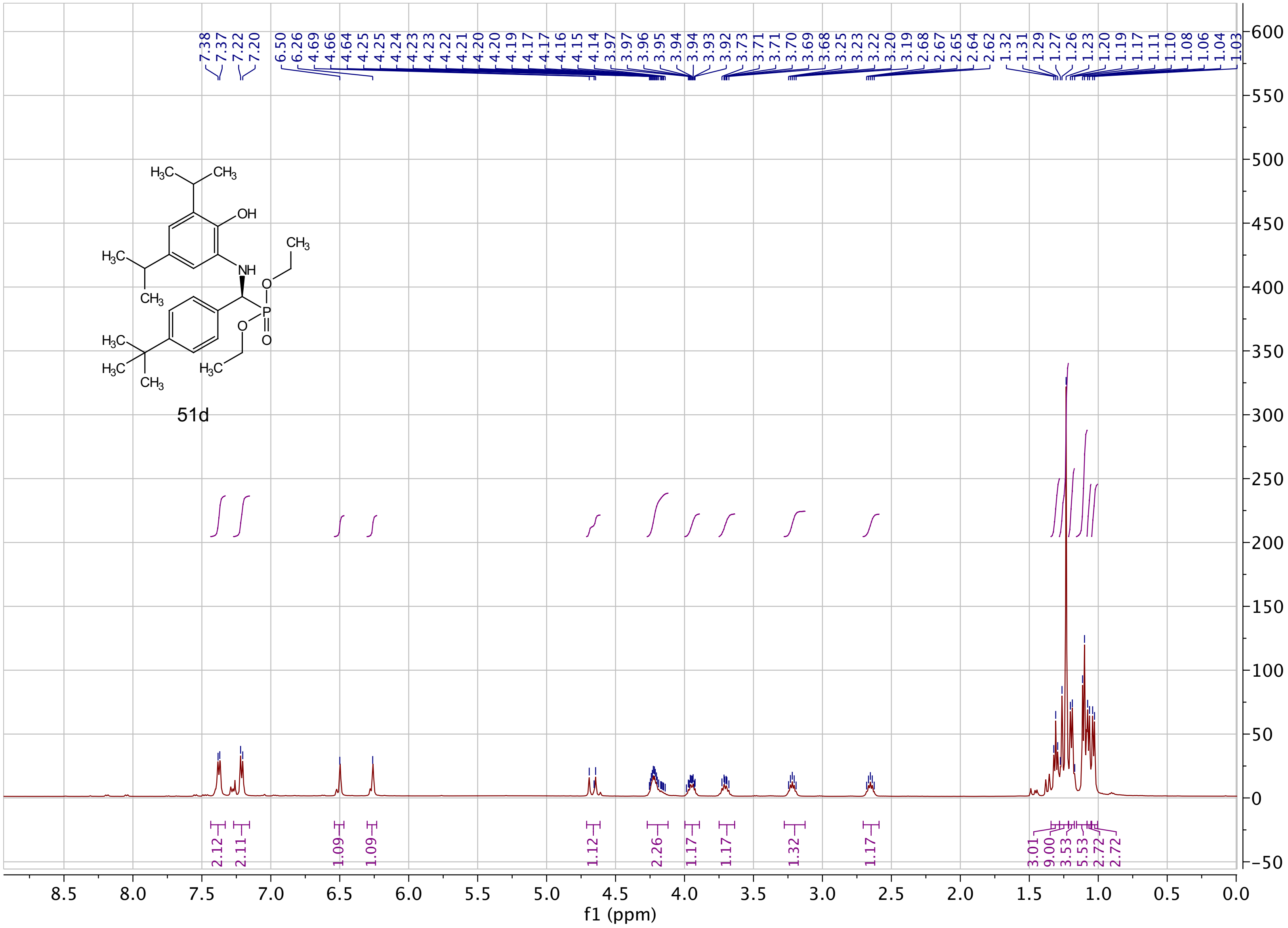
7.38
7.37
7.22
7.20
6.50
6.26
4.69
4.66
4.64
4.25
4.25
4.24
4.23
4.23
4.22
4.21
4.20
4.20
4.19
4.17
4.16
4.15
4.14
3.97
3.97
3.96
3.95
3.94
3.94
3.93
3.92
3.73
3.71
3.71
3.70
3.69
3.68
3.25
3.23
3.22
3.20
3.19
2.68
2.67
2.65
2.64
2.62
1.32
1.31
1.29
1.27
1.26
1.23
1.20
1.19
1.17
1.11
1.10
1.08
1.06
1.04
1.03
H3C
CH3
OH
CH3
H3C
NH
CH3
O
P
O
O
H3C
H3C
H3C
CH3
51d
2.12
2.11
1.09
1.09
1.12
2.26
1.17
1.17
1.32
1.17
3.01
9.00
3.53
5.53
2.72
2.72
f1 (ppm)

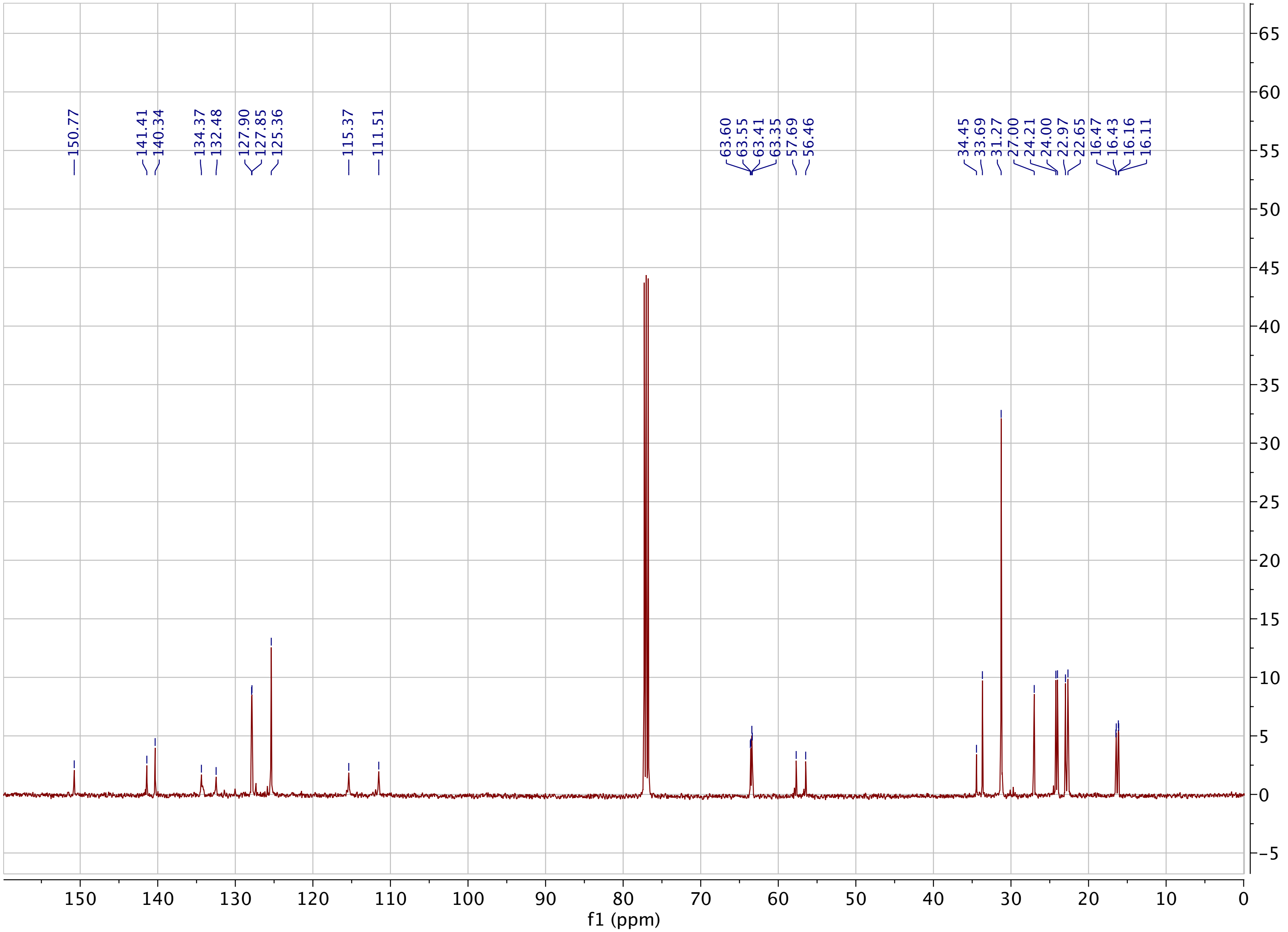
150.77
141.41
140.34
134.37
132.48
127.90
127.85
125.36
115.37
111.51
63.60
63.55
63.41
63.35
57.69
56.46
34.45
33.69
31.27
27.00
24.21
24.00
22.97
22.65
16.47
16.43
16.16
16.11
f1 (ppm)

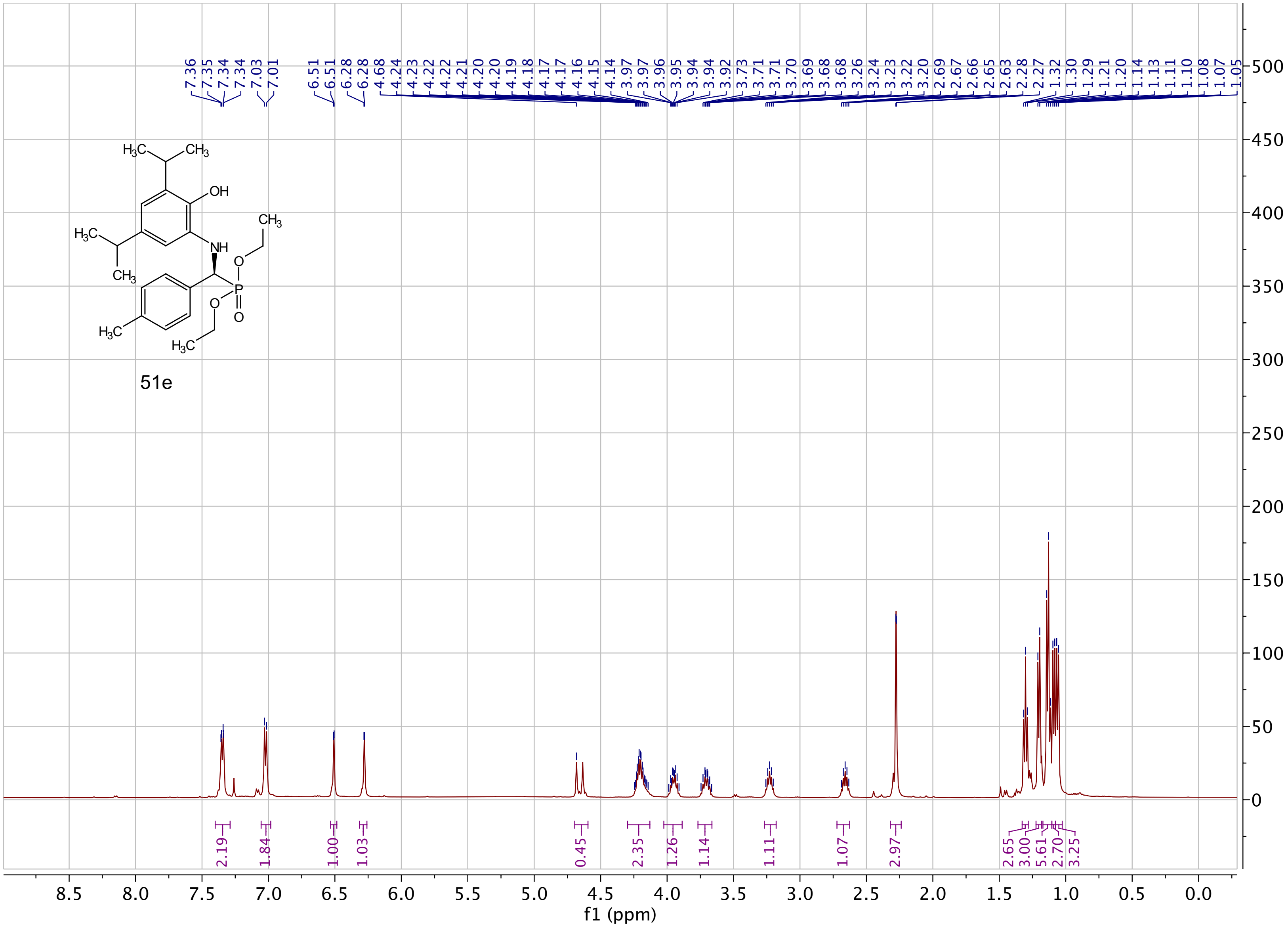

51e

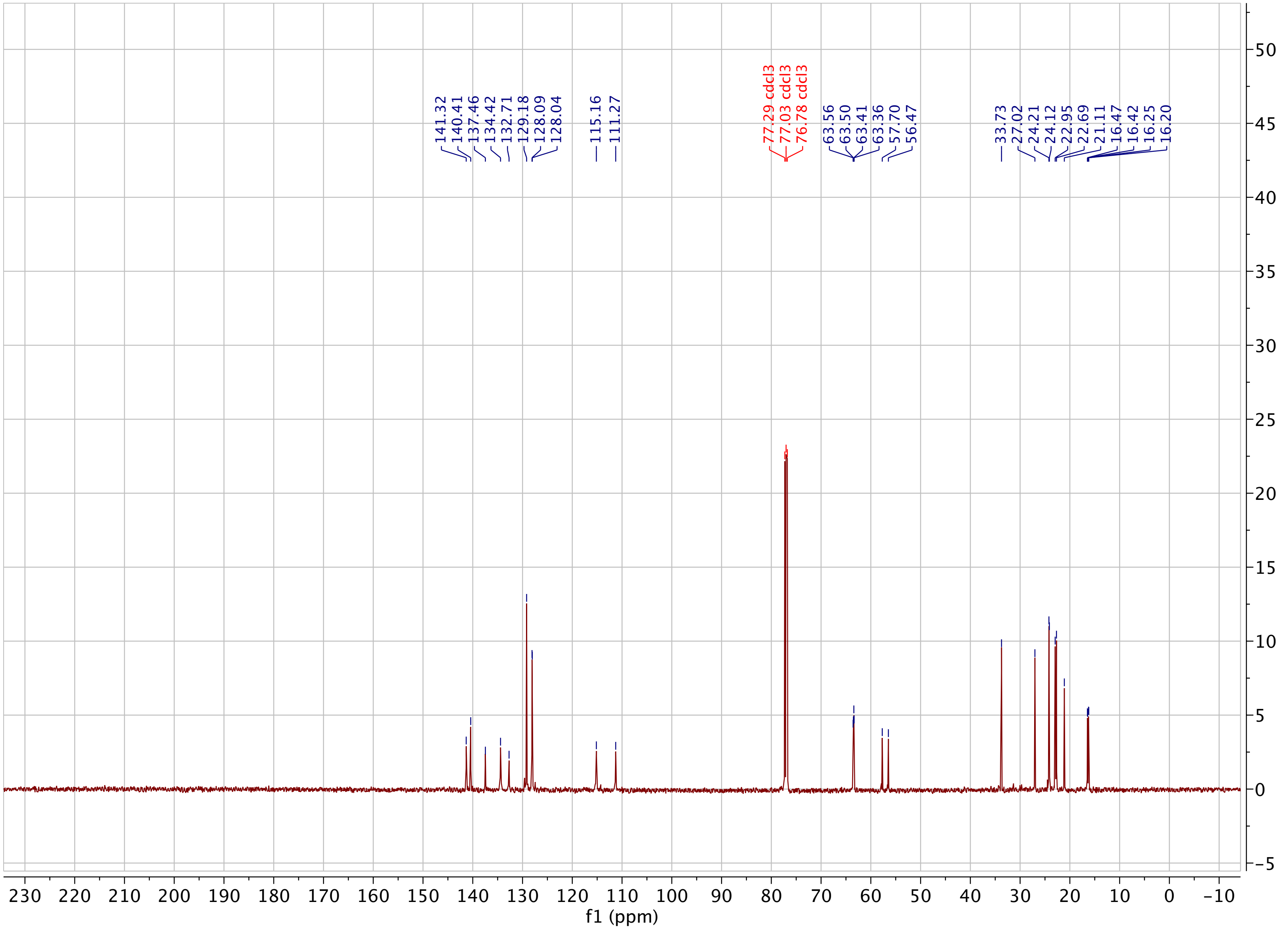
141.32
140.41
137.46
134.42
132.71
129.18
128.09
128.04
115.16
111.27
77.29 cdcl3
77.03 cdcl3
76.78 cdcl3
63.56
63.50
63.41
63.36
57.70
56.47
33.73
27.02
24.21
24.12
22.95
22.69
21.11
16.47
16.42
16.25
16.20
f1 (ppm)

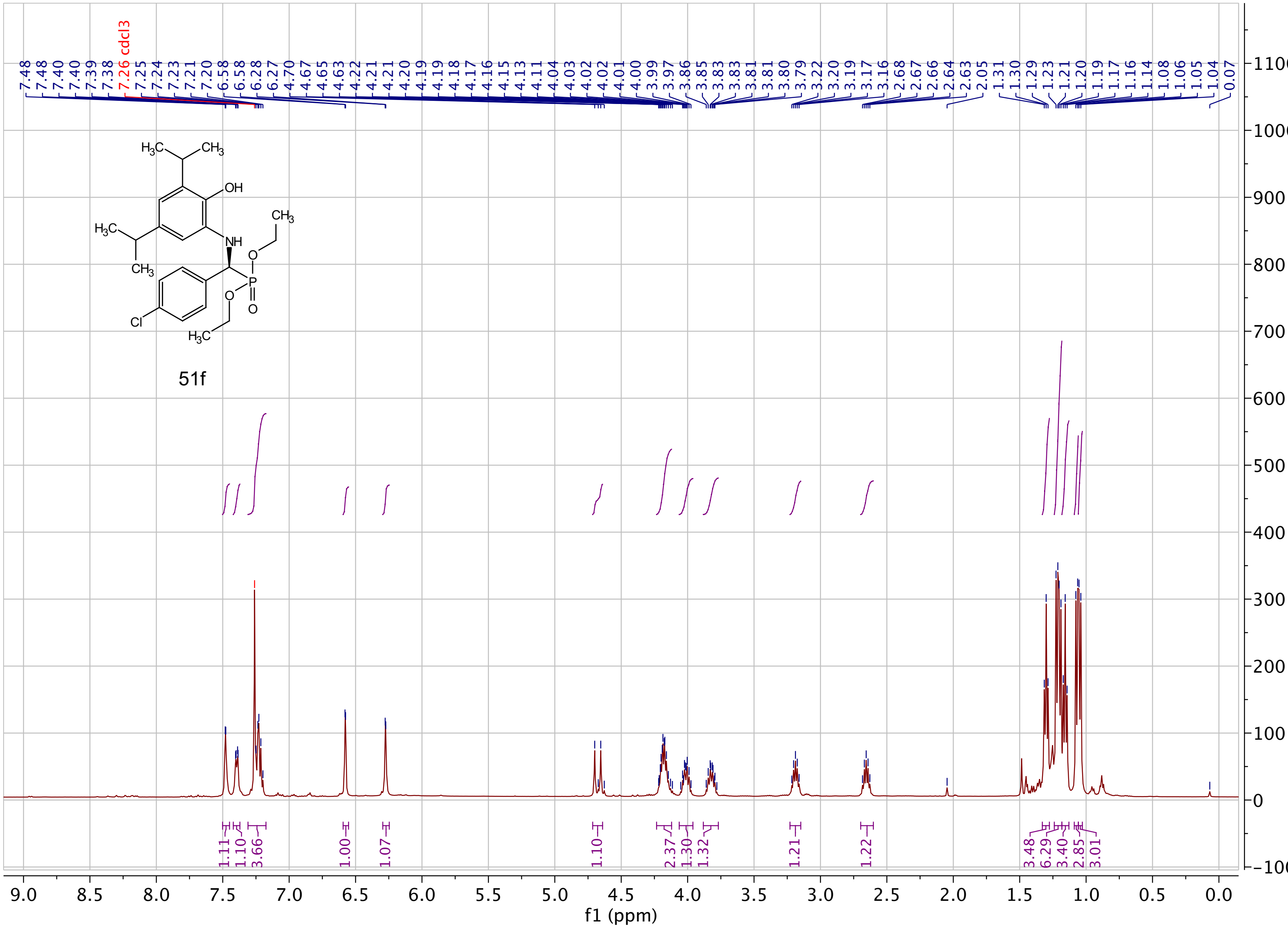

51f
f1 (ppm)

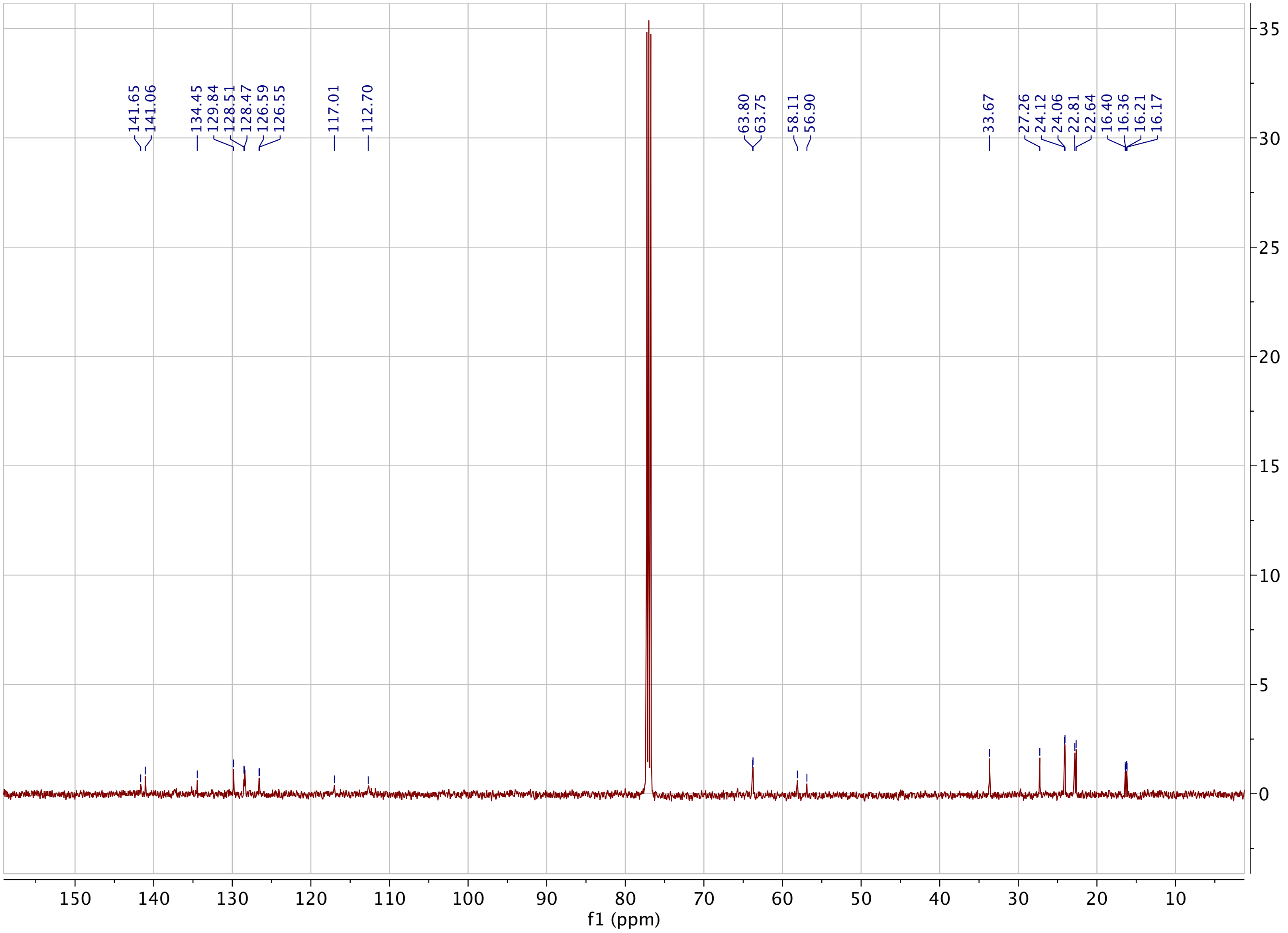
141.65
141.06
134.45
129.84
128.51
128.47
126.59
126.55
117.01
112.70
63.80
63.75
58.11
56.90
33.67
27.26
24.12
24.06
22.81
22.64
16.40
16.36
16.21
16.17
f1 (ppm)

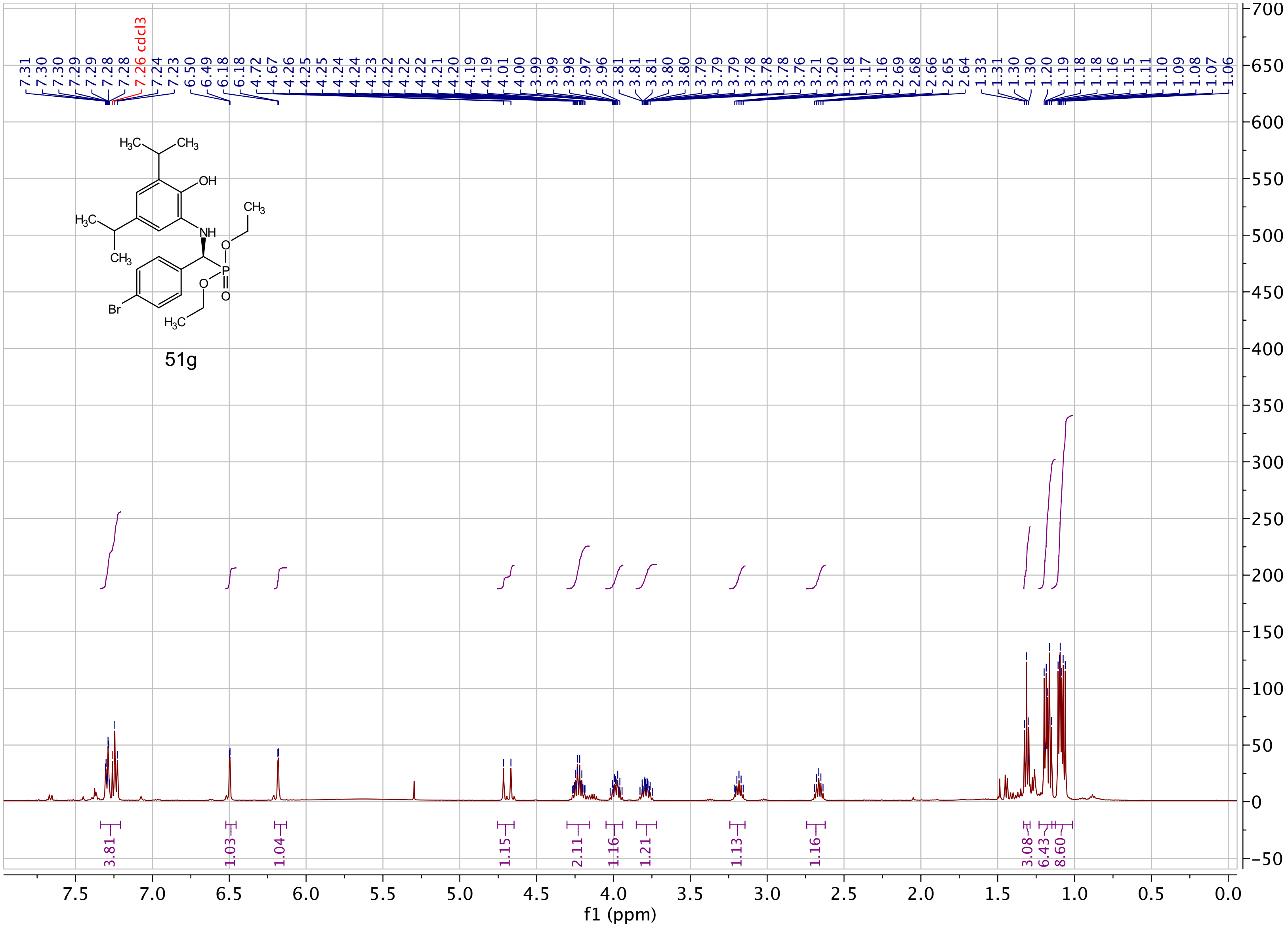

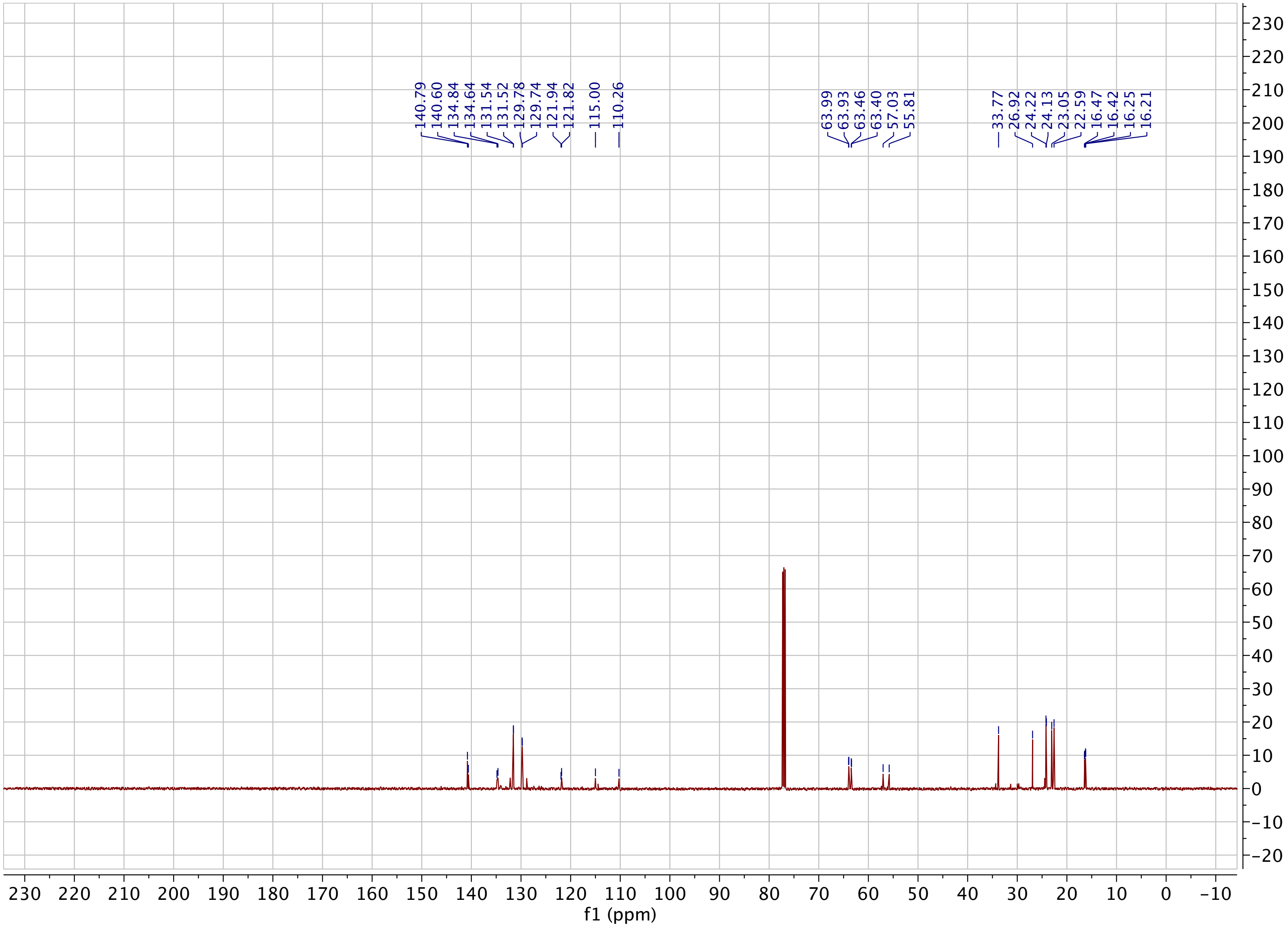
140.79
140.60
134.84
134.64
131.54
131.52
129.78
129.74
121.94
121.82
115.00
110.26
63.99
63.93
63.46
63.40
57.03
55.81
33.77
26.92
24.22
24.13
23.05
22.59
16.47
16.42
16.25
16.21
f1 (ppm)

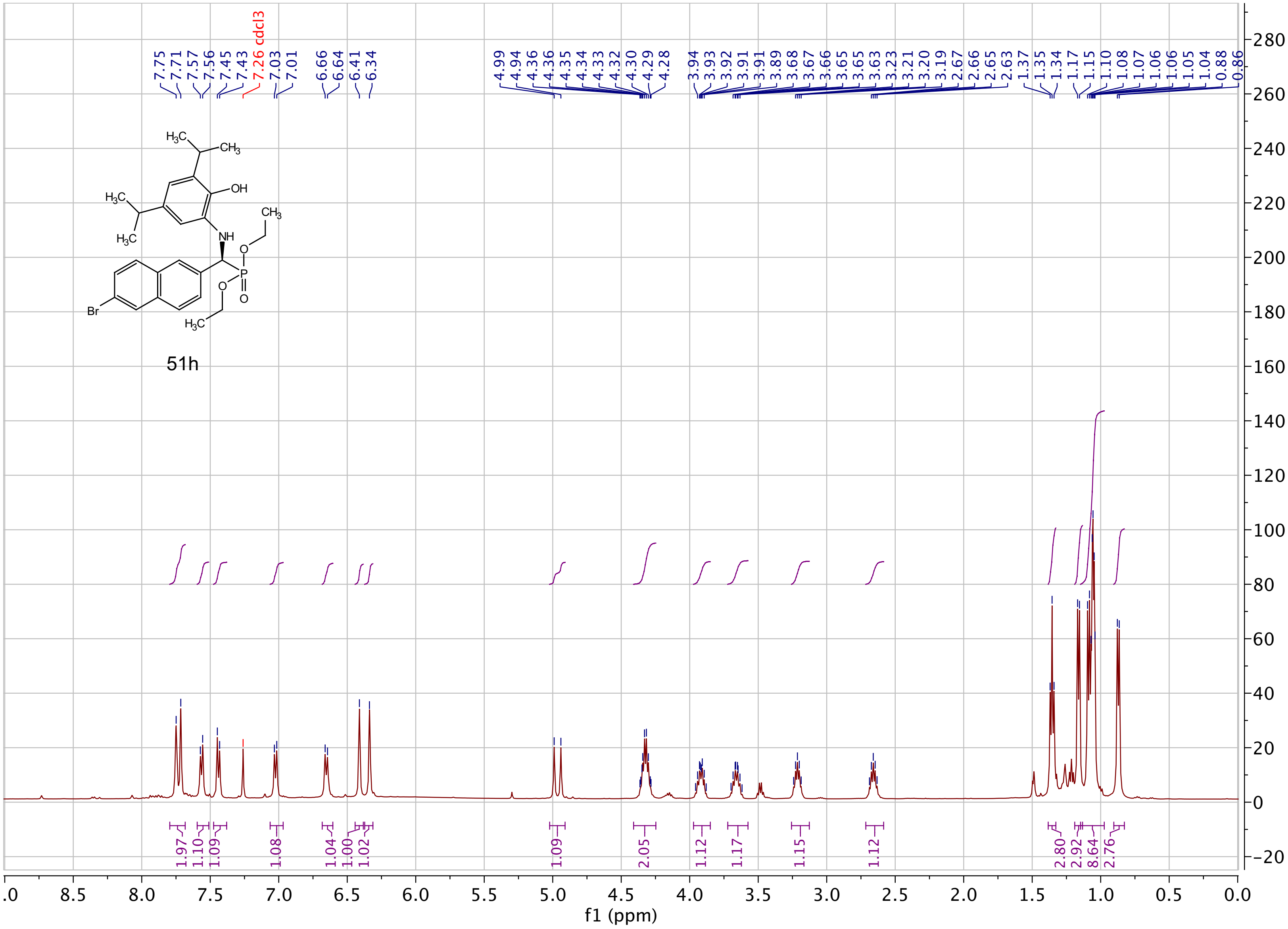
7.75 7.71 7.57 7.56 7.45 7.43 7.26 cdcl3 7.03 7.01 6.66 6.64 6.41 6.34
4.99 4.94 4.36 4.36 4.35 4.34 4.33 4.32 4.30 4.29 4.28
3.94 3.93 3.92 3.91 3.91 3.89 3.68 3.67 3.66 3.65 3.65 3.63 3.23 3.21 3.20 3.19 2.67 2.66 2.65 2.63
1.37 1.35 1.34 1.17 1.15 1.10 1.08 1.07 1.06 1.06 1.05 1.04 0.88 0.86
51h
1.97 1.10 1.09 1.08 1.04 1.00 1.02 1.09 2.05 1.12 1.17 1.15 1.12 2.80 2.92 8.64 2.76
f1 (ppm)

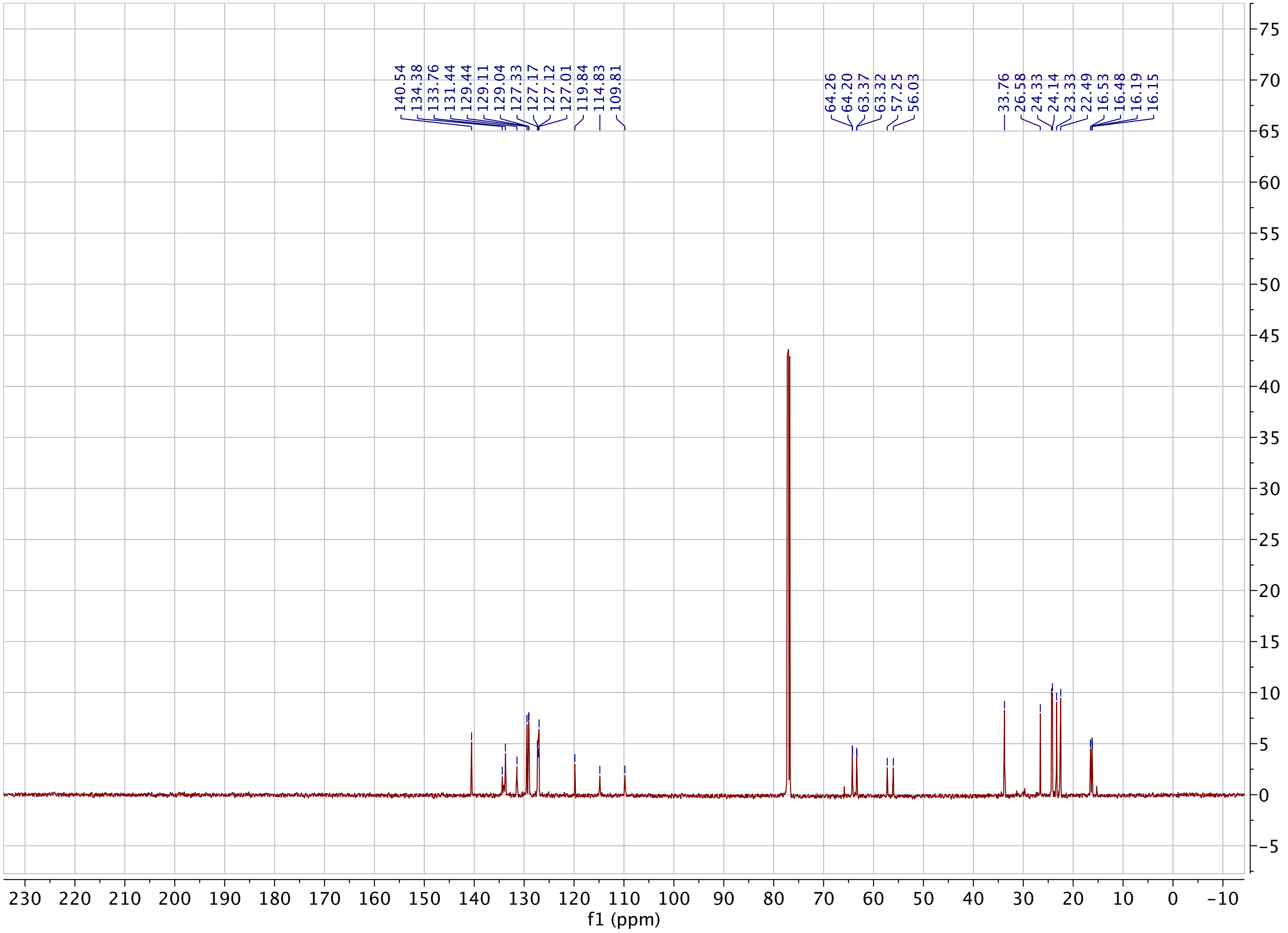
140.54
134.38
133.76
131.44
129.44
129.11
129.04
127.33
127.17
127.12
127.01
119.84
114.83
109.81
64.26
64.20
63.37
63.32
57.25
56.03
33.76
26.58
24.33
24.14
23.33
22.49
16.53
16.48
16.19
16.15
f1 (ppm)

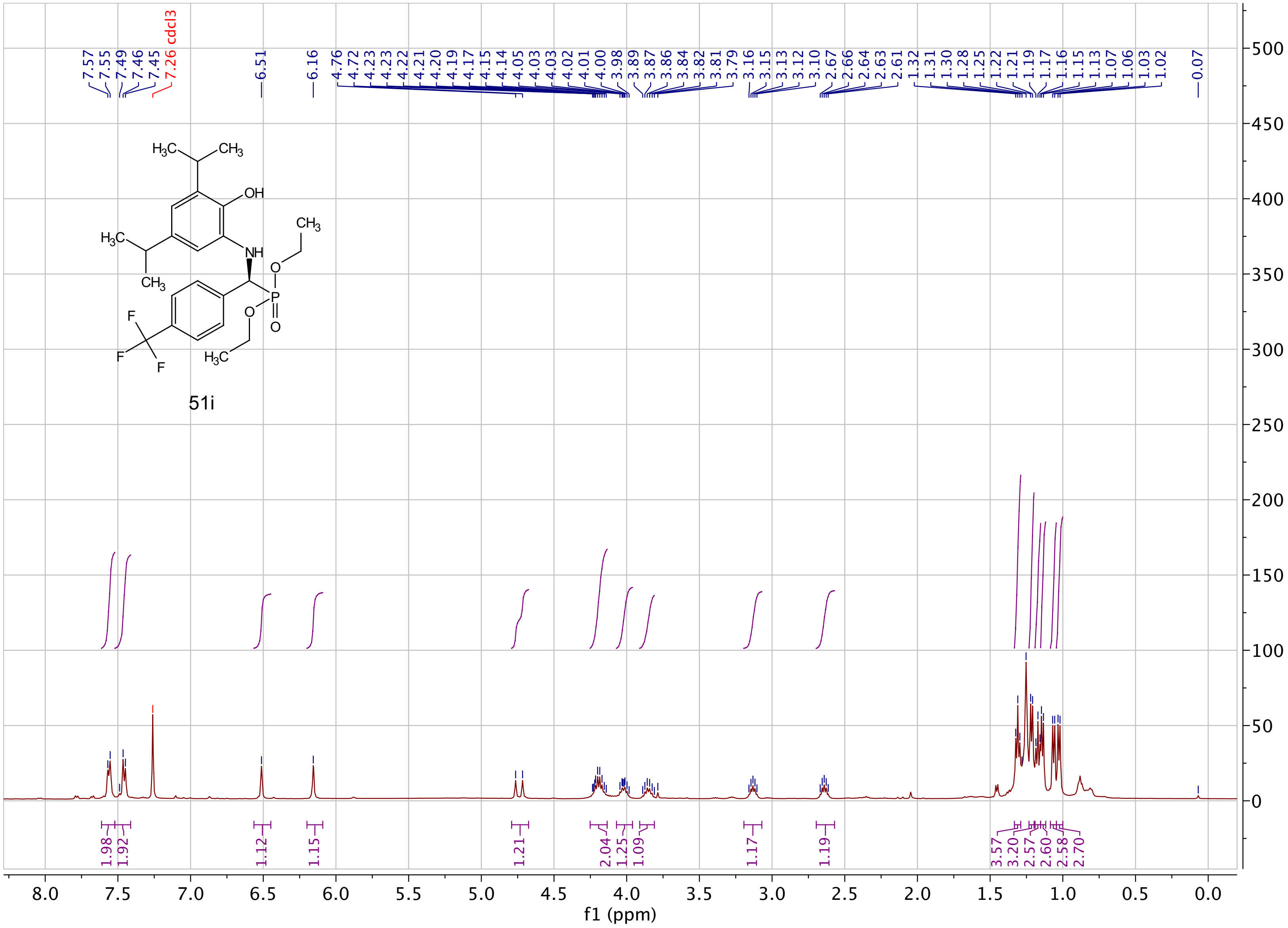

7.57
7.55
7.49
7.46
7.45
7.26 cdcl3
6.51
6.16
4.76
4.72
4.23
4.23
4.22
4.21
4.20
4.19
4.17
4.15
4.14
4.05
4.03
4.03
4.02
4.01
4.00
3.98
3.89
3.87
3.86
3.84
3.82
3.81
3.79
3.16
3.15
3.13
3.12
3.10
2.67
2.66
2.64
2.63
2.61
1.32
1.31
1.30
1.28
1.25
1.22
1.21
1.19
1.17
1.16
1.15
1.13
1.07
1.06
1.03
1.02
0.07
H3C
CH3
OH
H3C
NH
CH3
CH3
O
P
O
O
F
F
F
H3C
51i
1.98
1.92
1.12
1.15
1.21
2.04
1.25
1.09
1.17
1.19
3.57
3.20
2.57
2.60
2.58
2.70
f1 (ppm)

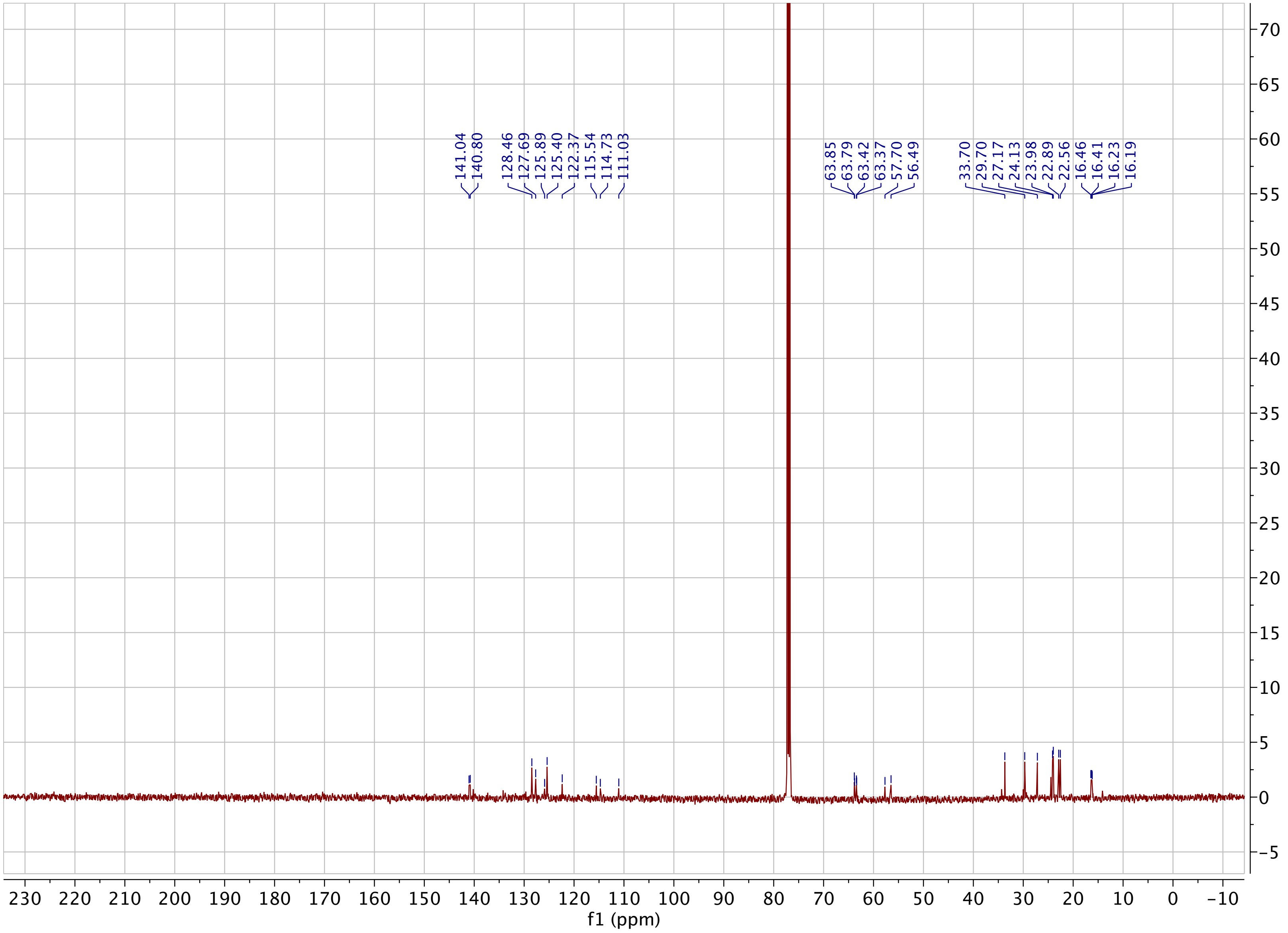
141.04
140.80
128.46
127.69
125.89
125.40
122.37
115.54
114.73
111.03
63.85
63.79
63.42
63.37
57.70
56.49
33.70
29.70
27.17
24.13
23.98
22.89
22.56
16.46
16.41
16.23
16.19
f1 (ppm)

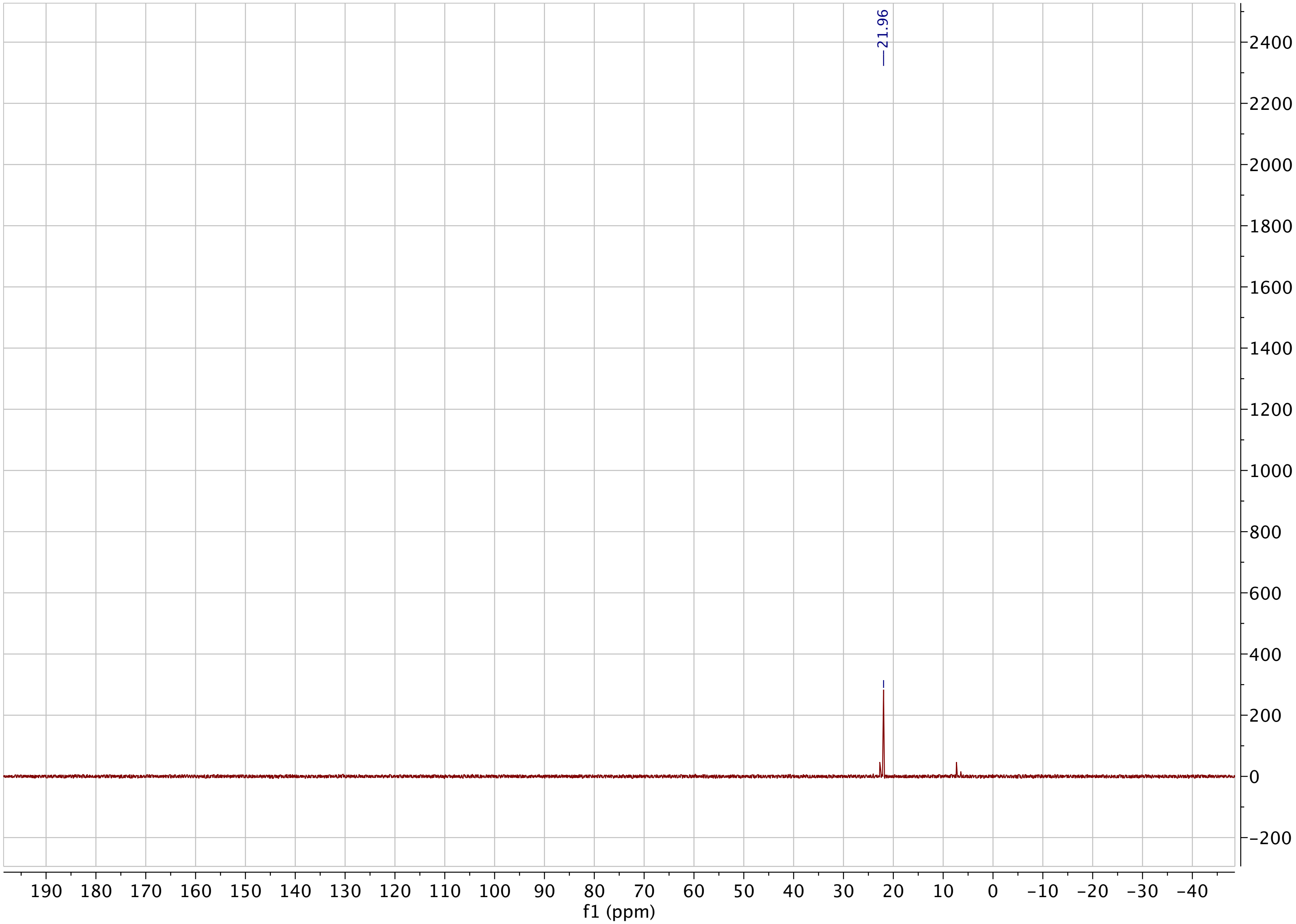

21.96
f1 (ppm)

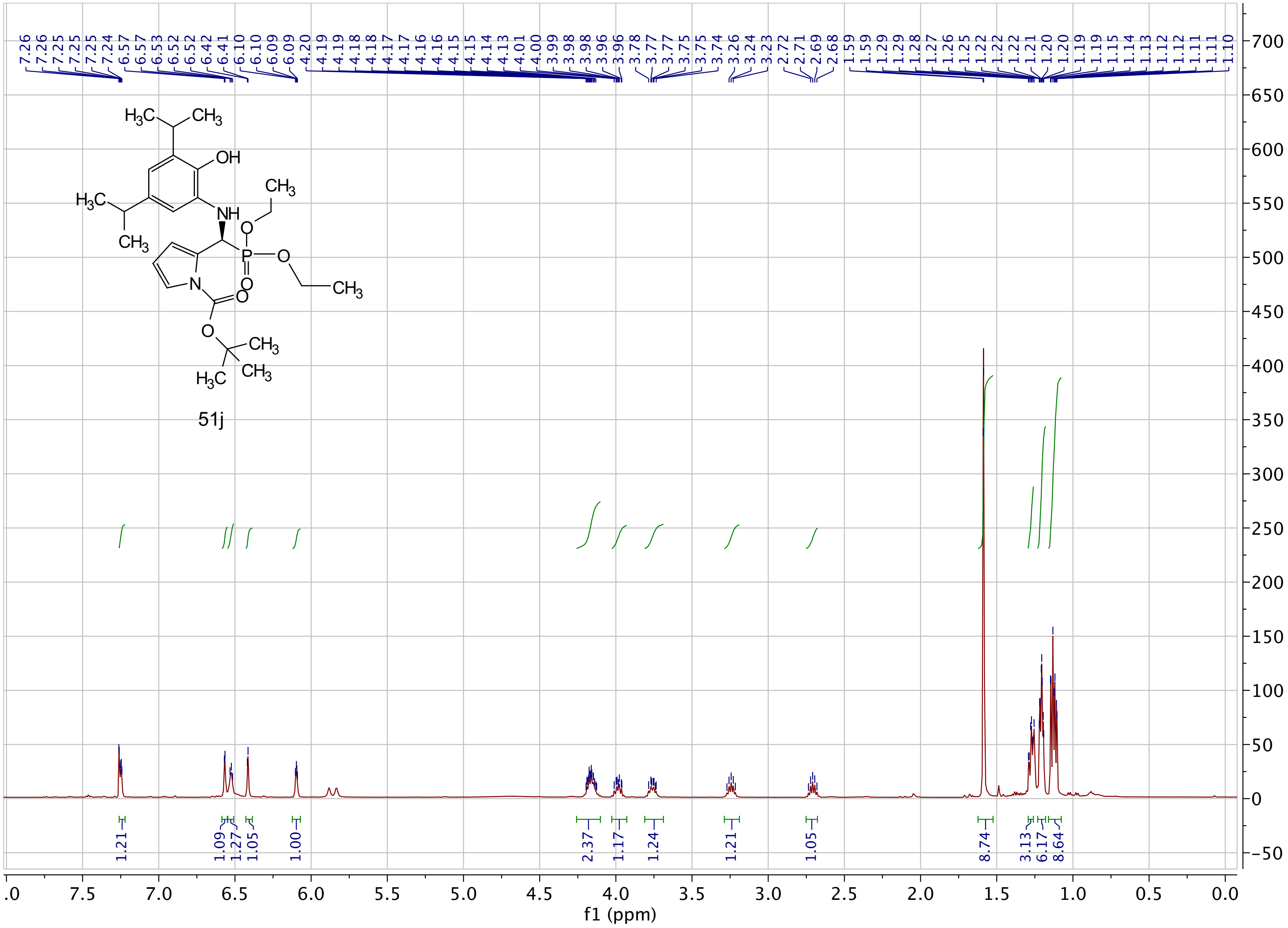

51j

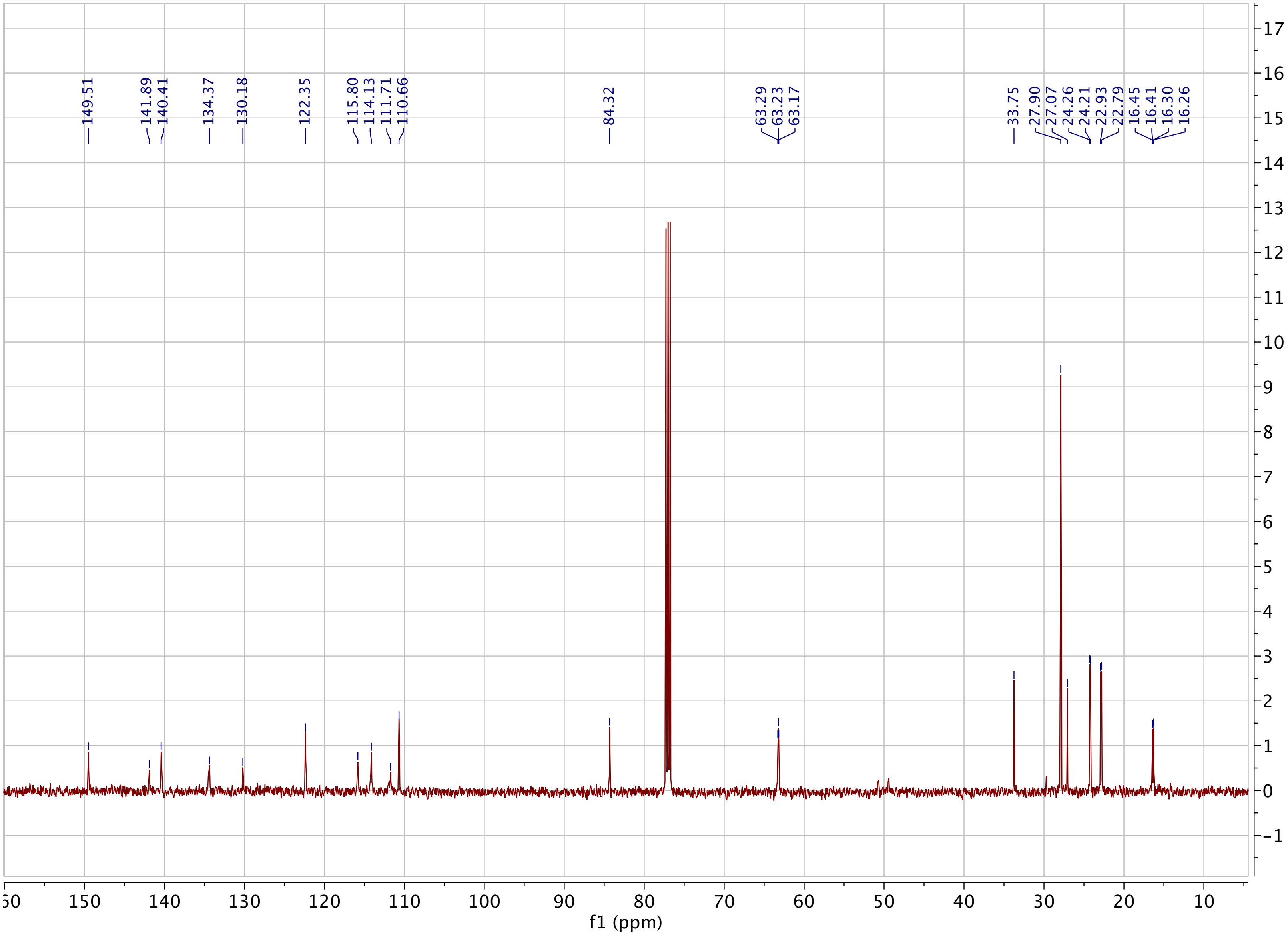

149.51
141.89
140.41
134.37
130.18
122.35
115.80
114.13
111.71
110.66
84.32
63.29
63.23
63.17
33.75
27.90
27.07
24.26
24.21
22.93
22.79
16.45
16.41
16.30
16.26
f1 (ppm)

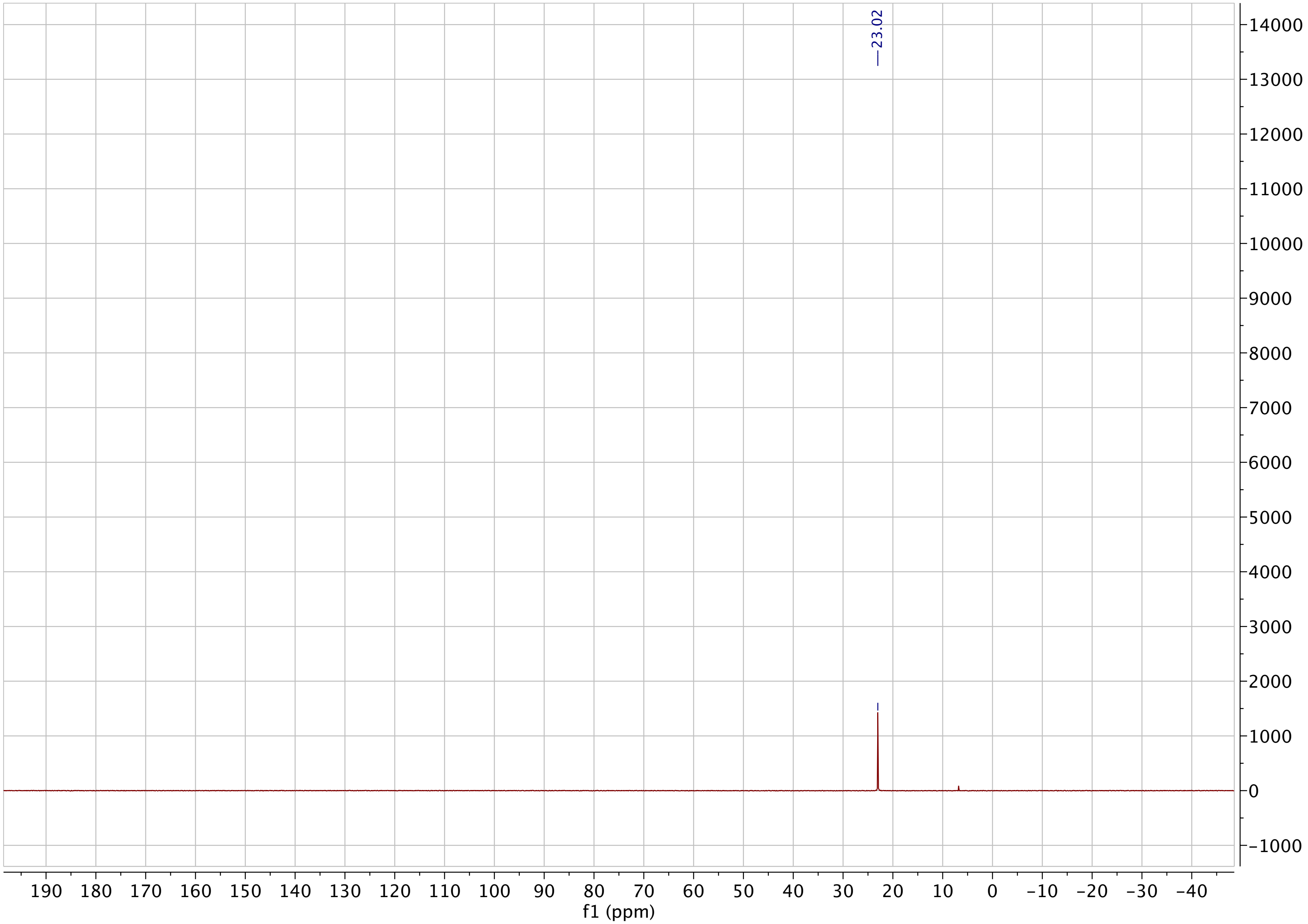

23.02
14000
13000
12000
11000
10000
9000
8000
7000
6000
5000
4000
3000
2000
1000
0
−1000
190 180 170 160 150 140 130 120 110 100 90 80 70 60 50 40 30 20 10 0 −10 −20 −30 −40
f1 (ppm)

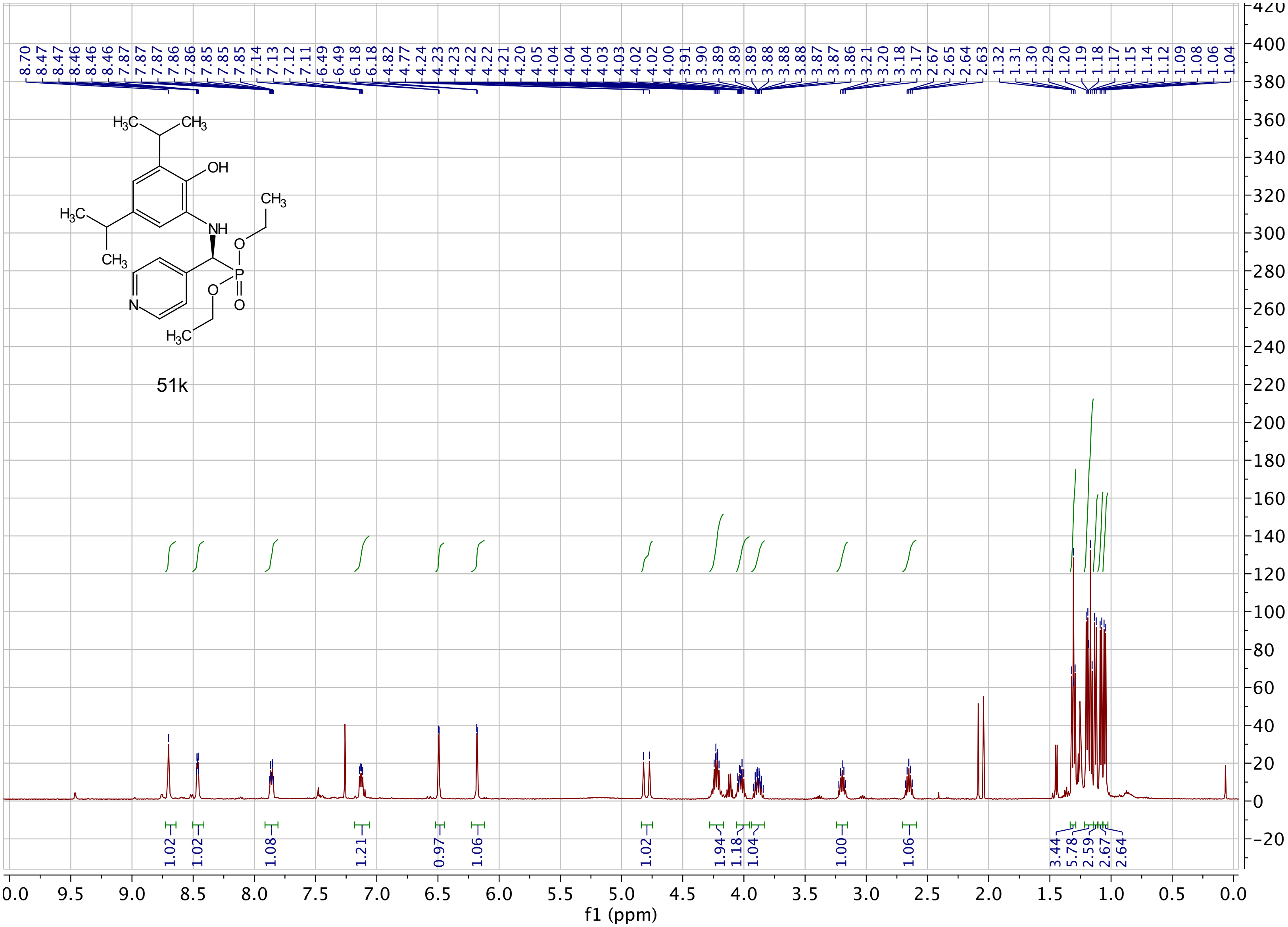

51k

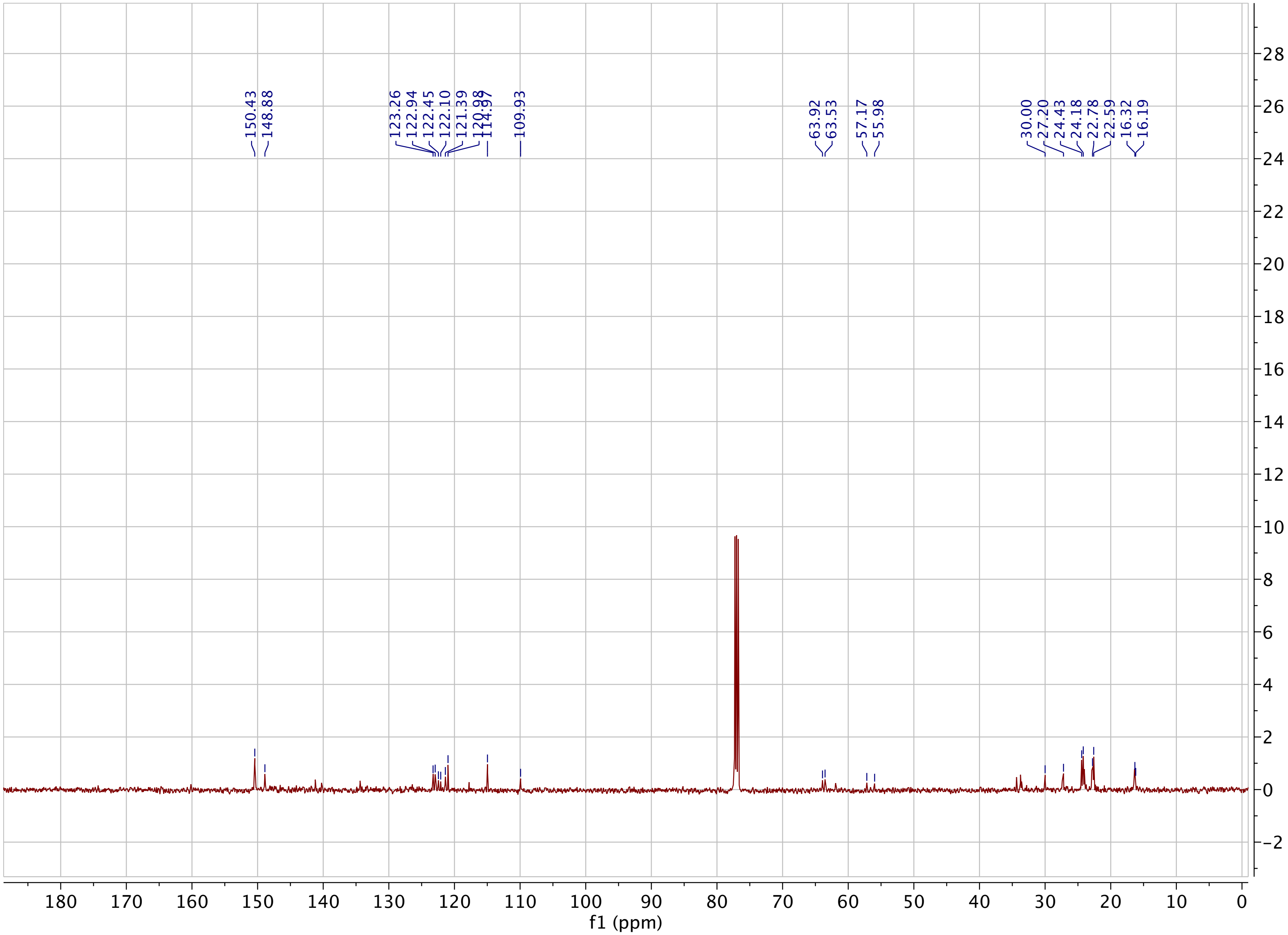
150.43
148.88
123.26
122.94
122.45
122.10
121.39
120.98
114.97
109.93
63.92
63.53
57.17
55.98
30.00
27.20
24.43
24.18
22.78
22.59
16.32
16.19
f1 (ppm)

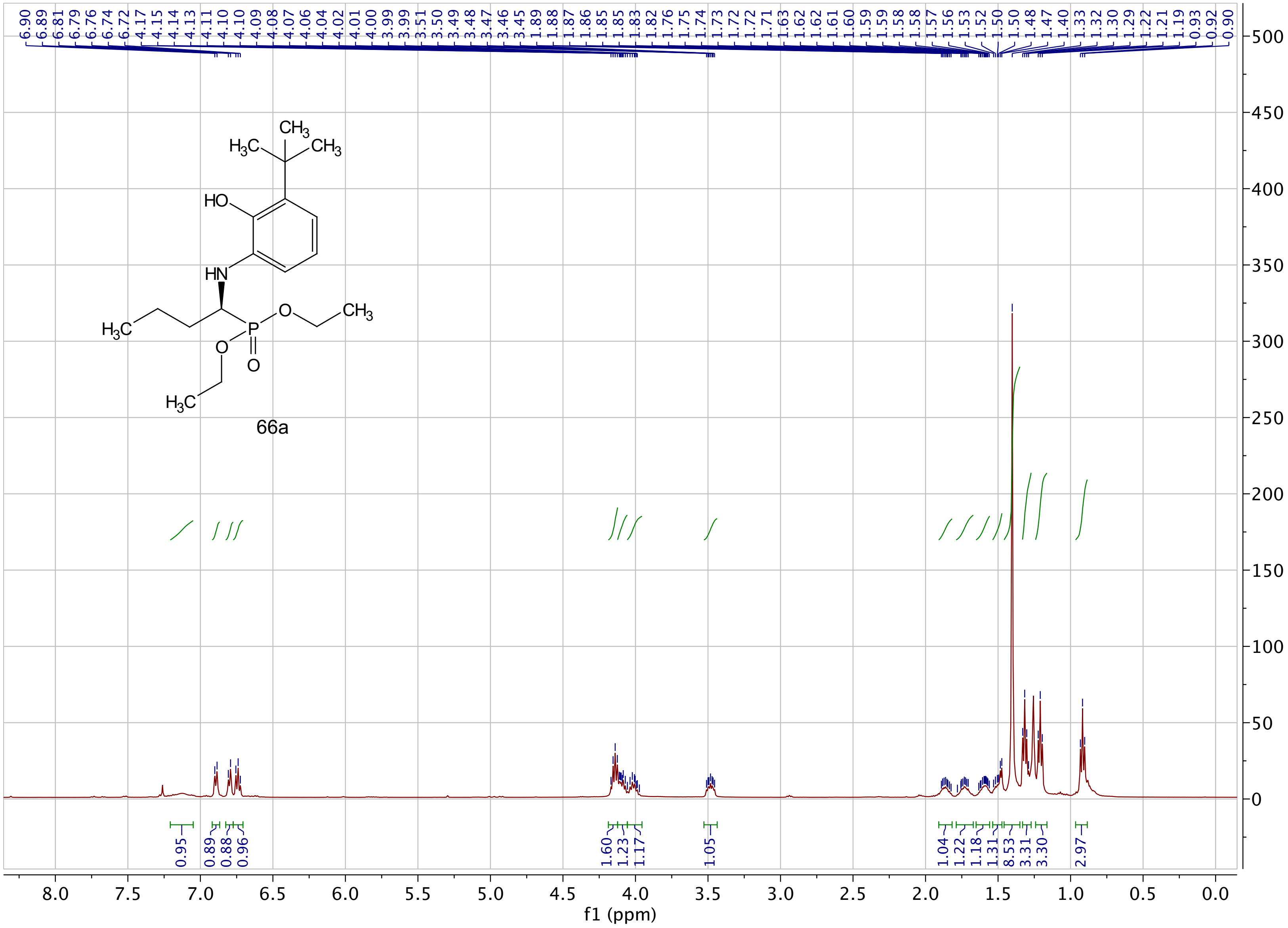

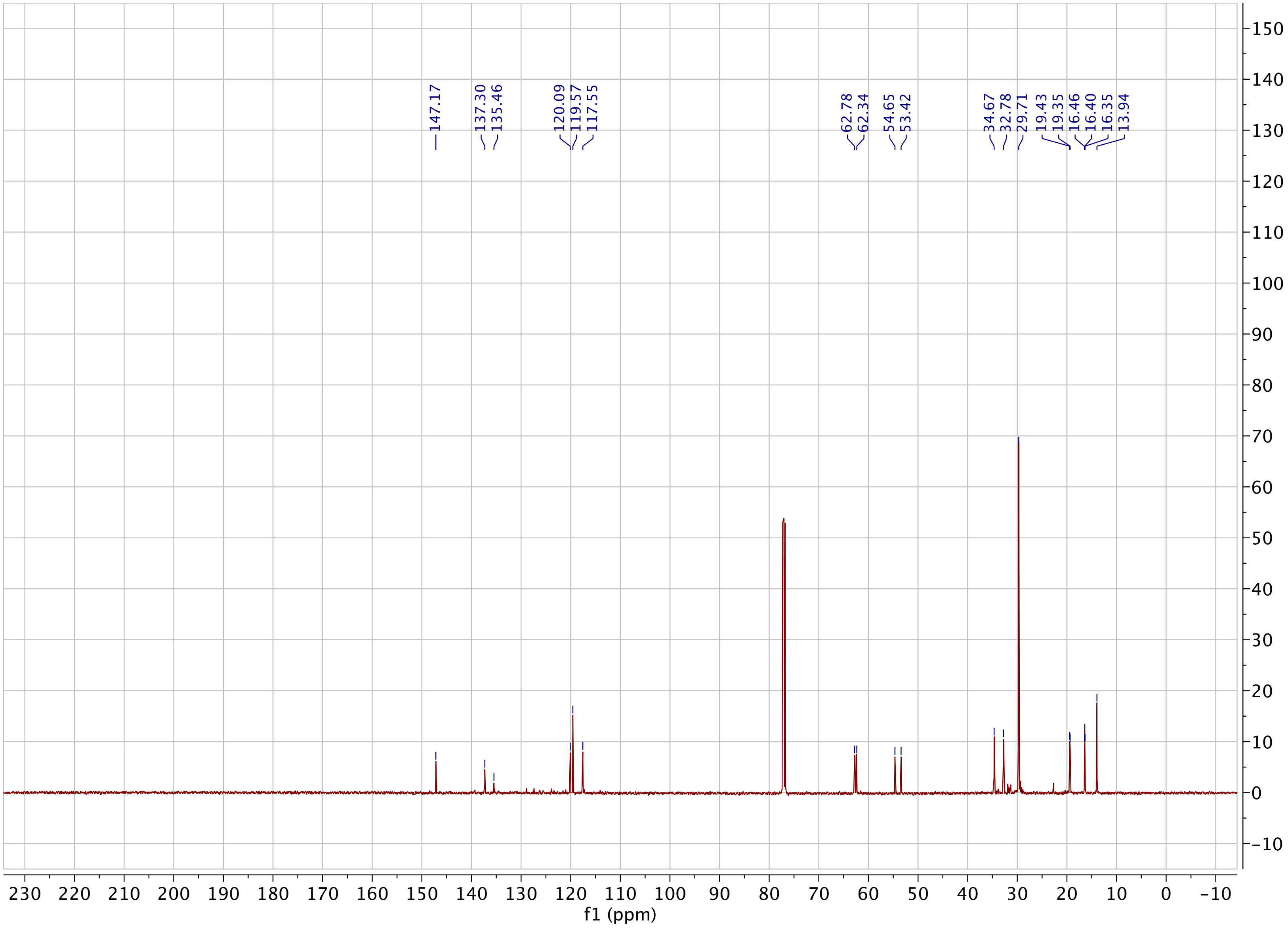
147.17
137.30
135.46
120.09
119.57
117.55
62.78
62.34
54.65
53.42
34.67
32.78
29.71
19.43
19.35
16.46
16.40
16.35
13.94
f1 (ppm)

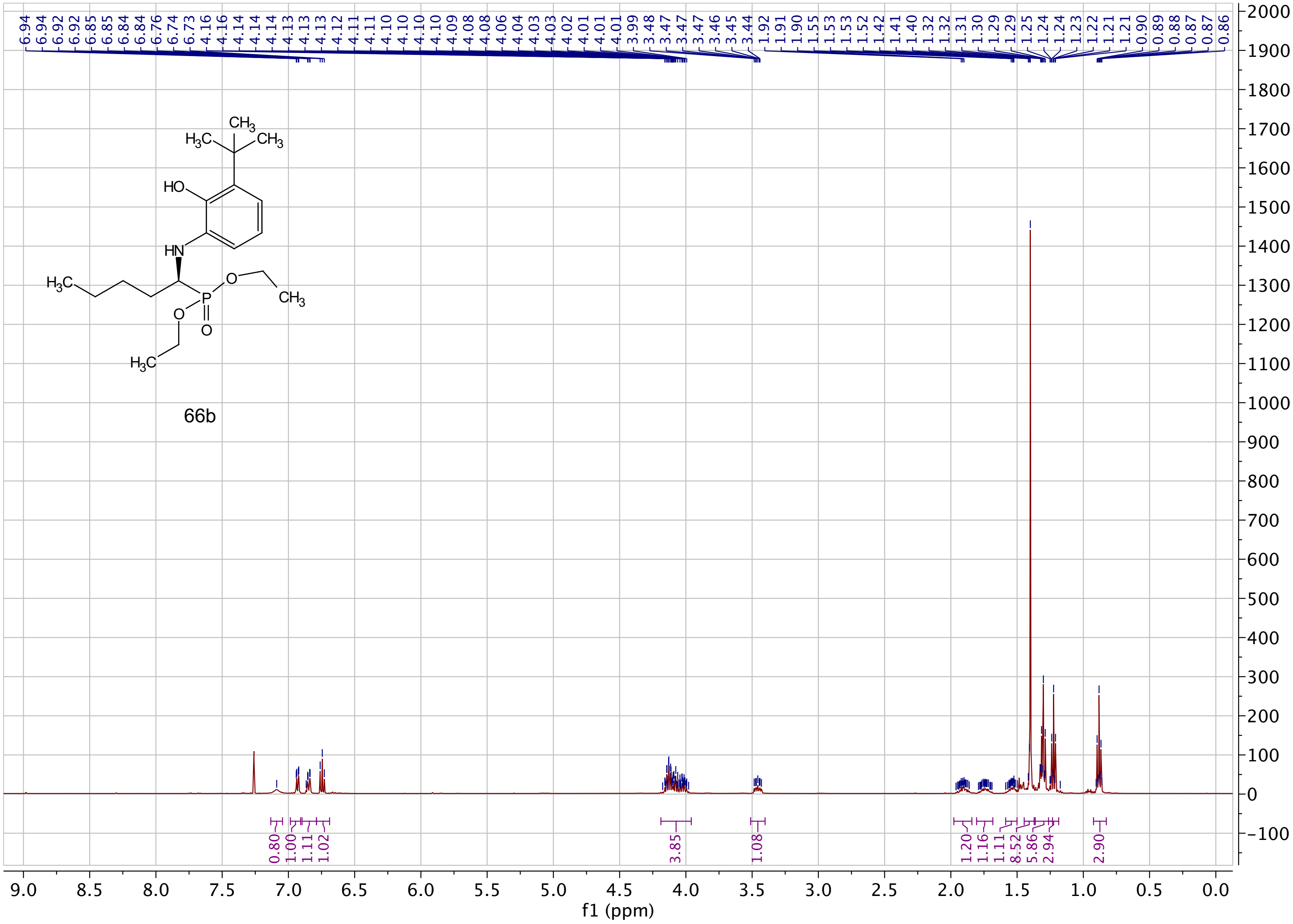
66b
f1 (ppm)

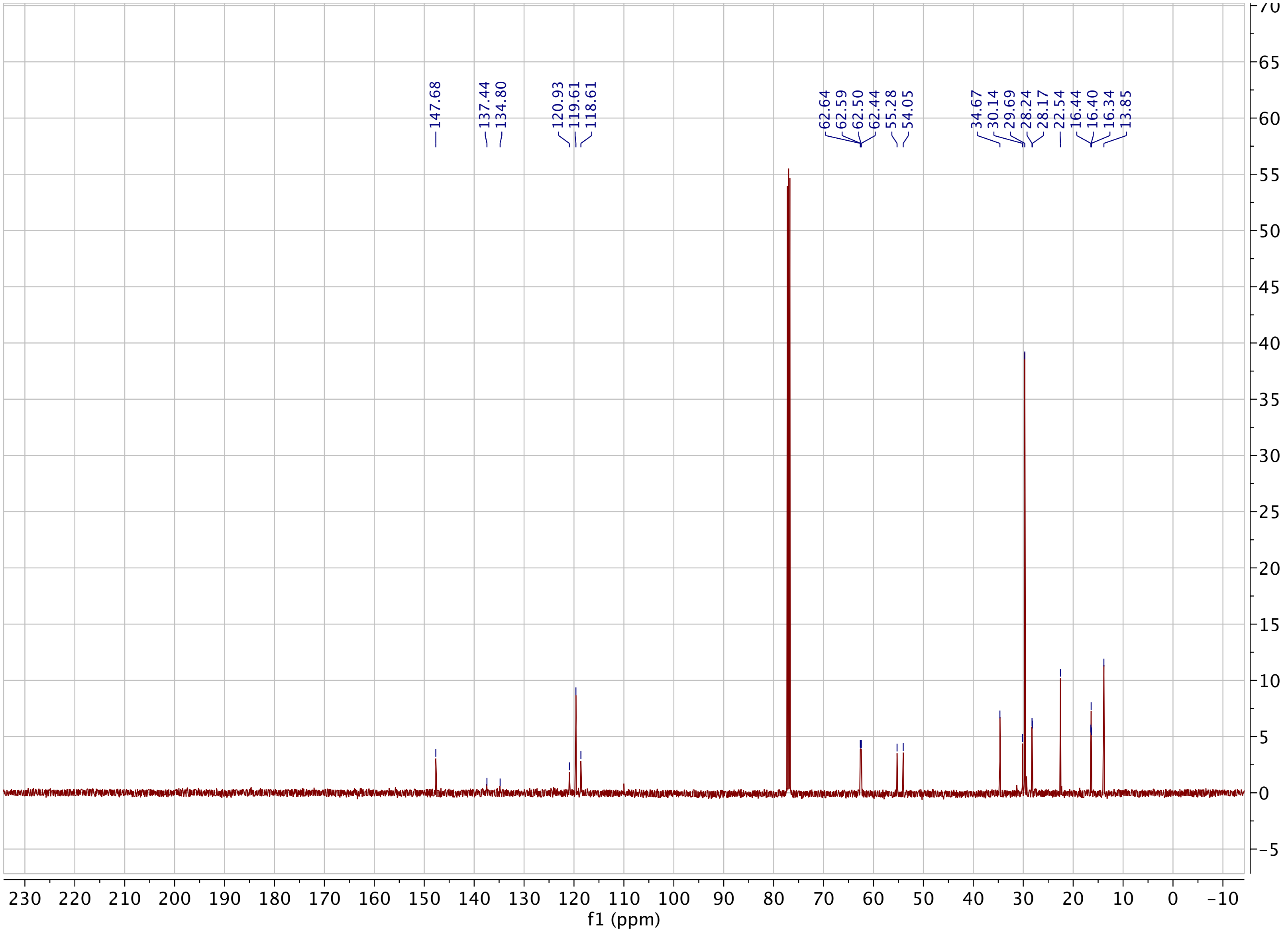

147.68
137.44
134.80
120.93
119.61
118.61
62.64
62.59
62.50
62.44
55.28
54.05
34.67
30.14
29.69
28.24
28.17
22.54
16.44
16.40
16.34
13.85
f1 (ppm)

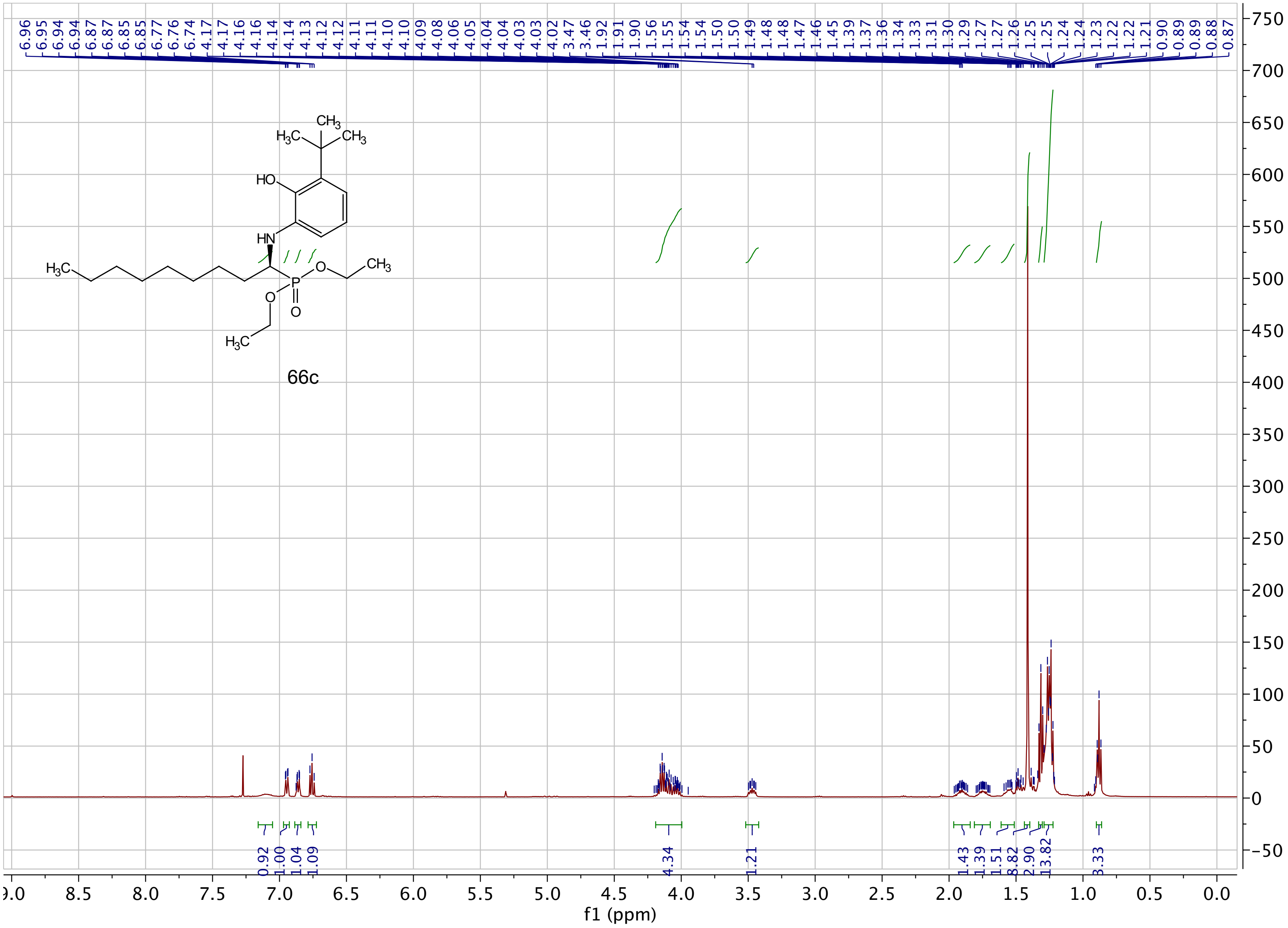
66c
f1 (ppm)

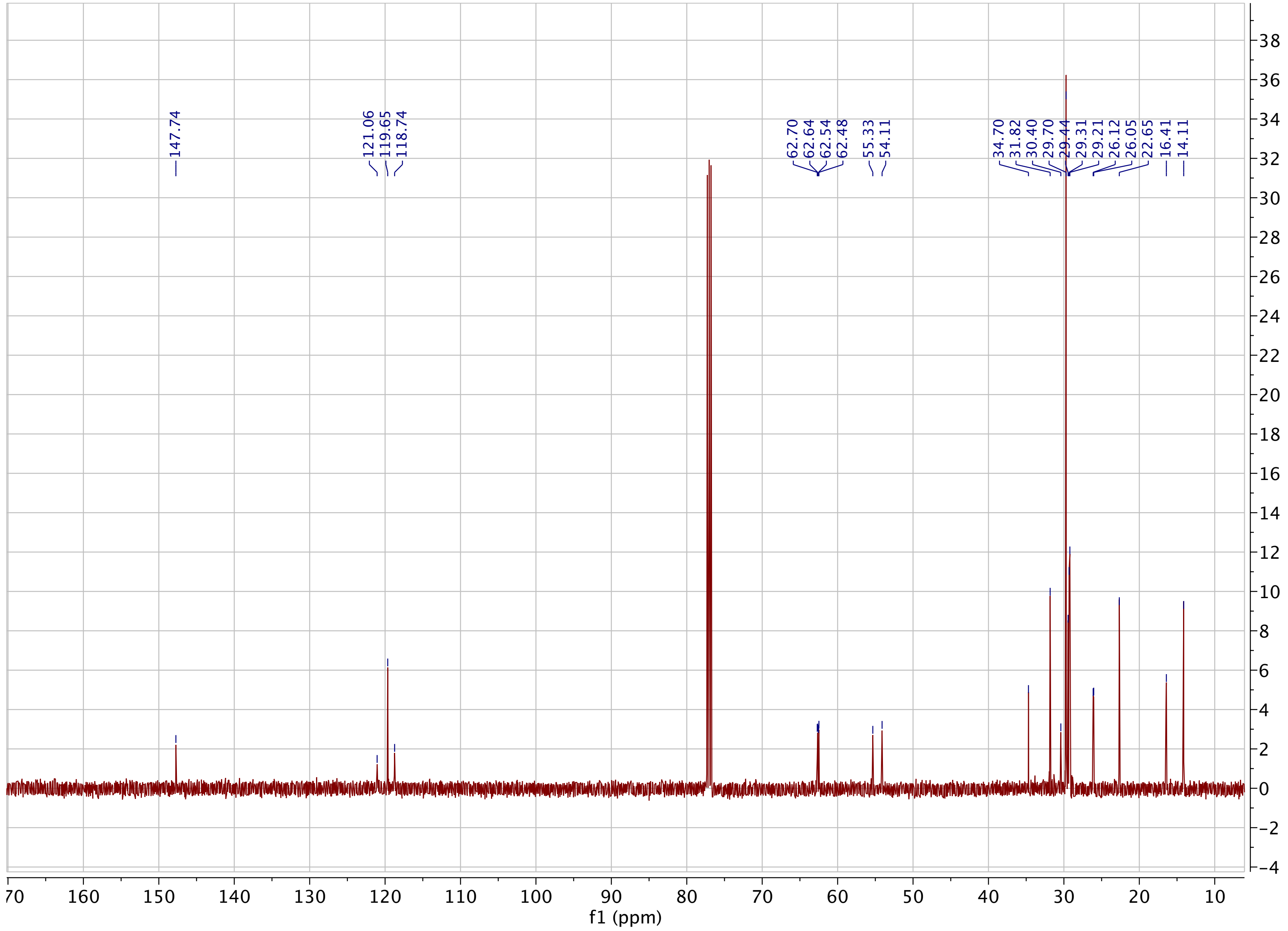

147.74
121.06
119.65
118.74
62.70
62.64
62.54
62.48
55.33
54.11
34.70
31.82
30.40
29.70
29.44
29.31
29.21
26.12
26.05
22.65
16.41
14.11
f1 (ppm)

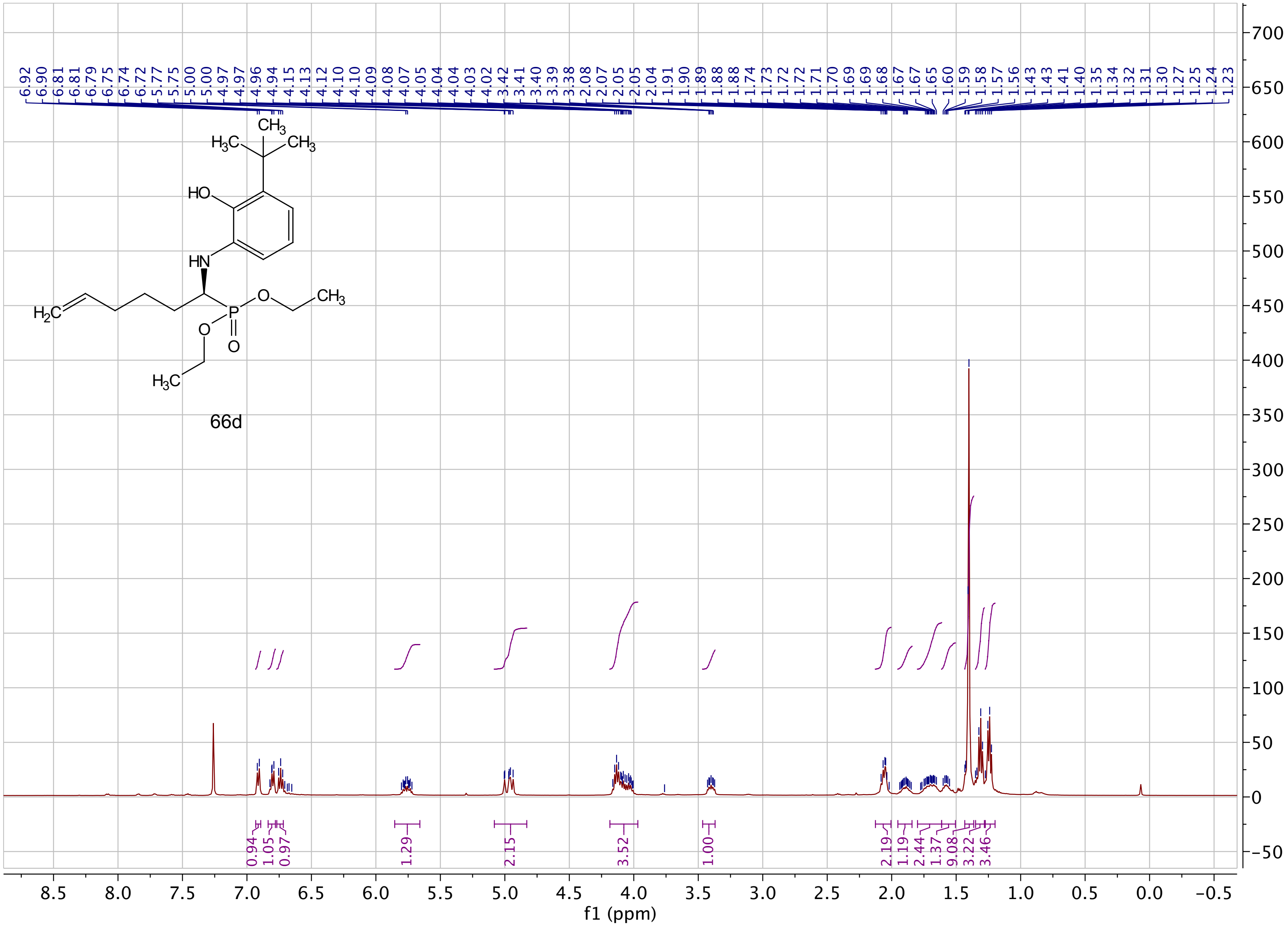
66d
f1 (ppm)

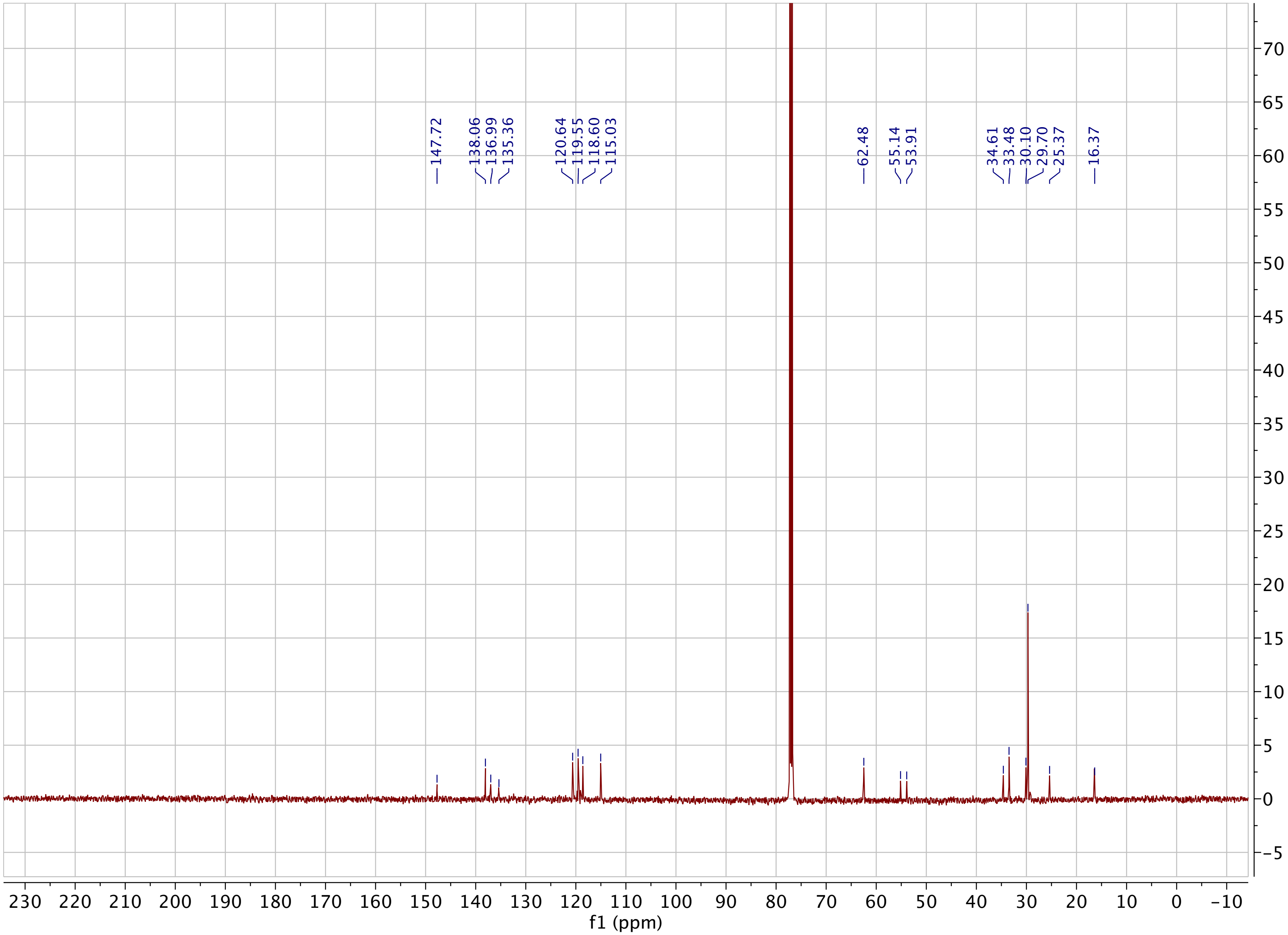
147.72
138.06
136.99
135.36
120.64
119.55
118.60
115.03
62.48
55.14
53.91
34.61
33.48
30.10
29.70
25.37
16.37
f1 (ppm)

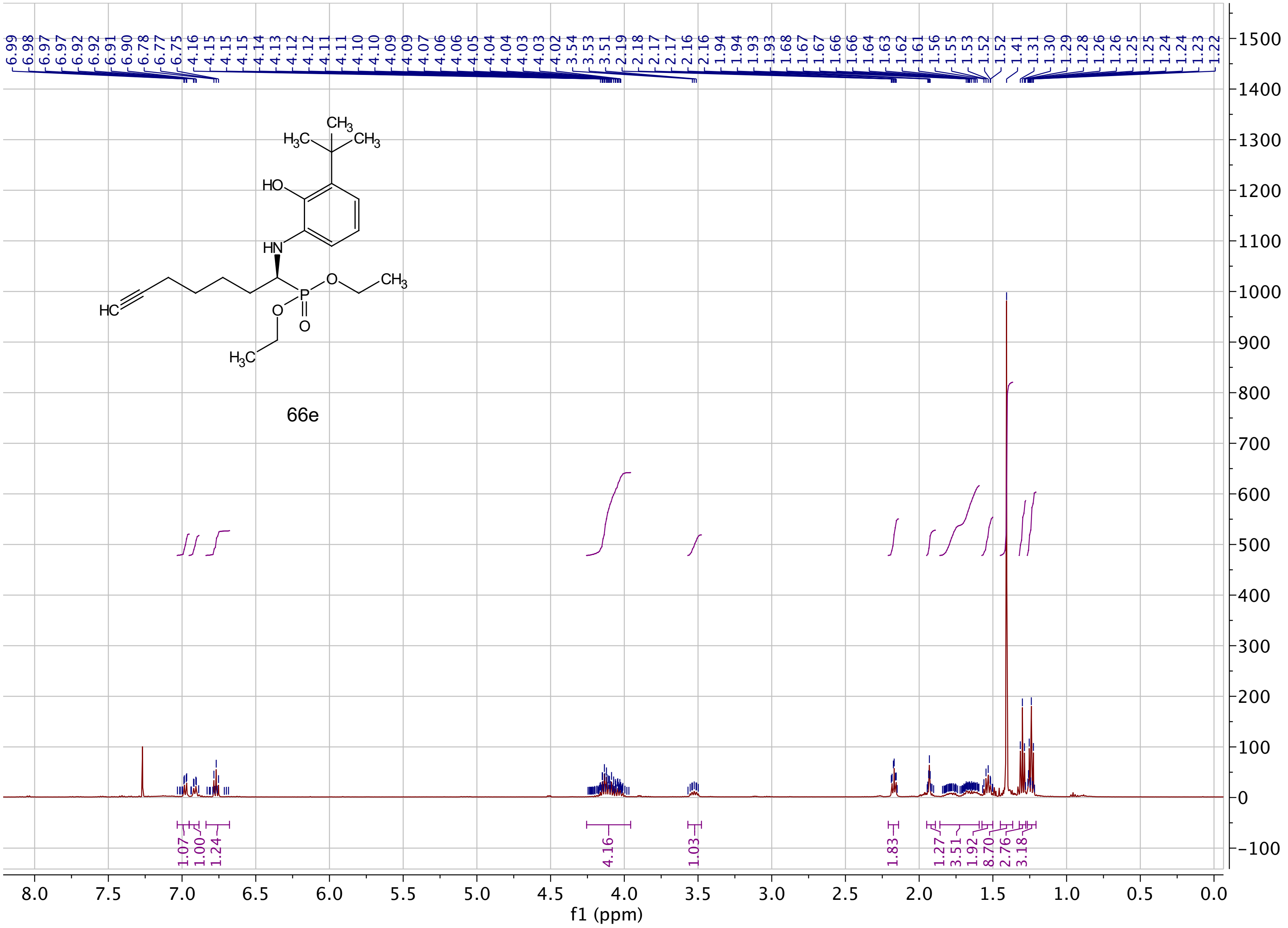
6.99 6.98 6.97 6.97 6.92 6.92 6.91 6.90 6.78 6.77 6.75 4.16 4.15 4.15 4.15 4.14 4.13 4.12 4.12 4.11 4.11 4.10 4.10 4.09 4.09 4.07 4.06 4.06 4.05 4.04 4.04 4.03 4.03 4.02 3.54 3.53 3.51 2.19 2.18 2.17 2.17 2.16 2.16 1.94 1.94 1.93 1.93 1.68 1.67 1.67 1.66 1.66 1.64 1.63 1.62 1.61 1.56 1.55 1.53 1.52 1.52 1.41 1.31 1.30 1.29 1.28 1.26 1.26 1.25 1.25 1.24 1.24 1.23 1.22
CH3
H3C
CH3
HO
HN
HC
P
O
CH3
O
O
H3C
66e
1.07
1.00
1.24
4.16
1.03
1.83
1.27
3.51
1.92
8.70
2.76
3.18
f1 (ppm)

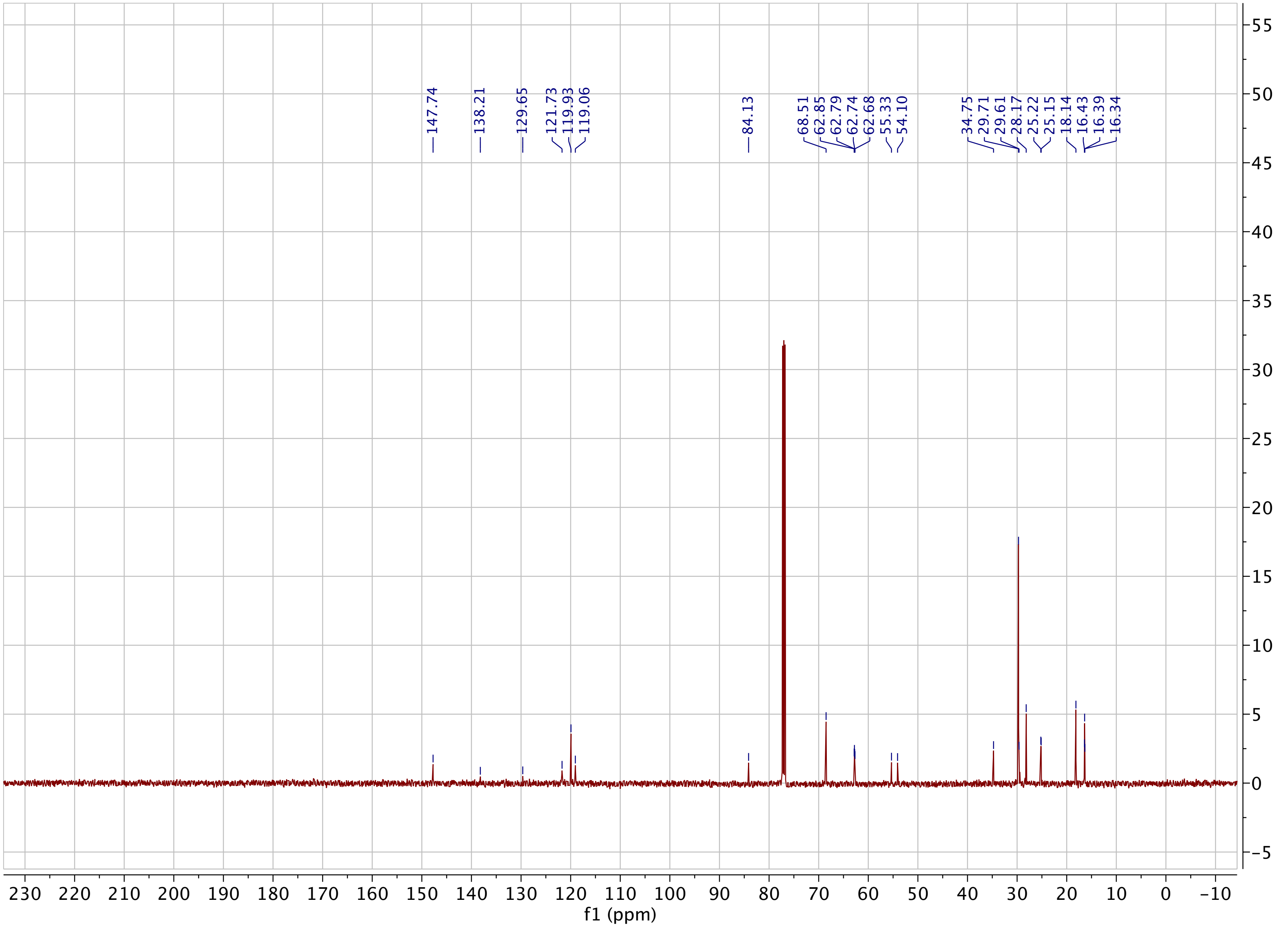
147.74
138.21
129.65
121.73
119.93
119.06
84.13
68.51
62.85
62.79
62.74
62.68
55.33
54.10
34.75
29.71
29.61
28.17
25.22
25.15
18.14
16.43
16.39
16.34
f1 (ppm)

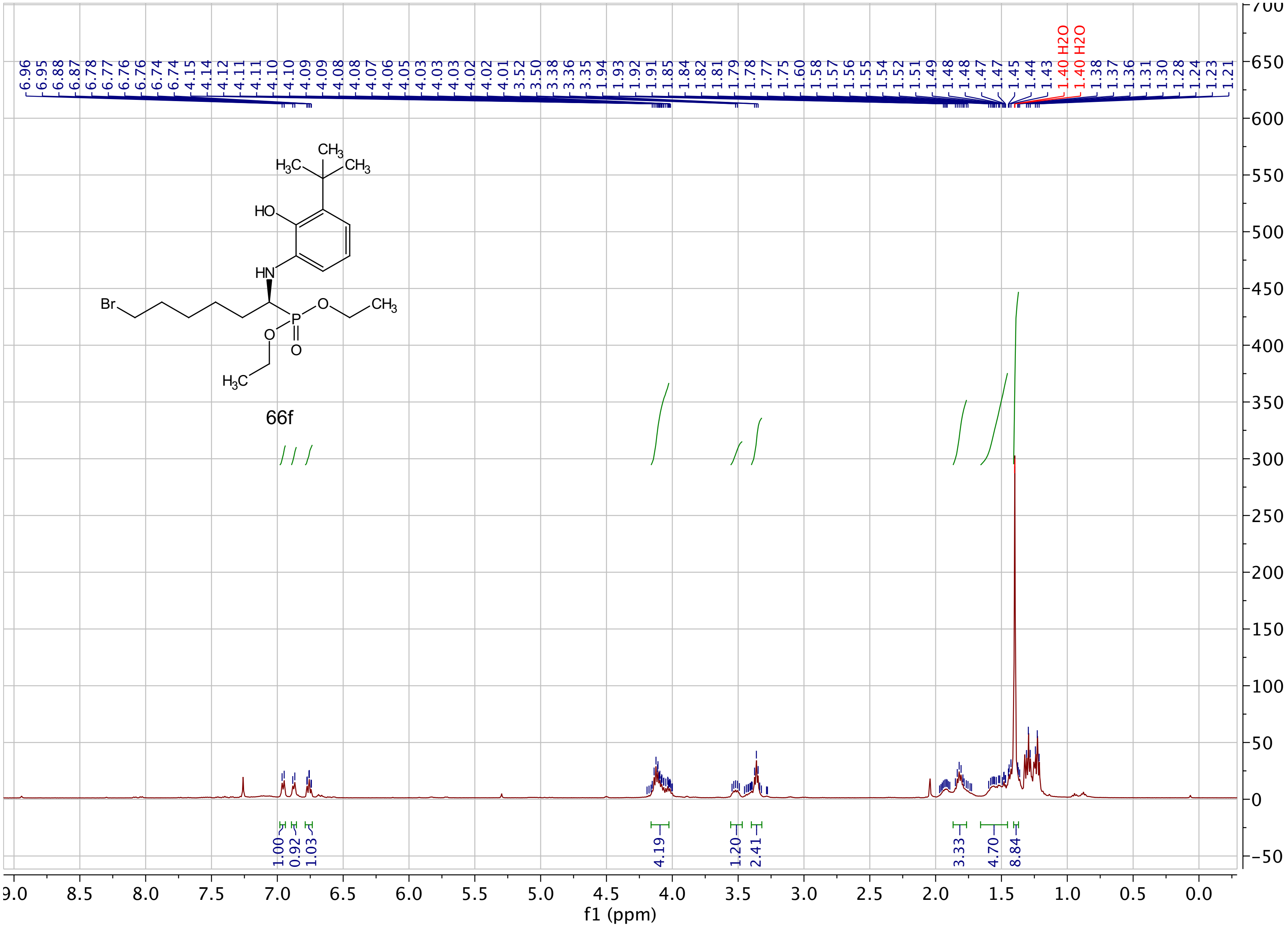
66f
f1 (ppm)

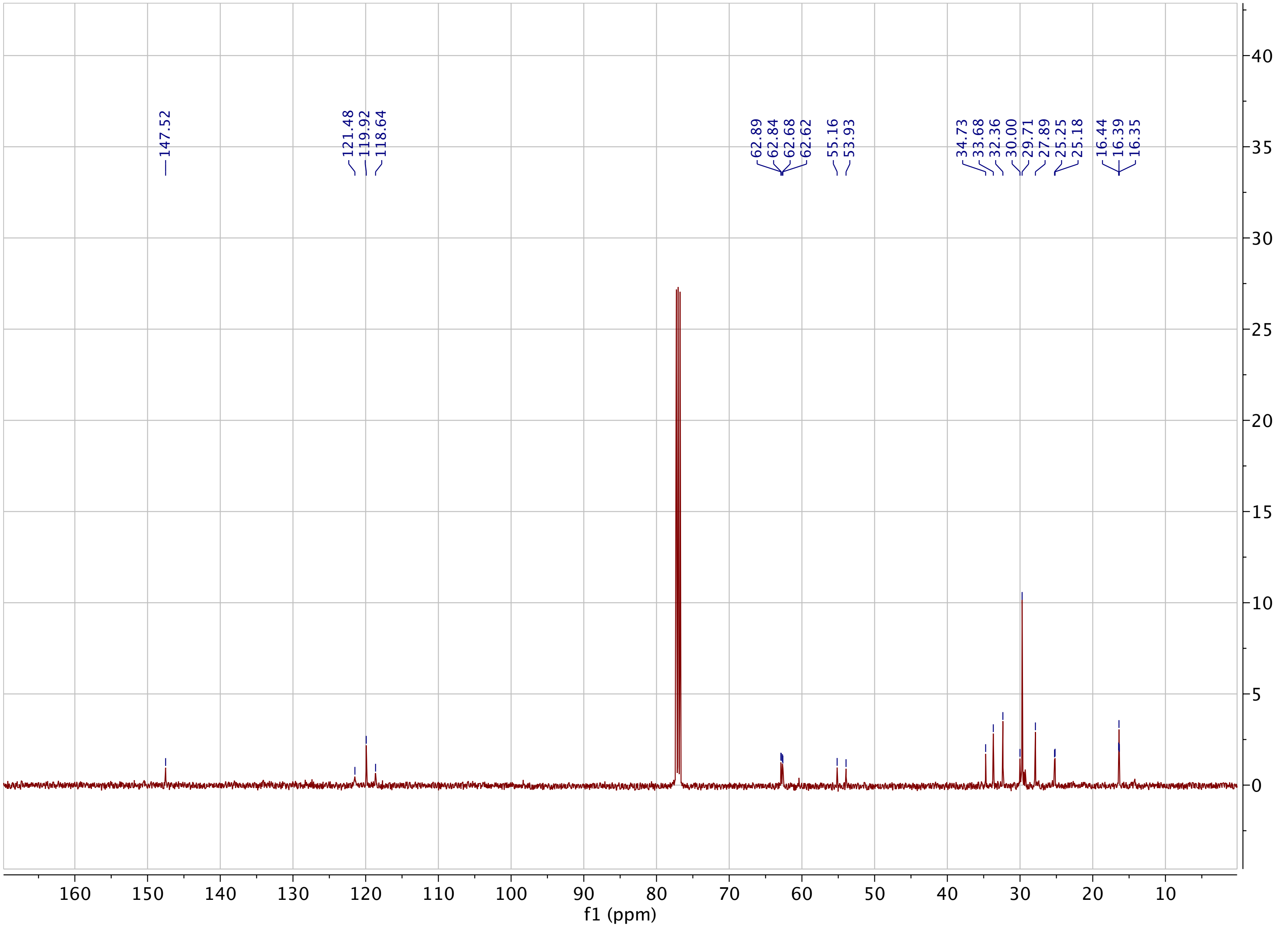
147.52
121.48
119.92
118.64
62.89
62.84
62.68
62.62
55.16
53.93
34.73
33.68
32.36
30.00
29.71
27.89
25.25
25.18
16.44
16.39
16.35
40
35
30
25
20
15
10
5
0
160
150
140
130
120
110
100
90
80
70
60
50
40
30
20
10
f1 (ppm)

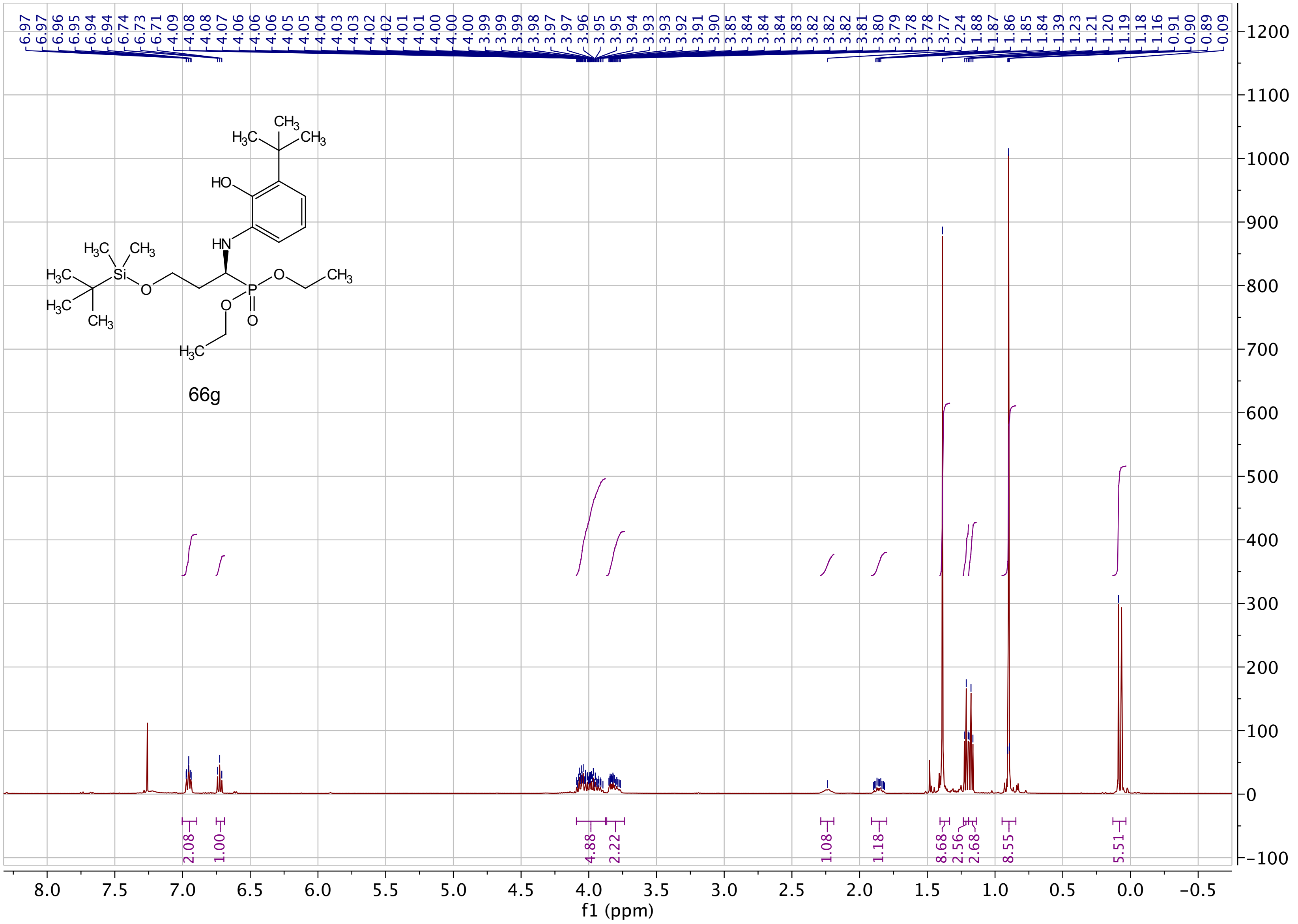

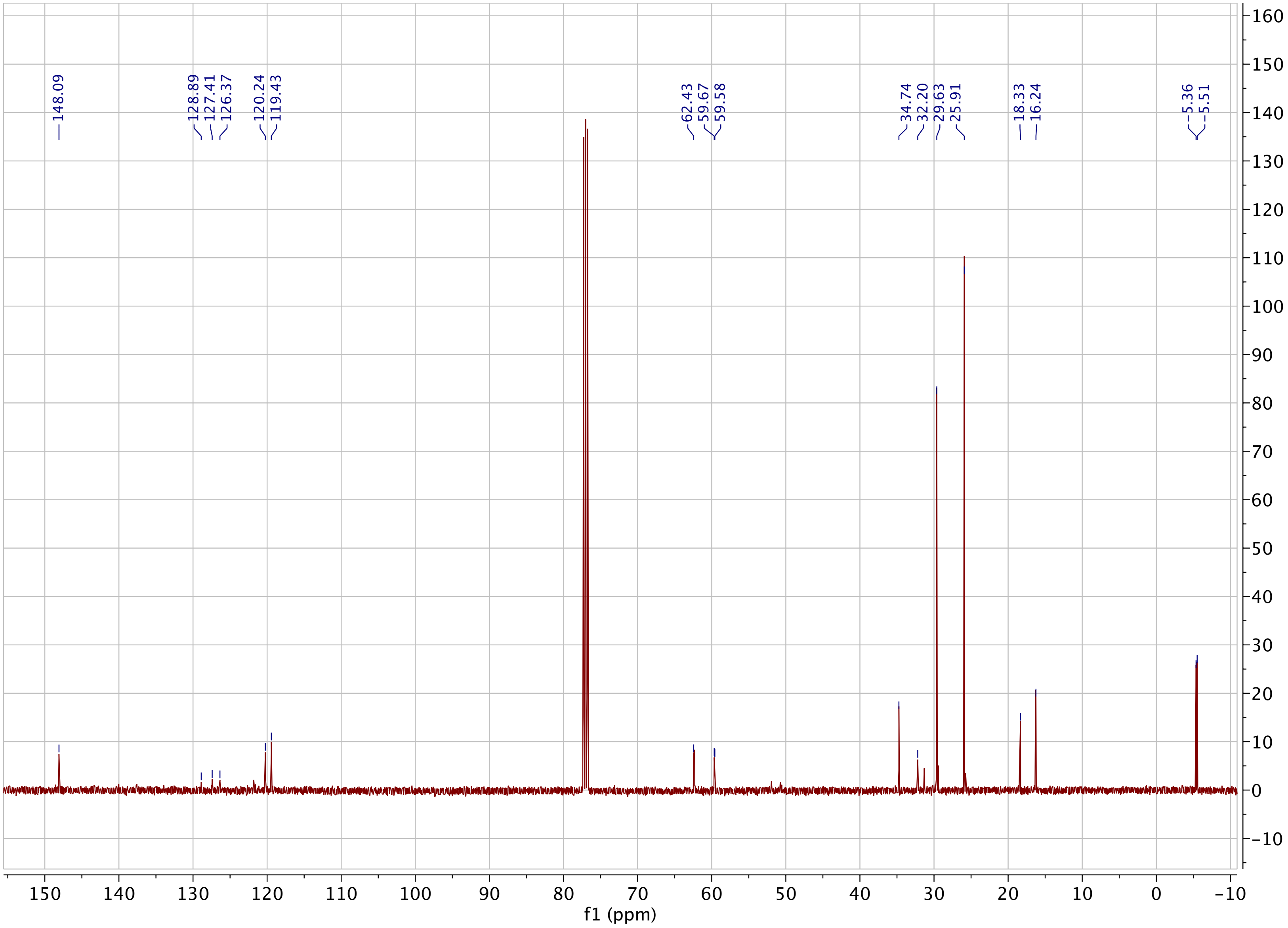
148.09
128.89
127.41
126.37
120.24
119.43
62.43
59.67
59.58
34.74
32.20
29.63
25.91
18.33
16.24
-5.36
-5.51
f1 (ppm)

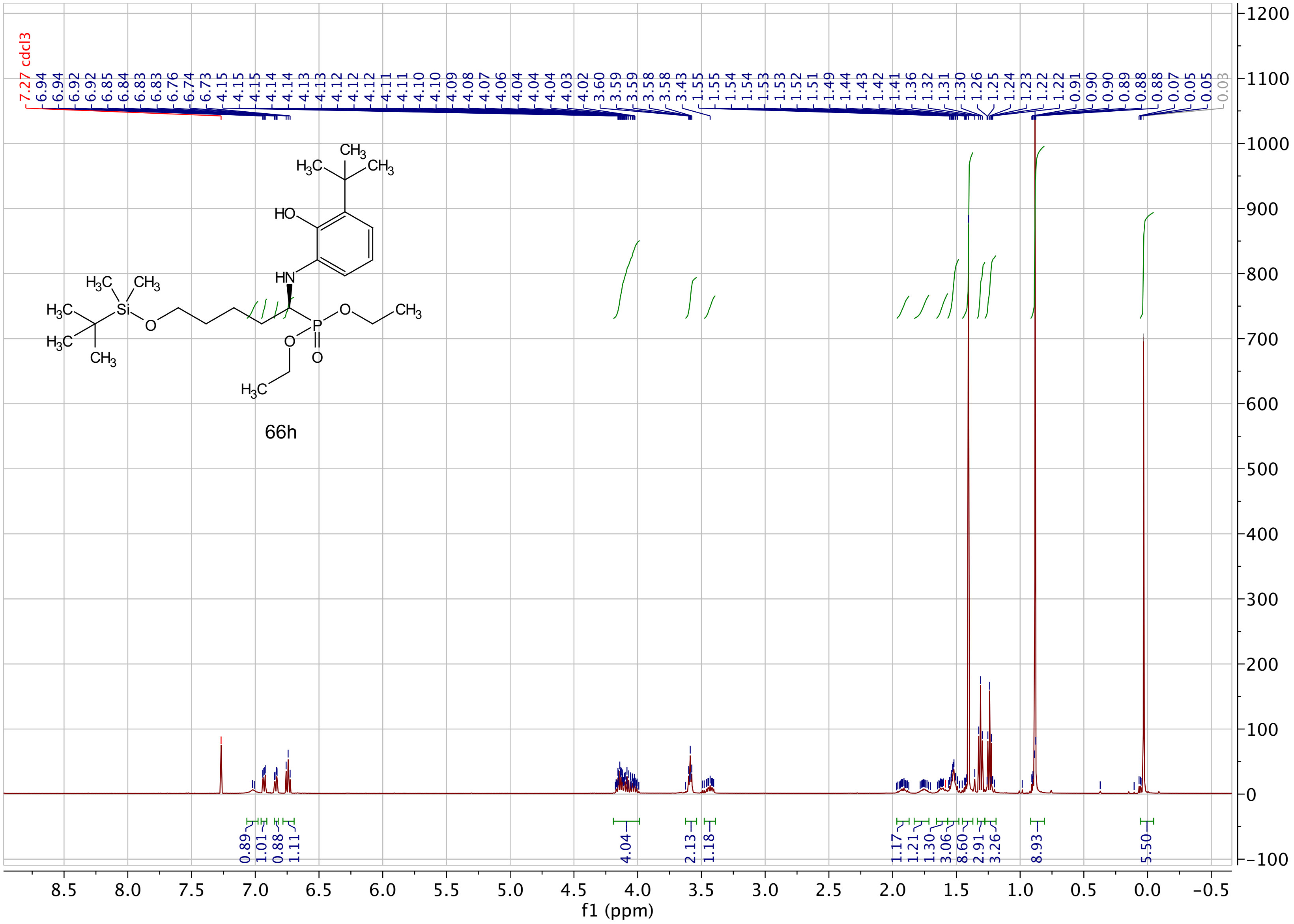

7.27 cdcl3
66h
f1 (ppm)

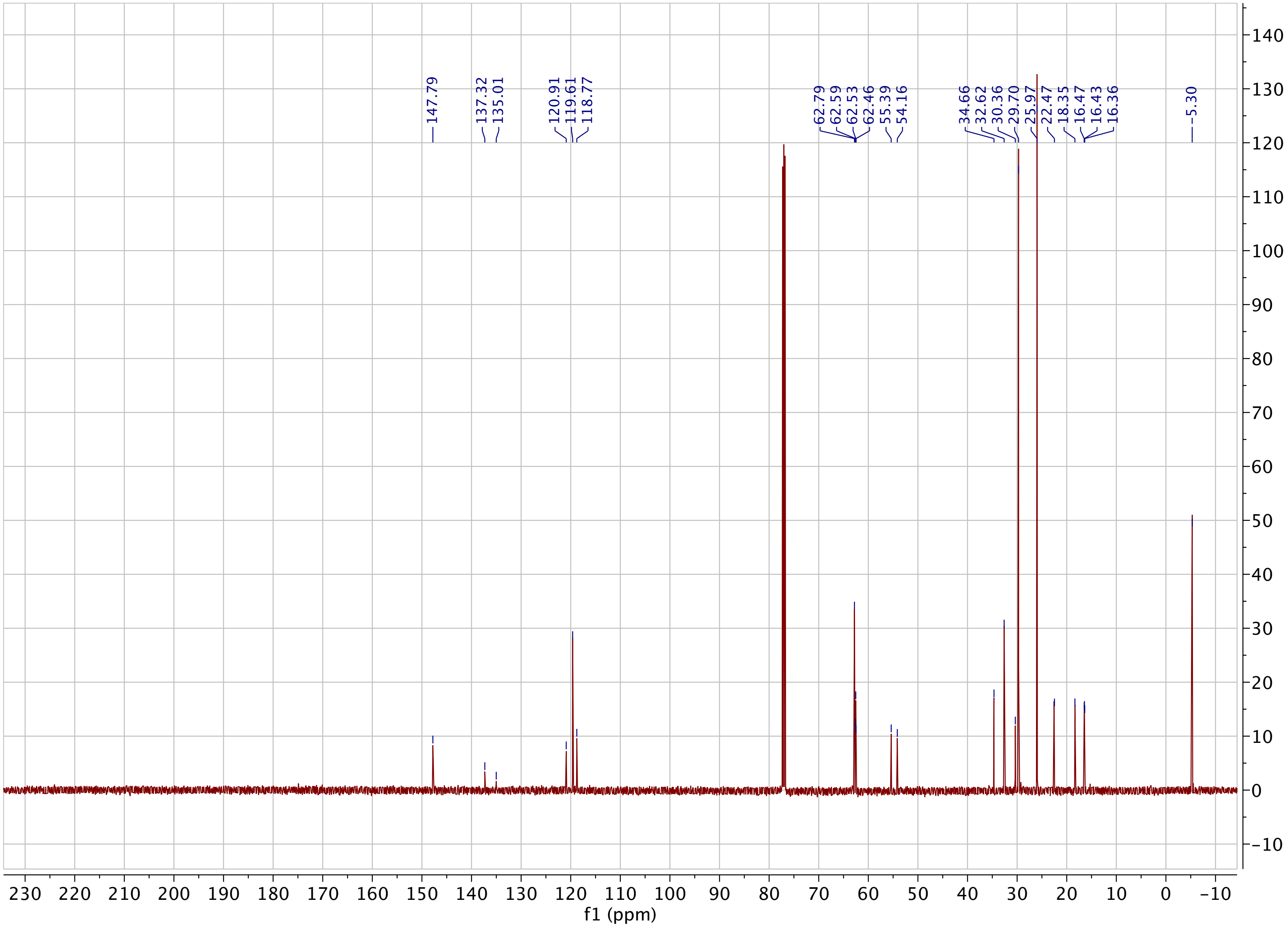

147.79
137.32
135.01
120.91
119.61
118.77
62.79
62.59
62.53
62.46
55.39
54.16
34.66
32.62
30.36
29.70
25.97
22.47
18.35
16.47
16.43
16.36
-5.30
f1 (ppm)

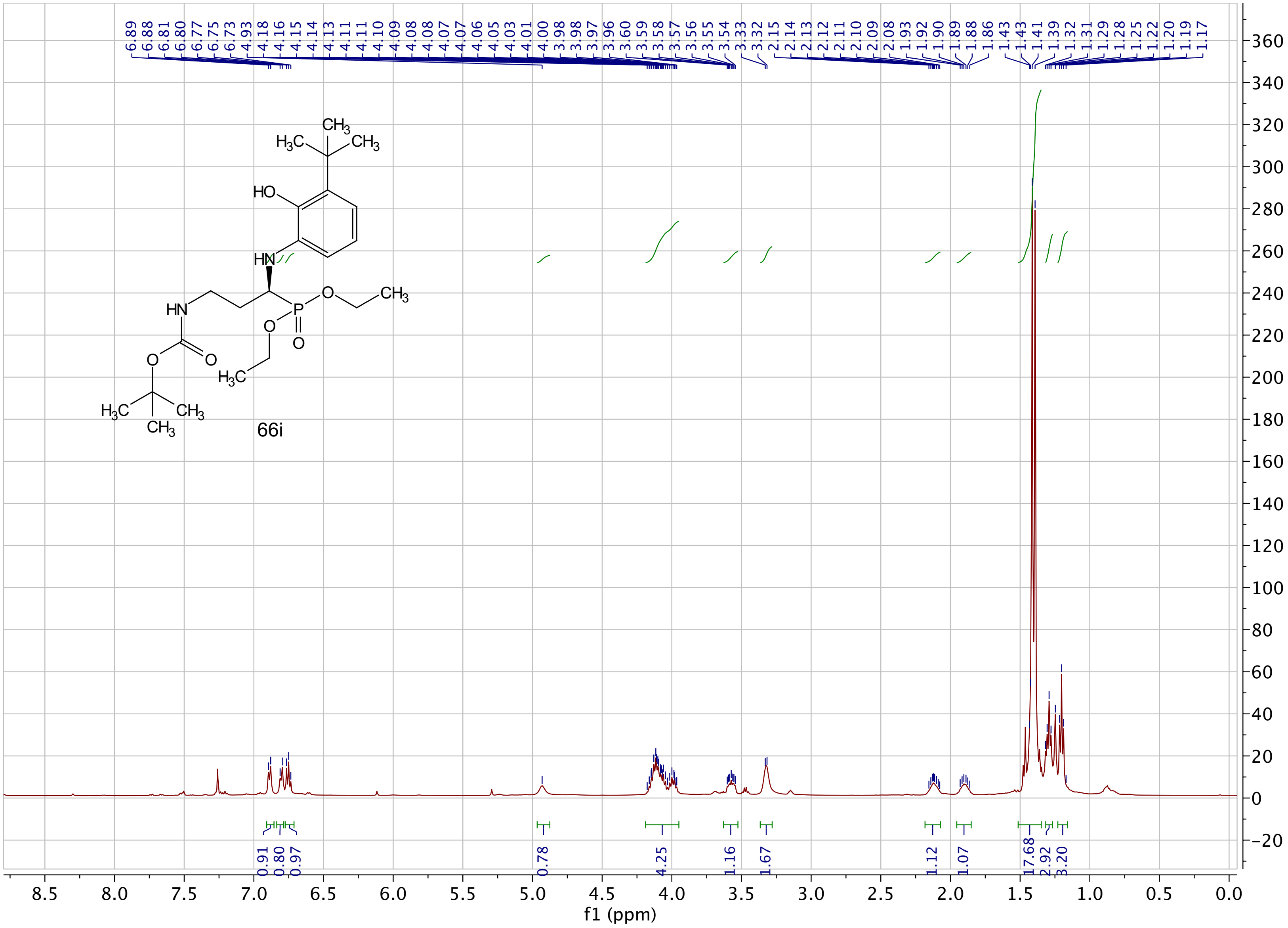

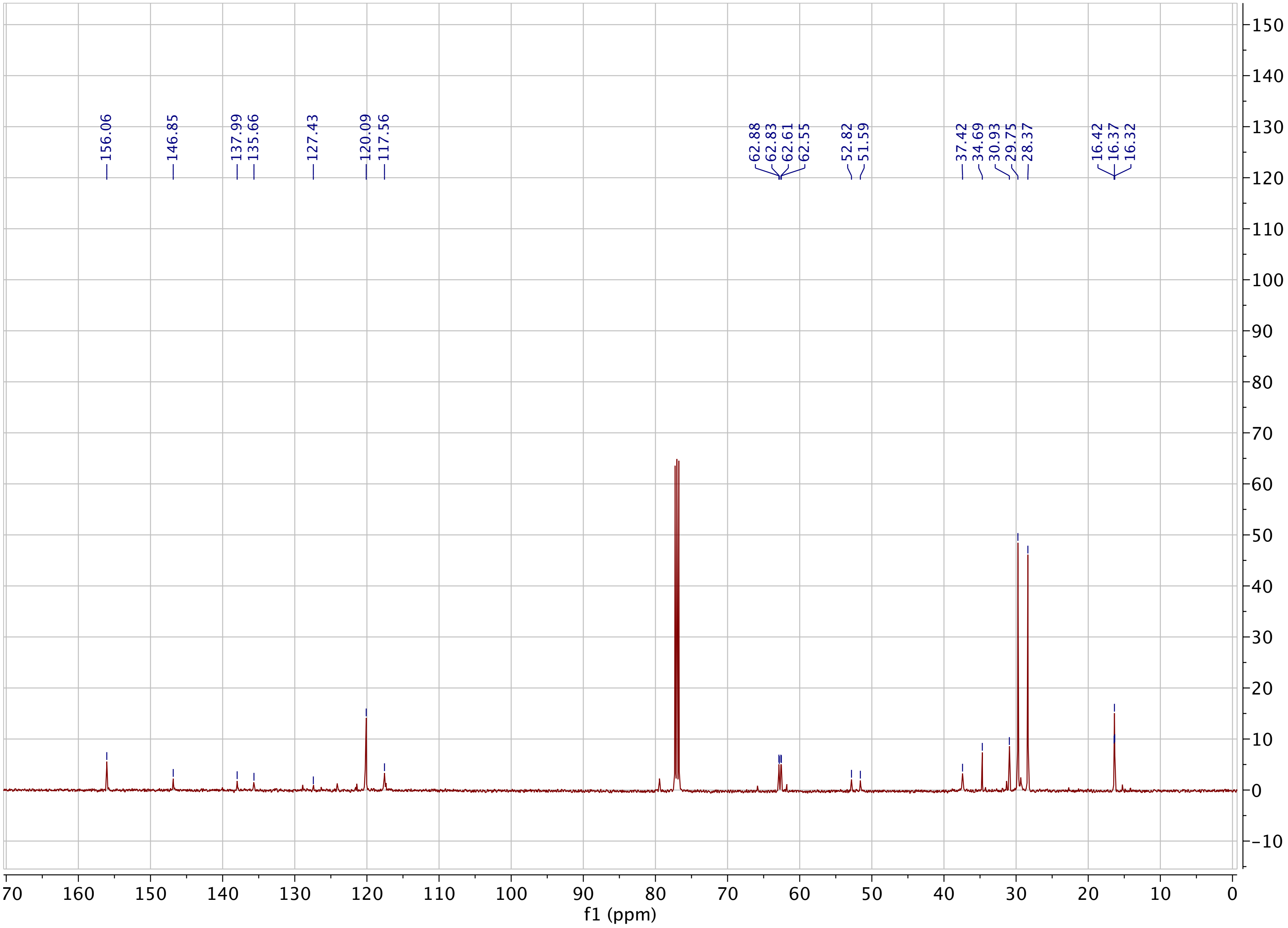
156.06
146.85
137.99
135.66
127.43
120.09
117.56
62.88
62.83
62.61
62.55
52.82
51.59
37.42
34.69
30.93
29.75
28.37
16.42
16.37
16.32
f1 (ppm)

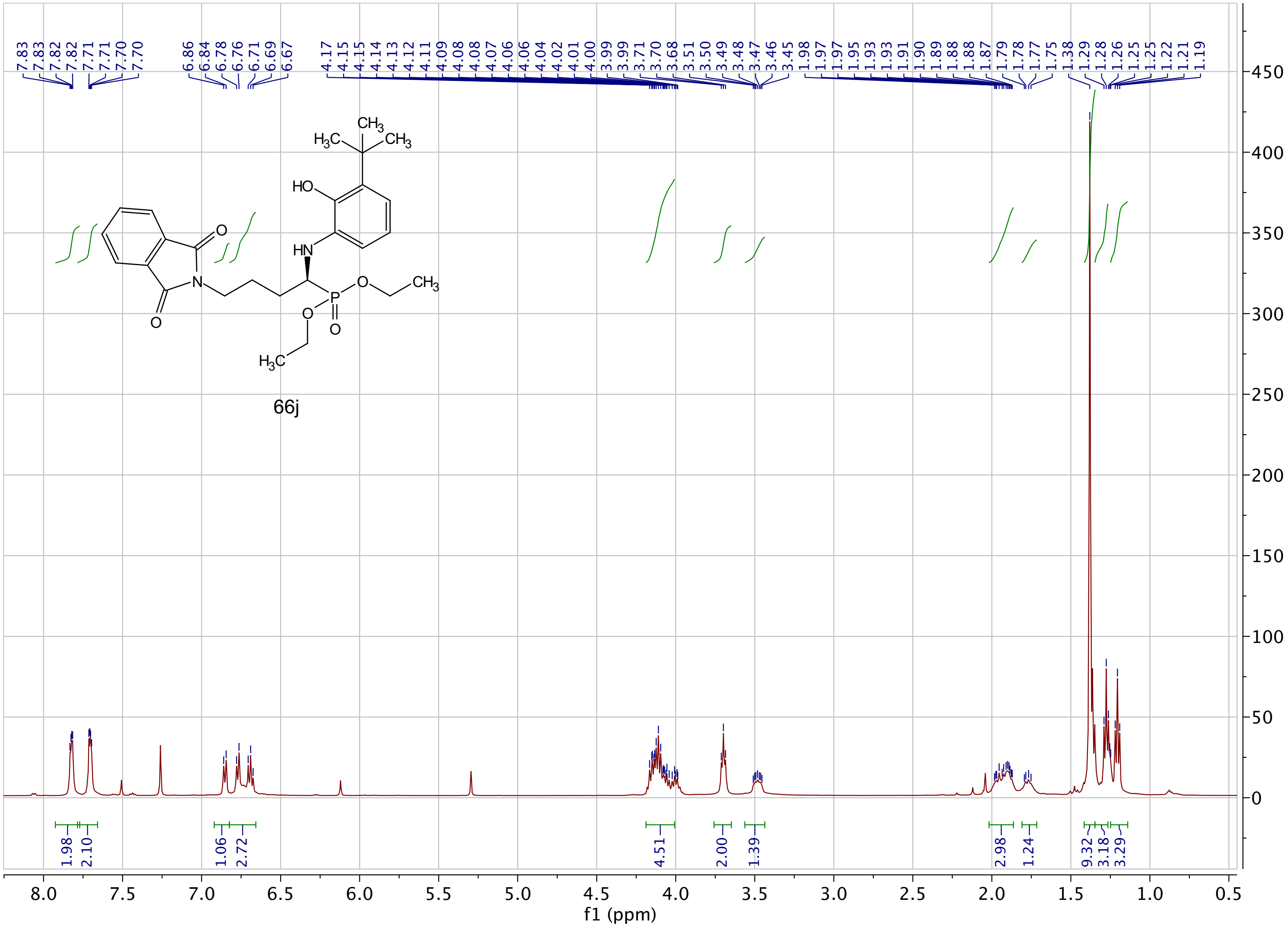

66j
f1 (ppm)

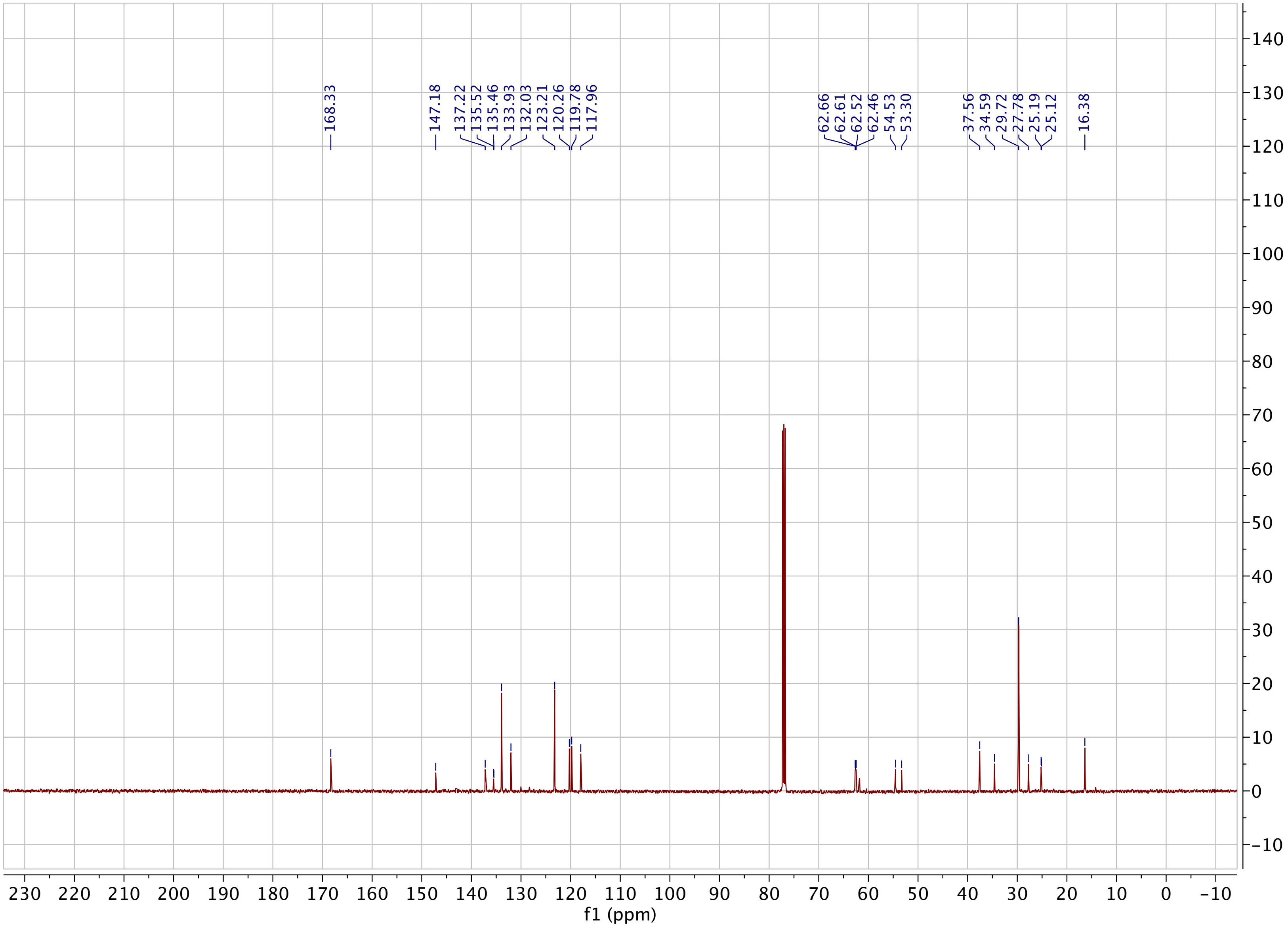
168.33
147.18
137.22
135.52
135.46
133.93
132.03
123.21
120.26
119.78
117.96
62.66
62.61
62.52
62.46
54.53
53.30
37.56
34.59
29.72
27.78
25.19
25.12
16.38
f1 (ppm)

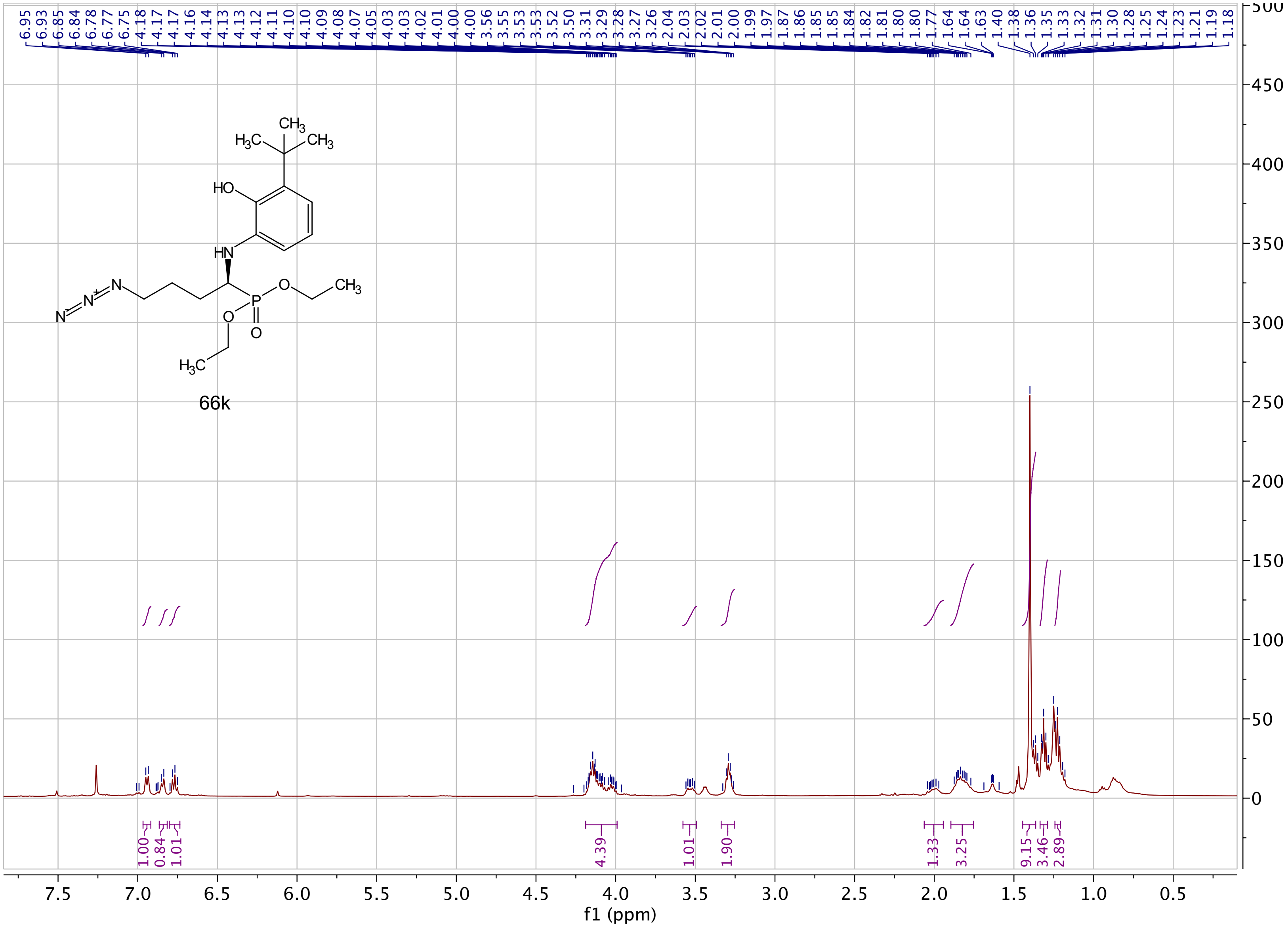
66k
f1 (ppm)

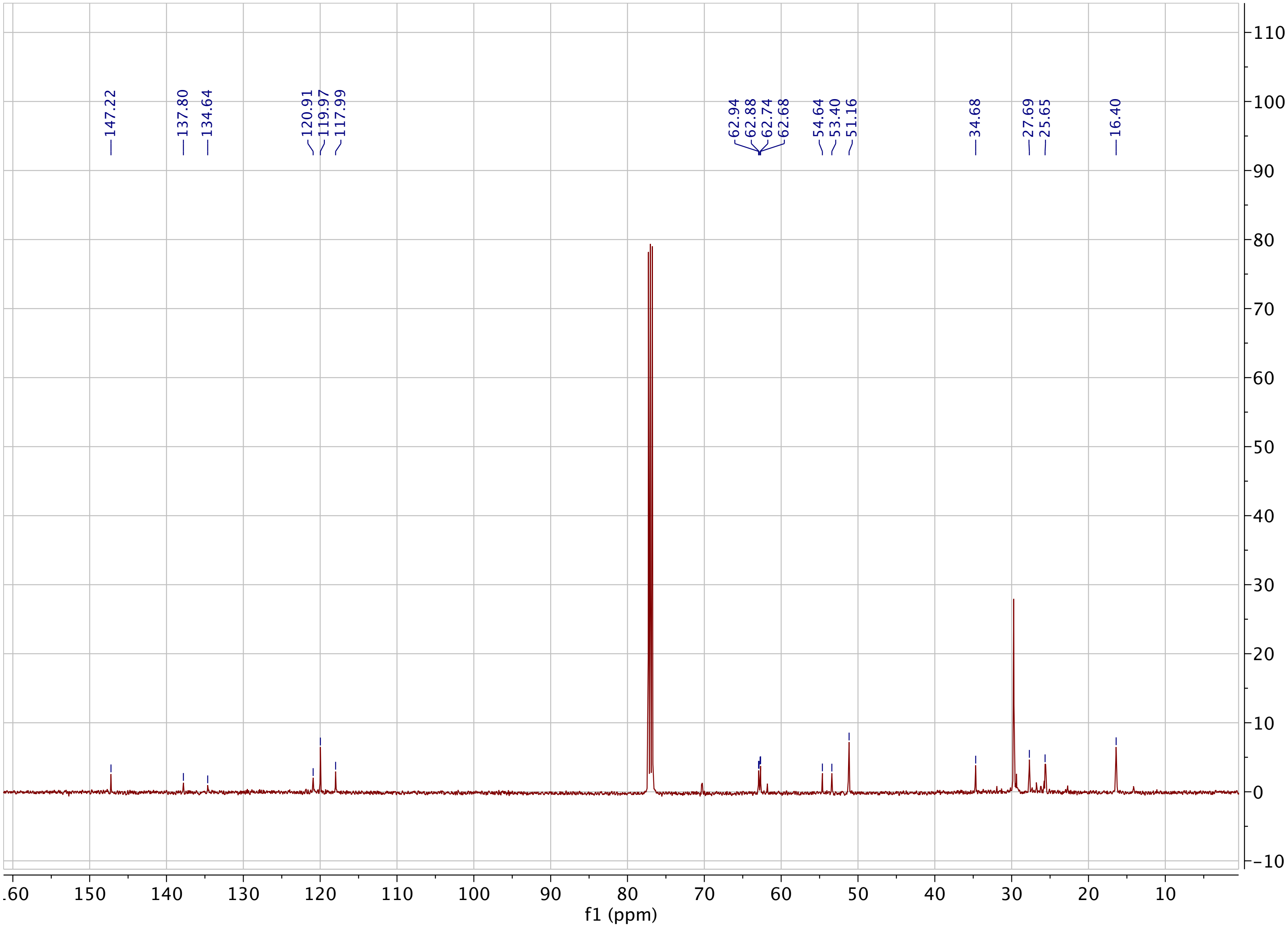

147.22
137.80
134.64
120.91
119.97
117.99
62.94
62.88
62.74
62.68
54.64
53.40
51.16
34.68
27.69
25.65
16.40
f1 (ppm)

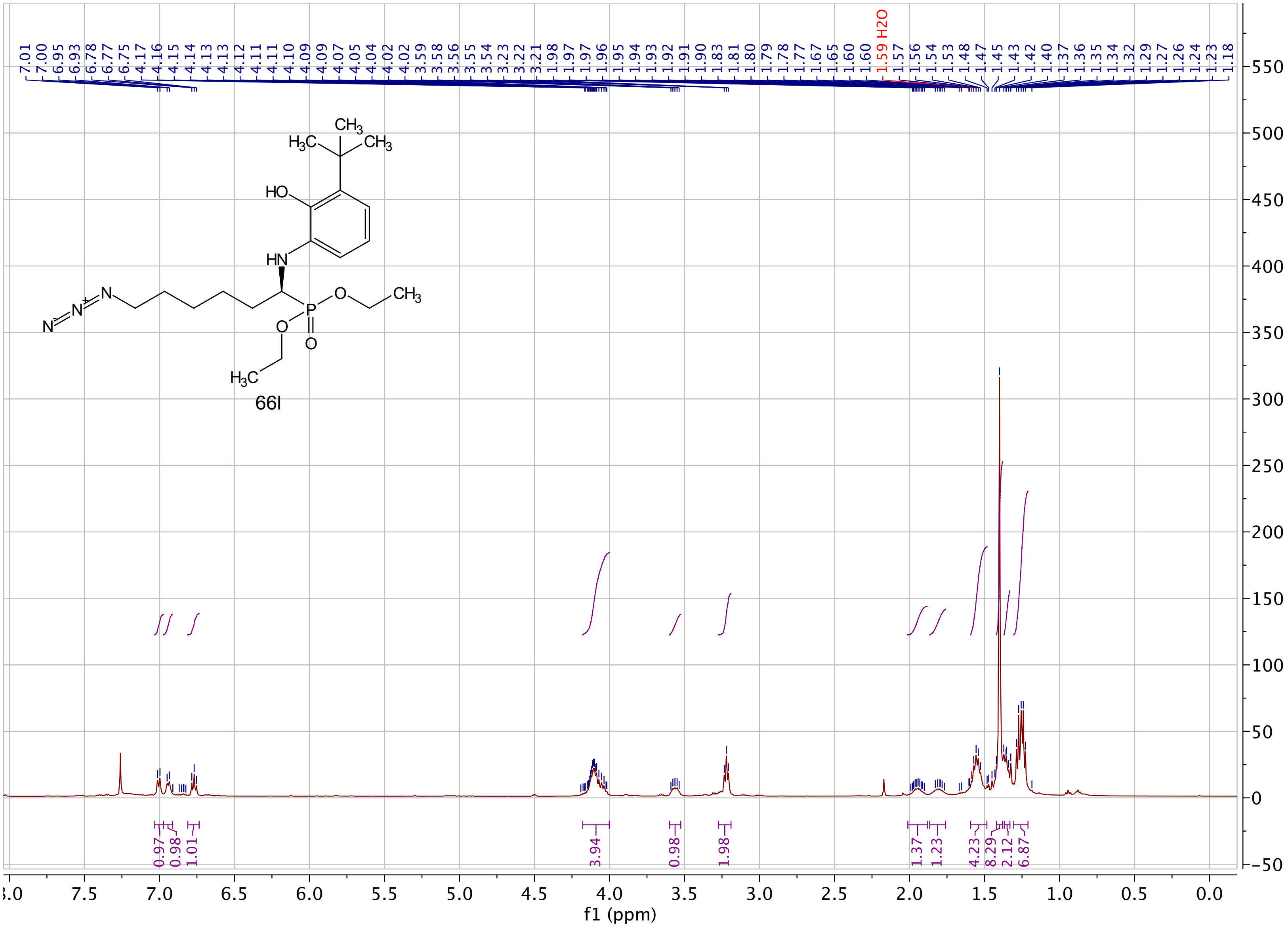
66l
f1 (ppm)
1.59 H2O

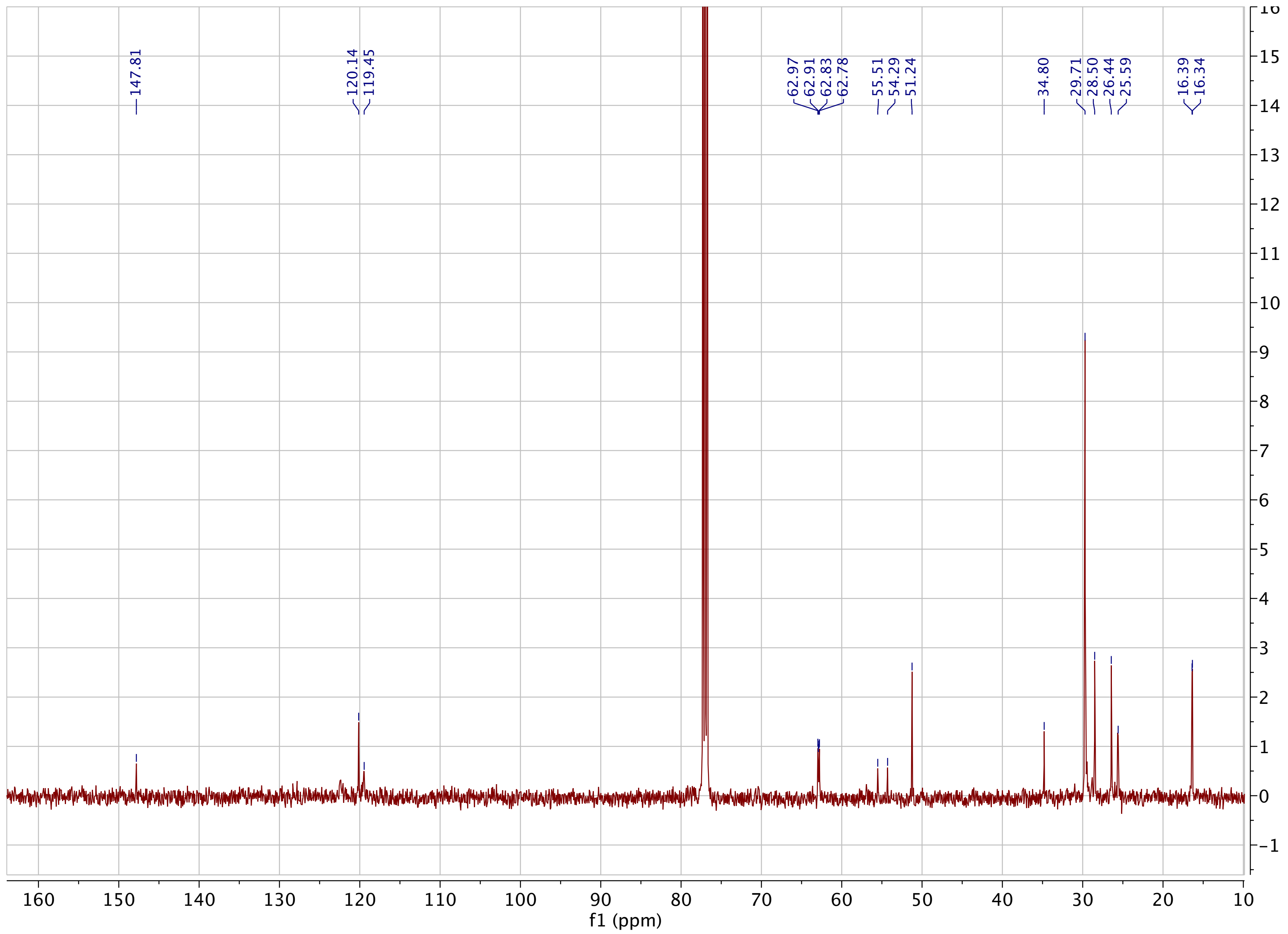

147.81
120.14
119.45
62.97
62.91
62.83
62.78
55.51
54.29
51.24
34.80
29.71
28.50
26.44
25.59
16.39
16.34
f1 (ppm)

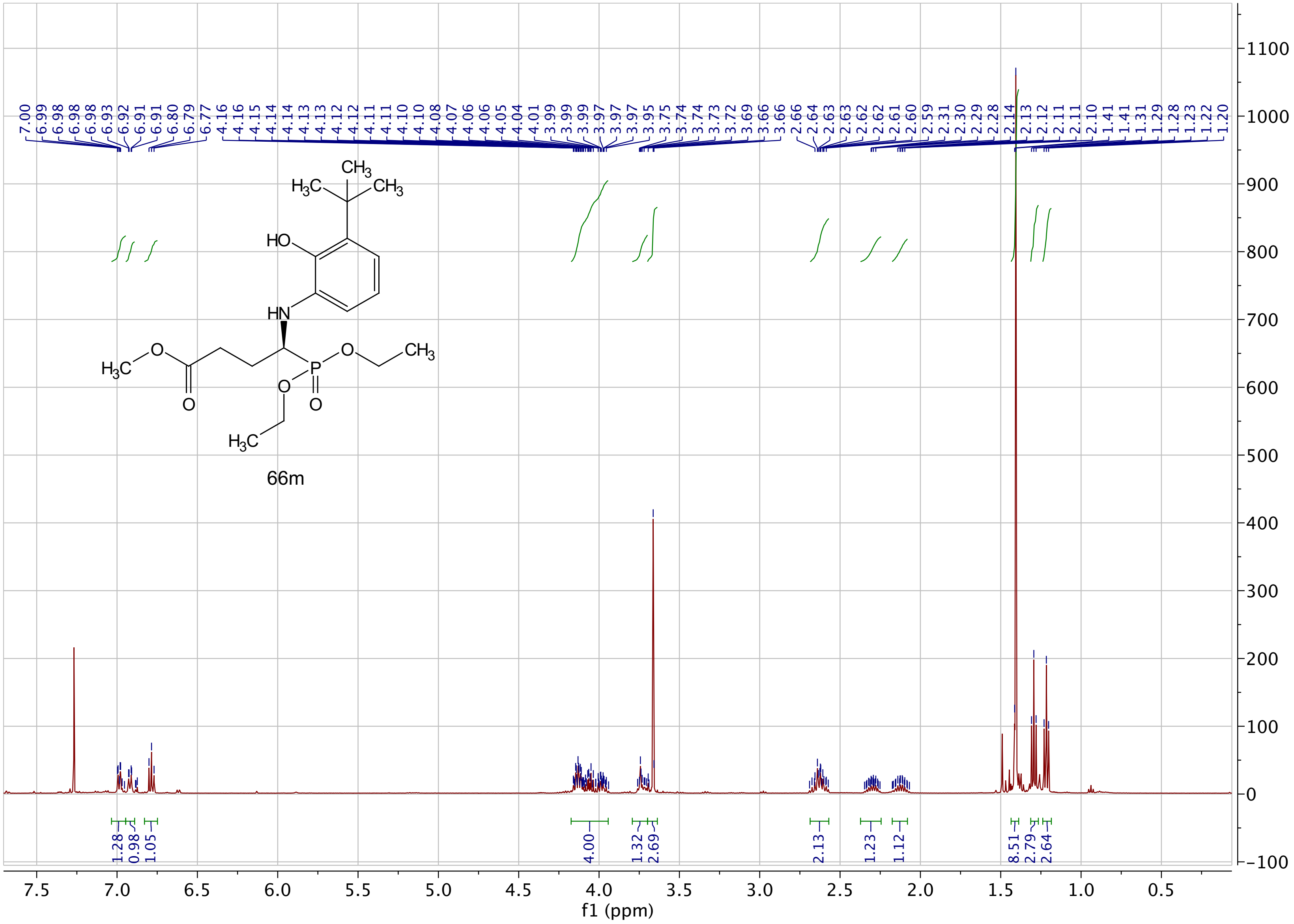

66m

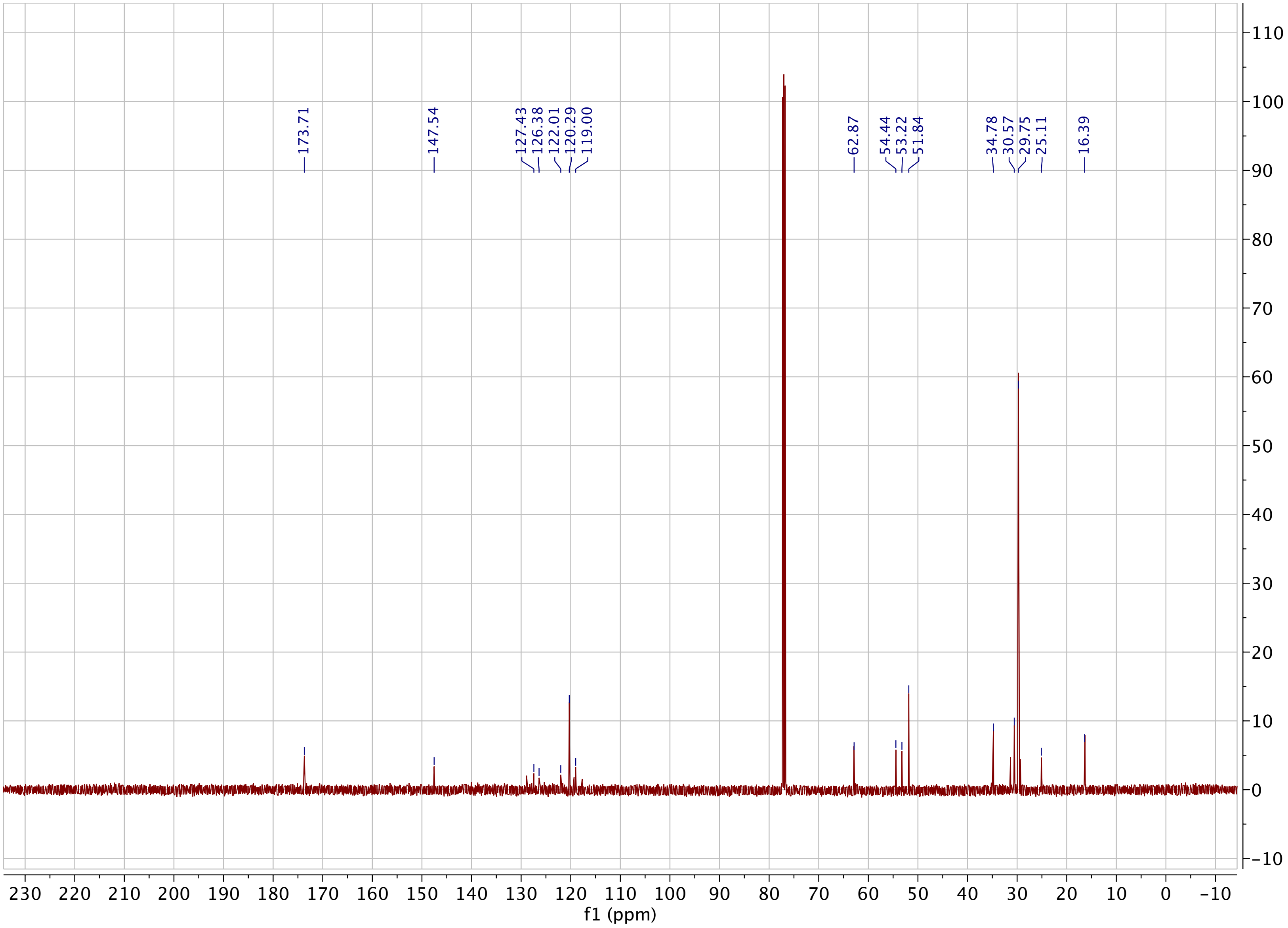

173.71
147.54
127.43
126.38
122.01
120.29
119.00
62.87
54.44
53.22
51.84
34.78
30.57
29.75
25.11
16.39
f1 (ppm)

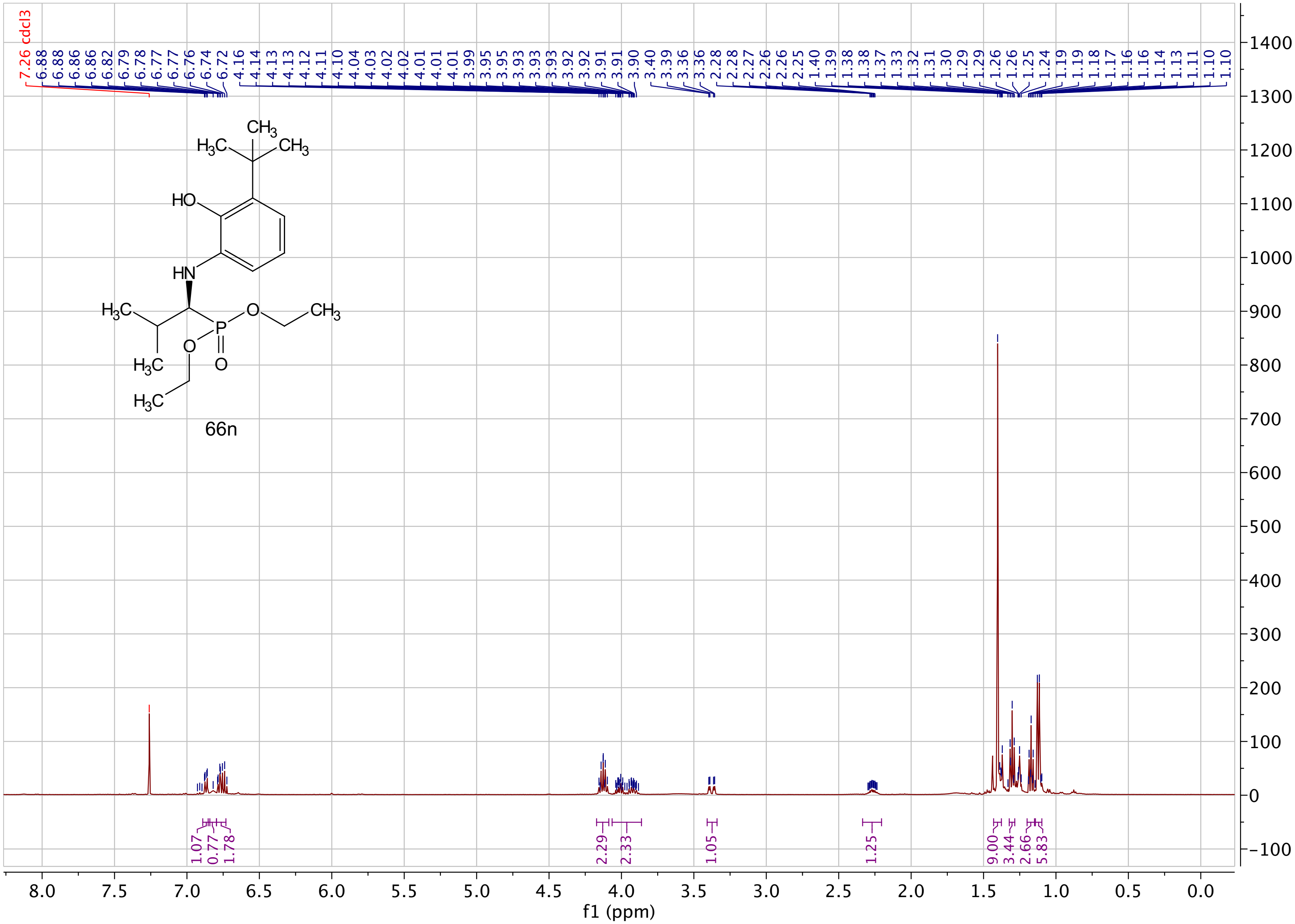

7.26 cdcl3
6.88
6.88
6.86
6.86
6.82
6.79
6.78
6.77
6.77
6.76
6.74
6.72
4.16
4.14
4.13
4.13
4.12
4.11
4.10
4.04
4.03
4.02
4.02
4.01
4.01
4.01
3.99
3.95
3.95
3.93
3.93
3.93
3.92
3.92
3.91
3.91
3.90
3.40
3.39
3.36
3.36
2.28
2.28
2.27
2.26
2.26
2.25
1.40
1.39
1.38
1.38
1.37
1.33
1.32
1.31
1.30
1.29
1.29
1.26
1.26
1.25
1.24
1.19
1.19
1.18
1.17
1.16
1.16
1.14
1.13
1.11
1.10
1.10
66n
1.07
0.77
1.78
2.29
2.33
1.05
1.25
9.00
3.44
2.66
5.83
f1 (ppm)

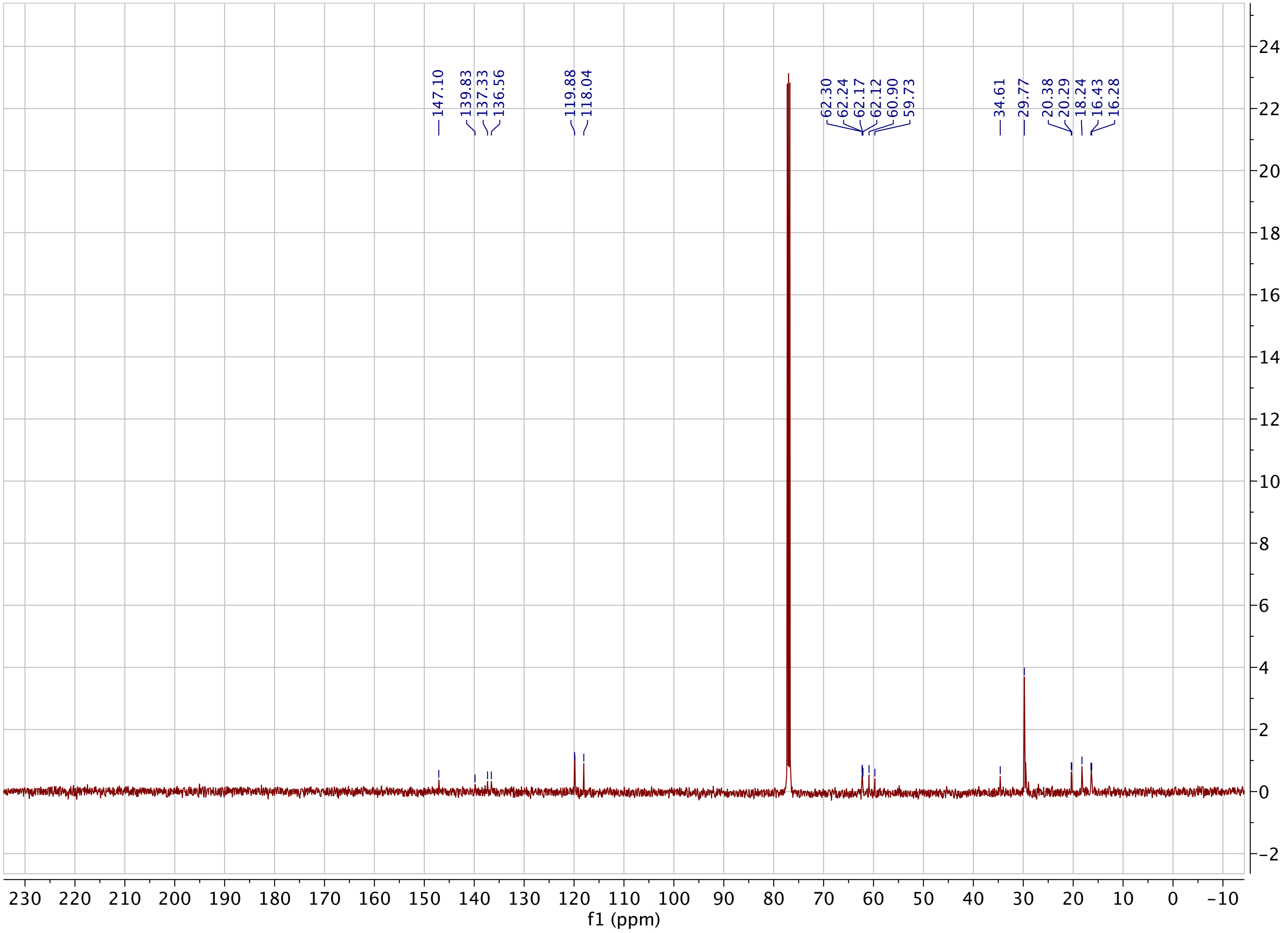
147.10
139.83
137.33
136.56
119.88
118.04
62.30
62.24
62.17
62.12
60.90
59.73
34.61
29.77
20.38
20.29
18.24
16.43
16.28
f1 (ppm)

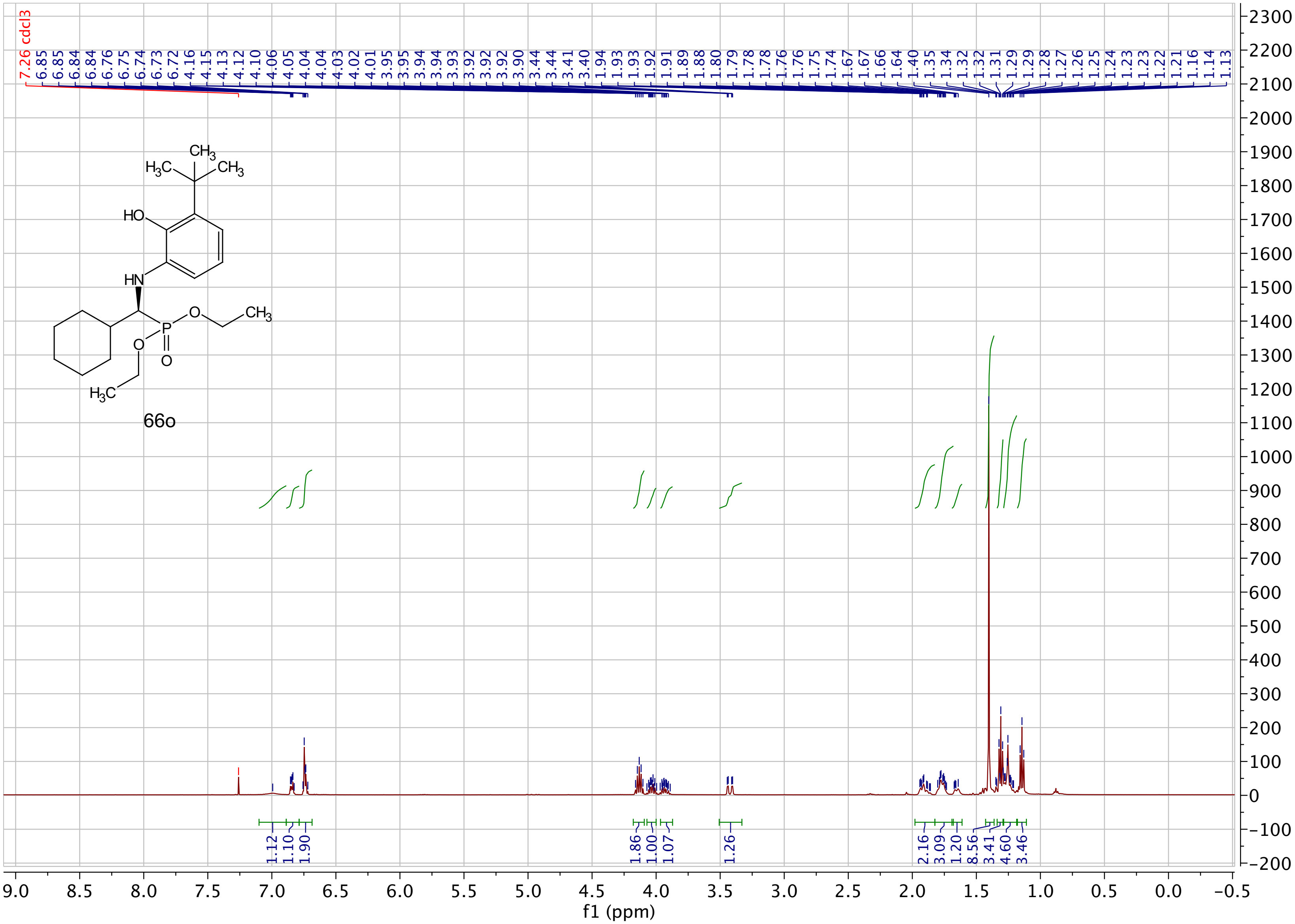

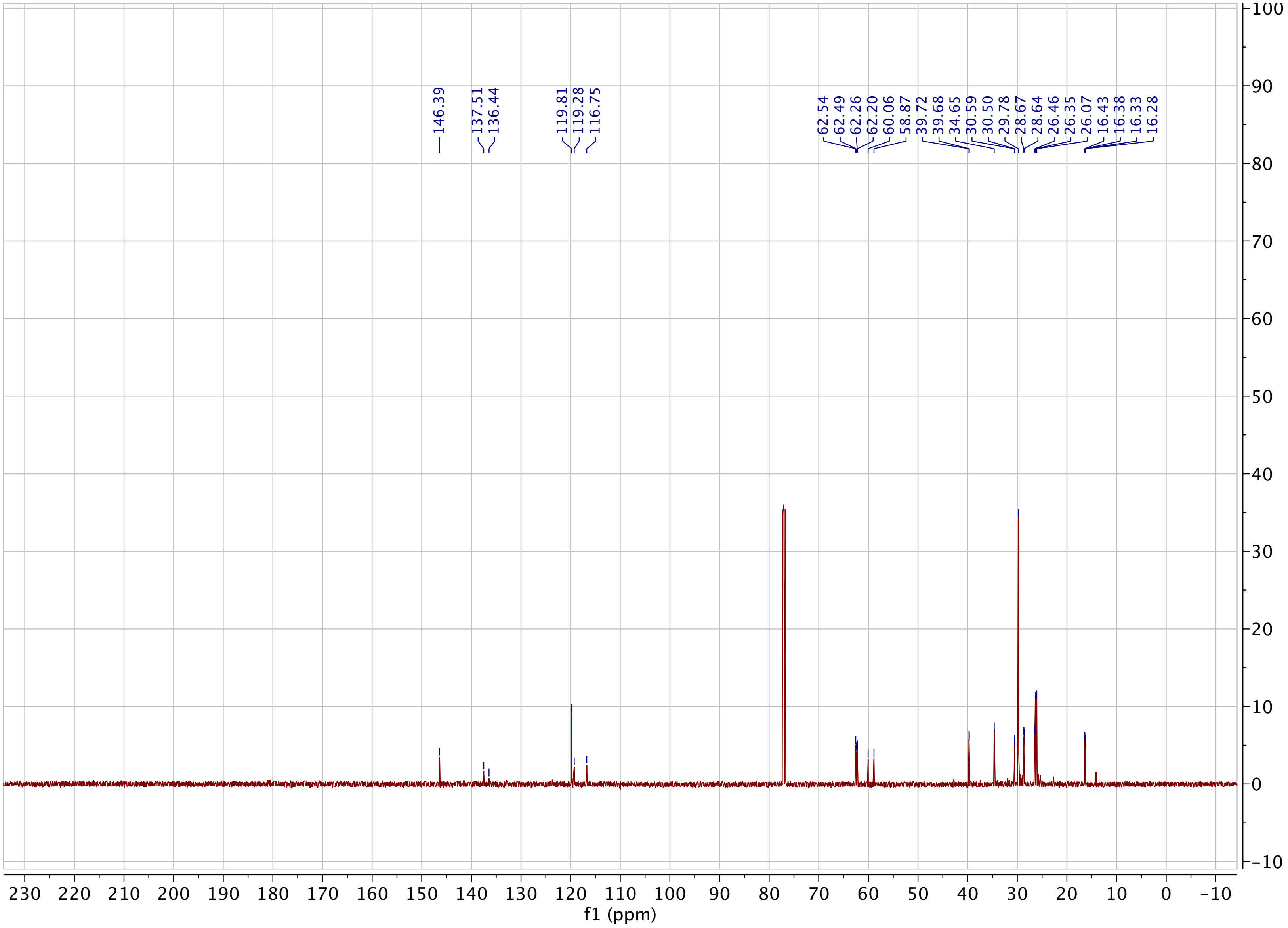

146.39
137.51
136.44
119.81
119.28
116.75
62.54
62.49
62.26
62.20
60.06
58.87
39.72
39.68
34.65
30.59
30.50
29.78
28.67
28.64
26.46
26.35
26.07
16.43
16.38
16.33
16.28
f1 (ppm)

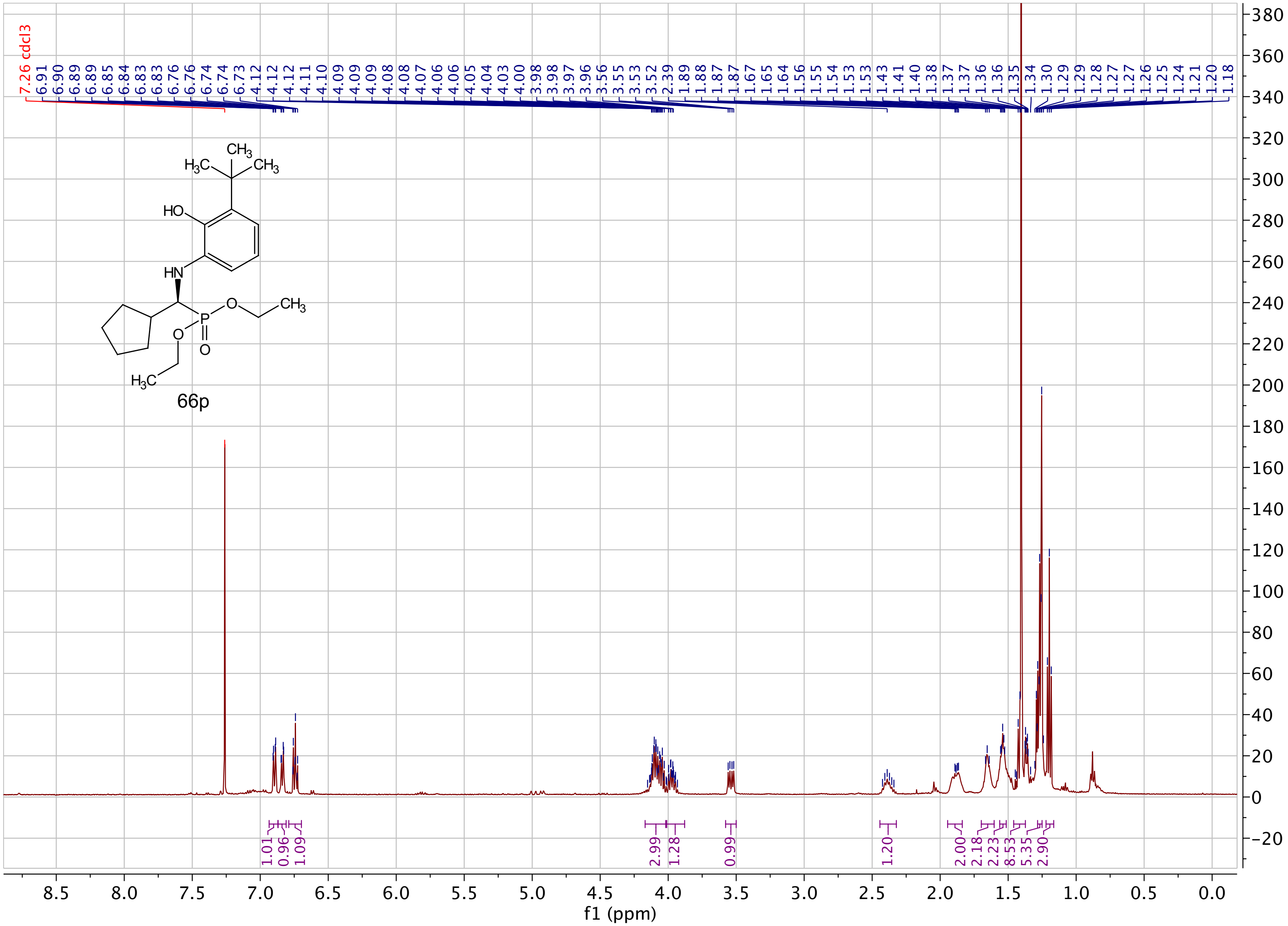

7.26 cdcl3
6.91
6.90
6.89
6.89
6.85
6.84
6.83
6.83
6.76
6.74
6.74
6.73
4.12
4.12
4.11
4.10
4.09
4.09
4.09
4.08
4.08
4.07
4.06
4.06
4.05
4.04
4.03
4.00
3.98
3.98
3.97
3.96
3.56
3.55
3.53
3.52
2.39
1.89
1.88
1.87
1.87
1.67
1.65
1.64
1.56
1.55
1.54
1.53
1.53
1.43
1.41
1.40
1.38
1.37
1.37
1.36
1.35
1.34
1.30
1.29
1.29
1.28
1.27
1.27
1.26
1.25
1.24
1.21
1.20
1.18
CH3
H3C
CH3
HO
HN
O
CH3
P
O
O
H3C
66p
1.01
0.96
1.09
2.99
1.28
0.99
1.20
2.00
2.18
2.23
8.53
5.35
2.90
f1 (ppm)

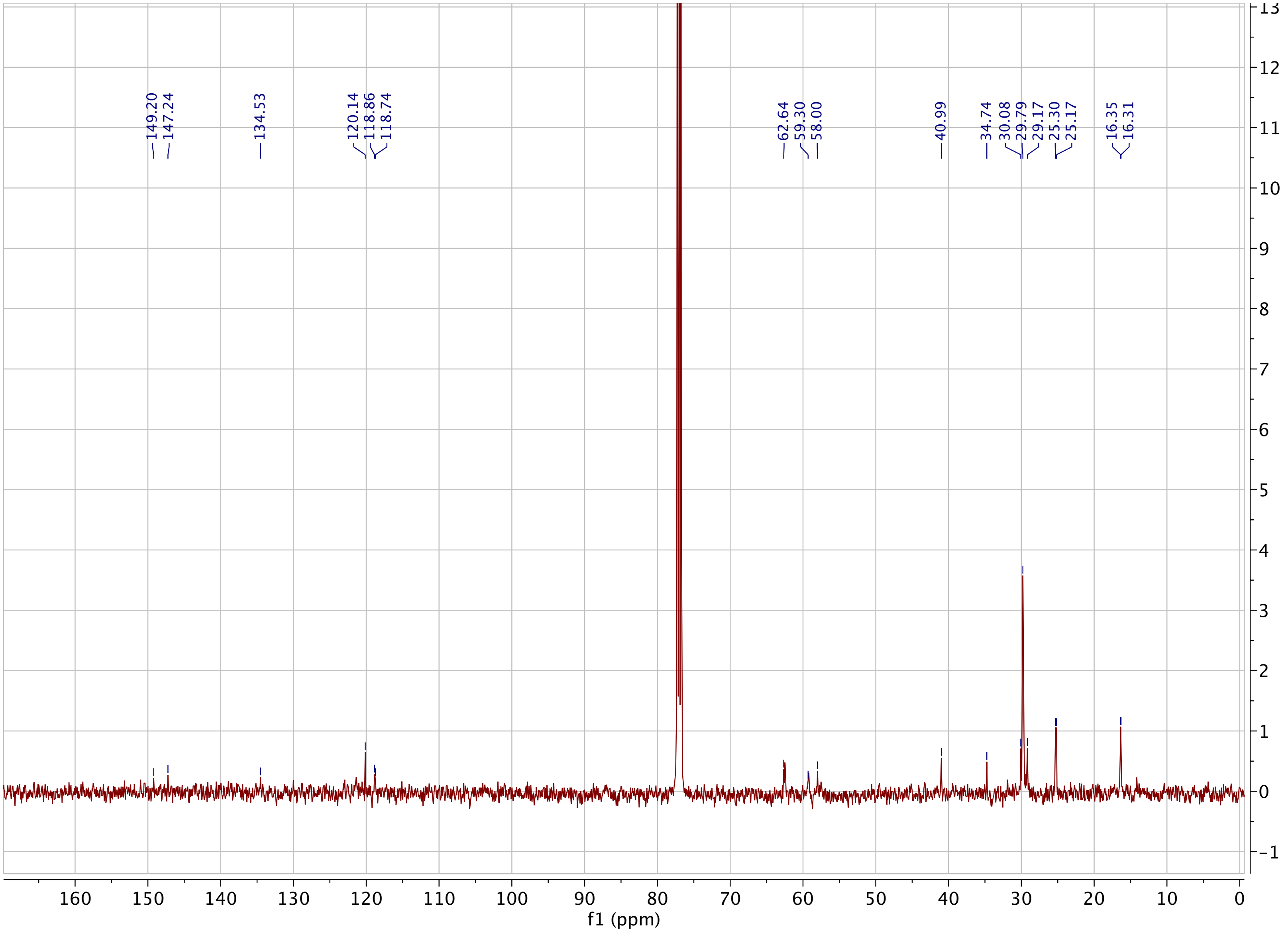
149.20
147.24
134.53
120.14
118.86
118.74
62.64
59.30
58.00
40.99
34.74
30.08
29.79
29.17
25.30
25.17
16.35
16.31
f1 (ppm)

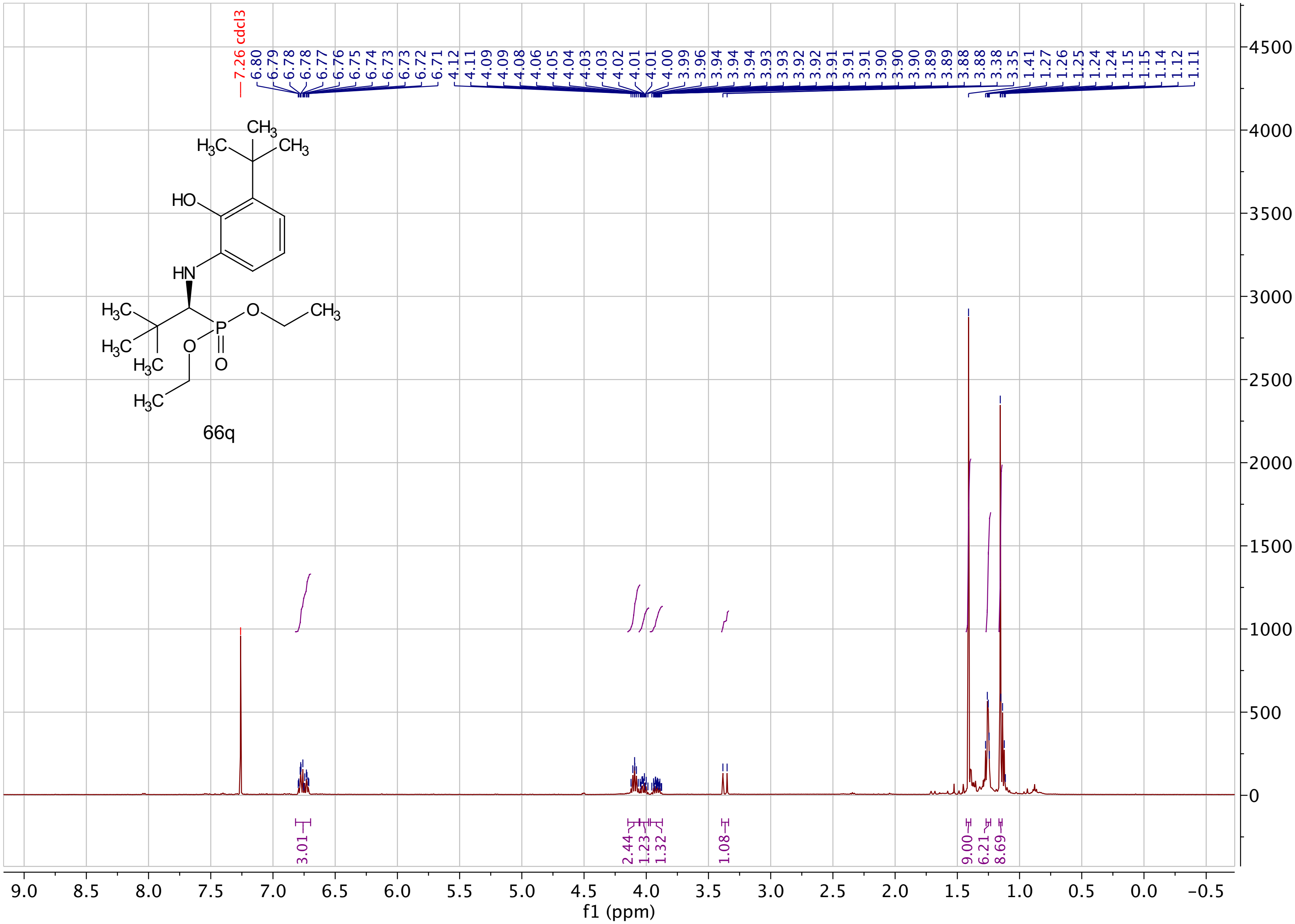
7.26 cdcl3
6.80 6.79 6.78 6.78 6.77 6.76 6.75 6.74 6.73 6.73 6.72 6.71 4.12 4.11 4.09 4.09 4.08 4.06 4.05 4.04 4.03 4.03 4.02 4.01 4.01 4.00 3.99 3.96 3.94 3.94 3.94 3.93 3.93 3.92 3.92 3.91 3.91 3.91 3.90 3.90 3.90 3.89 3.89 3.88 3.88 3.38 3.35 1.41 1.27 1.26 1.25 1.24 1.24 1.15 1.15 1.14 1.12 1.11
CH3
H3C
CH3
HO
HN
H3C
O
CH3
P
H3C
O
H3C
O
H3C
66q
3.01
2.44
1.23
1.32
1.08
9.00
6.21
8.69
f1 (ppm)

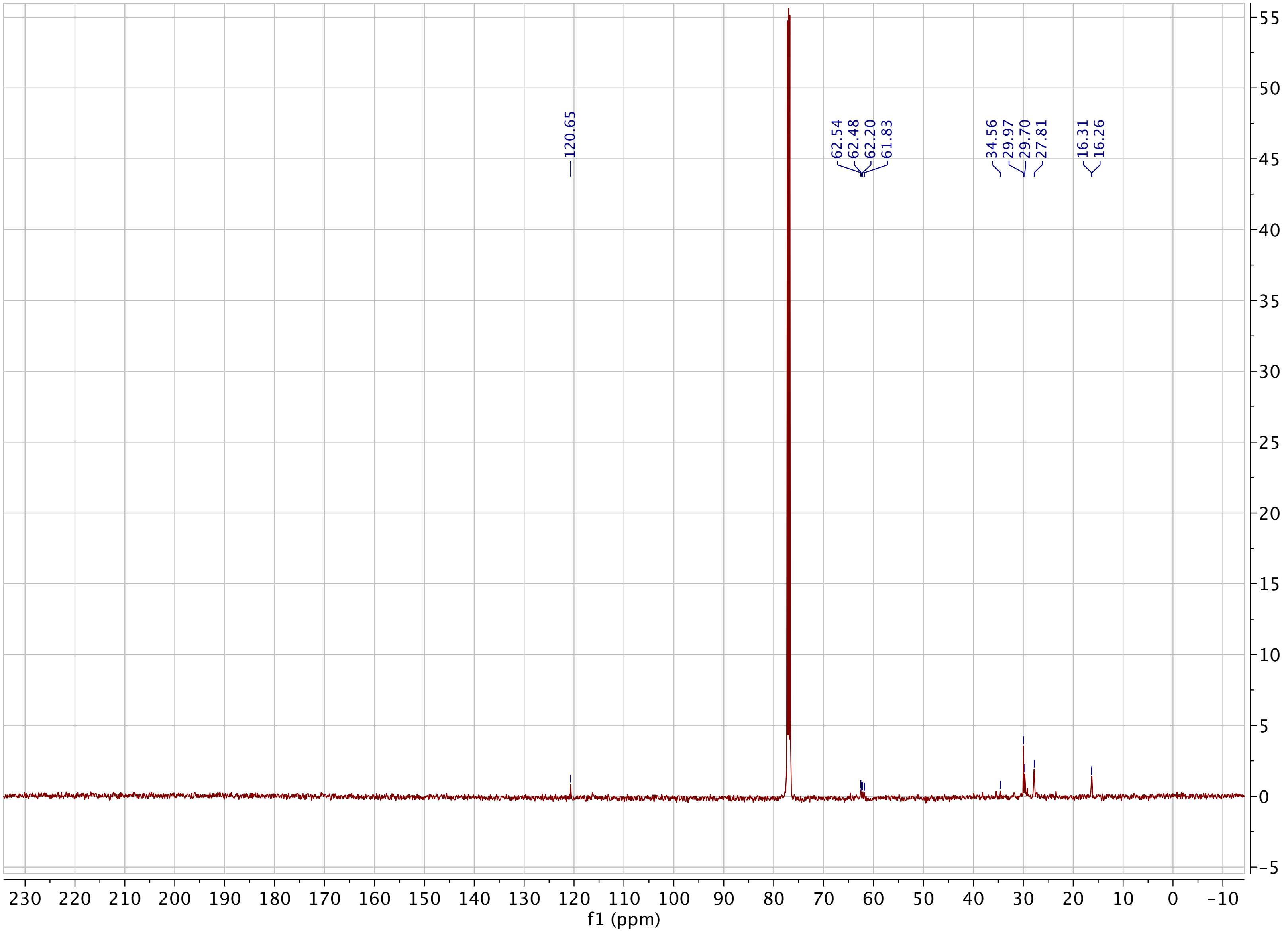

120.65
62.54
62.48
62.20
61.83
34.56
29.97
29.70
27.81
16.31
16.26
230 220 210 200 190 180 170 160 150 140 130 120 110 100 90 80 70 60 50 40 30 20 10 0 -10
f1 (ppm)
55 50 45 40 35 30 25 20 15 10 5 0 -5

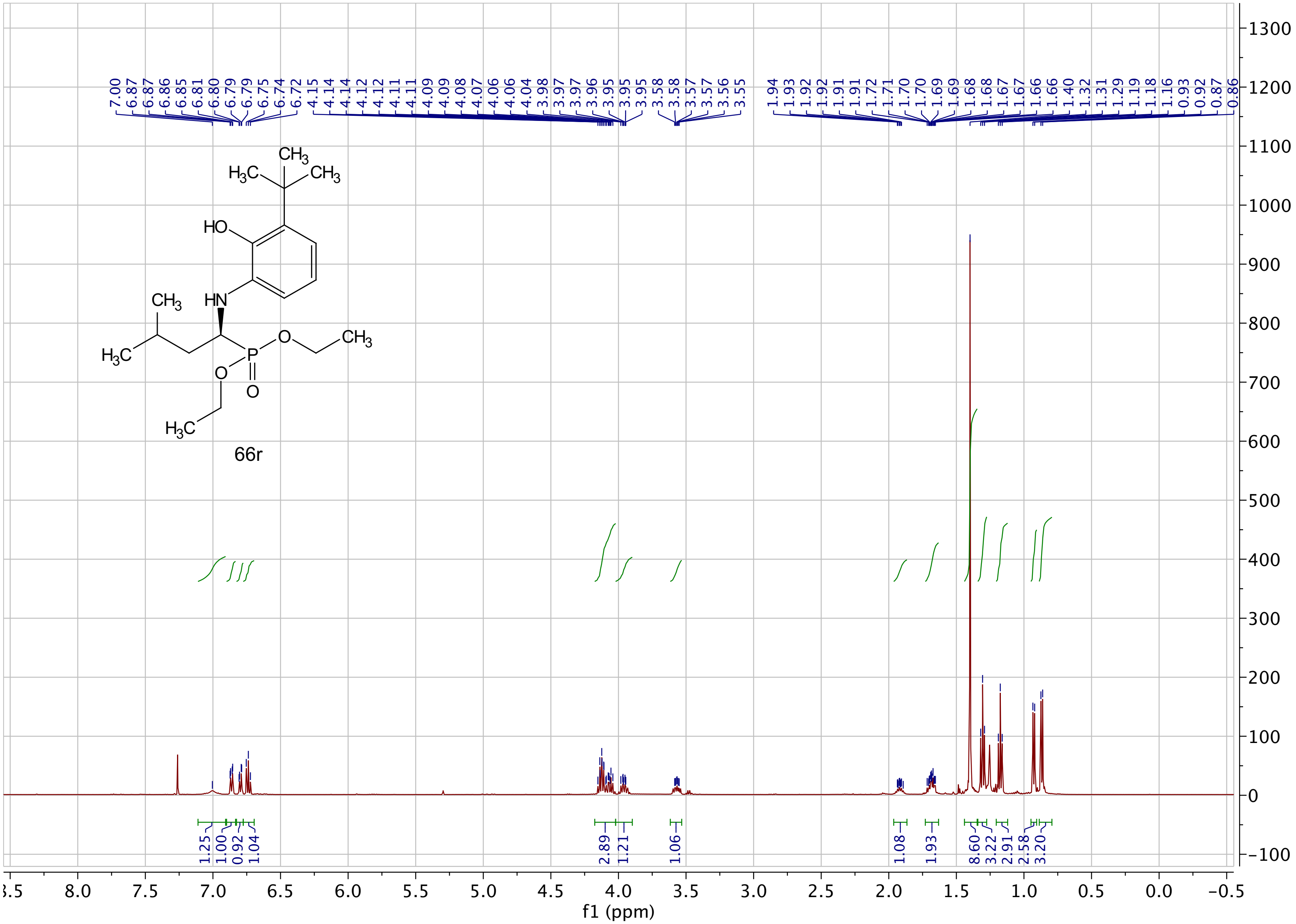

7.00
6.87
6.87
6.86
6.85
6.81
6.80
6.79
6.79
6.75
6.74
6.72
4.15
4.14
4.14
4.12
4.12
4.11
4.11
4.09
4.09
4.08
4.07
4.06
4.06
4.04
3.98
3.97
3.97
3.96
3.95
3.95
3.95
3.58
3.58
3.57
3.57
3.56
3.55
1.94
1.93
1.92
1.92
1.91
1.91
1.72
1.71
1.70
1.70
1.69
1.69
1.68
1.68
1.67
1.67
1.66
1.66
1.40
1.32
1.31
1.29
1.19
1.18
1.16
0.93
0.92
0.87
0.86
CH3
H3C
CH3
HO
CH3
HN
O
CH3
H3C
P
O
O
H3C
1.25
1.00
0.92
1.04
2.89
1.21
1.06
1.08
1.93
8.60
3.22
2.91
2.58
3.20
f1 (ppm)

66r

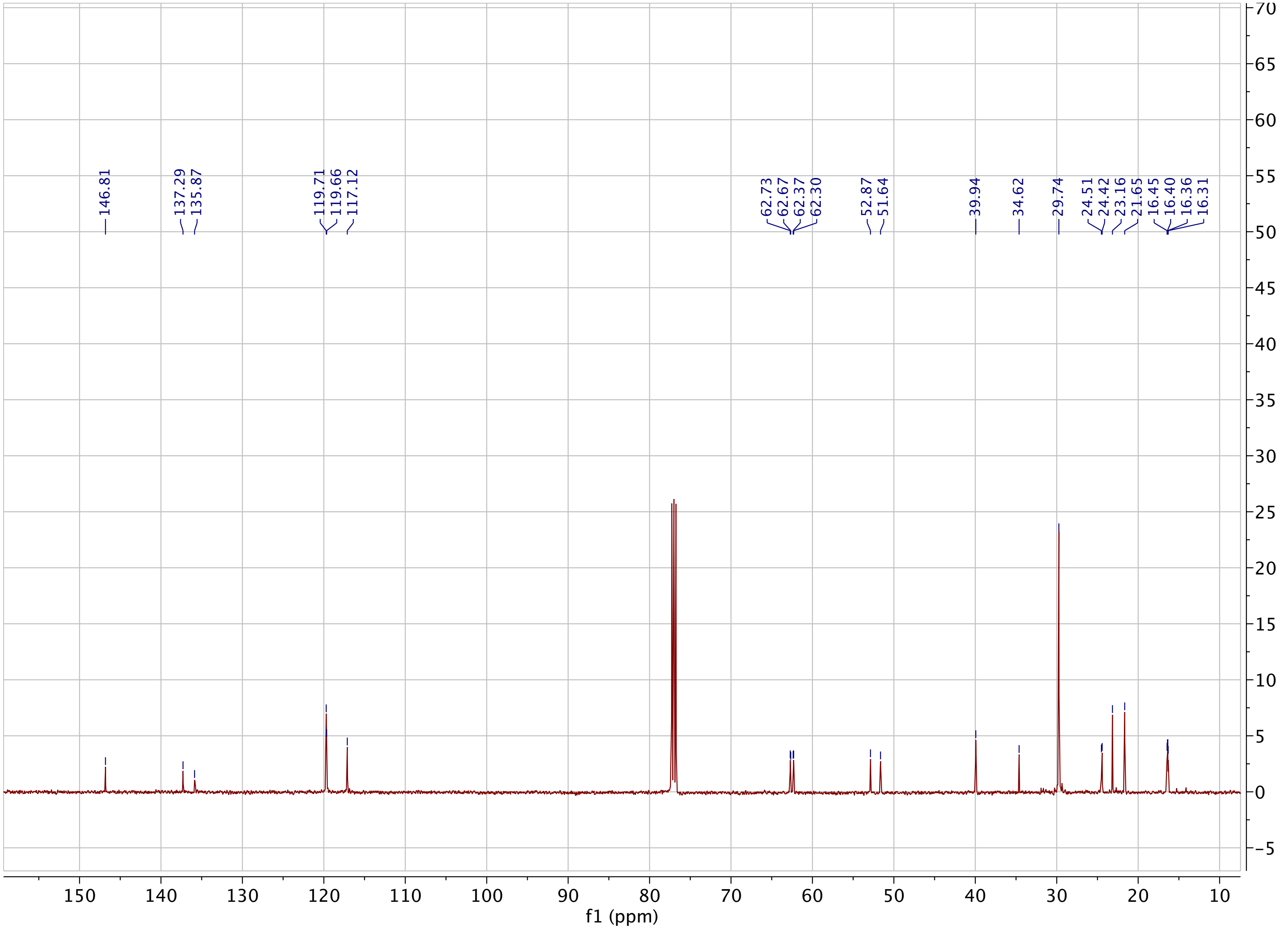
146.81
137.29
135.87
119.71
119.66
117.12
62.73
62.67
62.37
62.30
52.87
51.64
39.94
34.62
29.74
24.51
24.42
23.16
21.65
16.45
16.40
16.36
16.31
f1 (ppm)

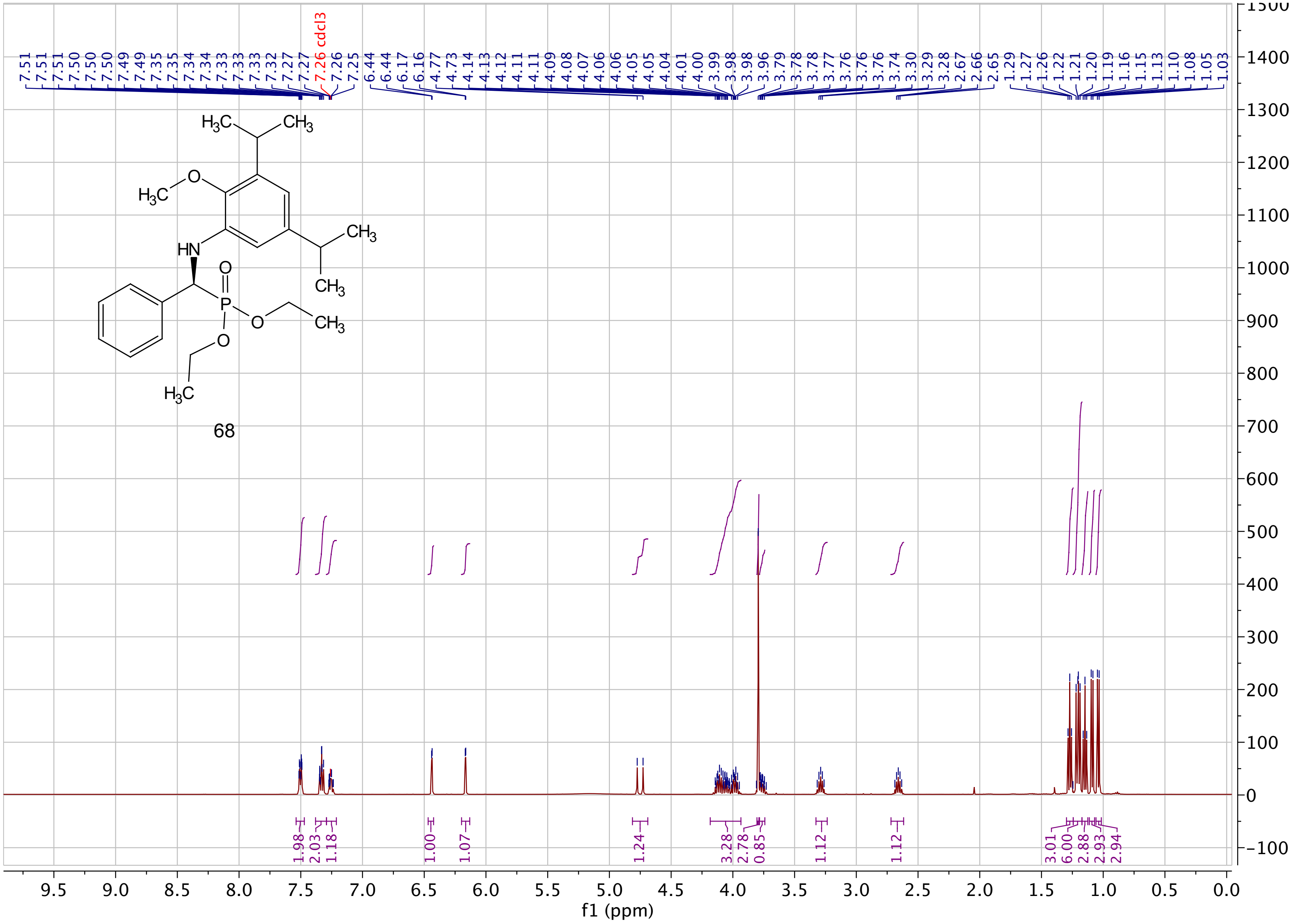
7.51 7.51 7.50 7.50 7.50 7.49 7.49 7.35 7.35 7.34 7.34 7.33 7.33 7.33 7.32 7.27 7.27 7.26 cdcl3 7.26 7.25 6.44 6.44 6.17 6.16 4.77 4.73 4.14 4.13 4.12 4.11 4.11 4.09 4.08 4.07 4.06 4.06 4.05 4.05 4.05 4.04 4.01 4.00 3.99 3.98 3.98 3.96 3.79 3.78 3.78 3.77 3.76 3.76 3.76 3.74 3.30 3.29 3.28 2.67 2.66 2.65 1.29 1.27 1.26 1.22 1.21 1.20 1.19 1.16 1.15 1.13 1.10 1.08 1.05 1.03
H3C
CH3
O
H3C
HN
CH3
O
P
O
CH3
CH3
O
H3C
68
1.98 2.03 1.18 1.00 1.07 1.24 3.28 2.78 0.85 1.12 1.12 3.01 6.00 2.88 2.93 2.94
9.5 9.0 8.5 8.0 7.5 7.0 6.5 6.0 5.5 5.0 4.5 4.0 3.5 3.0 2.5 2.0 1.5 1.0 0.5 0.0
f1 (ppm)
1500 1400 1300 1200 1100 1000 900 800 700 600 500 400 300 200 100 0 -100

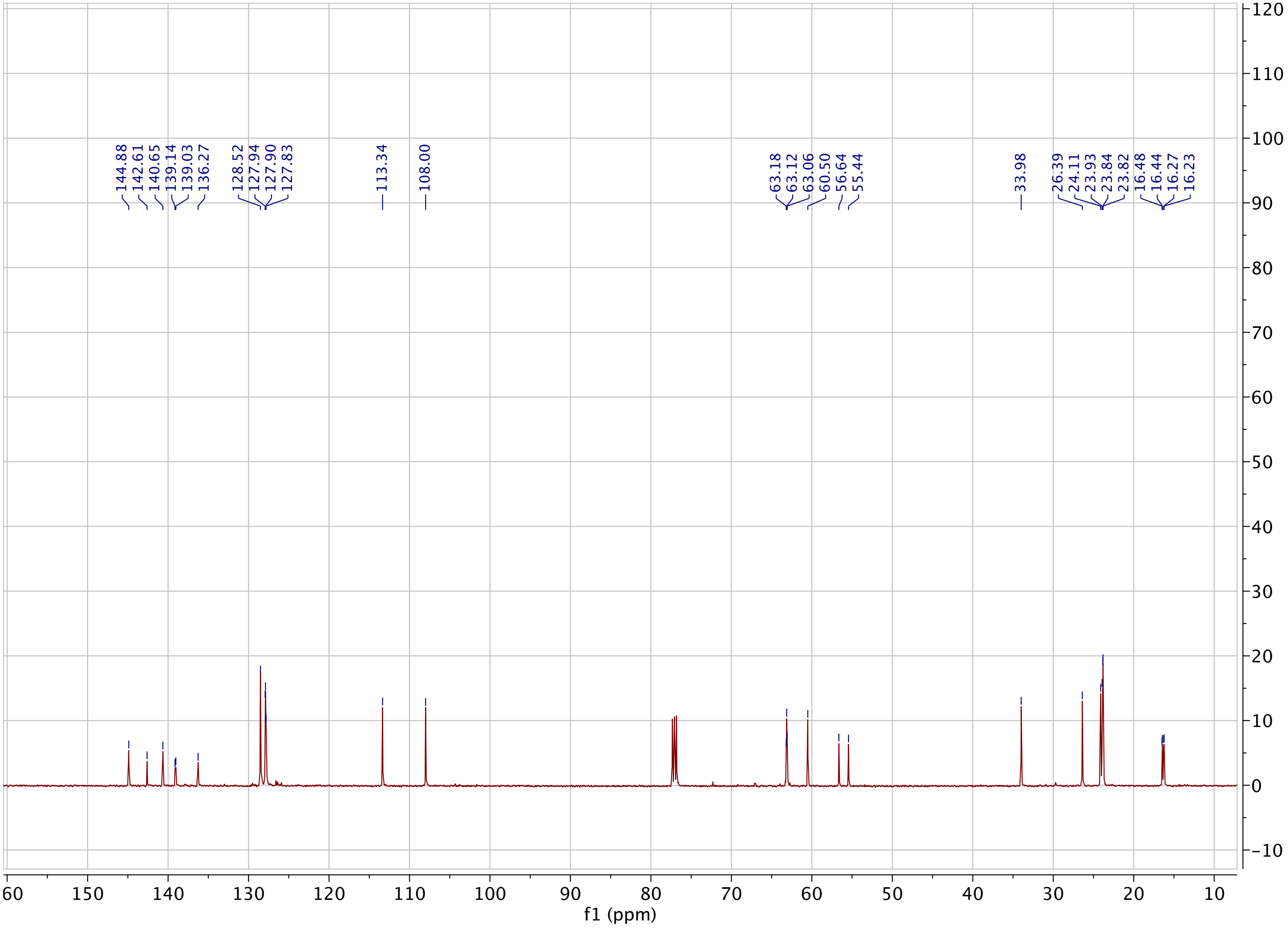

144.88
142.61
140.65
139.14
139.03
136.27
128.52
127.94
127.90
127.83
113.34
108.00
63.18
63.12
63.06
60.50
56.64
55.44
33.98
26.39
24.11
23.93
23.84
23.82
16.48
16.44
16.27
16.23
f1 (ppm)

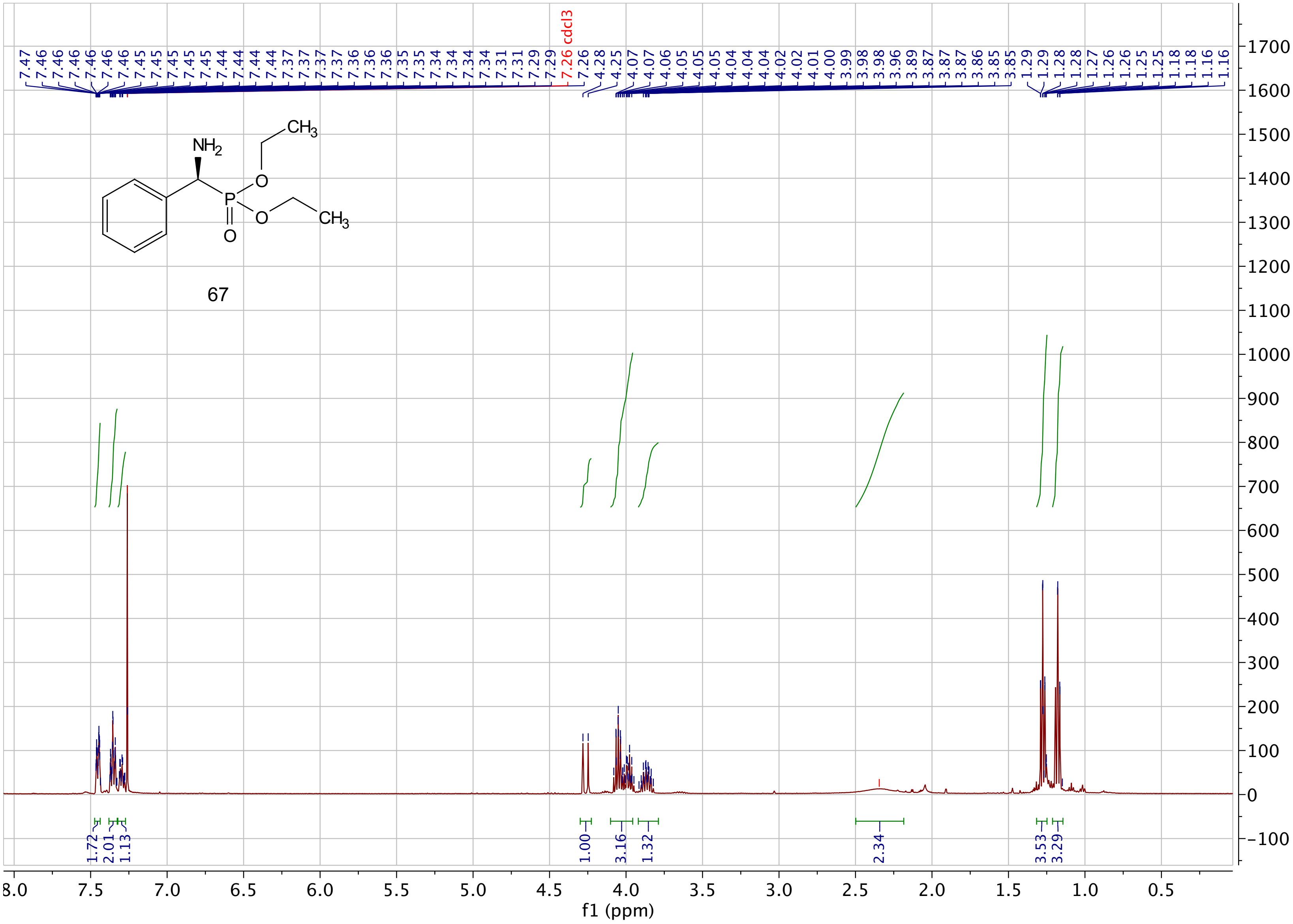

NH2
CH3
O
P
O
CH3
O
67
7.26 cdcl3
1.72
2.01
1.13
1.00
3.16
1.32
2.34
3.53
3.29
f1 (ppm)

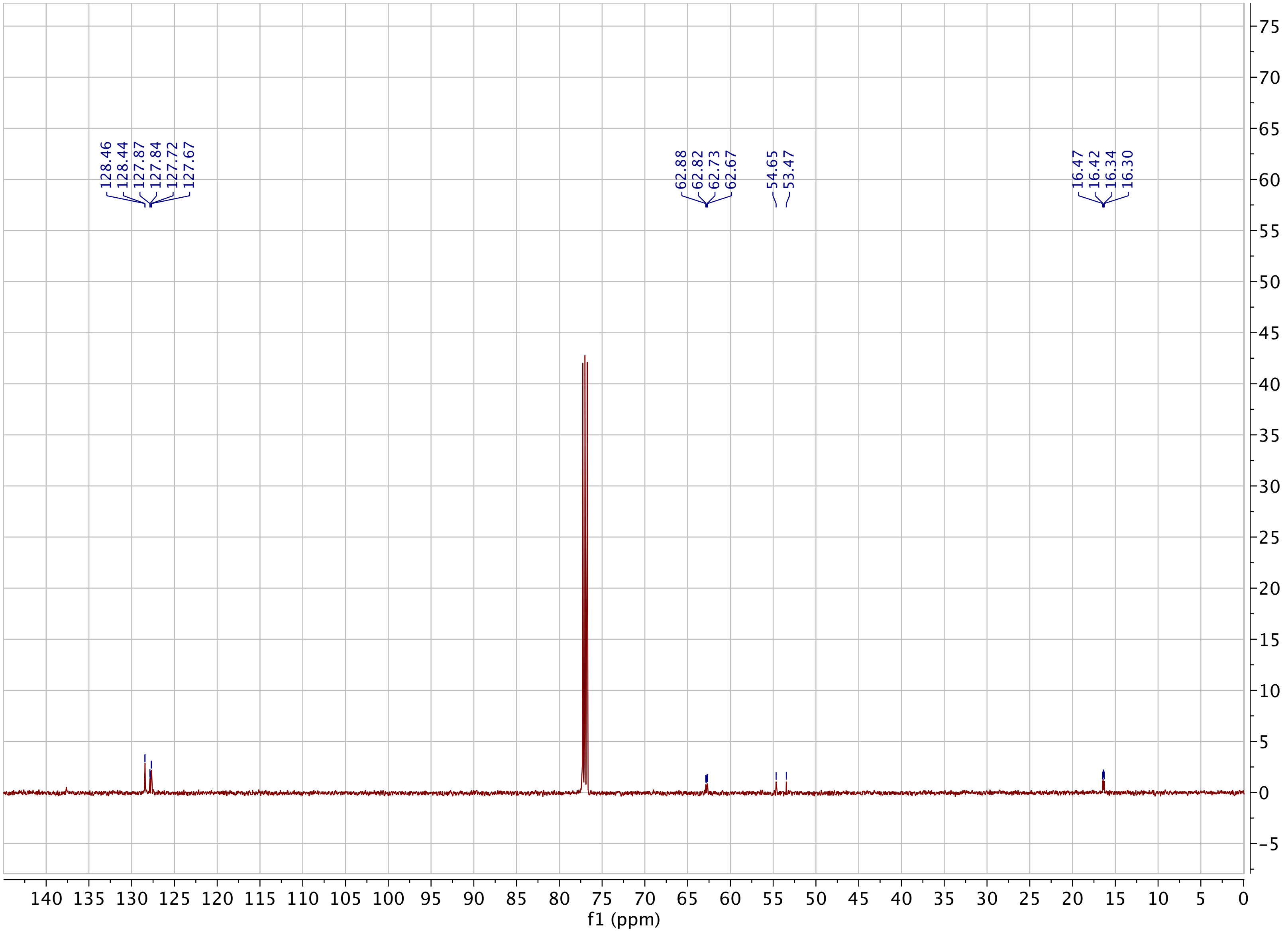
128.46
128.44
127.87
127.84
127.72
127.67
62.88
62.82
62.73
62.67
54.65
53.47
16.47
16.42
16.34
16.30
f1 (ppm)

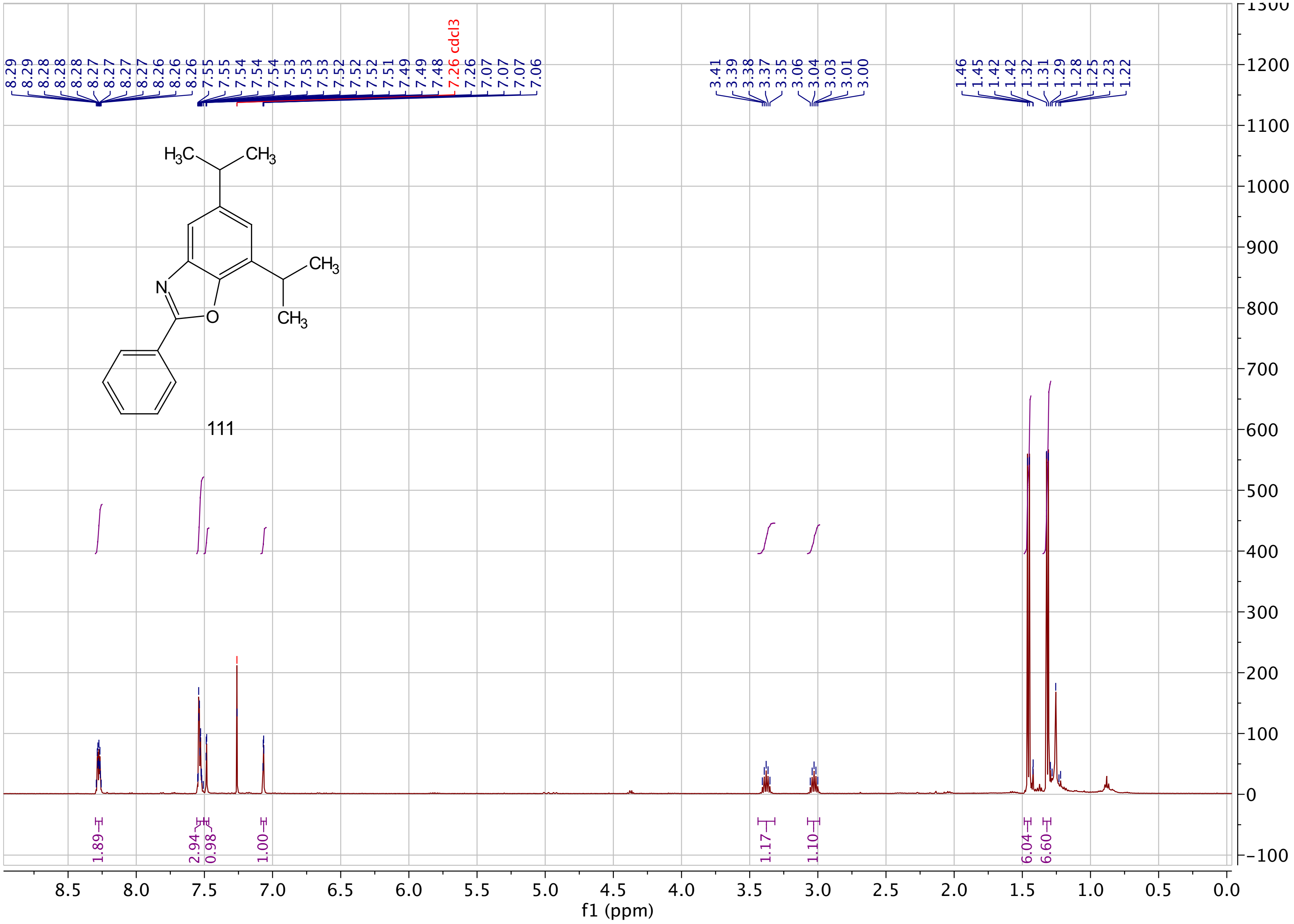

8.29
8.29
8.28
8.28
8.28
8.27
8.27
8.27
8.27
8.26
8.26
8.26
7.55
7.55
7.54
7.54
7.54
7.53
7.53
7.53
7.52
7.52
7.52
7.51
7.49
7.49
7.48
7.26 cdcl3
7.26
7.07
7.07
7.07
7.06
3.41
3.39
3.38
3.37
3.35
3.06
3.04
3.03
3.01
3.00
1.46
1.45
1.42
1.42
1.32
1.31
1.29
1.28
1.25
1.23
1.22
H_3C
CH_3
CH_3
N
O
CH_3
111
1.89
2.94
0.98
1.00
1.17
1.10
6.04
6.60
f1 (ppm)

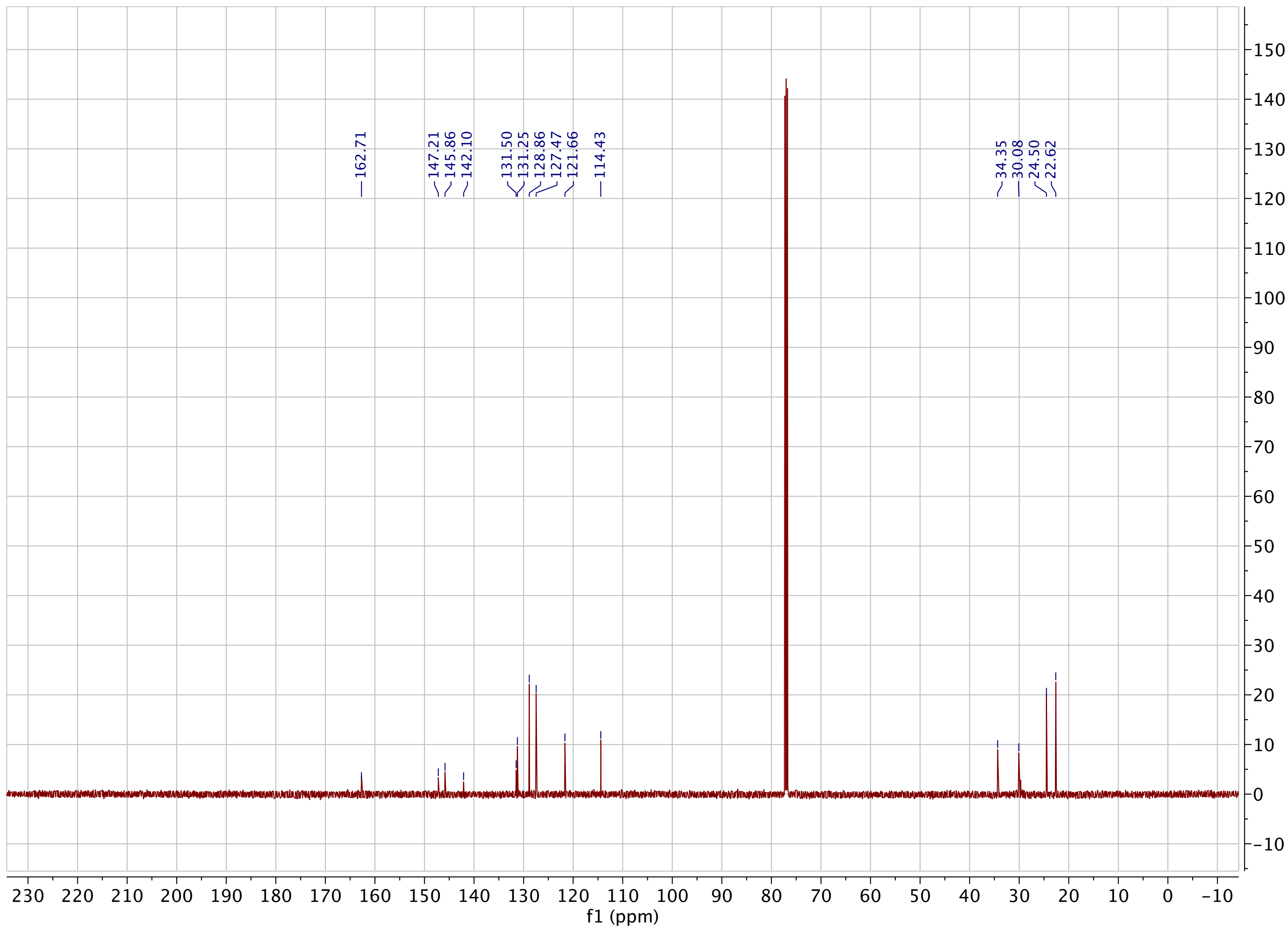
162.71
147.21
145.86
142.10
131.50
131.25
128.86
127.47
121.66
114.43
34.35
30.08
24.50
22.62
f1 (ppm)

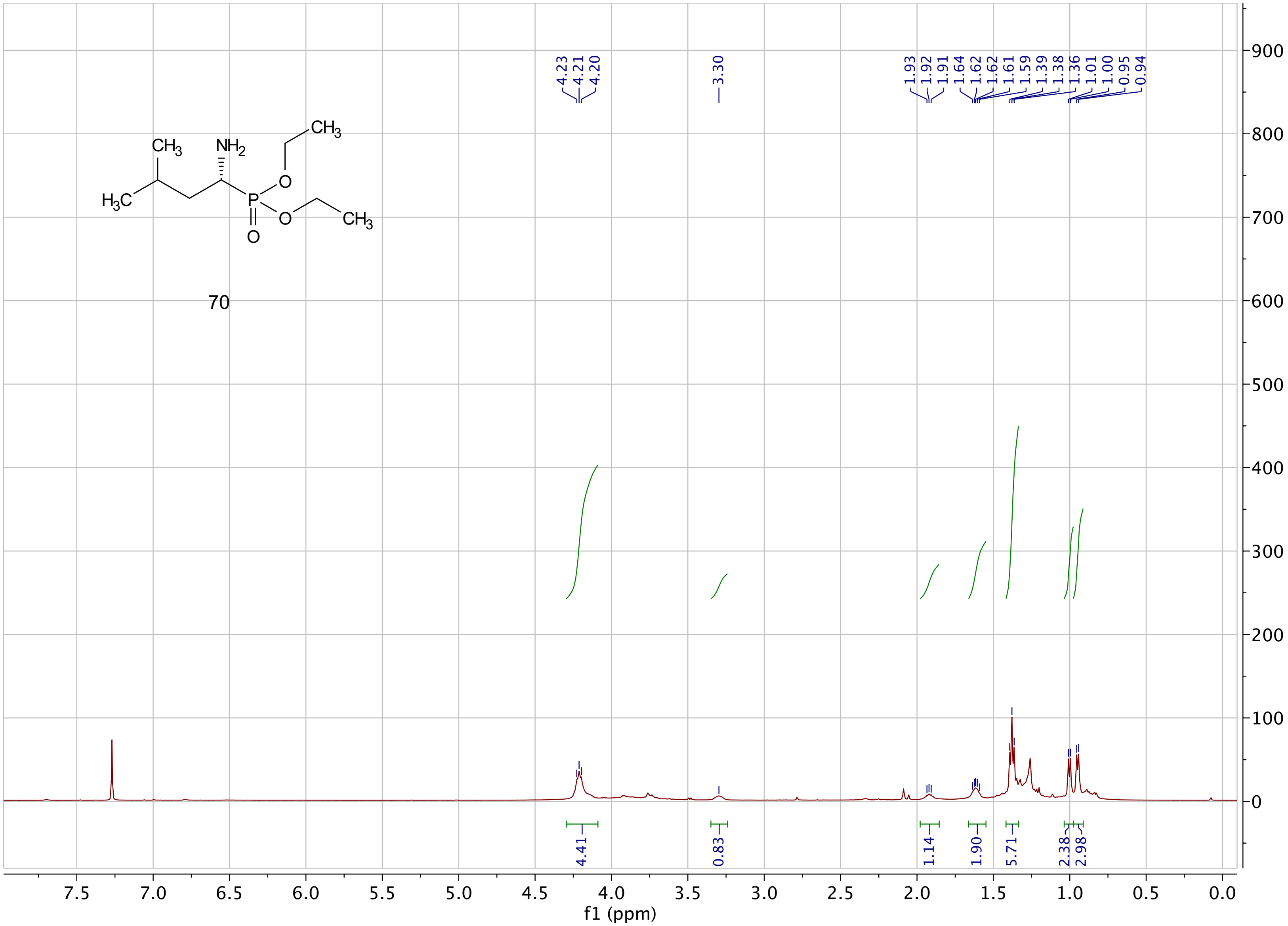
70
4.23
4.21
4.20
3.30
1.93
1.92
1.91
1.64
1.62
1.62
1.61
1.59
1.39
1.38
1.36
1.01
1.00
0.95
0.94
4.41
0.83
1.14
1.90
5.71
2.38
2.98
f1 (ppm)

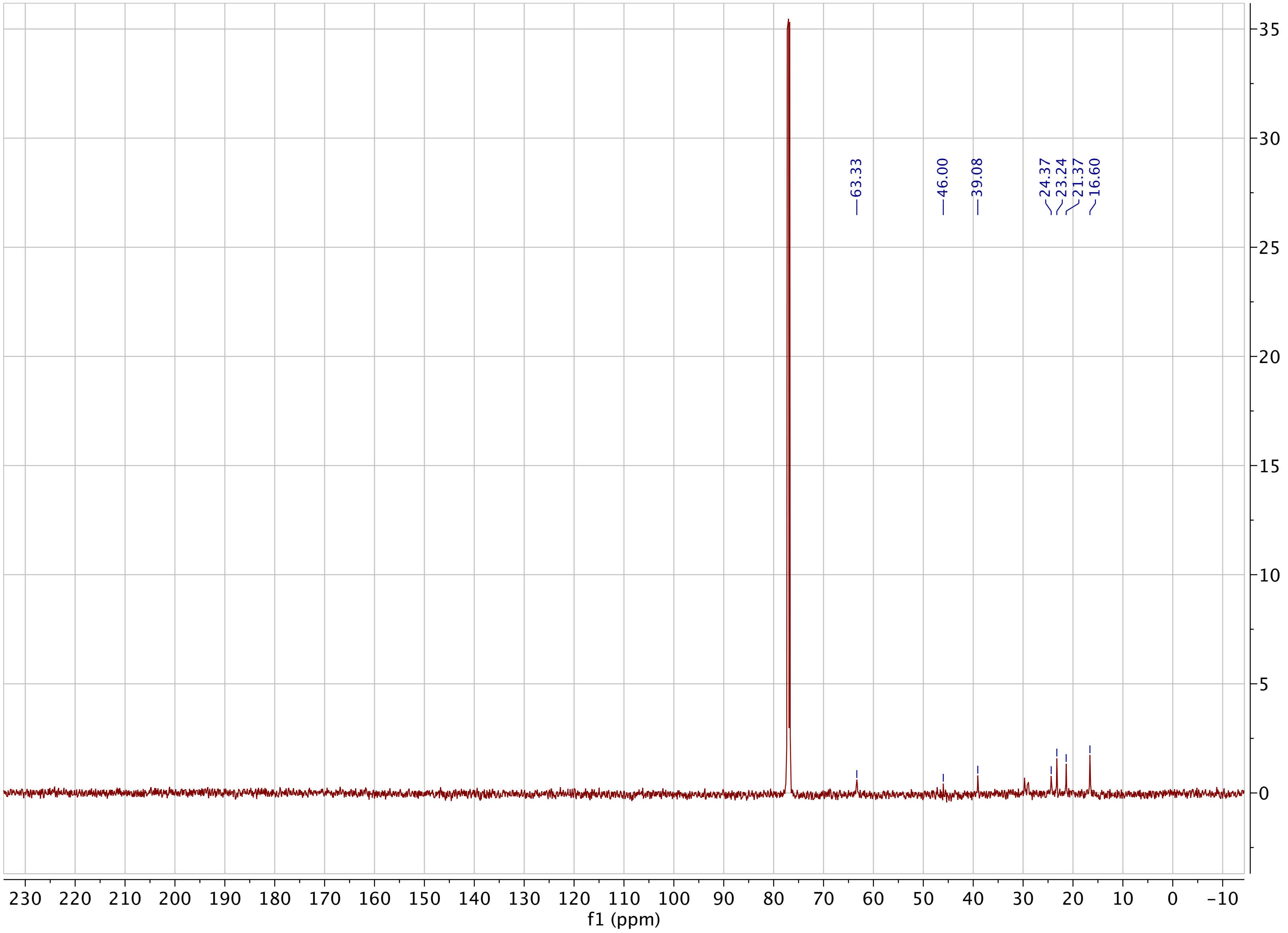

63.33
46.00
39.08
24.37
23.24
21.37
16.60
35
30
25
20
15
10
5
0
230 220 210 200 190 180 170 160 150 140 130 120 110 100 90 80 70 60 50 40 30 20 10 0 –10
f1 (ppm)

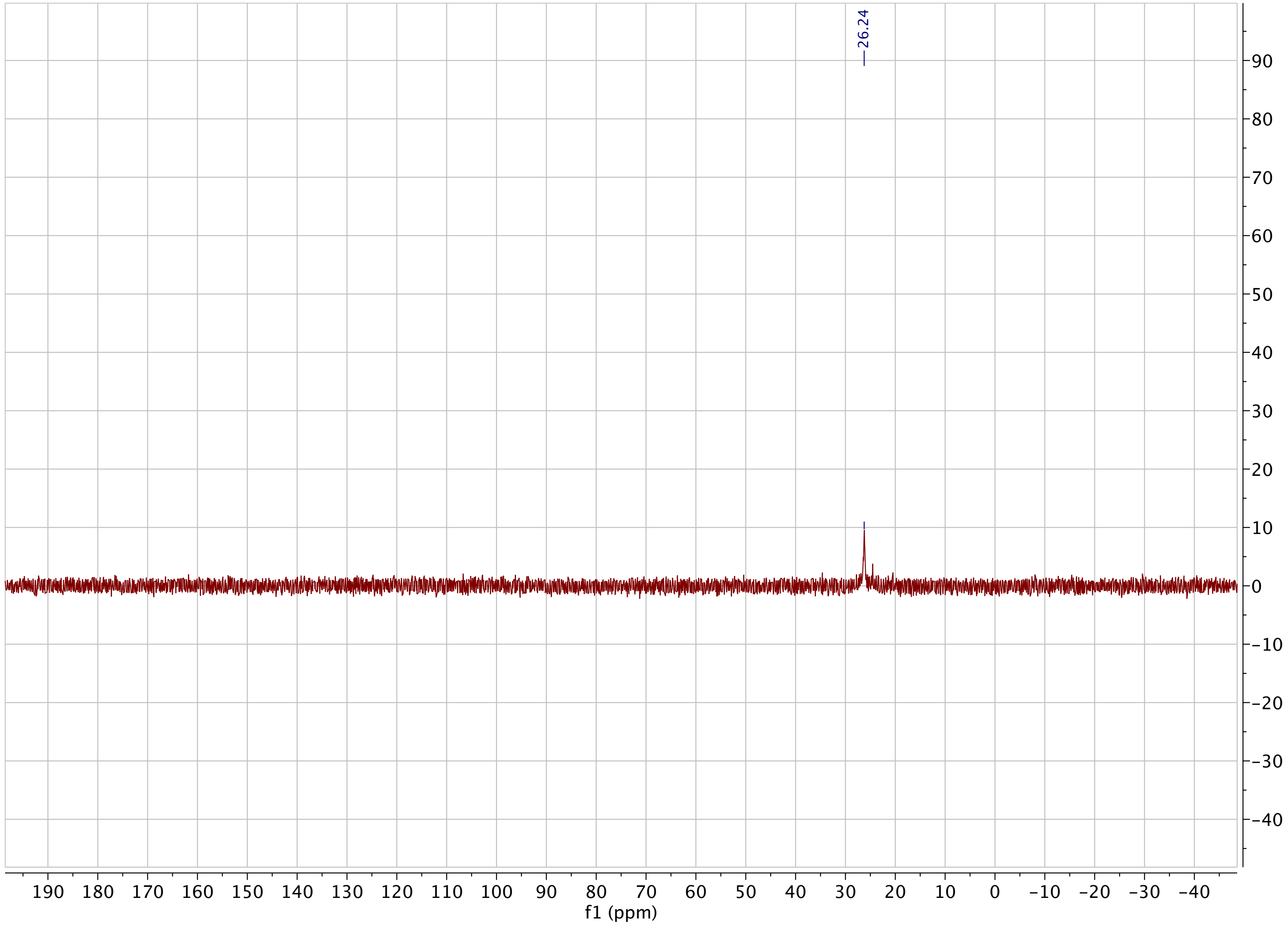
26.24
f1 (ppm)
